# Burden and Trends of Common Oral Disorders Across the Association of Southeast Asian Nations From 1990 to 2021

**DOI:** 10.1016/j.identj.2025.109395

**Published:** 2026-01-28

**Authors:** Yu Cao, Raymond Chung Wen Wong, Pornchai Jansisyanont, Firdaus Hariri, Lisa Rinanda Amir, Michelle Sunico Segarra, Lei Zheng

**Affiliations:** a Faculty of Dentistry, National University of Singapore, Singapore, Singapore; b Department of Oral and Maxillofacial Surgery, Faculty of Dentistry, Chulalongkorn University, Bangkok, Thailand; c Department of Oral and Maxillofacial Clinical Sciences, Faculty of Dentistry, Universiti Malaya, Kuala Lumpur, Malaysia; d Department of Oral Biology, Faculty of Dentistry, Universitas Indonesia, Jawa Barat, Indonesia; e Section of Operative Dentistry, College of Dentistry, University of the Philippines Manila, Manila, Philippines

**Keywords:** Oral health, Dental caries, Periodontal diseases, Edentulism, Global Burden of Disease, Southeast Asia

## Abstract

**Introduction:**

Oral disorders are among the most prevalent diseases globally, yet their burden in Southeast Asia remains poorly characterized. Understanding regional patterns is critical for health planning and prevention.

**Methods:**

We analysed data from the Global Burden of Disease 2021 study to assess the prevalence, incidence, and disability-adjusted life years of oral disorders across the 10 Association of Southeast Asian Nations (ASEAN) member states from 1990 to 2021. Indicators were age-standardized and analysed by sex, age, country, and socio-demographic index.

**Results:**

Oral disorders accounted for nearly half of all-cause prevalence and around 1% of total disease burden in 2021. From 1990 to 2021, prevalent cases rose by over 50% and incident cases by more than 30%, while age-standardized rates showed slight declines. Population growth and ageing were the primary drivers of these increases. Caries of permanent teeth contributed the largest share, followed by periodontal disease and edentulism. Higher socio-demographic index correlated with lower overall burden but a higher proportion of periodontal disease. These findings indicate that oral health remains a major source of morbidity across ASEAN despite modest epidemiologic improvement. Demographic pressures continue to offset gains from prevention and care.

**Conclusions:**

Expanding prevention and primary oral healthcare – including sugar reduction, fluoridation, and sealant or varnish programs – could substantially reduce disability and unmet need. Routine regional surveillance using harmonized indicators is essential to guide equitable policy and investment within the post-2025 ASEAN health agenda.

## Introduction

Oral health is a fundamental component of overall well-being. Common conditions such as dental caries, periodontal disease, and tooth loss affect billions worldwide, causing pain, limiting function, and impairing nutrition and productivity. Oral diseases affect more than 3.5 billion people globally and impose major economic costs.^1^ In 2019, global spending on oral diseases reached $387 billion (∼4.8% of total health expenditure), with productivity losses adding $323 billion.^2^ Crucially, the impact of oral health extends beyond the oral cavity, with growing evidence linking oral disorders to major systemic noncommunicable diseases, including cardiovascular disease,^3^ diabetes mellitus,^4^ and respiratory conditions,^5^ thereby amplifying the overall disease burden. In response, the World Health Organization and FDI World Dental Federation have called for stronger global action.^6^^,^^7^

Despite this burden, oral disorders remain underprioritized compared with other noncommunicable diseases and are leading contributors to disability burden. The Association of Southeast Asian Nations (ASEAN), home to over 670 million people (∼9% of the global population) and the world’s fifth-largest economy,^8^ offers a diverse setting to examine these challenges. Member states range from lower- to high-income economies, such as Cambodia and Laos, to Singapore and Brunei, with rapid growth averaging 4.5% annually.^9^, ^10^, ^11^

The region is undergoing rapid demographic and epidemiologic transitions, yet health priorities have not kept pace. Recent studies utilizing Global Burden of Disease (GBD) data have provided critical insights into the trends and burden of dental caries^12^^,^^13^ as well as the broader spectrum of oral conditions worldwide.^14^ Nevertheless, oral health remains a neglected area within ASEAN, receiving limited policy attention despite its substantial burden. National data are fragmented, and wide disparities persist in health-system capacity and access to care – from Singapore’s high dentist density to persistent shortages in Cambodia and Laos. Notably, oral health was absent from ASEAN’s post-2015 health development agenda.^15^ As the post-2015 period concludes and planning for the post-2025 framework begins, there is an urgent need for robust, comparable data to elevate oral health within regional policy agendas and guide equitable resource allocation.

To address this gap, we analysed data from the GBD 2021 study to quantify the burden of oral disorders in ASEAN from 1990 to 2021. We further examined trends by sex, age, country, and socio-demographic index (SDI) to inform evidence-based policy and guide resource allocation across the region.

## Methods

### Data sources and scope

This study was designed as an observational, descriptive epidemiological analysis utilizing estimates from the GBD 2021 study, accessed via the public GBD Results Tool (https://vizhub.healthdata.org/gbd-results/). GBD 2021 provides harmonized estimates for 371 diseases and 88 risk factors across 204 countries and territories from 1990 to 2021, integrating data from surveys, surveillance systems, clinical records, and published studies.^16^ Detailed analytical methods are described in the IHME technical documentation and recent GBD oral health analyses.^14^

Within GBD 2021, the aggregate category of oral disorders comprises five subtypes: caries of deciduous teeth, caries of permanent teeth, periodontal disease, edentulism, and other oral disorders. Consistent with the GBD hierarchical framework, lip and oral cavity cancer and orofacial clefts were excluded from the present analysis. This exclusion reflects fundamental etiological, clinical, and methodological distinctions. Common oral disorders are predominantly infectious or lifestyle-related conditions, whereas lip and oral cavity cancers are neoplastic, and orofacial clefts are developmental in origin. Clinically and from a health-system perspective, these conditions differ substantially in terms of disease severity, functional impairment, and care complexity. Methodologically, the GBD study classifies lip and oral cavity cancer under ‘Neoplasms’ and orofacial clefts under ‘Congenital birth defects’; maintaining this separation ensures consistency with the parent GBD framework and avoids conflating distinct epidemiological disease clusters.

Regarding specific definitions, caries was defined by clinical evidence of decay or extraction due to caries in primary or permanent dentition. Periodontal disease was defined by severe tissue destruction (eg, CPITN class IV, attachment loss >6 mm, or pocket depth >5 mm), and edentulism as complete loss of natural permanent teeth. ‘Other oral disorders’ included dental, tongue, and jaw conditions not otherwise classified (eg, temporomandibular disorders and developmental anomalies). Corresponding ICD code ranges are listed in Appendix Table 1.

We extracted estimates for the ASEAN region and its 10 member states – Brunei, Cambodia, Indonesia, Laos, Malaysia, Myanmar, the Philippines, Singapore, Thailand, and Viet Nam – for the period 1990 to 2021. Indicators included prevalence, incidence, and disability-adjusted life years (DALYs), each expressed as age-standardized rates per 100,000 population, absolute counts, and proportions of all-cause totals.

### Socio-demographic index

Burden gradients were assessed using the SDI, a composite measure based on income per capita, average educational attainment, and total fertility rate. SDI values range from 0 to 1, with higher scores indicating greater socio-demographic development. GBD classifies countries into five SDI quintiles (low, low-middle, middle, high-middle, and high).^14^ We used country-year SDI data from the GBD 2021 dataset.

### Statistical analysis

We analysed temporal trends in prevalence, incidence, and DALYs for ASEAN and each member state from 1990 to 2021, stratified by sex and age group. Percentage changes between 1990 and 2021 were calculated for both age-standardized rates and absolute counts.

To identify drivers of change, we decomposed net differences in case counts into contributions from population growth, population ageing, and age-specific rate change (epidemiologic change) using 5-year age groups and equal allocation of interaction terms.^17^ Positive values indicate increases attributable to a factor, and negative values indicate reductions.

Associations between oral disease burden and SDI were examined using Spearman rank correlation (*ρ*) and locally weighted scatterplot smoothing (LOWESS). Statistical significance was defined as *P* < .05.

## Results

### Prevalence

**Fig. 1:**
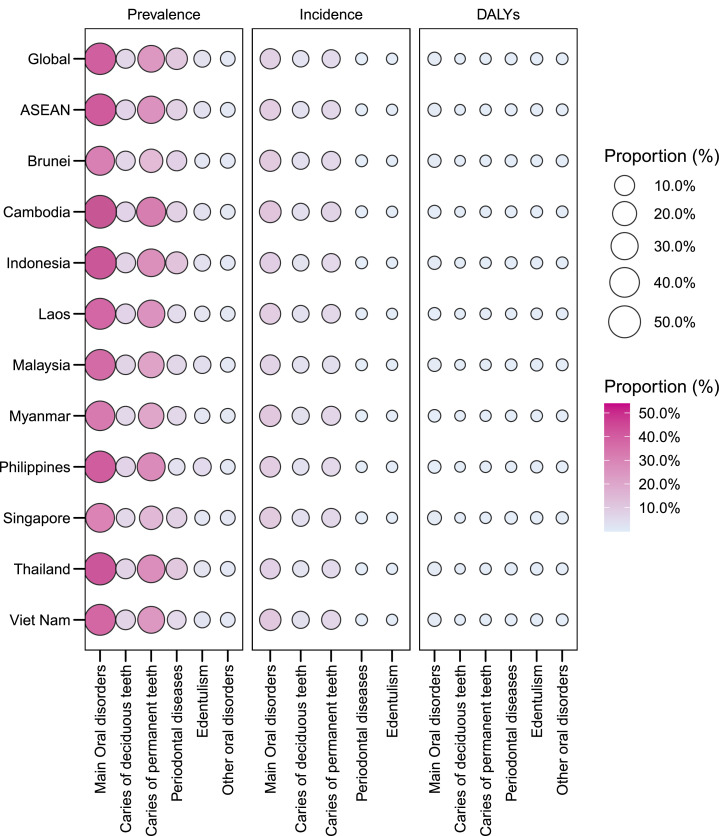
Proportion of oral disorders in total prevalence, incidence, and DALYs at the global and ASEAN levels, 2021.

In 2021, oral disorders constituted a major component of the disease burden in ASEAN (Appendix Table 2; Figure 1), accounting for 48.8% (44.5-53.2) of all-cause prevalence, slightly above the global average (47.7% [44.0-51.6]). Proportions exceeded 50% in Cambodia (53.99%), Indonesia (52.27%), and Thailand (51.14%), while lower shares were observed in Singapore (36.40%), Brunei (37.04%), and Myanmar (39.41%).

**Fig. 2:**
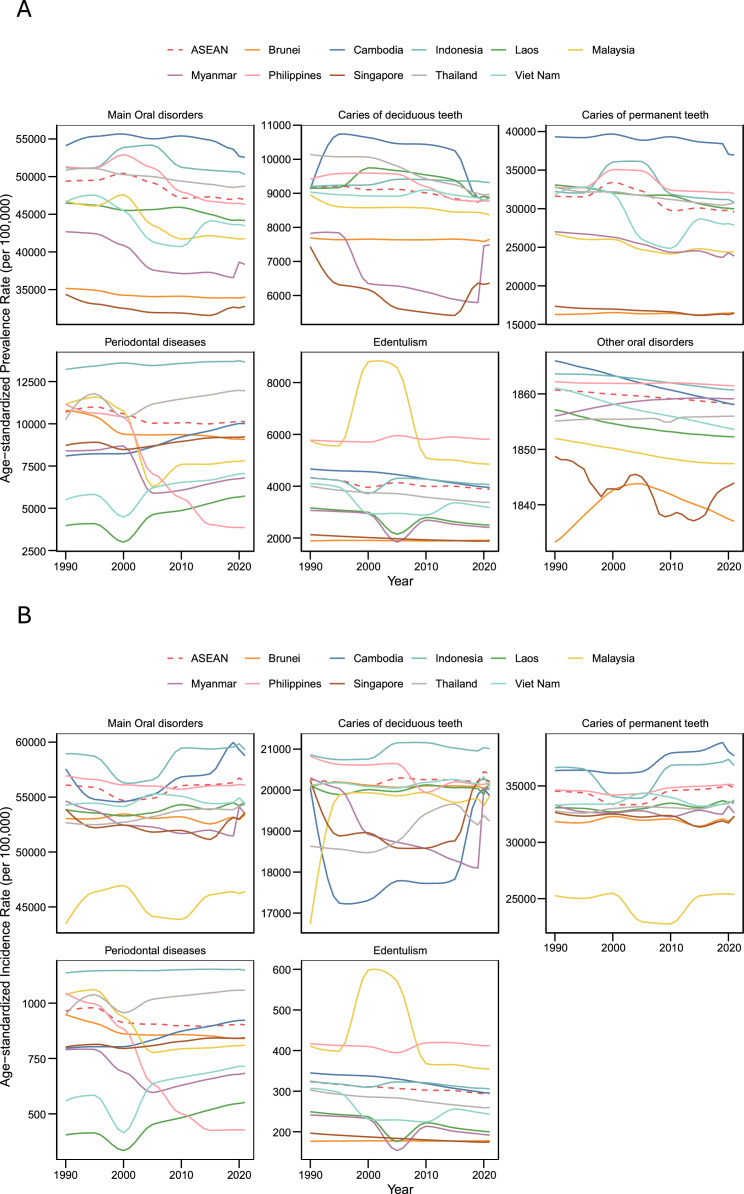
Trends in age-standardized rates of (A) prevalence, and (B) incidence for oral disorders in ASEAN countries, 1990 to 2021.

**Fig. 3:**
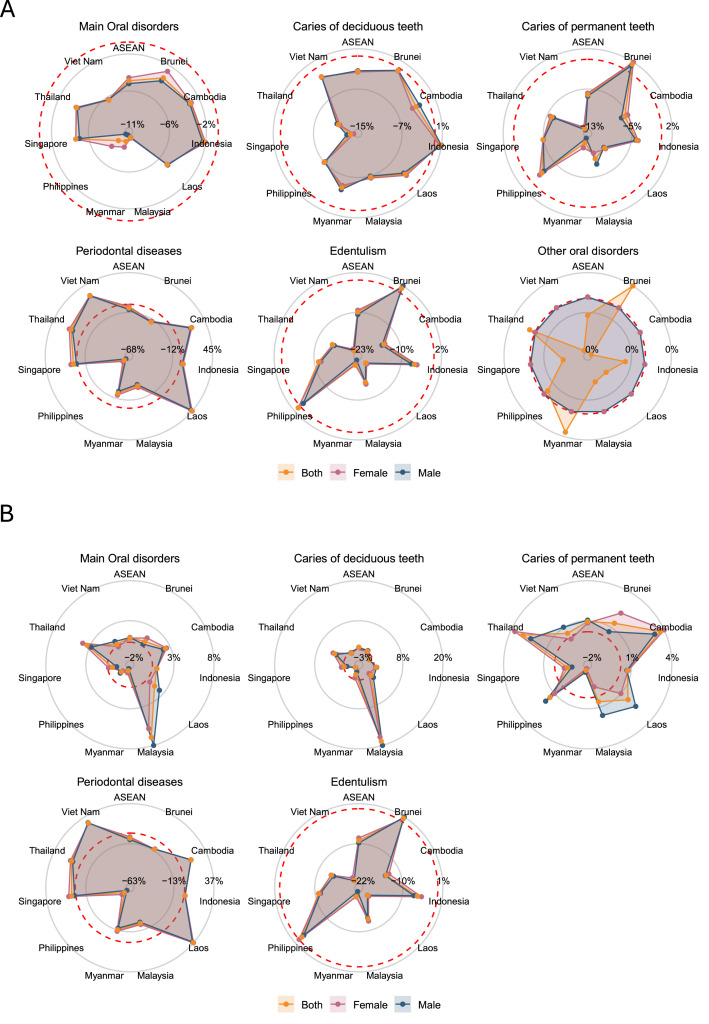
Changes in age-standardized rates of (A) prevalence and (B) incidence for oral disorders by country and sex in ASEAN, 1990 to 2021.

Prevalent cases rose from 209.6 million (188.5-233.4) in 1990 to 319.6 million (290.1-350.8) in 2021, a 52.5% (47.3-58.7) increase. Despite this growth, the age-standardized prevalence rate (ASPR) declined by 5.0% (–7.0 to –2.9), from 49,385.3 (44,775.4-54,107.5) to 46,931.2 (42,762.9-51,177.3) per 100,000 (Appendix Table 3; Figures 2A and 3A).

**Fig. 4:**
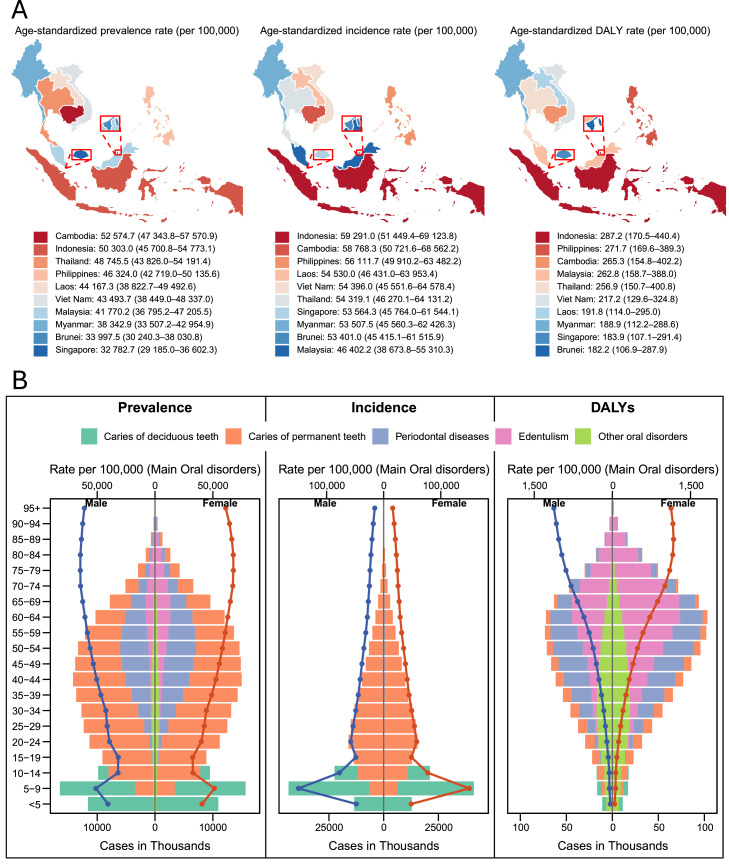
(A) Geographic distribution of age-standardized prevalence, incidence, and DALY rates (per 100,000 population) for oral disorders across ASEAN countries in 2021. (B) Age- and sex-specific distributions of prevalence, incidence, and DALYs for oral disorders in ASEAN, 2021. Bars represent age-specific counts, and lines indicate rates per 100,000 population.

At the country level (Appendix Table 3; Figures 2A, 3A, 4A), Indonesia had the highest prevalence count in 2021 (140.8 million [127.1-153.8]), followed by the Philippines (50.8 million [46.5-55.4]) and Viet Nam (44.0 million [38.5-49.1]). All countries recorded increases in count, ranging from +26.9% (16.4-37.8) in Thailand to +97.6% (76.9-122.2) in Singapore. ASPRs declined across all countries, most notably in Myanmar (–10.2%), Malaysia (–10.7%), and Viet Nam (–6.7%), while Indonesia showed only a slight reduction (–1.7%).

By condition (Appendix Table 3), caries of permanent teeth dominated (209.0 million [178.3-244.5]), followed by periodontal disease (73.2 million [60.6-86.4]), deciduous-teeth caries (50.7 million [42.0-60.5]), and edentulism (24.3 million [20.8-28.6]). From 1990 to 2021, cases increased for permanent-teeth caries (+58.1%), periodontal disease (+113.2%), and edentulism (+134.3%), but declined slightly for deciduous-teeth caries (–2.8%). In 2021, ASPRs were highest for permanent-teeth caries (29,577.9 [25,319.9-34,442.3]), followed by periodontal disease (10,122.7 [8449.1-11,880.1]), deciduous caries (8868.4 [7352.1-10,518.9]), and edentulism (3888.3 [3346.8-4557.2]) per 100,000 (Figures 2A and 3A).

Across ages (Appendix Table 4; Figure 4B), prevalent cases peaked in adults aged 25 to 44 years, while age-specific rates rose with age and plateaued at ∼65,000 to 68,000 per 100,000 among those aged 70 to 79. Patterns were similar between sexes, with slightly higher rates in females.

### Incidence

In 2021, oral disorders contributed substantially to the overall disease-onset burden in ASEAN (Appendix Table 2; Figure 1), accounting for 11.3% (9.9-13.1) of all-cause incidence – above the global average (10.4% [9.2-11.7]). Proportions exceeded 13% in Cambodia (13.7%) and Viet Nam (13.0%), with similarly high shares in Myanmar (13.0%), Brunei (12.5%), and Singapore (12.4%). Lower proportions were found in Malaysia (9.5%) and Thailand (10.1%).

Incident cases rose from 278.5 million (237.1-330.8) in 1990 to 372.2 million (325.2-430.0) in 2021 (+33.7% [28.0-39.2]). However, the age-standardized incidence rate (ASIR) remained nearly unchanged (+0.6% [–1.2 to +2.5]), rising slightly from 56,085.3 (48,474.3-65,369.6) to 56,404.0 (48,936.6-65,459.6) per 100,000 population (Appendix Table 3; Figures 2B and 3B).

At the country level (Appendix Table 3; Figures 2B, 3B, 4A), Indonesia had the largest number of new cases in 2021 (161.2 million [140.5-186.8]), followed by the Philippines (66.0 million [58.5-74.8]) and Viet Nam (52.9 million [44.8-62.4]). All countries except Thailand (–3.1% [–12.2 to +6.3]) showed rising incidence counts, ranging from +24.0% (17.2-31.4) in Myanmar to +65.5% (52.1-84.6) in Malaysia and +97.6% in Singapore. ASIRs remained largely stable, with slight declines in Myanmar (–2.1%), Philippines (–1.5%), and Singapore (–0.6%), and modest increases in Thailand (+3.1%) and Malaysia (+6.8%).

Across age groups (Appendix Table 4; Figure 4B), incidence peaked during school age to early adulthood (5-24 years). Age-specific rates reached ∼150,000 per 100,000 at 5 to 9 years, reflecting contributions from both deciduous and permanent caries, then declined steadily through adulthood to <25,000 beyond age 70. Patterns were consistent between sexes, with minimal differences across ages.

### DALYs

Oral disorders accounted for a small but notable share of the total disease burden in ASEAN, contributing 0.77% (0.48-1.12) of all-cause DALYs – comparable to the global average of 0.80% (0.51-1.17) (Appendix Table 2; Figure 1). Proportions exceeded 1% in Singapore (1.27%) and Thailand (1.01%), while lower shares were recorded in Laos (0.45%) and Myanmar (0.45%).

DALY counts nearly doubled from 0.89 million (0.51-1.38) in 1990 to 1.75 million (1.06-2.64) in 2021, an increase of 97.2% (87.3-110.0) (Appendix Table 3). Despite this rise, the age-standardized DALY rate (ASDR) declined by 6.4% (–11.1 to –0.2), from 275.5 (163.2-414.6) to 257.9 (157.0-385.7) per 100,000 (Figures S1 and S2).

At the country level (Appendix Table 3; Appendix Figures 1 and 2; Figure 4A), Indonesia contributed the highest DALY count in 2021 (785.8k [460.1-1231.3]), followed by the Philippines (254.7k [160.0-368.6]) and Thailand (239.3k [140.1-370.4]). All countries recorded increases, most notably in Singapore (+171.9%), Cambodia (+122.4%), and Malaysia (+110.0%), while Myanmar showed a smaller rise (+52.2%). ASDRs declined in most countries, especially Malaysia (–16.0%), the Philippines (–14.6%), and Myanmar (–14.2%), with minor reductions in Thailand (–3.1%) and Indonesia (–1.8%).

Across ages (Appendix Table 4; Figure 4B), DALY rates rose steadily with age, from ∼40 per 100,000 in early childhood (<5 years) to >1000 per 100,000 beyond age 70. Rates were slightly higher in females, particularly at older ages.

### Decomposition of changes in oral disorder burden

**Fig. 5:**
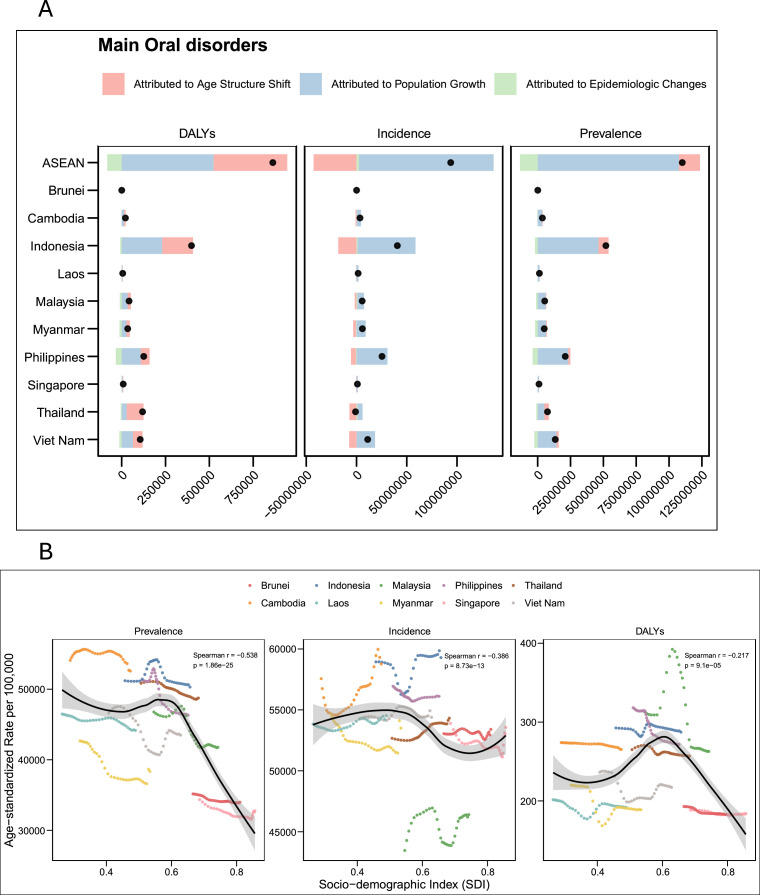
(A) Decomposition of changes in DALYs, incidence, and prevalence of oral disorders in ASEAN countries, 1990 to 2021. Bars show the contributions of population growth (blue), age-structure shift (red), and epidemiologic changes (green) to total change, with black dots representing the net change. (B) Associations between age-standardized prevalence, incidence, and DALY rates of oral disorders and the socio-demographic index (SDI) in ASEAN countries, 1990 to 2021. Each point represents country-year data, with the black line showing the fitted LOWESS curve and the shaded area indicating the 95% confidence band.

Decomposition analysis (Appendix Table 5; Figure 5A) showed that the increase in oral disorder burden across ASEAN was primarily driven by population growth, with smaller effects from ageing and modest offsets from epidemiologic improvements. For prevalence, population growth contributed +97.7%, ageing +14.6%, and epidemiologic improvement –12.3%. For incidence, population growth accounted for +143.0%, ageing –45.6%, and epidemiologic change +2.6%. For DALYs, both population growth (+60.8%) and ageing (+48.8%) dominated, while epidemiologic improvement partly reduced the total (–9.6%).

Across countries, population growth was the leading contributor to burden increases, while the effect of ageing varied depending on demographic structure, and epidemiologic change was generally negative. Patterns were largely similar across oral conditions; however, caries of deciduous teeth showed the opposite trend, with a net decline in burden driven by a shrinking child population and ageing exerting a reverse effect, along with small contributions from epidemiologic improvement (Appendix Table 5).

### Association between oral disorder burden and socio-demographic development

Correlation analyses across ASEAN countries (Figure 5B) revealed clear gradients between the SDI and oral disorder burden. For main oral disorders, age-standardized prevalence (*ρ* = –0.538, *P* = 1.86 × 10^–^²⁵), incidence (*ρ* = –0.386, *P* = 8.73 × 10^–^¹³), and DALYs (*ρ* = –0.217, *P* = 9.1 × 10^–^⁵) were negatively correlated with SDI.

Notably, by condition (Appendix Figures 3-7), all oral diseases except periodontal disease showed negative correlations with SDI. In contrast, periodontal disease demonstrated positive correlations for prevalence (*ρ* = 0.346, *P* = 2.07 × 10^–^¹⁰), incidence (*ρ* = 0.349, *P* = 1.37 × 10^–^¹⁰), and DALYs (*ρ* = 0.348, *P* = 1.59 × 10^–^¹⁰).

## Discussion

### Main findings

Oral disorders represent a major source of morbidity in ASEAN, accounting for nearly half of all-cause prevalence in 2021. They remain widespread, predominantly nonfatal contributors to health loss across the region.^6^ From 1990 to 2021, prevalent and incident cases increased markedly, while age-standardized rates declined modestly, reflecting demographic expansion and population ageing rather than worsening risk.^6^

Caries of permanent teeth remained the largest contributor, whereas sharp rises in periodontitis and edentulism highlight the effects of ageing and limited rehabilitation access. The slight decline in deciduous teeth caries likely reflects demographic ageing and a smaller proportion of young children, rather than major advances in early-childhood prevention. Periodontal disease continues to cause significant disability where maintenance care is limited.^18^

Substantial cross-country variation was observed. Indonesia carried the highest absolute burden due to its large population, whereas several middle-income countries – notably Malaysia, Viet Nam, and Myanmar – achieved notable rate reductions, reflecting advances in oral-health coverage and health system capacity, in line with WHO reporting.^6^^,^^18^

### Rates vs counts and decomposition

The divergence between rising counts and stable or declining rates was primarily demographic. Decomposition analysis indicated population growth as the main driver of increases, with ageing contributing secondarily and epidemiologic improvement partly offsetting these effects. Growth and ageing dominated changes in prevalence and DALYs, whereas incidence was shaped by younger-age concentration, consistent with the accumulation of chronic conditions in prevalence vs new onset in incidence.^6^

### Age profile

The age pattern followed a predictable sequence: caries dominated in childhood, permanent-teeth caries persisted through adulthood, periodontal disease increased from midlife, and edentulism concentrated in older age. The incidence peak at 5 to 9 years reflected mixed dentition and the high susceptibility of newly erupted first molars (∼6-7 years), influenced by enamel immaturity, deep pits and fissures, and plaque retention.^19^^,^^20^ Additional risks include limited brushing ability, high sugar intake in school settings, and uneven coverage of sealants or fluoride varnish.^21^^,^^22^ From midlife, periodontal burden rises with cumulative plaque exposure, shared risks such as diabetes and smoking, and immune ageing (immunosenescence and inflammaging).^23^ Greater tooth retention into older age may sustain measured periodontitis burden, as more natural teeth remain at risk.^24^ In later life, functional decline and comorbidities can further impair prosthesis use and maintenance, prolonging disability duration.

### Sex differences

Sex differences are nuanced. After age-standardization, prevalence and incidence are similar by sex overall, but women show higher ASDRs for all oral disorders in 2021, consistent with longer female longevity and more years lived with disability across the life course.^6^ For periodontitis in ASEAN, female ASPR and ASDR are slightly higher in 2021, whereas studies from other settings (eg, US NHANES 2009-2012) report higher male burden, indicating heterogeneity in risk, tooth retention, and ascertainment.^25^^,^^26^ Behaviour also differs: men report poorer oral-hygiene practices, fewer dental visits, and more problem-oriented care-seeking, which can widen disability gaps even when incidence and prevalence are similar.^27^

### Socio-demographic gradients

SDI gradients were strong and condition-specific. For all oral disorders combined, SDI correlated inversely with ASPR, ASIR, and ASDR, most sharply for prevalence. Permanent-teeth caries showed the clearest inverse gradient, while deciduous-teeth caries had inverse patterns for prevalence and DALYs but flatter incidence. Edentulism showed weak inverse gradients, whereas periodontitis correlated positively with SDI. These differences likely reflect variations in sugar exposure, preventive coverage, access, and care quality rather than age structure.^18^^,^^21^

After adjustment, higher SDI remained associated with lower rates for most oral disorders, reflecting stronger prevention and primary care.^6^ The inverse pattern for permanent-teeth caries aligns with sugar policies, fluoride exposure, and school-based sealant delivery.^28^^,^^29^ The flatter incidence for deciduous-teeth caries may reflect better detection during mixed dentition.^28^^,^^29^ Edentulism’s weak inverse gradient likely reflects improved restorative care and prosthetic access.^6^ In contrast, periodontitis increased with SDI, consistent with greater tooth retention, diagnostic ascertainment, and shared risks such as smoking and diabetes.^30^, ^31^, ^32^ These mechanisms align with WHO’s framework integrating oral health within UHC and PHC.^18^^,^^21^

### Policy and practice implications

Actionable levers include expanding water fluoridation^33^ and scaling sealant or varnish programs within essential benefit packages.^22^^,^^28^ ASEAN countries such as the Philippines^34^ and Malaysia^35^ have adopted sugar-sweetened beverage taxes, offering upstream opportunities for risk reduction. Singapore provides a model of sustained nationwide fluoridation since the 1950s.^36^ A life-course oral health package – emphasizing sugar reduction, fluoride exposure, preventive care, and rehabilitation across all ages – aligns with the WHO Global Oral Health Action Plan 2023 to 2030.^18^

### Limitations

This study is based on the GBD 2021 framework, and the accuracy of estimates depends on the availability and quality of underlying data. Uncertainty remains for countries or subnational areas with limited surveillance, diagnostic capacity, or reporting consistency. GBD modelling assumptions – such as the redistribution of nonspecific causes and covariate-based imputations – may also influence burden estimates. Oral cancer and cleft lip/palate were excluded, as they are classified under neoplasms and congenital anomalies within the GBD hierarchy, outside the scope of nonneoplastic, noncongenital oral disorders.

Despite these limitations, the study’s strengths include harmonized case definitions, consistent analytic methods over three decades, comprehensive coverage of all ASEAN countries, and a decomposition framework that distinguishes demographic from epidemiologic change – aligned with WHO reporting principles.^6^

## Conclusions

Prevalent and incident cases and DALYs rose sharply from 1990 to 2021 despite small declines in age-standardized rates, with demographic change as the primary driver. This underscores the importance of routine monitoring using harmonized indicators, disaggregated by sex, age, and socio-demographic development, so that investments can be prioritized in settings or subpopulations where epidemiological improvements have not kept pace with demographic momentum.

## Author contributions

Yu Cao: Contributed to conception and design; contributed to data acquisition, analysis, and interpretation; drafted and critically revised the manuscript. Raymond C. W. Wong: Contributed to conception; contributed to data interpretation; critically revised the manuscript for important intellectual content. Pornchai Jansisyanont, Firdaus Hariri, Lisa Rinanda Amir, and Michelle C. Sunico-Segarra: Contributed to data interpretation; critically revised the manuscript for important intellectual content. Lei Zheng: Contributed to conception and design; contributed to data interpretation; drafted and critically revised the manuscript. All authors gave their final approval and agree to be accountable for all aspects of the work.

## Declaration of generative AI and AI-assisted technologies in the writing process

During the preparation of this manuscript, the authors used *ChatGPT (OpenAI, GPT-5 model)* to assist in language editing and refinement of grammar and phrasing. The authors reviewed and verified all content for accuracy and scientific integrity, and take full responsibility for the final version of the manuscript.

## Conflict of interest

The authors declare no conflicts of interest.

## Appendix Supplementary materials

Image, application 1
