# Supplementary material for: Burden and Trends of Common Oral Disorders Across the Association of Southeast Asian Nations From 1990 to 2021
Source: Int Dent J. 2026 Jan 28;76(2):109395. doi: 10.1016/j.identj.2025.109395 (PMC12873728; doi:10.1016/j.identj.2025.109395)
Supplement: Supplementary file 1 [file mmc1.docx]

**Appendix List**

**Appendix Figure 1.** Trends in age-standardized DALY rates for total oral disorders in ASEAN countries, 1990–2021.

**Appendix Figure 2.** Changes in age-standardized DALY rates for oral disorders by country and sex in ASEAN, 1990–2021.

**Appendix Figures 3–7.** Associations between age-standardized (A) prevalence, (B) incidence, and (C) DALY rates of oral disorders and the Socio-demographic Index (SDI) in ASEAN countries, 1990–2021. Each point represents country-year data; the black line shows the fitted LOWESS curve and the shaded area indicates the 95% confidence band. Negative Spearman correlations (ρ = −0.538 for prevalence, −0.386 for incidence, and −0.217 for DALYs) indicate that oral disorder burden generally decreases with increasing SDI, reflecting socioeconomic gradients across the region.
  **Figure 3.** Caries of deciduous teeth
  **Figure 4.** Caries of permanent teeth
  **Figure 5.** Periodontal disease
  **Figure 6.** Edentulism
  **Figure 7.** Other oral disorders

**Appendix Table 1.** Definition and classification of oral disorders analyzed in this study, including case definitions, ICD-10 codes, and mapping to the GBD 2021 cause hierarchy.

**Appendix Table 2.** Percent contribution of oral disorders to all-cause prevalence, incidence, and disability-adjusted life years (DALYs) in 2021.

**Appendix Table 3.** Number and age-standardized rate of oral disorders by sex and country, 1990 and 2021.

**Appendix Table 4.** Number and rate of oral disorders by age group, sex, and country in 2021.

**Appendix Table 5.** Decomposition of changes in oral disorder burden between 1990 and 2021.


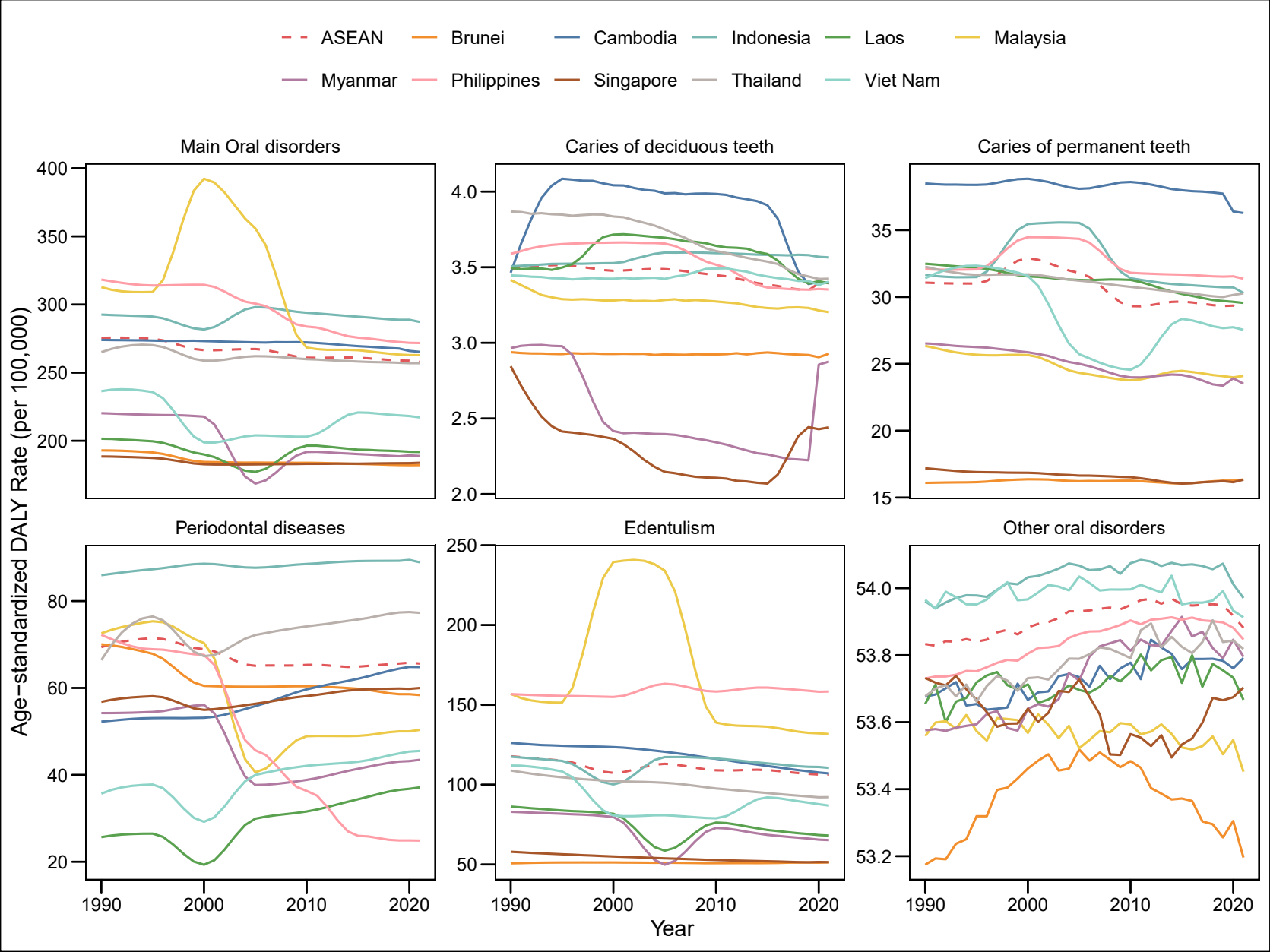


**Appendix Figure 1.** Trends in age-standardized DALY rates for total oral disorders in ASEAN countries, 1990–2021.


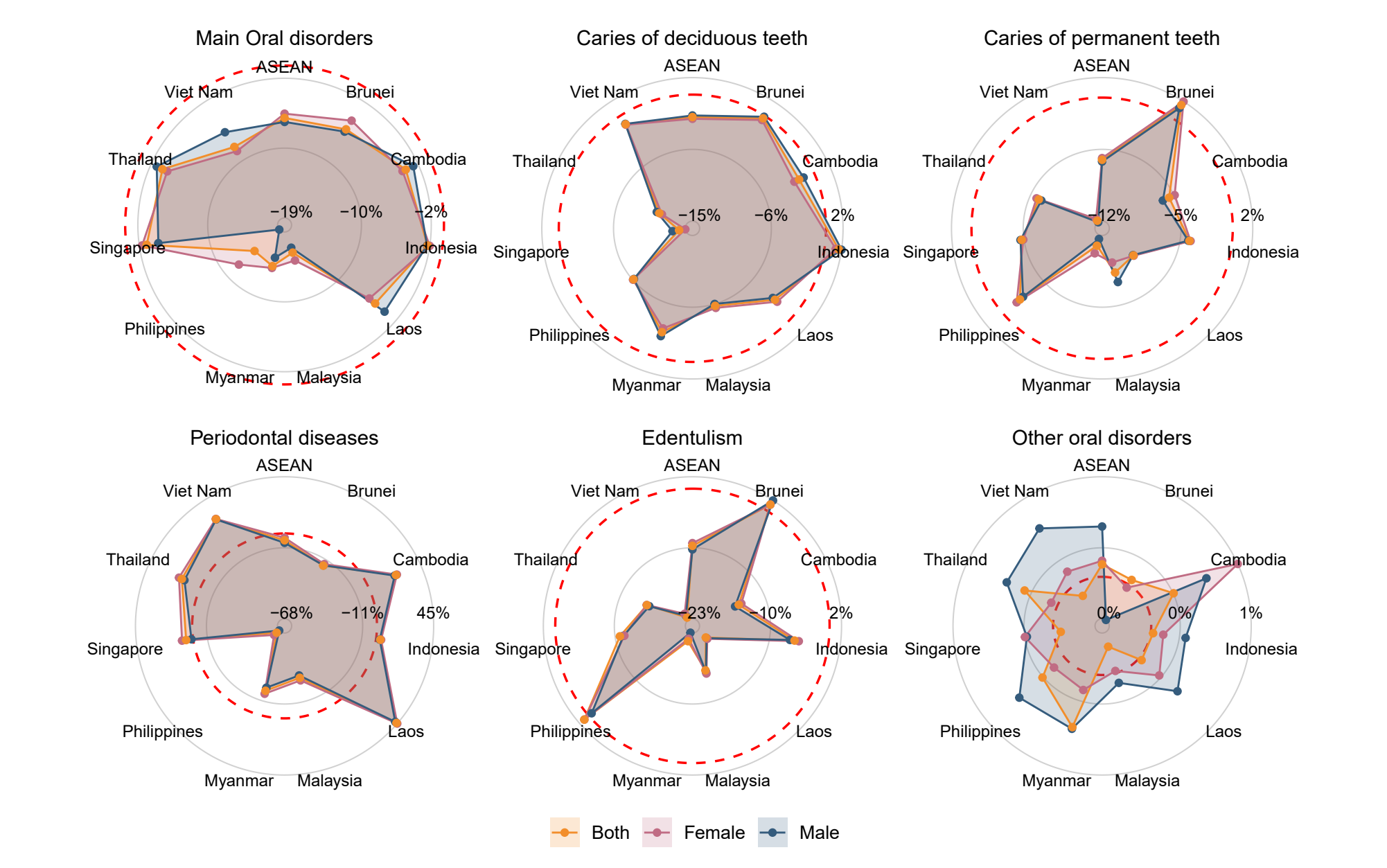


**Appendix Figure 2.** Changes in age-standardized DALY rates for oral disorders by country and sex in ASEAN, 1990–2021.


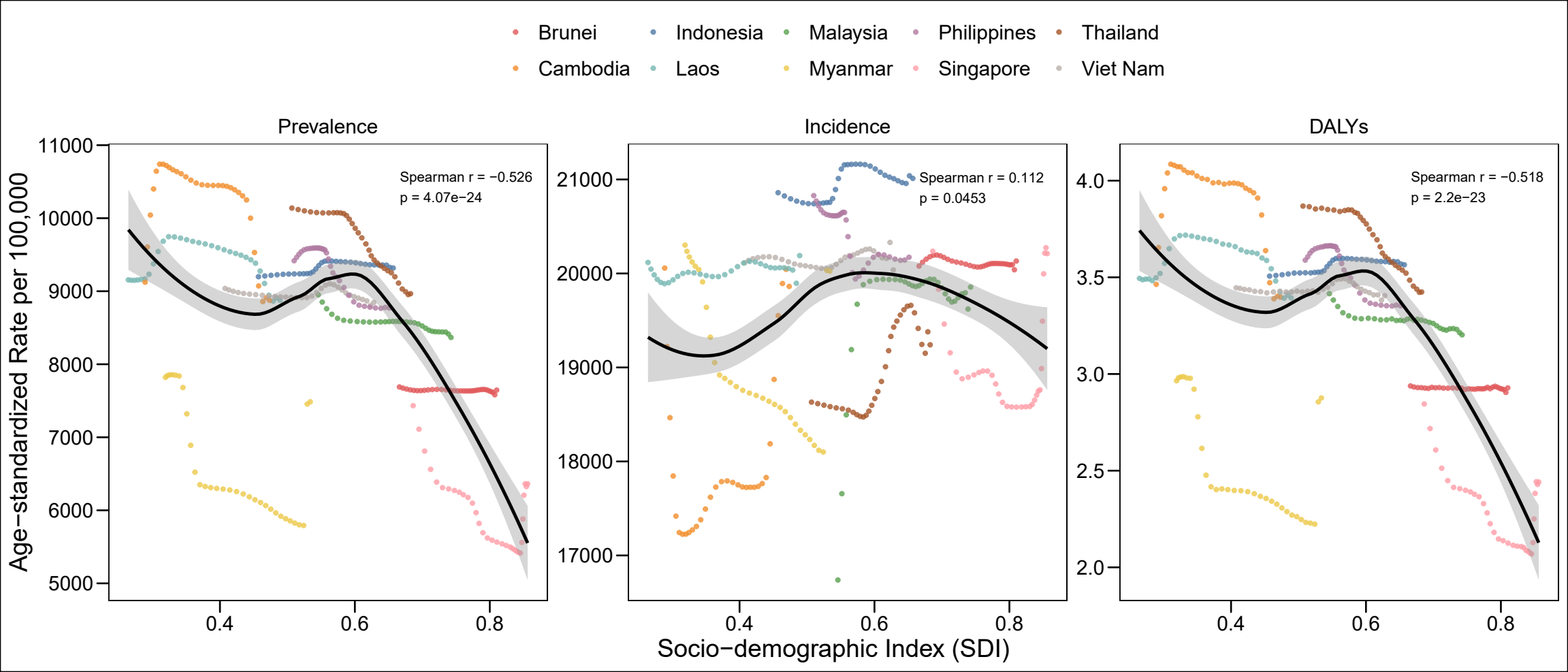


**Appendix Figure 3.** Association between age-standardized prevalence, incidence, and DALY rates of caries of deciduous teeth and Socio-demographic Index (SDI), 1990–2021.


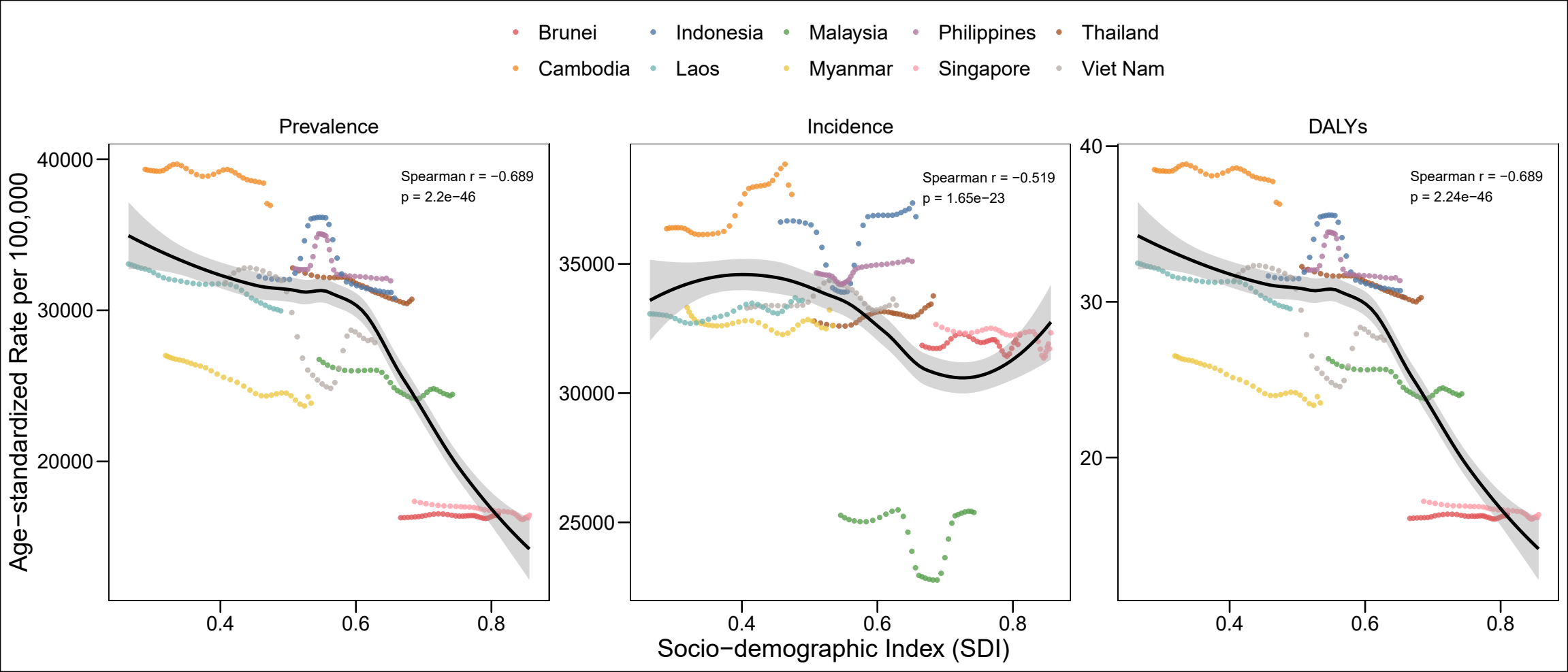


**Appendix Figure 4.** Association between age-standardized prevalence, incidence, and DALY rates of caries of permanent teeth and SDI, 1990–2021.


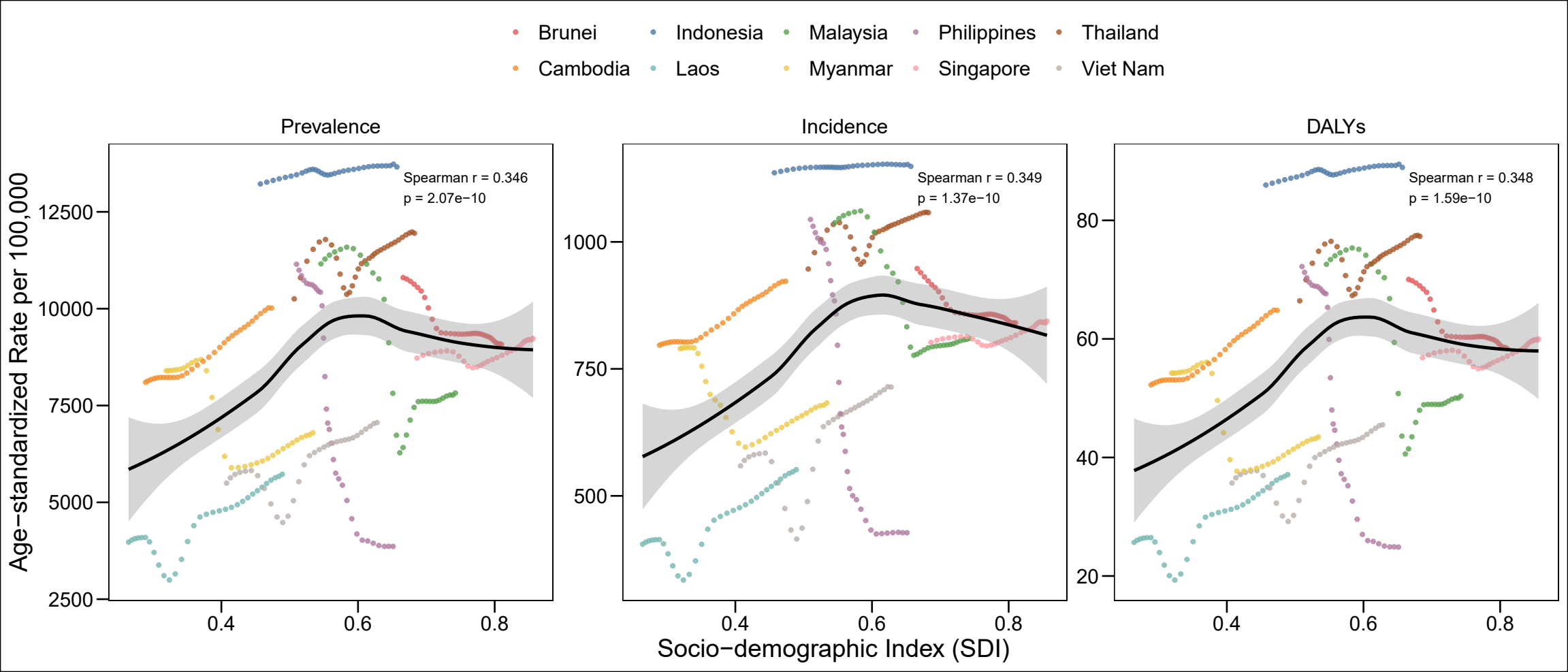


**Appendix Figure 5.** Association between age-standardized prevalence, incidence, and DALY rates of periodontal disease and SDI, 1990–2021.


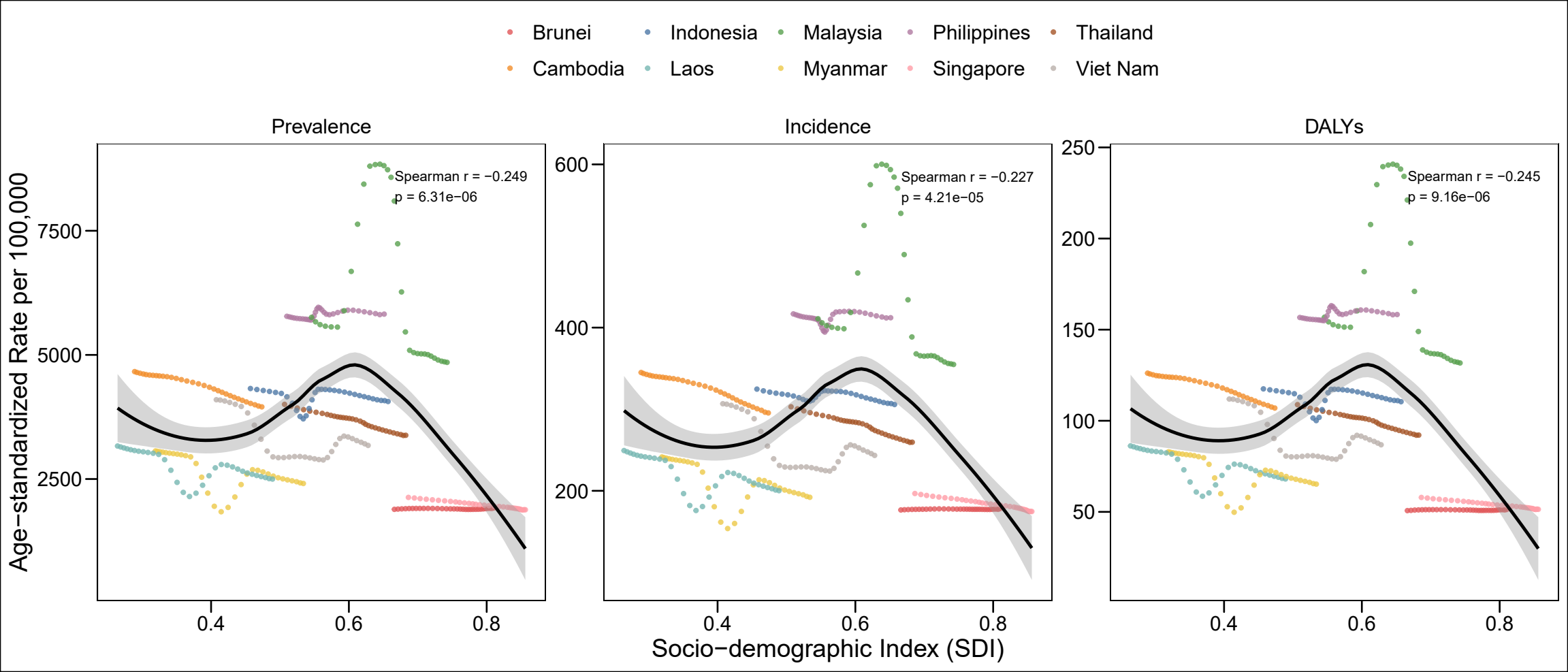


**Appendix Figure 6.** Association between age-standardized prevalence, incidence, and DALY rates of edentulism and SDI, 1990–2021.


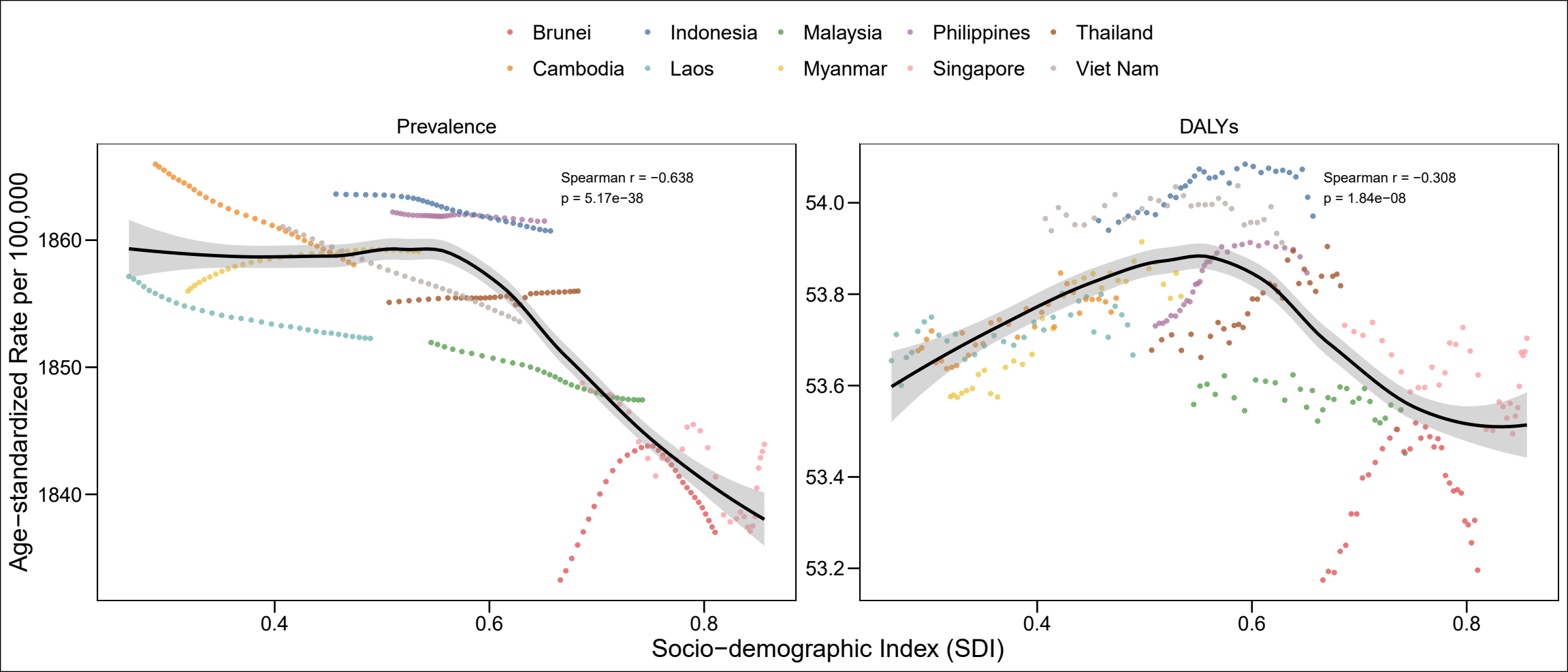


**Appendix Figure 7.** Association between age-standardized prevalence, incidence, and DALY rates of other oral disorders and SDI, 1990–2021.

**Appendix Table 1.** Definition and classification of oral disorders analyzed in this study, including case definitions, ICD-10 codes, and mapping to the GBD 2021 cause hierarchy.

| **Cause Name** | **ICD10** | **ICD10 Used in Hospital/Claims Analyses** | **ICD9** | **ICD9 Used in Hospital/Claims Analyses** | **Abbreviated Definition** |
| --- | --- | --- | --- | --- | --- |
| Oral disorders | K00-K08.499, K08.8-K14.9, M26-M27.9 | K00-K08.499, K08.8-K14.9, M26-M27.9 | 520-525.54, 525.8-526.61, 526.69-529.9, V07.31, V45.84, V49.82, V58.5, V72.2 | 520-525.54, 525.8-526.61, 526.69-529.9 | Oral disorders comprises caries of deciduous and permanent teeth, chronic periodontal diseases, edentulism (total tooth loss), and other oral disorders (a heterogeneous group including a variety of tooth, tongue, and jaw disorders and malformations not included in the other causes). |
| Caries of deciduous teeth | K00-K08.499, K08.8-K14.9, M26-M27.9 | K00-K08.499, K08.8-K14.9, M26-M27.9 | 520-525.54, 525.8-526.61, 526.69-529.9, V07.31, V45.84, V49.82, V58.5, V72.2 | 520-525.54, 525.8-526.61, 526.69-529.9 | Caries of deciduous teeth is defined as primary dentition showing unmistakable cavity, undermined enamel, a detectably softened floor or wall, a tooth with a temporary filling or a tooth that is filled but also decay is present, or teeth extracted due to caries. |
| Caries of permanent teeth | K02-K02.9 | K02-K02.9 | 521.0-521.09 | 521.0-521.09 | Caries of permanent teeth is defined as permanent dentition showing unmistakable cavity, undermined enamel, a detectably softened floor or wall, a tooth with a temporary filling or a tooth that is filled but also decay is present, or teeth extracted due to caries. |
| Periodontal diseases | K05-K06.9 | K05.3-K05.32 | 523-523.9 | 523.23, 523.25, 523.4 | Chronic periodontal disease is defined as Community Index of Periodontal Treatment Needs (CPITN) Class IV, attachment loss (AL) >6 mm, or gingival pocket depth (PD) >5 mm. It is caused by chronic bacterial infection around the teeth. |
| Edentulism | K08.0-K08.499 | K08.0-K08.199 | 525.0-525.19, 525.4-525.54 | 525.4-525.44 | Edentulism is defined as total tooth loss. |
| Other oral disorders | K00-K01.1, K03-K04.99, K07-K08, K08.8-K14.9, M26-M27.9 | K00-K01.1, K03-K05.22, K05.4-K08, K08.2-K08.499, K08.8-K14.9, M26-M27.9 | 520-521, 521.1-522.9, 524-525, 525.2-525.3, 525.8-526.61, 526.69-529.9 | 520-521, 521.1-523.22, 523.24, 523.3-523.33, 523.40-525.3, 525.5-525.54, 525.8-526.61, 526.69-529.9 | Other oral disorders encompasses a wide variety of dental, tongue, and jaw disorders and malformations, including all oral disorders that are not included in the case definitions of permanent or deciduous dental caries, periodontal disease, or edentulism and severe tooth loss, but excluding oral cancers and congenital malformations. |

**Appendix Table 2.** Percent contribution of oral disorders to all-cause prevalence, incidence, and disability-adjusted life years (DALYs) in 2021.

| location | measure | cause | percent |
| --- | --- | --- | --- |
| Global | DALYs | Main Oral disorders | 0.80% (0.51% to 1.17%) |
| Global | Prevalence | Main Oral disorders | 47.70% (43.96% to 51.62%) |
| Global | Incidence | Main Oral disorders | 10.37% (9.23% to 11.69%) |
| Global | DALYs | Caries of deciduous teeth | 0.01% (0.00% to 0.01%) |
| Global | Prevalence | Caries of deciduous teeth | 7.84% (6.55% to 9.09%) |
| Global | Incidence | Caries of deciduous teeth | 3.77% (2.96% to 4.87%) |
| Global | DALYs | Caries of permanent teeth | 0.08% (0.04% to 0.14%) |
| Global | Prevalence | Caries of permanent teeth | 28.62% (24.92% to 33.31%) |
| Global | Incidence | Caries of permanent teeth | 6.31% (5.61% to 7.11%) |
| Global | DALYs | Periodontal diseases | 0.24% (0.10% to 0.47%) |
| Global | Prevalence | Periodontal diseases | 12.99% (10.94% to 15.05%) |
| Global | Incidence | Periodontal diseases | 0.23% (0.20% to 0.26%) |
| Global | DALYs | Edentulism | 0.33% (0.23% to 0.43%) |
| Global | Prevalence | Edentulism | 4.27% (3.65% to 5.01%) |
| Global | Incidence | Edentulism | 0.06% (0.05% to 0.08%) |
| Global | DALYs | Other oral disorders | 0.15% (0.10% to 0.21%) |
| Global | Prevalence | Other oral disorders | 1.93% (1.85% to 2.01%) |
| ASEAN | DALYs | Main Oral disorders | 0.77% (0.48% to 1.12%) |
| ASEAN | Prevalence | Main Oral disorders | 48.79% (44.53% to 53.19%) |
| ASEAN | Incidence | Main Oral disorders | 11.34% (9.92% to 13.09%) |
| ASEAN | DALYs | Caries of deciduous teeth | 0.01% (0.00% to 0.02%) |
| ASEAN | Prevalence | Caries of deciduous teeth | 9.22% (7.67% to 10.91%) |
| ASEAN | Incidence | Caries of deciduous teeth | 4.10% (3.03% to 5.79%) |
| ASEAN | DALYs | Caries of permanent teeth | 0.09% (0.04% to 0.16%) |
| ASEAN | Prevalence | Caries of permanent teeth | 30.75% (26.35% to 35.78%) |
| ASEAN | Incidence | Caries of permanent teeth | 7.00% (6.18% to 7.89%) |
| ASEAN | DALYs | Periodontal diseases | 0.21% (0.09% to 0.42%) |
| ASEAN | Prevalence | Periodontal diseases | 10.53% (8.76% to 12.34%) |
| ASEAN | Incidence | Periodontal diseases | 0.18% (0.15% to 0.21%) |
| ASEAN | DALYs | Edentulism | 0.29% (0.20% to 0.39%) |
| ASEAN | Prevalence | Edentulism | 4.04% (3.48% to 4.74%) |
| ASEAN | Incidence | Edentulism | 0.06% (0.05% to 0.07%) |
| ASEAN | DALYs | Other oral disorders | 0.17% (0.11% to 0.24%) |
| ASEAN | Prevalence | Other oral disorders | 1.93% (1.85% to 2.01%) |
| Brunei | DALYs | Main Oral disorders | 0.77% (0.46% to 1.22%) |
| Brunei | Prevalence | Main Oral disorders | 37.04% (33.19% to 41.29%) |
| Brunei | Incidence | Main Oral disorders | 12.45% (10.61% to 14.41%) |
| Brunei | DALYs | Caries of deciduous teeth | 0.01% (0.00% to 0.02%) |
| Brunei | Prevalence | Caries of deciduous teeth | 8.33% (6.53% to 10.20%) |
| Brunei | Incidence | Caries of deciduous teeth | 4.69% (3.43% to 6.27%) |
| Brunei | DALYs | Caries of permanent teeth | 0.08% (0.04% to 0.15%) |
| Brunei | Prevalence | Caries of permanent teeth | 18.00% (14.76% to 21.81%) |
| Brunei | Incidence | Caries of permanent teeth | 7.52% (6.24% to 8.82%) |
| Brunei | DALYs | Periodontal diseases | 0.27% (0.11% to 0.55%) |
| Brunei | Prevalence | Periodontal diseases | 9.89% (7.54% to 12.64%) |
| Brunei | Incidence | Periodontal diseases | 0.20% (0.15% to 0.24%) |
| Brunei | DALYs | Edentulism | 0.16% (0.11% to 0.21%) |
| Brunei | Prevalence | Edentulism | 2.09% (1.62% to 2.60%) |
| Brunei | Incidence | Edentulism | 0.04% (0.03% to 0.05%) |
| Brunei | DALYs | Other oral disorders | 0.25% (0.17% to 0.35%) |
| Brunei | Prevalence | Other oral disorders | 2.00% (1.91% to 2.09%) |
| Cambodia | DALYs | Main Oral disorders | 0.65% (0.38% to 0.99%) |
| Cambodia | Prevalence | Main Oral disorders | 53.99% (48.70% to 58.96%) |
| Cambodia | Incidence | Main Oral disorders | 13.71% (12.01% to 15.81%) |
| Cambodia | DALYs | Caries of deciduous teeth | 0.01% (0.00% to 0.02%) |
| Cambodia | Prevalence | Caries of deciduous teeth | 9.11% (7.27% to 10.93%) |
| Cambodia | Incidence | Caries of deciduous teeth | 4.63% (3.40% to 6.71%) |
| Cambodia | DALYs | Caries of permanent teeth | 0.10% (0.05% to 0.19%) |
| Cambodia | Prevalence | Caries of permanent teeth | 37.93% (32.17% to 43.54%) |
| Cambodia | Incidence | Caries of permanent teeth | 8.79% (7.62% to 9.93%) |
| Cambodia | DALYs | Periodontal diseases | 0.16% (0.06% to 0.34%) |
| Cambodia | Prevalence | Periodontal diseases | 10.28% (7.45% to 13.27%) |
| Cambodia | Incidence | Periodontal diseases | 0.22% (0.16% to 0.26%) |
| Cambodia | DALYs | Edentulism | 0.22% (0.14% to 0.31%) |
| Cambodia | Prevalence | Edentulism | 4.06% (3.17% to 4.97%) |
| Cambodia | Incidence | Edentulism | 0.07% (0.05% to 0.08%) |
| Cambodia | DALYs | Other oral disorders | 0.15% (0.10% to 0.22%) |
| Cambodia | Prevalence | Other oral disorders | 1.91% (1.83% to 1.98%) |
| Indonesia | DALYs | Main Oral disorders | 0.83% (0.50% to 1.26%) |
| Indonesia | Prevalence | Main Oral disorders | 52.27% (47.68% to 56.70%) |
| Indonesia | Incidence | Main Oral disorders | 11.02% (9.58% to 12.70%) |
| Indonesia | DALYs | Caries of deciduous teeth | 0.01% (0.00% to 0.02%) |
| Indonesia | Prevalence | Caries of deciduous teeth | 9.68% (7.99% to 11.45%) |
| Indonesia | Incidence | Caries of deciduous teeth | 3.90% (2.83% to 5.57%) |
| Indonesia | DALYs | Caries of permanent teeth | 0.09% (0.04% to 0.17%) |
| Indonesia | Prevalence | Caries of permanent teeth | 32.02% (27.51% to 36.98%) |
| Indonesia | Incidence | Caries of permanent teeth | 6.84% (5.99% to 7.73%) |
| Indonesia | DALYs | Periodontal diseases | 0.28% (0.12% to 0.58%) |
| Indonesia | Prevalence | Periodontal diseases | 14.19% (10.88% to 17.47%) |
| Indonesia | Incidence | Periodontal diseases | 0.21% (0.17% to 0.25%) |
| Indonesia | DALYs | Edentulism | 0.28% (0.18% to 0.38%) |
| Indonesia | Prevalence | Edentulism | 4.22% (3.60% to 5.02%) |
| Indonesia | Incidence | Edentulism | 0.06% (0.05% to 0.07%) |
| Indonesia | DALYs | Other oral disorders | 0.17% (0.10% to 0.24%) |
| Indonesia | Prevalence | Other oral disorders | 1.93% (1.85% to 2.02%) |
| Laos | DALYs | Main Oral disorders | 0.45% (0.26% to 0.69%) |
| Laos | Prevalence | Main Oral disorders | 45.61% (40.22% to 51.11%) |
| Laos | Incidence | Main Oral disorders | 11.59% (9.85% to 13.56%) |
| Laos | DALYs | Caries of deciduous teeth | 0.01% (0.00% to 0.02%) |
| Laos | Prevalence | Caries of deciduous teeth | 9.17% (7.40% to 10.99%) |
| Laos | Incidence | Caries of deciduous teeth | 4.29% (3.08% to 6.17%) |
| Laos | DALYs | Caries of permanent teeth | 0.08% (0.03% to 0.16%) |
| Laos | Prevalence | Caries of permanent teeth | 30.94% (25.24% to 36.95%) |
| Laos | Incidence | Caries of permanent teeth | 7.14% (6.13% to 8.26%) |
| Laos | DALYs | Periodontal diseases | 0.08% (0.03% to 0.19%) |
| Laos | Prevalence | Periodontal diseases | 5.91% (4.13% to 8.12%) |
| Laos | Incidence | Periodontal diseases | 0.12% (0.08% to 0.16%) |
| Laos | DALYs | Edentulism | 0.12% (0.08% to 0.17%) |
| Laos | Prevalence | Edentulism | 2.58% (2.02% to 3.21%) |
| Laos | Incidence | Edentulism | 0.04% (0.03% to 0.05%) |
| Laos | DALYs | Other oral disorders | 0.15% (0.09% to 0.22%) |
| Laos | Prevalence | Other oral disorders | 1.91% (1.83% to 1.99%) |
| Malaysia | DALYs | Main Oral disorders | 0.87% (0.56% to 1.25%) |
| Malaysia | Prevalence | Main Oral disorders | 44.08% (39.04% to 49.55%) |
| Malaysia | Incidence | Main Oral disorders | 9.47% (7.97% to 11.30%) |
| Malaysia | DALYs | Caries of deciduous teeth | 0.01% (0.00% to 0.02%) |
| Malaysia | Prevalence | Caries of deciduous teeth | 8.83% (7.02% to 10.58%) |
| Malaysia | Incidence | Caries of deciduous teeth | 4.05% (2.88% to 5.62%) |
| Malaysia | DALYs | Caries of permanent teeth | 0.09% (0.04% to 0.16%) |
| Malaysia | Prevalence | Caries of permanent teeth | 25.78% (21.07% to 31.72%) |
| Malaysia | Incidence | Caries of permanent teeth | 5.18% (4.25% to 6.18%) |
| Malaysia | DALYs | Periodontal diseases | 0.17% (0.07% to 0.37%) |
| Malaysia | Prevalence | Periodontal diseases | 8.26% (6.22% to 10.74%) |
| Malaysia | Incidence | Periodontal diseases | 0.17% (0.13% to 0.20%) |
| Malaysia | DALYs | Edentulism | 0.41% (0.27% to 0.55%) |
| Malaysia | Prevalence | Edentulism | 5.12% (4.06% to 6.22%) |
| Malaysia | Incidence | Edentulism | 0.07% (0.06% to 0.09%) |
| Malaysia | DALYs | Other oral disorders | 0.19% (0.12% to 0.27%) |
| Malaysia | Prevalence | Other oral disorders | 1.95% (1.86% to 2.03%) |
| Myanmar | DALYs | Main Oral disorders | 0.45% (0.27% to 0.69%) |
| Myanmar | Prevalence | Main Oral disorders | 39.41% (34.47% to 44.13%) |
| Myanmar | Incidence | Main Oral disorders | 12.97% (11.07% to 14.87%) |
| Myanmar | DALYs | Caries of deciduous teeth | 0.01% (0.00% to 0.01%) |
| Myanmar | Prevalence | Caries of deciduous teeth | 7.69% (6.03% to 9.77%) |
| Myanmar | Incidence | Caries of deciduous teeth | 4.85% (3.50% to 6.46%) |
| Myanmar | DALYs | Caries of permanent teeth | 0.06% (0.03% to 0.11%) |
| Myanmar | Prevalence | Caries of permanent teeth | 24.52% (19.82% to 29.68%) |
| Myanmar | Incidence | Caries of permanent teeth | 7.91% (6.81% to 9.06%) |
| Myanmar | DALYs | Periodontal diseases | 0.10% (0.04% to 0.22%) |
| Myanmar | Prevalence | Periodontal diseases | 6.99% (5.14% to 9.14%) |
| Myanmar | Incidence | Periodontal diseases | 0.17% (0.13% to 0.21%) |
| Myanmar | DALYs | Edentulism | 0.14% (0.09% to 0.20%) |
| Myanmar | Prevalence | Edentulism | 2.48% (1.95% to 3.07%) |
| Myanmar | Incidence | Edentulism | 0.05% (0.04% to 0.06%) |
| Myanmar | DALYs | Other oral disorders | 0.14% (0.09% to 0.20%) |
| Myanmar | Prevalence | Other oral disorders | 1.91% (1.83% to 1.99%) |
| Philippines | DALYs | Main Oral disorders | 0.65% (0.42% to 0.90%) |
| Philippines | Prevalence | Main Oral disorders | 48.10% (44.46% to 51.90%) |
| Philippines | Incidence | Main Oral disorders | 11.11% (9.85% to 12.55%) |
| Philippines | DALYs | Caries of deciduous teeth | 0.01% (0.00% to 0.02%) |
| Philippines | Prevalence | Caries of deciduous teeth | 9.11% (7.71% to 10.60%) |
| Philippines | Incidence | Caries of deciduous teeth | 3.99% (3.04% to 5.38%) |
| Philippines | DALYs | Caries of permanent teeth | 0.09% (0.04% to 0.16%) |
| Philippines | Prevalence | Caries of permanent teeth | 33.18% (29.44% to 37.38%) |
| Philippines | Incidence | Caries of permanent teeth | 6.95% (6.22% to 7.70%) |
| Philippines | DALYs | Periodontal diseases | 0.06% (0.03% to 0.13%) |
| Philippines | Prevalence | Periodontal diseases | 4.01% (3.25% to 4.91%) |
| Philippines | Incidence | Periodontal diseases | 0.08% (0.07% to 0.10%) |
| Philippines | DALYs | Edentulism | 0.33% (0.22% to 0.44%) |
| Philippines | Prevalence | Edentulism | 6.04% (5.21% to 7.06%) |
| Philippines | Incidence | Edentulism | 0.08% (0.07% to 0.09%) |
| Philippines | DALYs | Other oral disorders | 0.15% (0.10% to 0.22%) |
| Philippines | Prevalence | Other oral disorders | 1.93% (1.85% to 2.02%) |
| Singapore | DALYs | Main Oral disorders | 1.27% (0.81% to 1.95%) |
| Singapore | Prevalence | Main Oral disorders | 36.40% (32.59% to 40.37%) |
| Singapore | Incidence | Main Oral disorders | 12.44% (10.69% to 14.33%) |
| Singapore | DALYs | Caries of deciduous teeth | 0.01% (0.00% to 0.01%) |
| Singapore | Prevalence | Caries of deciduous teeth | 7.06% (5.42% to 8.77%) |
| Singapore | Incidence | Caries of deciduous teeth | 4.69% (3.45% to 6.09%) |
| Singapore | DALYs | Caries of permanent teeth | 0.09% (0.04% to 0.16%) |
| Singapore | Prevalence | Caries of permanent teeth | 18.26% (14.93% to 22.03%) |
| Singapore | Incidence | Caries of permanent teeth | 7.51% (6.35% to 8.77%) |
| Singapore | DALYs | Periodontal diseases | 0.47% (0.19% to 0.96%) |
| Singapore | Prevalence | Periodontal diseases | 10.25% (7.79% to 13.08%) |
| Singapore | Incidence | Periodontal diseases | 0.20% (0.15% to 0.24%) |
| Singapore | DALYs | Edentulism | 0.39% (0.28% to 0.54%) |
| Singapore | Prevalence | Edentulism | 2.09% (1.64% to 2.59%) |
| Singapore | Incidence | Edentulism | 0.04% (0.03% to 0.05%) |
| Singapore | DALYs | Other oral disorders | 0.32% (0.21% to 0.42%) |
| Singapore | Prevalence | Other oral disorders | 2.05% (1.95% to 2.14%) |
| Thailand | DALYs | Main Oral disorders | 1.01% (0.61% to 1.53%) |
| Thailand | Prevalence | Main Oral disorders | 51.14% (46.10% to 56.70%) |
| Thailand | Incidence | Main Oral disorders | 10.11% (8.69% to 11.83%) |
| Thailand | DALYs | Caries of deciduous teeth | 0.00% (0.00% to 0.01%) |
| Thailand | Prevalence | Caries of deciduous teeth | 9.41% (7.90% to 10.84%) |
| Thailand | Incidence | Caries of deciduous teeth | 3.58% (2.53% to 5.13%) |
| Thailand | DALYs | Caries of permanent teeth | 0.10% (0.04% to 0.18%) |
| Thailand | Prevalence | Caries of permanent teeth | 32.24% (26.80% to 38.40%) |
| Thailand | Incidence | Caries of permanent teeth | 6.28% (5.42% to 7.28%) |
| Thailand | DALYs | Periodontal diseases | 0.32% (0.13% to 0.68%) |
| Thailand | Prevalence | Periodontal diseases | 12.53% (9.92% to 15.82%) |
| Thailand | Incidence | Periodontal diseases | 0.20% (0.16% to 0.24%) |
| Thailand | DALYs | Edentulism | 0.42% (0.28% to 0.59%) |
| Thailand | Prevalence | Edentulism | 3.55% (2.78% to 4.35%) |
| Thailand | Incidence | Edentulism | 0.05% (0.04% to 0.06%) |
| Thailand | DALYs | Other oral disorders | 0.17% (0.11% to 0.24%) |
| Thailand | Prevalence | Other oral disorders | 1.95% (1.87% to 2.02%) |
| Viet Nam | DALYs | Main Oral disorders | 0.79% (0.48% to 1.21%) |
| Viet Nam | Prevalence | Main Oral disorders | 45.37% (40.22% to 50.34%) |
| Viet Nam | Incidence | Main Oral disorders | 12.99% (11.07% to 15.42%) |
| Viet Nam | DALYs | Caries of deciduous teeth | 0.01% (0.00% to 0.02%) |
| Viet Nam | Prevalence | Caries of deciduous teeth | 9.23% (7.46% to 11.09%) |
| Viet Nam | Incidence | Caries of deciduous teeth | 4.78% (3.45% to 6.93%) |
| Viet Nam | DALYs | Caries of permanent teeth | 0.10% (0.05% to 0.19%) |
| Viet Nam | Prevalence | Caries of permanent teeth | 29.07% (23.59% to 34.73%) |
| Viet Nam | Incidence | Caries of permanent teeth | 7.98% (6.81% to 9.25%) |
| Viet Nam | DALYs | Periodontal diseases | 0.18% (0.07% to 0.37%) |
| Viet Nam | Prevalence | Periodontal diseases | 7.37% (5.37% to 9.56%) |
| Viet Nam | Incidence | Periodontal diseases | 0.17% (0.13% to 0.21%) |
| Viet Nam | DALYs | Edentulism | 0.30% (0.20% to 0.42%) |
| Viet Nam | Prevalence | Edentulism | 3.31% (2.57% to 4.11%) |
| Viet Nam | Incidence | Edentulism | 0.06% (0.05% to 0.07%) |
| Viet Nam | DALYs | Other oral disorders | 0.20% (0.13% to 0.29%) |
| Viet Nam | Prevalence | Other oral disorders | 1.93% (1.85% to 2.01%) |

**Appendix Table 3.** Number and age-standardized rate of oral disorders by sex and country, 1990 and 2021.

| location | sex | cause | DALYs_number_1990 | DALYs_number_2021 | DALYs_number_change | DALYs_rate_1990 | DALYs_rate_2021 | DALYs_rate_change | Prevalence_number_1990 | Prevalence_number_2021 | Prevalence_number_change | Prevalence_rate_1990 | Prevalence_rate_2021 | Prevalence_rate_change | Incidence_number_1990 | Incidence_number_2021 | Incidence_number_change | Incidence_rate_1990 | Incidence_rate_2021 | Incidence_rate_change |
| --- | --- | --- | --- | --- | --- | --- | --- | --- | --- | --- | --- | --- | --- | --- | --- | --- | --- | --- | --- | --- |
| ASEAN | Both | Main Oral disorders | 887,262.9 (510,228.6 to 1,377,266.3) | 1,749,265.4 (1,056,472.8 to 2,636,776.5) | 97.2% (87.3% to 110.0%) | 275.5 (163.2 to 414.6) | 257.9 (157.0 to 385.7) | -6.4% (-11.1% to -0.2%) | 209,562,208.4 (188,453,387.1 to 233,442,286.4) | 319,633,616.2 (290,056,843.6 to 350,847,279.6) | 52.5% (47.3% to 58.7%) | 49,385.3 (44,775.4 to 54,107.5) | 46,931.2 (42,762.9 to 51,177.3) | -5.0% (-7.0% to -2.9%) | 278,470,380.1 (237,055,884.1 to 330,818,898.7) | 372,208,194.4 (325,156,152.4 to 429,959,319.9) | 33.7% (28.0% to 39.2%) | 56,085.3 (48,474.3 to 65,369.6) | 56,404.0 (48,936.6 to 65,459.6) | 0.6% (-1.2% to 2.5%) |
| ASEAN | Both | Caries of deciduous teeth | 19,900.5 (8,689.7 to 39,028.6) | 19,435.5 (8,345.3 to 38,666.5) | -2.3% (-7.3% to 3.5%) | 3.5 (1.5 to 6.8) | 3.4 (1.5 to 6.7) | -2.7% (-7.7% to 3.1%) | 52,225,621.3 (43,193,977.8 to 62,014,997.5) | 50,743,914.3 (42,011,579.3 to 60,495,537.1) | -2.8% (-4.8% to -1.0%) | 9,157.0 (7,571.9 to 10,852.3) | 8,868.4 (7,352.1 to 10,518.9) | -3.2% (-4.9% to -1.4%) | 116,513,012.5 (85,434,298.9 to 166,638,629.8) | 118,995,523.2 (87,782,129.6 to 172,583,995.9) | 2.1% (-0.7% to 5.4%) | 20,261.2 (14,901.6 to 28,862.0) | 20,399.7 (15,168.2 to 29,211.5) | 0.7% (-2.3% to 3.9%) |
| ASEAN | Both | Caries of permanent teeth | 130,790.0 (56,937.6 to 253,408.6) | 206,002.8 (90,221.0 to 406,234.2) | 57.5% (49.1% to 67.2%) | 31.1 (13.6 to 59.9) | 29.1 (12.7 to 56.8) | -6.3% (-8.5% to -3.9%) | 132,184,084.0 (110,937,056.7 to 156,843,100.3) | 208,997,607.4 (178,263,387.6 to 244,484,421.5) | 58.1% (49.7% to 67.7%) | 31,616.7 (27,031.5 to 36,853.6) | 29,577.9 (25,319.9 to 34,442.3) | -6.4% (-8.6% to -4.2%) | 157,733,460.9 (136,378,444.5 to 178,666,209.4) | 244,735,435.1 (216,231,426.5 to 275,364,802.9) | 55.2% (49.1% to 61.7%) | 34,535.9 (30,064.7 to 39,022.1) | 34,808.5 (30,638.3 to 39,161.3) | 0.8% (-1.9% to 3.3%) |
| ASEAN | Both | Periodontal diseases | 224,137.7 (88,960.8 to 488,528.9) | 476,089.7 (191,699.1 to 953,520.5) | 112.4% (83.8% to 152.0%) | 69.4 (27.3 to 150.2) | 65.6 (26.3 to 132.0) | -5.5% (-17.3% to 11.7%) | 34,331,872.9 (25,268,271.1 to 43,269,177.2) | 73,210,343.2 (60,602,261.1 to 86,448,108.4) | 113.2% (84.4% to 152.8%) | 10,690.4 (8,082.7 to 13,325.8) | 10,122.7 (8,449.1 to 11,880.1) | -5.3% (-17.3% to 11.6%) | 3,357,057.3 (2,535,859.2 to 4,034,188.6) | 6,498,745.5 (5,581,604.9 to 7,401,163.0) | 93.6% (71.3% to 127.1%) | 964.6 (754.2 to 1,134.8) | 902.4 (782.5 to 1,022.2) | -6.4% (-15.4% to 7.0%) |
| ASEAN | Both | Edentulism | 285,784.5 (183,372.8 to 397,314.0) | 668,425.7 (430,471.3 to 944,986.0) | 133.9% (112.2% to 165.6%) | 117.7 (74.9 to 166.1) | 105.9 (68.8 to 149.6) | -10.1% (-18.5% to 2.4%) | 10,386,847.2 (8,234,657.7 to 12,587,731.6) | 24,336,810.7 (20,812,612.2 to 28,601,232.0) | 134.3% (112.7% to 165.8%) | 4,330.2 (3,434.2 to 5,261.3) | 3,888.3 (3,346.8 to 4,557.2) | -10.2% (-18.3% to 2.4%) | 866,849.4 (696,539.3 to 1,057,779.8) | 1,978,490.6 (1,680,769.1 to 2,339,593.1) | 128.2% (107.4% to 163.0%) | 323.6 (261.9 to 392.5) | 293.4 (251.3 to 343.0) | -9.3% (-17.6% to 3.5%) |
| ASEAN | Both | Other oral disorders | 226,650.2 (138,628.3 to 339,933.1) | 379,311.6 (232,774.3 to 568,853.0) | 67.4% (64.7% to 70.1%) | 53.8 (33.1 to 80.7) | 53.9 (33.1 to 80.8) | 0.1% (-0.7% to 0.8%) | 7,779,565.2 (7,452,877.6 to 8,132,739.3) | 13,074,271.9 (12,514,688.4 to 13,626,646.8) | 68.1% (65.8% to 70.5%) | 1,860.7 (1,784.4 to 1,936.1) | 1,858.2 (1,781.1 to 1,933.8) | -0.1% (-0.3% to 0.0%) | NA | NA | NA | NA | NA | NA |
| ASEAN | Female | Main Oral disorders | 502,801.0 (294,518.4 to 767,892.6) | 1,000,943.5 (619,191.4 to 1,489,138.0) | 99.1% (88.7% to 112.6%) | 302.5 (181.0 to 454.2) | 284.8 (176.6 to 422.0) | -5.9% (-10.9% to 0.4%) | 106,863,441.9 (96,417,987.0 to 118,641,918.2) | 162,845,571.7 (148,228,773.0 to 178,053,717.0) | 52.4% (47.1% to 58.3%) | 49,884.6 (45,441.6 to 54,551.7) | 47,598.4 (43,443.1 to 51,784.8) | -4.6% (-6.7% to -2.5%) | 139,093,102.9 (118,974,100.3 to 164,604,486.8) | 183,859,733.6 (160,518,920.9 to 211,454,426.8) | 32.2% (26.7% to 38.0%) | 56,148.7 (48,667.7 to 65,505.4) | 56,449.3 (48,905.5 to 65,477.5) | 0.5% (-1.6% to 2.7%) |
| ASEAN | Female | Caries of deciduous teeth | 9,732.6 (4,224.6 to 19,214.7) | 9,423.9 (4,034.8 to 18,811.8) | -3.2% (-8.3% to 3.2%) | 3.5 (1.5 to 6.9) | 3.4 (1.5 to 6.8) | -2.9% (-8.1% to 3.4%) | 25,531,175.2 (21,127,447.1 to 30,334,989.4) | 24,613,898.7 (20,430,647.9 to 29,302,271.5) | -3.6% (-5.6% to -1.1%) | 9,164.6 (7,582.2 to 10,879.6) | 8,864.2 (7,379.0 to 10,511.0) | -3.3% (-5.2% to -0.9%) | 56,962,861.0 (41,676,157.4 to 81,189,725.9) | 57,771,893.8 (42,513,899.6 to 83,663,373.0) | 1.4% (-2.1% to 4.9%) | 20,265.1 (14,865.0 to 28,762.4) | 20,406.5 (15,135.1 to 29,177.1) | 0.7% (-2.8% to 4.0%) |
| ASEAN | Female | Caries of permanent teeth | 66,478.2 (28,864.2 to 128,513.7) | 103,229.4 (45,407.5 to 203,469.1) | 55.3% (46.7% to 65.1%) | 31.0 (13.6 to 59.7) | 29.1 (12.8 to 56.8) | -6.1% (-8.7% to -3.2%) | 67,371,047.5 (56,873,389.7 to 79,712,782.5) | 105,209,496.2 (90,001,984.4 to 122,458,947.1) | 56.2% (47.6% to 65.9%) | 31,647.5 (27,049.8 to 36,811.7) | 29,692.6 (25,441.0 to 34,498.5) | -6.2% (-8.7% to -3.2%) | 79,832,488.7 (69,185,693.2 to 90,297,757.9) | 121,468,897.8 (106,812,382.2 to 137,051,585.6) | 52.2% (46.4% to 59.7%) | 34,537.9 (30,001.7 to 39,057.4) | 34,774.6 (30,543.8 to 39,198.9) | 0.7% (-2.0% to 3.6%) |
| ASEAN | Female | Periodontal diseases | 116,368.7 (46,101.5 to 250,928.7) | 248,458.6 (99,969.4 to 493,935.3) | 113.5% (85.0% to 152.3%) | 69.2 (27.3 to 149.8) | 66.9 (26.9 to 133.5) | -3.4% (-15.4% to 13.2%) | 17,874,558.4 (13,233,698.7 to 22,556,137.4) | 38,358,572.4 (31,599,832.1 to 45,236,576.9) | 114.6% (86.1% to 153.5%) | 10,682.4 (8,110.2 to 13,308.4) | 10,345.4 (8,605.9 to 12,182.1) | -3.2% (-15.3% to 13.4%) | 1,759,929.6 (1,336,608.8 to 2,110,635.2) | 3,402,095.3 (2,938,880.2 to 3,851,326.1) | 93.3% (71.1% to 126.6%) | 974.4 (766.7 to 1,146.1) | 928.7 (808.8 to 1,047.5) | -4.7% (-14.1% to 8.5%) |
| ASEAN | Female | Edentulism | 186,885.1 (119,884.1 to 260,720.6) | 436,267.7 (279,169.9 to 613,747.5) | 133.4% (111.3% to 164.6%) | 141.1 (89.4 to 198.1) | 127.6 (82.4 to 180.4) | -9.6% (-17.7% to 2.5%) | 6,798,773.6 (5,416,120.9 to 8,269,358.6) | 15,921,674.3 (13,602,170.0 to 18,625,403.5) | 134.2% (113.0% to 165.4%) | 5,184.5 (4,130.3 to 6,283.3) | 4,684.5 (4,030.7 to 5,473.3) | -9.6% (-17.7% to 2.5%) | 537,823.7 (435,573.1 to 650,096.5) | 1,216,846.7 (1,043,576.9 to 1,423,573.1) | 126.3% (105.6% to 157.8%) | 371.3 (303.5 to 447.1) | 339.5 (293.4 to 394.2) | -8.6% (-16.8% to 3.7%) |
| ASEAN | Female | Other oral disorders | 123,336.4 (75,772.8 to 183,349.9) | 203,563.9 (124,991.9 to 302,784.5) | 65.0% (61.8% to 68.2%) | 57.7 (35.4 to 85.8) | 57.8 (35.4 to 85.9) | 0.1% (-0.9% to 1.2%) | 4,245,438.7 (4,052,687.3 to 4,432,835.8) | 7,050,550.3 (6,736,866.5 to 7,354,144.5) | 66.1% (63.7% to 68.6%) | 1,999.5 (1,915.5 to 2,082.0) | 1,999.7 (1,915.8 to 2,082.7) | 0.0% (-0.2% to 0.2%) | NA | NA | NA | NA | NA | NA |
| ASEAN | Male | Main Oral disorders | 384,462.0 (217,595.6 to 609,697.2) | 748,321.9 (438,625.5 to 1,172,446.7) | 94.6% (84.7% to 107.0%) | 245.6 (143.9 to 375.5) | 228.7 (136.5 to 351.3) | -6.9% (-11.7% to -1.2%) | 102,698,766.6 (92,079,985.3 to 114,764,561.4) | 156,788,044.5 (141,544,780.5 to 172,869,166.5) | 52.7% (47.3% to 59.0%) | 48,845.3 (44,135.2 to 53,740.2) | 46,235.7 (41,969.2 to 50,657.4) | -5.3% (-7.6% to -3.1%) | 139,377,277.2 (118,690,887.7 to 165,806,739.6) | 188,348,460.7 (163,885,173.6 to 217,935,662.8) | 35.1% (28.9% to 40.9%) | 56,020.3 (48,371.6 to 65,262.8) | 56,359.1 (48,833.9 to 65,363.2) | 0.6% (-1.9% to 2.9%) |
| ASEAN | Male | Caries of deciduous teeth | 10,168.0 (4,442.1 to 19,813.9) | 10,011.6 (4,317.8 to 19,854.7) | -1.5% (-7.2% to 4.6%) | 3.5 (1.5 to 6.8) | 3.4 (1.5 to 6.7) | -2.5% (-8.1% to 3.5%) | 26,694,446.1 (22,066,388.8 to 31,609,311.6) | 26,130,015.6 (21,598,841.2 to 31,104,211.8) | -2.1% (-4.5% to 0.1%) | 9,149.8 (7,562.0 to 10,816.7) | 8,872.4 (7,340.7 to 10,502.7) | -3.0% (-5.4% to -0.9%) | 59,550,151.5 (43,837,947.7 to 85,859,303.3) | 61,223,629.3 (45,275,228.3 to 88,920,622.9) | 2.8% (-1.4% to 6.2%) | 20,257.6 (14,954.1 to 29,100.8) | 20,393.2 (15,187.4 to 29,244.0) | 0.7% (-3.5% to 3.9%) |
| ASEAN | Male | Caries of permanent teeth | 64,311.8 (27,997.0 to 124,879.8) | 102,773.4 (45,110.6 to 202,444.0) | 59.8% (51.0% to 69.9%) | 31.1 (13.7 to 60.1) | 29.1 (12.7 to 56.6) | -6.5% (-8.9% to -3.7%) | 64,813,036.5 (54,213,811.3 to 77,309,821.8) | 103,788,111.2 (87,886,413.7 to 121,659,314.9) | 60.1% (51.3% to 70.3%) | 31,588.5 (26,947.8 to 36,807.0) | 29,465.0 (25,200.1 to 34,298.2) | -6.7% (-9.0% to -4.1%) | 77,900,972.2 (67,365,374.4 to 88,482,034.7) | 123,266,537.3 (108,923,858.6 to 138,577,303.8) | 58.2% (50.8% to 65.0%) | 34,535.1 (30,042.9 to 38,945.8) | 34,843.7 (30,705.4 to 39,145.6) | 0.9% (-2.3% to 3.8%) |
| ASEAN | Male | Periodontal diseases | 107,769.0 (42,524.2 to 235,614.5) | 227,631.1 (91,623.5 to 461,223.9) | 111.2% (83.1% to 152.8%) | 69.8 (27.5 to 151.0) | 64.6 (25.8 to 130.9) | -7.5% (-19.0% to 10.0%) | 16,457,314.5 (12,058,989.4 to 20,899,302.9) | 34,851,770.8 (28,629,677.5 to 41,489,169.7) | 111.8% (83.4% to 152.7%) | 10,725.2 (8,076.9 to 13,352.4) | 9,929.2 (8,236.9 to 11,690.9) | -7.4% (-19.1% to 9.8%) | 1,597,127.7 (1,200,472.7 to 1,924,681.3) | 3,096,650.2 (2,623,303.6 to 3,526,547.4) | 93.9% (71.2% to 127.5%) | 954.6 (739.3 to 1,124.0) | 876.5 (756.6 to 994.5) | -8.2% (-17.0% to 5.5%) |
| ASEAN | Male | Edentulism | 98,899.3 (62,848.8 to 139,381.1) | 232,158.0 (150,450.3 to 328,895.0) | 134.7% (111.6% to 169.9%) | 91.4 (58.6 to 130.0) | 81.7 (53.5 to 115.0) | -10.7% (-19.4% to 3.0%) | 3,588,073.6 (2,798,601.6 to 4,408,859.4) | 8,415,136.4 (7,154,619.0 to 9,981,861.8) | 134.5% (111.6% to 169.8%) | 3,363.8 (2,651.5 to 4,153.1) | 2,994.1 (2,548.0 to 3,560.1) | -11.0% (-19.4% to 2.7%) | 329,025.8 (261,647.2 to 407,485.0) | 761,643.9 (637,803.1 to 910,039.3) | 131.5% (108.8% to 170.8%) | 273.1 (219.1 to 337.4) | 245.7 (208.1 to 291.6) | -10.0% (-18.7% to 3.8%) |
| ASEAN | Male | Other oral disorders | 103,313.8 (63,147.6 to 156,583.2) | 175,747.7 (107,621.6 to 265,033.4) | 70.1% (66.9% to 73.5%) | 49.8 (30.5 to 75.4) | 50.0 (30.7 to 75.3) | 0.4% (-0.7% to 1.7%) | 3,534,126.5 (3,381,307.3 to 3,700,175.0) | 6,023,721.6 (5,760,172.2 to 6,292,654.7) | 70.4% (68.0% to 72.9%) | 1,715.2 (1,644.5 to 1,788.3) | 1,715.5 (1,644.2 to 1,788.5) | 0.0% (-0.2% to 0.2%) | NA | NA | NA | NA | NA | NA |
| Brunei | Both | Main Oral disorders | 346.2 (189.2 to 560.0) | 778.6 (441.3 to 1,257.3) | 124.9% (92.6% to 160.2%) | 192.9 (109.4 to 306.0) | 182.2 (106.9 to 287.9) | -5.6% (-18.2% to 6.7%) | 86,203.3 (76,669.8 to 97,401.2) | 151,022.3 (133,299.6 to 169,940.3) | 75.2% (57.9% to 93.2%) | 35,161.6 (31,698.9 to 38,924.9) | 33,997.5 (30,240.3 to 38,030.8) | -3.3% (-11.0% to 5.9%) | 153,333.7 (130,173.9 to 178,627.2) | 231,173.7 (198,308.0 to 265,155.7) | 50.8% (39.7% to 61.3%) | 53,048.7 (45,349.3 to 61,674.7) | 53,401.0 (45,415.1 to 61,515.9) | 0.7% (-4.4% to 6.3%) |
| Brunei | Both | Caries of deciduous teeth | 9.5 (4.1 to 18.4) | 9.5 (3.9 to 18.5) | -0.4% (-9.5% to 8.7%) | 2.9 (1.3 to 5.6) | 2.9 (1.2 to 5.7) | -0.3% (-8.9% to 8.7%) | 24,900.8 (19,677.8 to 30,579.0) | 24,739.5 (19,336.1 to 30,523.3) | -0.6% (-6.3% to 5.7%) | 7,689.0 (6,084.9 to 9,450.4) | 7,645.7 (5,952.2 to 9,419.5) | -0.6% (-6.0% to 5.0%) | 63,404.8 (46,788.0 to 84,524.1) | 66,322.4 (48,148.7 to 90,675.3) | 4.6% (-1.0% to 11.0%) | 20,078.5 (14,693.0 to 27,217.8) | 20,132.0 (14,756.7 to 27,197.7) | 0.3% (-4.3% to 6.1%) |
| Brunei | Both | Caries of permanent teeth | 43.4 (18.8 to 83.9) | 81.6 (35.6 to 154.8) | 88.1% (71.4% to 105.5%) | 16.1 (7.0 to 31.4) | 16.4 (7.2 to 31.0) | 1.6% (-6.7% to 10.2%) | 43,615.7 (35,912.0 to 53,355.8) | 82,289.2 (66,191.8 to 101,518.8) | 88.7% (73.0% to 104.8%) | 16,275.3 (13,568.9 to 19,506.9) | 16,520.3 (13,486.0 to 20,024.7) | 1.5% (-6.7% to 10.1%) | 87,921.2 (74,080.4 to 101,916.1) | 160,127.0 (134,658.0 to 184,712.6) | 82.1% (65.8% to 98.0%) | 31,846.2 (26,789.5 to 36,826.6) | 32,250.1 (27,198.3 to 37,510.5) | 1.3% (-6.8% to 11.0%) |
| Brunei | Both | Periodontal diseases | 115.1 (45.6 to 253.1) | 274.9 (108.7 to 565.2) | 138.9% (59.2% to 271.7%) | 70.0 (27.9 to 147.6) | 58.4 (23.0 to 117.4) | -16.7% (-41.9% to 20.5%) | 17,575.6 (12,716.5 to 22,448.9) | 42,460.6 (31,483.7 to 56,484.3) | 141.6% (59.5% to 274.1%) | 10,803.5 (8,282.1 to 13,387.6) | 9,077.4 (6,920.8 to 11,561.1) | -16.0% (-41.4% to 20.7%) | 1,828.9 (1,341.2 to 2,277.6) | 4,104.7 (3,112.6 to 5,218.4) | 124.4% (57.2% to 223.0%) | 947.5 (756.7 to 1,122.8) | 840.5 (664.0 to 1,026.4) | -11.3% (-32.6% to 20.7%) |
| Brunei | Both | Edentulism | 47.0 (30.0 to 68.7) | 156.3 (99.2 to 225.2) | 232.4% (211.2% to 251.9%) | 50.7 (32.9 to 73.6) | 51.3 (33.2 to 75.0) | 1.2% (-3.2% to 5.7%) | 1,731.2 (1,362.2 to 2,118.8) | 5,749.5 (4,405.9 to 7,245.8) | 232.1% (216.1% to 249.7%) | 1,889.2 (1,488.5 to 2,359.5) | 1,917.0 (1,490.7 to 2,390.9) | 1.5% (-2.5% to 5.4%) | 178.9 (143.2 to 223.2) | 619.6 (479.8 to 768.9) | 246.4% (224.8% to 267.7%) | 176.5 (140.6 to 223.1) | 178.3 (138.9 to 223.1) | 1.0% (-2.6% to 5.4%) |
| Brunei | Both | Other oral disorders | 131.2 (79.9 to 195.1) | 256.3 (156.7 to 387.8) | 95.3% (87.4% to 103.3%) | 53.2 (32.4 to 79.1) | 53.2 (32.6 to 80.4) | 0.0% (-3.3% to 3.7%) | 4,482.3 (4,255.6 to 4,697.3) | 8,831.5 (8,400.1 to 9,245.1) | 97.0% (93.7% to 100.3%) | 1,833.3 (1,752.0 to 1,913.8) | 1,837.0 (1,755.5 to 1,917.3) | 0.2% (0.1% to 0.3%) | NA | NA | NA | NA | NA | NA |
| Brunei | Female | Main Oral disorders | 153.8 (85.2 to 247.3) | 354.8 (203.8 to 566.0) | 130.8% (101.7% to 164.7%) | 180.9 (103.6 to 287.4) | 173.0 (102.0 to 270.3) | -4.3% (-16.9% to 8.5%) | 39,103.2 (34,572.7 to 44,270.8) | 68,879.2 (60,425.7 to 77,673.1) | 76.1% (59.4% to 94.0%) | 33,584.0 (29,884.3 to 37,553.5) | 32,811.2 (29,195.0 to 36,777.9) | -2.3% (-10.7% to 7.4%) | 72,455.4 (60,311.7 to 85,741.0) | 108,836.9 (94,180.9 to 125,479.6) | 50.2% (37.6% to 65.6%) | 52,840.0 (44,214.8 to 61,728.9) | 53,462.9 (45,701.4 to 62,796.2) | 1.2% (-5.5% to 8.3%) |
| Brunei | Female | Caries of deciduous teeth | 4.5 (2.0 to 9.0) | 4.5 (1.9 to 8.7) | -0.8% (-12.8% to 15.1%) | 2.9 (1.3 to 5.7) | 2.9 (1.2 to 5.6) | -0.6% (-12.8% to 14.7%) | 11,836.5 (9,370.0 to 14,672.8) | 11,739.3 (9,283.8 to 14,487.4) | -0.8% (-8.8% to 8.8%) | 7,593.9 (6,006.6 to 9,431.8) | 7,546.5 (5,996.5 to 9,296.3) | -0.6% (-9.0% to 8.6%) | 30,690.4 (22,414.9 to 40,955.7) | 31,889.3 (22,794.9 to 43,756.4) | 3.9% (-4.9% to 12.2%) | 20,133.6 (14,551.8 to 27,220.1) | 20,147.4 (14,470.8 to 27,372.6) | 0.1% (-7.3% to 7.7%) |
| Brunei | Female | Caries of permanent teeth | 20.2 (8.8 to 38.6) | 38.2 (16.4 to 74.1) | 88.6% (66.9% to 116.0%) | 16.1 (7.1 to 31.1) | 16.4 (7.2 to 31.8) | 2.0% (-9.9% to 13.4%) | 20,377.1 (16,700.0 to 24,982.4) | 38,575.6 (31,440.9 to 47,492.8) | 89.3% (69.1% to 116.9%) | 16,276.7 (13,400.1 to 19,778.8) | 16,590.0 (13,637.1 to 20,240.8) | 1.9% (-8.6% to 12.8%) | 41,017.3 (33,895.4 to 47,940.8) | 75,014.7 (63,523.5 to 86,571.9) | 82.9% (62.7% to 105.5%) | 31,723.6 (26,611.7 to 37,082.7) | 32,421.6 (27,249.6 to 37,189.3) | 2.2% (-7.0% to 14.8%) |
| Brunei | Female | Periodontal diseases | 41.1 (16.0 to 89.2) | 108.9 (42.2 to 227.9) | 165.0% (70.9% to 322.3%) | 57.2 (22.1 to 120.1) | 48.6 (18.8 to 102.2) | -15.2% (-43.6% to 28.7%) | 6,302.6 (4,445.0 to 8,292.3) | 16,890.4 (12,173.4 to 22,613.9) | 168.0% (72.8% to 328.7%) | 8,857.1 (6,521.8 to 11,359.8) | 7,573.5 (5,623.5 to 9,849.9) | -14.5% (-43.4% to 28.6%) | 669.9 (478.4 to 851.8) | 1,650.9 (1,209.3 to 2,135.4) | 146.4% (65.2% to 267.1%) | 813.4 (626.8 to 990.8) | 723.0 (554.4 to 902.7) | -11.1% (-34.8% to 24.3%) |
| Brunei | Female | Edentulism | 19.8 (12.6 to 28.4) | 67.8 (42.7 to 99.9) | 242.2% (218.4% to 268.4%) | 45.4 (29.2 to 65.5) | 46.0 (29.6 to 67.0) | 1.2% (-4.3% to 7.1%) | 734.0 (586.5 to 906.4) | 2,510.2 (1,953.1 to 3,095.2) | 242.0% (222.1% to 262.6%) | 1,699.2 (1,342.0 to 2,096.0) | 1,723.2 (1,357.0 to 2,108.6) | 1.4% (-3.2% to 6.5%) | 77.8 (62.0 to 96.8) | 282.1 (218.1 to 350.0) | 262.6% (238.0% to 288.6%) | 169.4 (131.8 to 214.6) | 170.9 (130.1 to 215.8) | 0.9% (-4.1% to 6.4%) |
| Brunei | Female | Other oral disorders | 68.1 (41.5 to 101.4) | 135.4 (82.9 to 202.7) | 98.9% (89.2% to 109.4%) | 59.3 (36.2 to 88.4) | 59.2 (36.4 to 88.8) | 0.0% (-4.3% to 4.2%) | 2,332.9 (2,211.9 to 2,460.7) | 4,677.7 (4,432.7 to 4,911.1) | 100.5% (96.7% to 104.4%) | 2,047.7 (1,940.4 to 2,139.5) | 2,047.7 (1,940.4 to 2,139.5) | 0.0% (0.0% to 0.0%) | NA | NA | NA | NA | NA | NA |
| Brunei | Male | Main Oral disorders | 192.4 (104.9 to 317.7) | 423.8 (238.2 to 694.3) | 120.2% (86.5% to 159.2%) | 202.8 (115.8 to 329.2) | 190.8 (109.5 to 303.2) | -5.9% (-18.9% to 7.6%) | 47,100.1 (42,052.7 to 53,678.0) | 82,143.1 (71,924.2 to 93,344.0) | 74.4% (56.2% to 93.6%) | 36,482.4 (32,918.3 to 40,743.7) | 35,116.5 (31,177.6 to 39,319.2) | -3.7% (-12.5% to 6.0%) | 80,878.3 (69,381.0 to 93,312.9) | 122,336.9 (103,434.1 to 141,114.7) | 51.3% (37.2% to 65.3%) | 53,204.9 (45,808.9 to 61,355.4) | 53,342.8 (44,577.8 to 61,822.1) | 0.3% (-7.7% to 8.1%) |
| Brunei | Male | Caries of deciduous teeth | 5.0 (2.2 to 9.6) | 5.0 (2.0 to 9.9) | -0.1% (-12.8% to 14.2%) | 3.0 (1.3 to 5.7) | 3.0 (1.2 to 5.9) | -0.1% (-12.7% to 13.5%) | 13,064.3 (10,234.8 to 16,241.1) | 13,000.2 (9,915.5 to 16,233.5) | -0.5% (-7.2% to 6.5%) | 7,776.6 (6,090.1 to 9,714.3) | 7,737.5 (5,902.6 to 9,592.2) | -0.5% (-7.5% to 5.9%) | 32,714.4 (24,254.1 to 44,042.6) | 34,433.2 (25,219.3 to 47,163.5) | 5.3% (-2.1% to 15.2%) | 20,025.7 (14,727.2 to 27,436.5) | 20,118.6 (14,853.5 to 27,078.5) | 0.5% (-6.0% to 9.3%) |
| Brunei | Male | Caries of permanent teeth | 23.1 (9.8 to 45.1) | 43.4 (18.7 to 82.0) | 87.7% (66.0% to 112.9%) | 16.1 (6.9 to 31.5) | 16.3 (7.1 to 30.6) | 1.2% (-10.6% to 14.3%) | 23,238.7 (18,776.0 to 29,372.6) | 43,713.6 (34,787.8 to 54,307.9) | 88.1% (67.3% to 110.9%) | 16,266.9 (13,435.6 to 19,833.8) | 16,456.7 (13,187.6 to 20,143.2) | 1.2% (-10.0% to 12.6%) | 46,903.8 (39,116.0 to 54,427.8) | 85,112.3 (70,388.1 to 100,567.2) | 81.5% (58.0% to 103.3%) | 31,939.2 (26,850.3 to 36,887.5) | 32,095.5 (26,561.6 to 37,812.3) | 0.5% (-11.8% to 13.0%) |
| Brunei | Male | Periodontal diseases | 74.0 (29.2 to 157.9) | 166.0 (65.9 to 339.2) | 124.4% (53.0% to 238.5%) | 80.8 (32.4 to 169.7) | 67.6 (27.0 to 134.7) | -16.3% (-39.8% to 20.2%) | 11,273.0 (8,133.9 to 14,437.8) | 25,570.2 (18,911.4 to 33,407.0) | 126.8% (54.2% to 243.5%) | 12,444.4 (9,780.0 to 15,230.8) | 10,496.2 (8,105.9 to 13,143.7) | -15.7% (-39.3% to 20.0%) | 1,159.0 (855.0 to 1,434.0) | 2,453.9 (1,896.9 to 3,094.5) | 111.7% (51.1% to 203.9%) | 1,057.8 (852.4 to 1,233.4) | 943.8 (761.8 to 1,138.6) | -10.8% (-31.4% to 18.9%) |
| Brunei | Male | Edentulism | 27.2 (17.1 to 39.1) | 88.5 (55.2 to 127.6) | 225.3% (199.2% to 253.3%) | 55.0 (34.9 to 80.1) | 56.2 (36.3 to 81.0) | 2.1% (-5.0% to 8.7%) | 997.3 (768.1 to 1,253.3) | 3,239.3 (2,449.9 to 4,123.5) | 224.8% (202.6% to 248.6%) | 2,043.9 (1,577.7 to 2,553.4) | 2,092.5 (1,609.9 to 2,645.3) | 2.4% (-4.4% to 7.8%) | 101.1 (78.8 to 128.1) | 337.5 (259.2 to 426.8) | 233.9% (208.6% to 257.9%) | 182.1 (141.3 to 233.4) | 184.8 (142.5 to 230.5) | 1.5% (-5.0% to 7.4%) |
| Brunei | Male | Other oral disorders | 63.1 (38.1 to 96.6) | 120.9 (74.2 to 183.2) | 91.4% (80.7% to 101.4%) | 47.9 (29.1 to 73.2) | 47.8 (29.3 to 72.1) | -0.3% (-5.1% to 4.3%) | 2,149.5 (2,039.6 to 2,271.3) | 4,153.8 (3,940.8 to 4,369.8) | 93.3% (90.2% to 96.4%) | 1,648.1 (1,571.3 to 1,728.3) | 1,648.1 (1,571.3 to 1,728.3) | 0.0% (0.0% to 0.0%) | NA | NA | NA | NA | NA | NA |
| Cambodia | Both | Main Oral disorders | 17,489.4 (10,282.7 to 27,093.0) | 38,903.9 (22,273.4 to 60,032.3) | 122.4% (113.3% to 132.9%) | 274.0 (165.0 to 410.3) | 265.3 (154.8 to 402.2) | -3.2% (-7.5% to 1.1%) | 5,152,959.8 (4,638,394.2 to 5,731,526.8) | 8,747,307.4 (7,811,105.1 to 9,669,052.5) | 69.8% (63.2% to 77.5%) | 54,091.0 (49,051.4 to 59,291.5) | 52,574.7 (47,343.8 to 57,570.9) | -2.8% (-5.8% to 0.4%) | 6,821,688.5 (5,744,659.3 to 8,296,493.7) | 10,298,408.5 (8,859,162.2 to 12,075,339.0) | 51.0% (42.1% to 60.8%) | 57,562.7 (49,655.3 to 67,776.7) | 58,768.3 (50,721.6 to 68,562.2) | 2.1% (-3.2% to 6.6%) |
| Cambodia | Both | Caries of deciduous teeth | 577.5 (242.2 to 1,139.8) | 600.7 (251.3 to 1,197.5) | 4.0% (-5.5% to 17.0%) | 3.5 (1.5 to 6.9) | 3.4 (1.4 to 6.8) | -1.9% (-10.4% to 10.1%) | 1,521,325.6 (1,225,609.4 to 1,800,955.3) | 1,569,584.0 (1,252,258.5 to 1,889,320.6) | 3.2% (-4.1% to 13.4%) | 9,123.6 (7,319.3 to 10,818.8) | 8,875.4 (7,078.6 to 10,675.8) | -2.7% (-9.4% to 7.2%) | 3,242,683.6 (2,366,373.2 to 4,750,135.8) | 3,535,268.6 (2,596,918.4 to 5,145,827.1) | 9.0% (-0.8% to 17.8%) | 20,056.9 (14,471.3 to 30,121.4) | 19,862.0 (14,625.3 to 28,694.1) | -1.0% (-9.2% to 8.1%) |
| Cambodia | Both | Caries of permanent teeth | 3,357.4 (1,431.2 to 6,389.4) | 6,122.5 (2,717.4 to 11,807.6) | 82.4% (70.4% to 96.8%) | 38.5 (16.7 to 72.2) | 36.3 (16.2 to 69.9) | -5.8% (-11.6% to -0.4%) | 3,399,725.6 (2,853,951.5 to 3,998,209.2) | 6,207,864.4 (5,217,005.1 to 7,277,496.3) | 82.6% (71.8% to 96.1%) | 39,329.2 (34,010.4 to 45,078.8) | 36,943.8 (31,237.0 to 42,446.6) | -6.1% (-11.5% to -1.1%) | 3,511,163.9 (2,999,251.8 to 4,009,486.5) | 6,578,429.9 (5,666,830.8 to 7,489,903.5) | 87.4% (76.5% to 98.8%) | 36,364.1 (31,815.6 to 40,885.1) | 37,688.4 (32,668.1 to 42,766.9) | 3.6% (-2.5% to 8.6%) |
| Cambodia | Both | Periodontal diseases | 3,118.2 (1,215.4 to 6,899.4) | 9,881.6 (3,822.3 to 21,568.7) | 216.9% (196.2% to 237.6%) | 52.3 (20.4 to 115.1) | 64.8 (25.0 to 141.4) | 24.0% (14.9% to 31.8%) | 480,130.8 (330,814.2 to 635,253.8) | 1,518,824.3 (1,081,626.6 to 1,995,925.7) | 216.3% (195.8% to 237.3%) | 8,099.3 (5,719.8 to 10,637.2) | 10,014.8 (7,241.5 to 12,936.9) | 23.7% (15.0% to 31.4%) | 50,581.7 (36,125.9 to 64,549.5) | 144,879.1 (107,300.0 to 181,370.0) | 186.4% (169.3% to 206.0%) | 796.7 (586.3 to 1,004.0) | 922.5 (694.6 to 1,128.3) | 15.8% (9.2% to 23.1%) |
| Cambodia | Both | Edentulism | 5,622.7 (3,583.3 to 7,928.2) | 13,176.3 (8,282.6 to 18,517.6) | 134.3% (123.8% to 145.2%) | 126.1 (80.5 to 178.0) | 107.0 (68.6 to 150.9) | -15.2% (-18.3% to -11.5%) | 205,014.9 (161,091.5 to 252,516.6) | 481,220.3 (373,383.5 to 594,643.6) | 134.7% (124.7% to 144.1%) | 4,663.3 (3,676.5 to 5,686.8) | 3,953.4 (3,095.4 to 4,841.9) | -15.2% (-18.3% to -11.9%) | 17,259.3 (13,772.3 to 21,183.6) | 39,831.0 (31,599.1 to 49,161.4) | 130.8% (119.8% to 141.7%) | 345.0 (276.8 to 422.1) | 295.4 (237.0 to 363.2) | -14.4% (-17.3% to -11.2%) |
| Cambodia | Both | Other oral disorders | 4,813.7 (2,932.4 to 7,086.9) | 9,122.8 (5,620.1 to 13,763.5) | 89.5% (82.6% to 96.8%) | 53.7 (32.9 to 79.5) | 53.8 (33.3 to 81.2) | 0.2% (-2.9% to 3.1%) | 165,928.1 (158,248.3 to 173,106.8) | 313,819.5 (300,391.8 to 326,708.9) | 89.1% (86.3% to 91.9%) | 1,866.0 (1,784.2 to 1,938.7) | 1,858.1 (1,777.7 to 1,930.0) | -0.4% (-0.6% to -0.2%) | NA | NA | NA | NA | NA | NA |
| Cambodia | Female | Main Oral disorders | 10,430.4 (6,156.8 to 15,999.9) | 22,777.9 (13,378.9 to 34,764.5) | 118.4% (109.3% to 128.5%) | 297.0 (180.1 to 442.4) | 286.2 (169.3 to 431.4) | -3.6% (-7.9% to 0.7%) | 2,751,034.5 (2,480,090.7 to 3,061,592.9) | 4,547,411.0 (4,067,191.0 to 5,028,528.1) | 65.3% (56.5% to 74.5%) | 54,585.5 (49,467.9 to 59,865.5) | 53,125.6 (47,842.2 to 58,428.2) | -2.7% (-7.2% to 1.9%) | 3,502,400.1 (2,961,728.8 to 4,213,670.5) | 5,138,981.7 (4,414,969.1 to 6,014,777.4) | 46.7% (37.2% to 58.8%) | 57,224.3 (49,279.6 to 67,138.6) | 58,520.8 (50,408.1 to 68,357.8) | 2.3% (-3.5% to 9.8%) |
| Cambodia | Female | Caries of deciduous teeth | 284.7 (119.0 to 552.3) | 290.5 (126.3 to 580.7) | 2.1% (-13.9% to 17.4%) | 3.5 (1.4 to 6.7) | 3.4 (1.5 to 6.7) | -2.5% (-18.1% to 12.2%) | 751,238.8 (598,844.6 to 893,873.0) | 758,731.7 (605,777.2 to 916,093.3) | 1.0% (-10.8% to 11.9%) | 9,113.6 (7,276.1 to 10,885.7) | 8,791.7 (7,014.9 to 10,597.9) | -3.5% (-14.6% to 6.8%) | 1,606,308.1 (1,164,371.8 to 2,337,375.8) | 1,721,434.1 (1,252,223.6 to 2,529,503.0) | 7.2% (-3.0% to 23.7%) | 20,037.8 (14,312.6 to 29,819.1) | 19,837.4 (14,457.3 to 28,957.6) | -1.0% (-11.0% to 14.5%) |
| Cambodia | Female | Caries of permanent teeth | 1,824.4 (781.6 to 3,483.0) | 3,186.2 (1,404.4 to 6,077.1) | 74.6% (59.3% to 92.7%) | 38.7 (16.7 to 73.4) | 36.7 (16.2 to 69.6) | -5.1% (-13.2% to 3.2%) | 1,853,121.0 (1,565,978.0 to 2,183,192.0) | 3,244,724.4 (2,727,072.0 to 3,804,435.8) | 75.1% (61.0% to 91.3%) | 39,619.5 (34,276.9 to 45,391.6) | 37,456.8 (31,487.7 to 43,536.8) | -5.5% (-13.3% to 2.6%) | 1,856,604.4 (1,595,875.2 to 2,104,874.8) | 3,315,025.0 (2,855,784.9 to 3,786,848.4) | 78.6% (66.5% to 91.4%) | 36,006.7 (31,516.2 to 40,708.9) | 37,431.1 (32,320.4 to 42,647.6) | 4.0% (-3.3% to 10.7%) |
| Cambodia | Female | Periodontal diseases | 1,703.6 (675.7 to 3,817.5) | 5,172.9 (2,027.0 to 11,525.5) | 203.7% (180.8% to 235.8%) | 50.5 (19.7 to 111.9) | 63.0 (24.5 to 140.1) | 24.8% (14.3% to 35.8%) | 263,578.0 (178,636.7 to 352,354.0) | 798,935.6 (574,426.0 to 1,039,839.3) | 203.1% (181.0% to 234.4%) | 7,858.9 (5,490.9 to 10,468.0) | 9,763.2 (7,049.2 to 12,637.5) | 24.2% (13.8% to 35.4%) | 28,162.6 (20,139.8 to 36,155.4) | 76,490.2 (57,080.8 to 94,100.6) | 171.6% (153.2% to 196.5%) | 792.1 (575.5 to 1,004.1) | 916.6 (692.1 to 1,112.5) | 15.7% (7.7% to 24.0%) |
| Cambodia | Female | Edentulism | 3,862.8 (2,454.3 to 5,437.7) | 9,112.9 (5,748.2 to 12,822.7) | 135.9% (122.7% to 149.2%) | 147.3 (93.3 to 207.2) | 125.7 (80.4 to 176.6) | -14.7% (-18.6% to -10.0%) | 141,126.4 (111,079.4 to 174,481.1) | 333,516.9 (260,502.5 to 414,256.2) | 136.3% (123.4% to 148.3%) | 5,449.9 (4,309.6 to 6,643.5) | 4,640.1 (3,651.5 to 5,647.4) | -14.9% (-18.9% to -10.5%) | 11,325.0 (9,082.4 to 13,634.6) | 26,032.4 (20,695.0 to 31,895.9) | 129.9% (115.6% to 142.9%) | 387.7 (314.3 to 470.7) | 335.6 (268.2 to 408.8) | -13.4% (-17.5% to -9.4%) |
| Cambodia | Female | Other oral disorders | 2,755.0 (1,680.2 to 4,075.5) | 5,015.3 (3,083.1 to 7,564.5) | 82.0% (72.8% to 90.3%) | 57.1 (35.2 to 84.6) | 57.5 (35.5 to 86.4) | 0.7% (-3.4% to 4.6%) | 95,416.6 (90,627.2 to 100,045.5) | 173,403.3 (165,234.1 to 181,422.2) | 81.7% (79.3% to 84.3%) | 1,993.8 (1,903.3 to 2,082.6) | 1,993.8 (1,903.3 to 2,082.6) | 0.0% (0.0% to 0.0%) | NA | NA | NA | NA | NA | NA |
| Cambodia | Male | Main Oral disorders | 7,059.0 (4,077.4 to 11,101.7) | 16,126.0 (9,133.0 to 25,496.5) | 128.4% (117.0% to 142.4%) | 243.7 (144.6 to 367.9) | 238.4 (138.4 to 367.7) | -2.2% (-7.2% to 2.8%) | 2,401,925.3 (2,133,408.2 to 2,693,331.4) | 4,199,896.4 (3,732,008.7 to 4,677,912.3) | 74.9% (67.3% to 84.1%) | 53,491.0 (48,168.4 to 58,889.9) | 51,934.1 (46,635.6 to 57,009.0) | -2.9% (-6.6% to 1.8%) | 3,319,288.4 (2,751,985.8 to 4,087,927.3) | 5,159,426.8 (4,447,297.9 to 6,129,877.6) | 55.4% (43.0% to 68.7%) | 57,977.4 (49,878.0 to 68,591.1) | 59,027.9 (51,113.2 to 69,367.9) | 1.8% (-5.2% to 7.9%) |
| Cambodia | Male | Caries of deciduous teeth | 292.8 (124.8 to 587.7) | 310.2 (128.2 to 623.3) | 6.0% (-6.4% to 22.2%) | 3.5 (1.5 to 6.9) | 3.4 (1.4 to 6.9) | -1.3% (-12.9% to 13.9%) | 770,086.7 (617,286.3 to 912,659.6) | 810,852.3 (649,722.2 to 993,476.7) | 5.3% (-3.5% to 17.1%) | 9,133.3 (7,343.1 to 10,864.1) | 8,955.3 (7,172.9 to 10,964.4) | -1.9% (-10.1% to 9.1%) | 1,636,375.5 (1,160,684.5 to 2,362,567.4) | 1,813,834.5 (1,322,394.7 to 2,725,427.3) | 10.8% (-4.5% to 23.8%) | 20,075.2 (14,010.0 to 29,901.2) | 19,884.6 (14,566.4 to 29,614.1) | -0.9% (-14.0% to 11.4%) |
| Cambodia | Male | Caries of permanent teeth | 1,533.0 (643.6 to 2,906.2) | 2,936.3 (1,306.3 to 5,745.2) | 91.5% (76.8% to 112.3%) | 38.3 (16.7 to 71.8) | 35.8 (16.1 to 70.5) | -6.5% (-12.8% to 1.7%) | 1,546,604.7 (1,278,081.1 to 1,845,036.5) | 2,963,140.1 (2,491,578.9 to 3,501,717.1) | 91.6% (77.7% to 109.6%) | 39,010.1 (33,240.7 to 44,748.7) | 36,388.0 (30,951.0 to 42,325.7) | -6.7% (-12.8% to 0.6%) | 1,654,559.5 (1,394,264.1 to 1,912,010.9) | 3,263,404.9 (2,797,596.8 to 3,686,133.4) | 97.2% (80.6% to 114.2%) | 36,808.7 (31,694.4 to 41,856.2) | 37,966.8 (32,990.8 to 42,594.6) | 3.1% (-4.3% to 10.5%) |
| Cambodia | Male | Periodontal diseases | 1,414.6 (544.1 to 3,084.6) | 4,708.7 (1,794.6 to 10,097.0) | 232.9% (208.9% to 262.1%) | 54.6 (21.0 to 118.0) | 67.1 (25.6 to 141.4) | 22.9% (14.1% to 34.1%) | 216,552.9 (149,874.5 to 289,710.4) | 719,888.7 (508,174.4 to 948,030.0) | 232.4% (207.7% to 260.5%) | 8,422.4 (6,013.6 to 11,022.5) | 10,338.0 (7,487.2 to 13,359.6) | 22.7% (14.3% to 33.7%) | 22,419.2 (16,101.6 to 28,896.3) | 68,388.8 (50,043.9 to 86,689.4) | 205.0% (181.8% to 229.7%) | 802.8 (601.1 to 1,003.7) | 927.9 (703.2 to 1,137.0) | 15.6% (8.1% to 25.9%) |
| Cambodia | Male | Edentulism | 1,760.0 (1,093.1 to 2,509.9) | 4,063.4 (2,534.0 to 5,833.8) | 130.9% (114.3% to 145.8%) | 97.8 (63.0 to 139.8) | 82.2 (53.0 to 119.0) | -15.9% (-21.2% to -11.0%) | 63,888.6 (49,538.8 to 79,625.5) | 147,703.3 (113,263.1 to 185,678.1) | 131.2% (118.5% to 143.7%) | 3,605.2 (2,807.8 to 4,476.3) | 3,034.8 (2,374.6 to 3,778.8) | -15.8% (-20.2% to -11.5%) | 5,934.2 (4,633.0 to 7,463.8) | 13,798.6 (10,828.6 to 17,307.9) | 132.5% (118.4% to 146.3%) | 290.7 (229.7 to 362.1) | 248.5 (198.9 to 309.7) | -14.5% (-19.5% to -9.4%) |
| Cambodia | Male | Other oral disorders | 2,058.7 (1,236.8 to 3,060.5) | 4,107.5 (2,531.8 to 6,150.2) | 99.5% (88.5% to 111.0%) | 49.5 (29.9 to 73.1) | 49.8 (30.8 to 74.4) | 0.5% (-4.0% to 4.9%) | 70,511.5 (67,009.9 to 74,306.2) | 140,416.2 (133,582.0 to 147,343.3) | 99.1% (95.0% to 102.6%) | 1,710.3 (1,630.8 to 1,786.0) | 1,710.3 (1,630.8 to 1,786.0) | 0.0% (0.0% to 0.0%) | NA | NA | NA | NA | NA | NA |
| Indonesia | Both | Main Oral disorders | 387,503.7 (219,746.0 to 610,402.7) | 785,768.8 (460,111.9 to 1,231,329.9) | 102.8% (89.8% to 117.5%) | 292.6 (171.2 to 448.3) | 287.2 (170.5 to 440.4) | -1.8% (-9.4% to 6.9%) | 88,901,933.1 (79,867,315.2 to 98,355,750.0) | 140,809,017.5 (127,141,615.5 to 153,840,370.4) | 58.4% (53.5% to 63.8%) | 51,198.2 (46,395.3 to 55,699.7) | 50,303.0 (45,700.8 to 54,773.1) | -1.7% (-3.1% to -0.3%) | 120,555,785.2 (102,559,572.3 to 143,254,689.2) | 161,189,388.4 (140,463,018.2 to 186,837,544.2) | 33.7% (28.2% to 40.0%) | 58,941.2 (51,073.6 to 68,775.9) | 59,291.0 (51,449.4 to 69,123.8) | 0.6% (-1.7% to 3.4%) |
| Indonesia | Both | Caries of deciduous teeth | 8,155.1 (3,556.9 to 16,155.6) | 8,156.5 (3,504.1 to 16,104.6) | 0.0% (-3.6% to 3.3%) | 3.5 (1.5 to 6.9) | 3.6 (1.5 to 7.0) | 1.7% (-2.0% to 4.9%) | 21,394,417.6 (17,755,263.9 to 25,465,712.3) | 21,317,995.5 (17,522,648.3 to 25,445,141.0) | -0.4% (-3.3% to 2.3%) | 9,198.9 (7,661.7 to 10,920.3) | 9,317.4 (7,663.6 to 11,066.1) | 1.3% (-1.8% to 4.1%) | 49,494,056.4 (35,749,029.7 to 71,641,378.6) | 49,178,160.2 (35,469,215.7 to 72,571,270.3) | -0.6% (-5.6% to 6.0%) | 20,859.2 (15,171.6 to 29,940.8) | 21,010.9 (15,322.3 to 30,546.3) | 0.7% (-3.7% to 6.8%) |
| Indonesia | Both | Caries of permanent teeth | 54,753.9 (23,832.2 to 106,496.4) | 88,589.1 (38,541.4 to 174,436.2) | 61.8% (53.3% to 70.0%) | 31.7 (14.0 to 61.2) | 30.3 (13.3 to 59.2) | -4.3% (-6.5% to -1.7%) | 55,327,423.0 (46,525,198.2 to 65,226,282.5) | 89,851,881.3 (76,783,390.7 to 104,871,373.6) | 62.4% (54.1% to 70.9%) | 32,234.4 (27,516.7 to 37,429.6) | 30,814.7 (26,496.8 to 35,610.3) | -4.4% (-6.4% to -2.0%) | 69,024,636.9 (59,439,925.0 to 78,379,172.5) | 107,767,679.6 (94,432,770.2 to 121,309,355.7) | 56.1% (49.8% to 64.1%) | 36,621.3 (31,825.8 to 41,414.3) | 36,825.7 (32,315.2 to 41,419.2) | 0.6% (-1.8% to 3.5%) |
| Indonesia | Both | Periodontal diseases | 117,028.2 (46,704.4 to 245,614.8) | 268,116.9 (106,780.4 to 566,039.2) | 129.1% (121.3% to 137.2%) | 86.0 (34.1 to 180.9) | 88.9 (35.4 to 187.1) | 3.5% (1.7% to 5.2%) | 17,894,238.7 (13,214,232.6 to 22,393,604.2) | 41,014,678.7 (30,942,525.7 to 50,701,864.7) | 129.2% (121.7% to 137.5%) | 13,221.0 (10,059.8 to 16,313.8) | 13,659.8 (10,473.1 to 16,815.6) | 3.3% (1.6% to 5.1%) | 1,686,125.8 (1,267,400.1 to 2,014,477.2) | 3,442,904.3 (2,713,509.0 to 4,006,775.4) | 104.2% (94.5% to 114.4%) | 1,136.0 (899.4 to 1,322.3) | 1,148.6 (912.5 to 1,334.3) | 1.1% (0.0% to 2.3%) |
| Indonesia | Both | Edentulism | 113,573.1 (71,917.7 to 159,126.9) | 263,664.5 (168,974.6 to 372,397.0) | 132.2% (95.0% to 187.3%) | 117.5 (74.6 to 166.4) | 110.5 (70.7 to 154.8) | -6.0% (-21.1% to 16.8%) | 4,118,763.4 (3,248,185.7 to 5,055,290.3) | 9,563,685.8 (8,035,652.5 to 11,275,083.5) | 132.2% (94.4% to 187.6%) | 4,322.9 (3,413.3 to 5,272.2) | 4,061.2 (3,469.3 to 4,840.1) | -6.1% (-20.8% to 16.9%) | 350,966.1 (279,729.7 to 430,345.9) | 800,644.3 (676,539.4 to 959,633.9) | 128.1% (91.7% to 185.8%) | 324.6 (262.6 to 395.8) | 305.8 (260.9 to 362.7) | -5.8% (-20.7% to 17.3%) |
| Indonesia | Both | Other oral disorders | 93,993.3 (57,914.0 to 140,927.3) | 157,241.6 (96,586.1 to 235,117.9) | 67.3% (64.4% to 70.3%) | 54.0 (33.2 to 80.7) | 54.0 (33.2 to 80.8) | 0.0% (-0.9% to 0.9%) | 3,222,803.2 (3,078,551.1 to 3,374,802.5) | 5,413,629.7 (5,172,673.8 to 5,665,289.5) | 68.0% (65.5% to 70.4%) | 1,863.6 (1,786.1 to 1,947.5) | 1,860.7 (1,783.4 to 1,944.8) | -0.2% (-0.2% to -0.1%) | NA | NA | NA | NA | NA | NA |
| Indonesia | Female | Main Oral disorders | 219,577.6 (126,694.8 to 344,648.5) | 443,861.1 (265,573.7 to 683,485.4) | 102.1% (87.8% to 119.1%) | 326.4 (194.0 to 496.5) | 321.3 (193.9 to 489.5) | -1.6% (-9.7% to 7.7%) | 45,306,587.6 (40,943,726.7 to 49,955,250.3) | 71,246,303.6 (64,609,376.0 to 77,666,638.6) | 57.3% (52.4% to 62.4%) | 52,076.7 (47,358.5 to 56,548.2) | 51,272.3 (46,756.2 to 55,778.4) | -1.5% (-3.2% to 0.1%) | 59,983,974.3 (51,140,786.1 to 71,132,273.6) | 79,299,394.3 (69,183,836.7 to 91,781,749.7) | 32.2% (26.6% to 38.6%) | 59,020.8 (51,140.1 to 68,982.1) | 59,368.6 (51,438.8 to 69,297.1) | 0.6% (-2.2% to 3.5%) |
| Indonesia | Female | Caries of deciduous teeth | 3,975.3 (1,727.6 to 7,917.9) | 3,972.3 (1,700.9 to 7,867.5) | -0.1% (-4.5% to 4.1%) | 3.5 (1.5 to 7.0) | 3.6 (1.5 to 7.0) | 1.3% (-3.1% to 5.5%) | 10,420,192.5 (8,612,048.4 to 12,440,318.6) | 10,387,231.9 (8,562,275.1 to 12,412,672.9) | -0.3% (-3.8% to 3.3%) | 9,206.2 (7,614.4 to 10,973.9) | 9,304.9 (7,685.2 to 11,052.7) | 1.1% (-2.4% to 4.7%) | 24,087,197.8 (17,383,995.7 to 34,606,529.5) | 23,947,273.1 (17,207,171.3 to 35,071,541.8) | -0.6% (-7.2% to 6.7%) | 20,855.4 (15,169.0 to 29,699.9) | 20,984.1 (15,232.2 to 30,310.2) | 0.6% (-5.5% to 7.7%) |
| Indonesia | Female | Caries of permanent teeth | 27,650.4 (11,991.9 to 53,665.7) | 43,811.5 (19,149.2 to 86,586.4) | 58.4% (49.9% to 67.3%) | 31.6 (14.0 to 61.1) | 30.3 (13.3 to 59.4) | -4.2% (-7.1% to -1.0%) | 28,015,373.1 (23,616,235.4 to 33,027,763.8) | 44,626,354.5 (38,128,281.9 to 52,018,074.7) | 59.3% (51.0% to 68.0%) | 32,260.2 (27,659.7 to 37,417.9) | 30,905.2 (26,591.3 to 35,720.9) | -4.2% (-6.8% to -1.1%) | 34,768,308.4 (29,922,873.6 to 39,640,037.2) | 53,062,745.6 (46,492,287.9 to 59,746,933.1) | 52.6% (46.3% to 60.2%) | 36,599.8 (31,655.2 to 41,630.6) | 36,820.2 (32,199.1 to 41,582.5) | 0.6% (-2.3% to 3.9%) |
| Indonesia | Female | Periodontal diseases | 63,180.5 (25,415.6 to 132,277.8) | 141,860.0 (56,895.6 to 298,110.5) | 124.5% (116.6% to 132.6%) | 90.0 (36.2 to 189.1) | 93.7 (37.7 to 196.7) | 4.1% (2.0% to 6.6%) | 9,682,984.3 (7,244,052.7 to 12,024,597.3) | 21,776,806.9 (16,668,217.8 to 26,801,515.2) | 124.9% (117.2% to 132.7%) | 13,867.0 (10,636.3 to 17,108.1) | 14,426.0 (11,212.6 to 17,652.7) | 4.0% (2.1% to 6.5%) | 910,721.2 (694,951.2 to 1,087,673.8) | 1,798,239.4 (1,436,305.2 to 2,085,741.8) | 97.5% (87.2% to 108.0%) | 1,187.6 (946.9 to 1,377.0) | 1,203.8 (962.0 to 1,396.1) | 1.4% (0.0% to 3.0%) |
| Indonesia | Female | Edentulism | 73,965.5 (46,776.0 to 104,027.1) | 170,793.9 (109,526.6 to 238,974.0) | 130.9% (94.2% to 185.6%) | 143.4 (90.7 to 201.8) | 135.9 (87.6 to 189.9) | -5.2% (-20.5% to 17.5%) | 2,685,142.2 (2,120,658.6 to 3,288,605.4) | 6,210,747.2 (5,247,362.7 to 7,286,728.8) | 131.3% (94.6% to 186.1%) | 5,276.1 (4,175.5 to 6,417.1) | 5,000.0 (4,279.8 to 5,895.4) | -5.2% (-20.2% to 17.8%) | 217,747.0 (174,455.0 to 265,223.6) | 491,136.2 (418,080.2 to 584,524.5) | 125.6% (89.1% to 181.7%) | 377.9 (308.4 to 457.6) | 360.4 (310.5 to 422.7) | -4.6% (-19.4% to 18.0%) |
| Indonesia | Female | Other oral disorders | 50,806.0 (31,439.7 to 75,551.2) | 83,423.5 (51,414.3 to 123,222.1) | 64.2% (60.5% to 67.7%) | 57.8 (35.7 to 85.6) | 57.9 (35.7 to 85.4) | 0.1% (-1.2% to 1.3%) | 1,747,307.9 (1,663,425.5 to 1,835,253.6) | 2,885,273.4 (2,745,816.3 to 3,041,248.4) | 65.1% (62.5% to 67.7%) | 2,003.2 (1,910.4 to 2,104.5) | 2,003.2 (1,910.4 to 2,104.5) | 0.0% (0.0% to 0.0%) | NA | NA | NA | NA | NA | NA |
| Indonesia | Male | Main Oral disorders | 167,926.1 (93,592.4 to 265,376.8) | 341,907.7 (193,658.3 to 549,048.2) | 103.6% (92.1% to 117.0%) | 256.7 (149.0 to 401.3) | 251.9 (146.4 to 394.2) | -1.9% (-8.7% to 6.0%) | 43,595,345.4 (38,993,632.8 to 48,449,813.8) | 69,562,713.9 (62,421,901.3 to 76,317,416.8) | 59.6% (54.0% to 65.5%) | 50,284.0 (45,238.4 to 54,933.6) | 49,323.8 (44,685.8 to 53,766.7) | -1.9% (-3.6% to -0.2%) | 60,571,810.9 (51,231,428.7 to 72,295,751.1) | 81,889,994.1 (71,125,119.6 to 95,010,140.7) | 35.2% (29.0% to 41.7%) | 58,859.6 (50,992.4 to 68,738.5) | 59,213.8 (51,093.6 to 69,139.3) | 0.6% (-2.1% to 3.5%) |
| Indonesia | Male | Caries of deciduous teeth | 4,179.8 (1,814.4 to 8,238.7) | 4,184.3 (1,784.6 to 8,246.5) | 0.1% (-5.2% to 4.8%) | 3.5 (1.5 to 6.9) | 3.6 (1.5 to 7.0) | 2.0% (-3.3% to 6.7%) | 10,974,225.1 (9,138,922.2 to 13,088,588.4) | 10,930,763.6 (8,946,405.8 to 12,992,582.1) | -0.4% (-4.4% to 3.1%) | 9,192.0 (7,676.4 to 10,907.3) | 9,329.3 (7,637.6 to 11,040.8) | 1.5% (-2.5% to 5.0%) | 25,406,858.6 (18,444,366.6 to 37,042,381.6) | 25,230,887.1 (18,355,207.0 to 37,499,728.4) | -0.7% (-6.4% to 6.0%) | 20,862.8 (15,242.0 to 30,172.0) | 21,036.2 (15,394.2 to 30,660.1) | 0.8% (-4.9% to 6.7%) |
| Indonesia | Male | Caries of permanent teeth | 27,103.5 (11,773.5 to 52,836.7) | 44,777.7 (19,404.0 to 87,883.0) | 65.2% (56.0% to 74.5%) | 31.7 (14.1 to 61.5) | 30.3 (13.2 to 59.0) | -4.4% (-7.3% to -1.4%) | 27,312,050.0 (22,917,074.7 to 32,415,481.1) | 45,225,526.8 (38,601,206.7 to 53,036,739.9) | 65.6% (56.9% to 74.8%) | 32,207.5 (27,413.5 to 37,428.0) | 30,723.0 (26,417.1 to 35,545.7) | -4.6% (-7.2% to -1.6%) | 34,256,328.5 (29,531,652.4 to 38,733,219.1) | 54,704,934.0 (47,845,604.8 to 61,478,297.6) | 59.7% (52.6% to 67.7%) | 36,644.6 (31,796.8 to 41,293.8) | 36,830.7 (32,134.2 to 41,438.9) | 0.5% (-2.6% to 3.7%) |
| Indonesia | Male | Periodontal diseases | 53,847.7 (21,223.2 to 113,799.2) | 126,256.9 (50,083.5 to 270,017.3) | 134.5% (126.1% to 144.3%) | 82.0 (32.6 to 173.2) | 84.4 (33.6 to 179.4) | 3.0% (0.7% to 5.3%) | 8,211,254.5 (5,947,723.4 to 10,373,426.1) | 19,237,871.8 (14,342,457.0 to 23,935,691.8) | 134.3% (126.1% to 144.0%) | 12,578.1 (9,452.8 to 15,602.3) | 12,925.8 (9,805.8 to 16,008.2) | 2.8% (0.5% to 5.1%) | 775,404.6 (577,984.7 to 936,146.5) | 1,644,664.8 (1,278,364.5 to 1,933,042.2) | 112.1% (102.7% to 123.7%) | 1,082.6 (850.3 to 1,274.9) | 1,095.9 (860.6 to 1,283.7) | 1.2% (0.0% to 2.9%) |
| Indonesia | Male | Edentulism | 39,607.7 (25,043.6 to 55,894.3) | 92,870.7 (58,929.4 to 131,464.0) | 134.5% (95.3% to 194.5%) | 89.5 (57.4 to 128.0) | 83.4 (53.4 to 115.6) | -6.8% (-22.5% to 17.2%) | 1,433,621.2 (1,118,352.1 to 1,761,776.7) | 3,352,938.6 (2,803,393.9 to 4,034,214.1) | 133.9% (94.1% to 194.2%) | 3,292.1 (2,596.5 to 4,061.5) | 3,059.6 (2,608.0 to 3,706.8) | -7.1% (-22.4% to 17.4%) | 133,219.2 (105,431.1 to 164,507.9) | 309,508.2 (259,680.8 to 378,121.8) | 132.3% (93.3% to 194.1%) | 269.5 (216.1 to 332.5) | 251.0 (214.4 to 300.1) | -6.8% (-22.0% to 17.6%) |
| Indonesia | Male | Other oral disorders | 43,187.4 (26,363.8 to 65,573.5) | 73,818.1 (45,070.6 to 111,740.7) | 70.9% (67.2% to 74.6%) | 50.0 (30.6 to 75.4) | 50.1 (30.6 to 75.8) | 0.3% (-1.0% to 1.7%) | 1,475,495.4 (1,408,279.0 to 1,554,023.5) | 2,528,356.4 (2,414,071.1 to 2,651,635.7) | 71.4% (68.7% to 74.0%) | 1,720.2 (1,646.1 to 1,803.4) | 1,720.2 (1,646.1 to 1,803.4) | 0.0% (0.0% to 0.0%) | NA | NA | NA | NA | NA | NA |
| Laos | Both | Main Oral disorders | 5,753.7 (3,454.2 to 8,882.3) | 11,653.4 (6,856.0 to 18,255.8) | 102.5% (93.7% to 114.5%) | 201.5 (122.7 to 302.3) | 191.8 (114.0 to 295.0) | -4.8% (-9.6% to 0.8%) | 1,866,654.7 (1,646,933.2 to 2,108,703.0) | 3,177,454.5 (2,759,070.2 to 3,607,393.2) | 70.2% (62.5% to 79.8%) | 46,481.5 (41,245.8 to 51,715.9) | 44,167.3 (38,822.7 to 49,492.6) | -5.0% (-8.3% to -1.0%) | 2,599,230.8 (2,153,838.7 to 3,158,483.4) | 4,215,918.9 (3,582,237.4 to 4,969,092.0) | 62.2% (52.8% to 72.2%) | 53,838.5 (45,659.4 to 63,795.0) | 54,530.0 (46,431.0 to 63,953.4) | 1.3% (-4.0% to 6.3%) |
| Laos | Both | Caries of deciduous teeth | 227.8 (98.2 to 456.9) | 271.9 (116.7 to 555.8) | 19.3% (9.8% to 29.9%) | 3.5 (1.5 to 7.0) | 3.4 (1.5 to 6.9) | -2.9% (-10.7% to 5.7%) | 596,890.1 (490,981.6 to 705,018.3) | 711,126.0 (572,855.1 to 853,764.9) | 19.1% (11.8% to 25.6%) | 9,155.6 (7,530.4 to 10,877.2) | 8,876.8 (7,155.5 to 10,674.6) | -3.0% (-9.1% to 2.5%) | 1,279,408.0 (941,399.5 to 1,845,287.3) | 1,603,287.4 (1,155,085.6 to 2,317,320.0) | 25.3% (16.9% to 37.2%) | 20,117.0 (14,649.4 to 29,538.6) | 20,189.6 (14,482.6 to 29,235.8) | 0.4% (-6.0% to 10.1%) |
| Laos | Both | Caries of permanent teeth | 1,178.3 (504.3 to 2,258.8) | 2,151.3 (911.8 to 4,133.7) | 82.6% (68.9% to 97.5%) | 32.5 (14.3 to 62.1) | 29.6 (12.7 to 56.7) | -9.0% (-14.8% to -3.0%) | 1,191,035.1 (982,085.2 to 1,426,239.0) | 2,170,654.5 (1,741,308.1 to 2,656,741.2) | 82.2% (70.0% to 96.7%) | 33,067.7 (27,924.7 to 38,726.9) | 29,964.8 (24,368.6 to 35,767.8) | -9.4% (-14.8% to -3.0%) | 1,303,807.2 (1,089,189.6 to 1,516,515.1) | 2,568,697.0 (2,181,480.9 to 2,921,028.2) | 97.0% (81.8% to 109.8%) | 33,067.3 (27,951.9 to 37,939.3) | 33,588.7 (28,606.6 to 38,143.5) | 1.6% (-6.0% to 8.0%) |
| Laos | Both | Periodontal diseases | 647.6 (248.8 to 1,413.6) | 2,210.5 (844.6 to 4,889.5) | 241.3% (222.7% to 264.1%) | 25.7 (9.6 to 56.6) | 37.1 (14.0 to 79.7) | 44.6% (36.3% to 53.1%) | 99,512.6 (68,949.0 to 133,647.7) | 338,619.1 (232,033.6 to 459,101.3) | 240.3% (222.0% to 261.8%) | 3,971.9 (2,787.2 to 5,308.1) | 5,725.3 (3,992.6 to 7,882.9) | 44.1% (36.0% to 52.4%) | 10,541.3 (7,288.9 to 13,843.0) | 33,986.8 (23,602.1 to 45,471.3) | 222.4% (201.7% to 244.2%) | 404.9 (280.3 to 538.1) | 551.5 (389.7 to 730.0) | 36.2% (26.2% to 44.6%) |
| Laos | Both | Edentulism | 1,724.0 (1,100.6 to 2,453.4) | 3,126.4 (1,986.9 to 4,472.3) | 81.4% (72.1% to 90.5%) | 86.2 (55.0 to 123.1) | 68.1 (43.5 to 97.7) | -21.0% (-24.7% to -17.4%) | 62,524.7 (48,359.4 to 76,807.1) | 113,369.9 (88,146.4 to 140,406.4) | 81.3% (73.4% to 88.6%) | 3,164.6 (2,470.9 to 3,889.4) | 2,498.9 (1,955.1 to 3,100.1) | -21.0% (-24.2% to -17.8%) | 5,474.3 (4,305.4 to 6,803.7) | 9,947.7 (7,814.8 to 12,445.3) | 81.7% (73.7% to 90.0%) | 249.3 (197.5 to 312.3) | 200.2 (158.2 to 251.7) | -19.7% (-23.2% to -15.9%) |
| Laos | Both | Other oral disorders | 1,976.1 (1,214.0 to 2,963.9) | 3,893.4 (2,379.7 to 5,835.1) | 97.0% (89.7% to 104.4%) | 53.7 (33.1 to 80.2) | 53.7 (33.0 to 80.4) | 0.0% (-3.0% to 2.9%) | 67,891.9 (64,885.8 to 70,748.6) | 133,698.1 (128,054.6 to 139,128.6) | 96.9% (94.6% to 99.2%) | 1,857.1 (1,777.2 to 1,928.7) | 1,852.3 (1,772.9 to 1,924.0) | -0.3% (-0.4% to -0.2%) | NA | NA | NA | NA | NA | NA |
| Laos | Female | Main Oral disorders | 3,181.5 (1,909.6 to 4,865.6) | 6,228.5 (3,676.7 to 9,642.9) | 95.8% (86.4% to 107.2%) | 215.5 (132.2 to 322.5) | 203.1 (121.6 to 308.5) | -5.8% (-10.8% to -0.3%) | 957,904.9 (842,817.3 to 1,081,407.5) | 1,593,122.6 (1,392,159.4 to 1,818,543.5) | 66.3% (56.5% to 76.9%) | 46,848.7 (41,564.6 to 52,294.7) | 44,499.4 (39,349.6 to 50,060.4) | -5.0% (-10.1% to 0.4%) | 1,316,895.7 (1,100,751.4 to 1,606,824.2) | 2,081,479.9 (1,767,665.5 to 2,448,161.7) | 58.1% (45.9% to 69.9%) | 54,148.7 (46,213.8 to 64,568.0) | 54,413.0 (46,164.0 to 63,919.2) | 0.5% (-6.7% to 7.5%) |
| Laos | Female | Caries of deciduous teeth | 112.4 (48.3 to 225.2) | 134.1 (57.7 to 270.9) | 19.2% (3.9% to 36.8%) | 3.5 (1.5 to 7.0) | 3.4 (1.5 to 6.9) | -2.5% (-15.0% to 12.1%) | 294,082.4 (242,329.1 to 351,276.4) | 350,050.0 (286,609.7 to 423,133.1) | 19.0% (7.8% to 32.8%) | 9,154.4 (7,564.6 to 10,955.1) | 8,908.0 (7,293.7 to 10,778.4) | -2.7% (-11.9% to 8.7%) | 634,334.9 (472,029.5 to 902,427.7) | 784,508.2 (567,077.3 to 1,124,569.9) | 23.7% (8.8% to 37.8%) | 20,230.2 (14,889.0 to 29,242.2) | 20,126.2 (14,506.3 to 28,911.7) | -0.5% (-12.4% to 11.1%) |
| Laos | Female | Caries of permanent teeth | 611.5 (261.6 to 1,164.6) | 1,077.2 (460.2 to 2,096.2) | 76.2% (60.4% to 95.4%) | 32.6 (14.2 to 62.5) | 29.7 (12.6 to 58.2) | -9.0% (-16.9% to 0.1%) | 619,869.0 (511,691.7 to 736,414.0) | 1,092,392.9 (886,352.8 to 1,347,295.3) | 76.2% (61.1% to 93.1%) | 33,314.1 (27,977.5 to 38,977.9) | 30,254.1 (24,728.3 to 36,686.1) | -9.2% (-17.4% to -0.9%) | 674,168.3 (563,178.7 to 787,923.4) | 1,274,986.9 (1,086,575.4 to 1,457,178.2) | 89.1% (70.6% to 107.5%) | 33,261.9 (28,124.1 to 38,395.8) | 33,541.7 (28,823.0 to 38,387.8) | 0.8% (-9.0% to 10.3%) |
| Laos | Female | Periodontal diseases | 312.8 (120.1 to 695.0) | 1,038.8 (402.2 to 2,253.9) | 232.1% (208.3% to 263.3%) | 23.8 (8.8 to 53.5) | 34.6 (13.2 to 75.4) | 45.3% (35.4% to 58.2%) | 48,299.5 (33,818.5 to 64,845.0) | 159,992.0 (109,801.6 to 220,376.3) | 231.2% (208.4% to 259.0%) | 3,703.1 (2,595.0 to 4,970.0) | 5,367.4 (3,726.1 to 7,434.7) | 44.9% (35.5% to 56.6%) | 5,195.7 (3,570.8 to 6,922.0) | 16,227.7 (11,168.9 to 21,944.9) | 212.3% (192.1% to 239.7%) | 384.2 (268.7 to 514.4) | 525.2 (363.4 to 708.6) | 36.7% (27.8% to 47.3%) |
| Laos | Female | Edentulism | 1,056.3 (673.6 to 1,507.9) | 1,898.1 (1,212.6 to 2,701.0) | 79.7% (66.2% to 90.9%) | 98.1 (62.3 to 141.2) | 77.8 (49.4 to 111.9) | -20.7% (-25.9% to -15.7%) | 38,364.5 (30,101.8 to 47,120.9) | 69,039.5 (53,653.9 to 85,536.8) | 80.0% (70.4% to 89.3%) | 3,601.5 (2,814.0 to 4,404.6) | 2,860.1 (2,252.9 to 3,540.3) | -20.6% (-24.6% to -16.2%) | 3,196.8 (2,515.7 to 3,932.5) | 5,757.0 (4,526.1 to 7,131.3) | 80.1% (70.7% to 91.0%) | 272.4 (215.9 to 337.4) | 219.8 (173.5 to 275.3) | -19.3% (-23.4% to -13.9%) |
| Laos | Female | Other oral disorders | 1,088.4 (671.9 to 1,620.9) | 2,080.3 (1,268.3 to 3,117.3) | 91.1% (81.2% to 100.6%) | 57.4 (35.5 to 85.5) | 57.6 (35.2 to 86.1) | 0.2% (-4.2% to 4.5%) | 37,479.5 (35,611.4 to 39,326.7) | 71,711.2 (68,217.8 to 75,167.1) | 91.3% (89.0% to 93.5%) | 1,993.8 (1,903.3 to 2,082.6) | 1,993.8 (1,903.3 to 2,082.6) | 0.0% (0.0% to 0.0%) | NA | NA | NA | NA | NA | NA |
| Laos | Male | Main Oral disorders | 2,572.2 (1,536.1 to 4,012.7) | 5,424.9 (3,157.3 to 8,495.8) | 110.9% (98.9% to 124.7%) | 186.3 (112.6 to 284.2) | 180.1 (106.7 to 277.3) | -3.3% (-8.8% to 2.7%) | 908,749.9 (795,635.9 to 1,033,999.3) | 1,584,331.9 (1,370,235.3 to 1,811,386.0) | 74.3% (64.3% to 86.2%) | 46,089.6 (40,509.7 to 51,931.9) | 43,831.3 (38,221.5 to 49,485.3) | -4.9% (-9.7% to 0.5%) | 1,282,335.1 (1,053,722.4 to 1,573,502.7) | 2,134,439.1 (1,805,132.1 to 2,531,463.4) | 66.4% (53.0% to 81.8%) | 53,519.7 (44,986.7 to 64,062.0) | 54,646.0 (46,203.1 to 64,591.5) | 2.1% (-5.1% to 9.3%) |
| Laos | Male | Caries of deciduous teeth | 115.4 (48.6 to 230.6) | 137.8 (58.4 to 279.7) | 19.5% (4.4% to 34.5%) | 3.5 (1.5 to 7.0) | 3.4 (1.4 to 6.9) | -3.2% (-15.5% to 9.3%) | 302,807.7 (245,943.4 to 360,150.8) | 361,076.0 (288,043.3 to 433,576.9) | 19.2% (8.0% to 30.3%) | 9,156.8 (7,418.3 to 10,969.8) | 8,846.6 (7,046.0 to 10,641.6) | -3.4% (-12.7% to 5.5%) | 645,073.1 (461,189.0 to 936,693.2) | 818,779.1 (583,248.7 to 1,158,975.7) | 26.9% (13.9% to 44.1%) | 20,007.3 (14,131.9 to 29,802.1) | 20,250.7 (14,391.1 to 28,741.3) | 1.2% (-9.6% to 14.9%) |
| Laos | Male | Caries of permanent teeth | 566.8 (241.7 to 1,093.9) | 1,074.0 (451.1 to 2,065.6) | 89.5% (70.6% to 109.3%) | 32.3 (14.1 to 61.3) | 29.4 (12.7 to 56.5) | -9.1% (-17.3% to 0.1%) | 571,166.1 (467,734.7 to 697,663.0) | 1,078,261.5 (853,024.2 to 1,318,155.0) | 88.8% (72.2% to 106.2%) | 32,809.0 (27,316.4 to 38,800.5) | 29,672.6 (23,896.4 to 35,704.1) | -9.6% (-17.2% to -1.4%) | 629,638.9 (521,130.7 to 734,596.6) | 1,293,710.1 (1,076,673.8 to 1,518,430.9) | 105.5% (85.2% to 122.3%) | 32,859.5 (27,604.2 to 38,051.4) | 33,636.5 (28,300.9 to 39,430.9) | 2.4% (-8.3% to 11.2%) |
| Laos | Male | Periodontal diseases | 334.8 (129.3 to 730.0) | 1,171.7 (446.4 to 2,624.8) | 250.0% (221.4% to 280.1%) | 27.7 (10.4 to 60.5) | 39.7 (15.1 to 87.2) | 43.2% (31.3% to 54.6%) | 51,213.1 (34,606.6 to 68,307.0) | 178,627.1 (122,262.5 to 240,025.7) | 248.8% (220.1% to 277.3%) | 4,270.1 (2,956.9 to 5,671.1) | 6,095.2 (4,256.2 to 8,307.7) | 42.7% (30.6% to 54.2%) | 5,345.6 (3,631.4 to 6,977.8) | 17,759.1 (12,378.5 to 23,555.7) | 232.2% (201.1% to 260.8%) | 427.8 (296.0 to 560.4) | 578.1 (414.2 to 753.5) | 35.1% (21.2% to 46.4%) |
| Laos | Male | Edentulism | 667.6 (421.3 to 943.5) | 1,228.3 (760.3 to 1,785.0) | 84.0% (72.2% to 97.5%) | 73.2 (47.5 to 103.2) | 57.9 (36.7 to 83.9) | -20.9% (-25.4% to -16.2%) | 24,160.2 (18,546.2 to 30,297.5) | 44,330.4 (34,112.3 to 54,811.6) | 83.5% (73.5% to 92.3%) | 2,682.3 (2,066.0 to 3,348.5) | 2,118.9 (1,658.1 to 2,651.4) | -21.0% (-24.9% to -17.5%) | 2,277.5 (1,771.7 to 2,891.1) | 4,190.7 (3,265.7 to 5,303.9) | 84.0% (72.7% to 94.3%) | 225.1 (176.4 to 284.0) | 180.6 (141.4 to 227.6) | -19.7% (-24.3% to -15.9%) |
| Laos | Male | Other oral disorders | 887.6 (540.4 to 1,331.6) | 1,813.1 (1,101.2 to 2,725.9) | 104.3% (93.7% to 114.7%) | 49.6 (30.4 to 73.9) | 49.8 (30.5 to 74.4) | 0.4% (-4.1% to 4.7%) | 30,412.3 (28,949.3 to 31,987.4) | 61,986.9 (58,944.5 to 65,063.1) | 103.8% (100.6% to 106.4%) | 1,710.3 (1,630.8 to 1,786.0) | 1,710.3 (1,630.8 to 1,786.0) | 0.0% (0.0% to 0.0%) | NA | NA | NA | NA | NA | NA |
| Malaysia | Both | Main Oral disorders | 38,234.5 (22,247.2 to 58,394.9) | 80,288.2 (48,105.5 to 119,475.9) | 110.0% (85.4% to 130.8%) | 312.7 (188.4 to 463.7) | 262.8 (158.7 to 388.0) | -16.0% (-24.2% to -8.5%) | 7,803,223.6 (6,923,895.1 to 8,879,435.7) | 13,164,804.5 (11,506,914.8 to 14,955,107.5) | 68.7% (55.2% to 80.7%) | 46,768.1 (42,015.1 to 52,272.6) | 41,770.2 (36,795.2 to 47,205.5) | -10.7% (-17.0% to -4.3%) | 8,619,417.0 (7,104,527.6 to 10,406,654.0) | 14,267,369.7 (11,929,035.5 to 16,902,718.8) | 65.5% (52.1% to 84.6%) | 43,460.9 (36,439.9 to 51,967.1) | 46,402.2 (38,673.8 to 55,310.3) | 6.8% (-0.7% to 17.0%) |
| Malaysia | Both | Caries of deciduous teeth | 790.8 (341.1 to 1,625.6) | 832.3 (350.6 to 1,717.4) | 5.2% (-12.3% to 21.6%) | 3.4 (1.5 to 7.0) | 3.2 (1.3 to 6.6) | -6.2% (-21.7% to 7.8%) | 2,072,622.3 (1,674,693.9 to 2,474,129.5) | 2,174,495.9 (1,719,444.1 to 2,624,869.5) | 4.9% (-12.6% to 18.9%) | 8,951.2 (7,225.0 to 10,700.7) | 8,366.9 (6,613.1 to 10,081.6) | -6.5% (-22.5% to 5.3%) | 3,839,281.1 (2,632,550.4 to 5,402,677.4) | 5,259,497.9 (3,768,510.1 to 7,427,512.8) | 37.0% (20.1% to 70.2%) | 16,738.4 (11,378.9 to 23,744.8) | 19,855.2 (14,379.1 to 27,401.2) | 18.6% (3.3% to 49.2%) |
| Malaysia | Both | Caries of permanent teeth | 4,412.4 (1,855.4 to 8,388.6) | 8,107.2 (3,502.4 to 15,675.3) | 83.7% (67.5% to 102.1%) | 26.3 (11.4 to 50.9) | 24.1 (10.5 to 46.4) | -8.5% (-15.0% to -0.2%) | 4,453,688.5 (3,547,057.5 to 5,518,681.2) | 8,208,726.1 (6,641,796.5 to 10,200,156.7) | 84.3% (68.4% to 103.0%) | 26,752.3 (21,579.8 to 32,497.0) | 24,435.3 (19,914.9 to 30,195.9) | -8.7% (-15.0% to -1.0%) | 4,594,387.7 (3,783,262.3 to 5,610,186.2) | 8,638,384.5 (7,017,142.6 to 10,181,957.6) | 88.0% (71.1% to 103.9%) | 25,273.7 (21,069.2 to 30,257.9) | 25,382.1 (20,531.6 to 30,047.1) | 0.4% (-7.2% to 7.5%) |
| Malaysia | Both | Periodontal diseases | 9,348.7 (3,734.8 to 20,433.9) | 16,074.7 (6,239.7 to 34,736.4) | 71.9% (13.0% to 161.1%) | 72.6 (28.8 to 156.2) | 50.4 (19.6 to 107.8) | -30.6% (-53.4% to 3.5%) | 1,428,886.7 (1,016,278.3 to 1,859,244.1) | 2,488,546.8 (1,849,045.1 to 3,271,223.6) | 74.2% (15.2% to 162.5%) | 11,157.0 (8,232.0 to 14,169.6) | 7,822.4 (5,895.3 to 10,193.8) | -29.9% (-52.8% to 3.5%) | 143,331.7 (104,177.9 to 177,773.4) | 261,710.4 (203,344.8 to 322,841.4) | 82.6% (32.9% to 157.6%) | 1,037.9 (809.4 to 1,242.7) | 810.1 (640.8 to 982.3) | -22.0% (-40.9% to 6.4%) |
| Malaysia | Both | Edentulism | 14,828.2 (9,555.1 to 20,847.9) | 37,600.9 (23,720.4 to 52,933.3) | 153.6% (142.3% to 165.6%) | 156.8 (100.2 to 220.1) | 131.7 (84.0 to 186.5) | -16.0% (-18.9% to -12.7%) | 539,116.9 (432,863.8 to 649,434.5) | 1,374,293.8 (1,087,075.3 to 1,669,819.5) | 154.9% (144.4% to 168.1%) | 5,755.7 (4,587.8 to 6,966.3) | 4,849.8 (3,846.8 to 5,888.9) | -15.7% (-18.3% to -12.5%) | 42,416.5 (34,549.0 to 50,664.8) | 107,777.0 (85,690.6 to 131,020.8) | 154.1% (140.7% to 170.2%) | 410.8 (334.0 to 490.3) | 354.8 (284.0 to 428.8) | -13.6% (-16.4% to -10.1%) |
| Malaysia | Both | Other oral disorders | 8,854.4 (5,407.2 to 13,167.7) | 17,673.0 (10,787.1 to 26,246.1) | 99.6% (93.1% to 106.9%) | 53.6 (33.1 to 79.6) | 53.5 (32.6 to 79.4) | -0.2% (-3.1% to 3.2%) | 303,958.8 (290,189.1 to 316,355.6) | 609,948.4 (583,592.2 to 635,300.7) | 100.7% (98.1% to 103.1%) | 1,851.9 (1,772.7 to 1,923.6) | 1,847.4 (1,768.5 to 1,919.4) | -0.2% (-0.3% to -0.2%) | NA | NA | NA | NA | NA | NA |
| Malaysia | Female | Main Oral disorders | 21,176.7 (12,574.9 to 31,836.4) | 44,221.5 (26,371.6 to 65,620.3) | 108.8% (87.6% to 128.9%) | 345.1 (210.1 to 506.2) | 293.5 (176.2 to 434.1) | -15.0% (-22.6% to -7.4%) | 3,912,407.2 (3,446,680.0 to 4,461,719.3) | 6,458,434.4 (5,657,198.2 to 7,376,462.6) | 65.1% (52.1% to 79.1%) | 47,353.8 (42,378.1 to 52,772.5) | 42,333.3 (37,315.2 to 48,018.0) | -10.6% (-17.0% to -4.0%) | 4,258,911.6 (3,489,078.4 to 5,189,780.9) | 6,819,605.9 (5,638,910.3 to 8,187,611.1) | 60.1% (46.3% to 79.3%) | 43,603.8 (36,190.5 to 52,608.1) | 46,054.1 (37,840.4 to 55,876.9) | 5.6% (-3.1% to 17.6%) |
| Malaysia | Female | Caries of deciduous teeth | 384.8 (161.7 to 794.2) | 404.6 (172.8 to 784.3) | 5.1% (-13.3% to 24.3%) | 3.4 (1.4 to 7.0) | 3.2 (1.4 to 6.2) | -6.0% (-22.1% to 10.6%) | 1,008,824.7 (806,931.2 to 1,223,427.1) | 1,057,111.4 (845,823.8 to 1,293,853.8) | 4.8% (-11.5% to 19.9%) | 8,957.9 (7,152.0 to 10,888.0) | 8,392.7 (6,729.9 to 10,249.7) | -6.3% (-20.7% to 6.9%) | 1,879,819.5 (1,327,433.4 to 2,654,328.0) | 2,536,238.9 (1,774,407.3 to 3,647,977.3) | 34.9% (16.4% to 63.7%) | 16,832.0 (11,794.2 to 23,942.7) | 19,750.7 (13,973.4 to 27,849.9) | 17.3% (1.8% to 43.6%) |
| Malaysia | Female | Caries of permanent teeth | 2,207.8 (922.5 to 4,210.2) | 3,865.7 (1,650.5 to 7,652.1) | 75.1% (55.2% to 99.0%) | 26.4 (11.4 to 50.4) | 23.9 (10.3 to 46.9) | -9.6% (-18.9% to 0.4%) | 2,236,075.3 (1,765,221.2 to 2,789,357.6) | 3,929,438.0 (3,139,142.6 to 4,988,182.2) | 75.7% (56.3% to 99.5%) | 26,921.8 (21,394.6 to 32,962.5) | 24,305.2 (19,379.2 to 30,819.8) | -9.7% (-19.2% to 0.7%) | 2,282,471.6 (1,842,203.1 to 2,761,831.0) | 4,091,629.7 (3,250,821.8 to 4,865,161.3) | 79.3% (61.2% to 103.9%) | 25,271.5 (20,503.0 to 30,315.5) | 25,074.2 (19,922.1 to 29,994.7) | -0.8% (-10.8% to 11.7%) |
| Malaysia | Female | Periodontal diseases | 4,503.3 (1,795.2 to 9,850.4) | 7,791.5 (3,002.2 to 16,885.1) | 73.0% (13.9% to 161.8%) | 69.6 (27.6 to 150.6) | 49.8 (19.3 to 107.1) | -28.5% (-51.7% to 5.7%) | 691,521.3 (491,634.9 to 892,163.8) | 1,212,125.1 (902,336.0 to 1,595,646.3) | 75.3% (15.9% to 164.6%) | 10,744.9 (7,852.4 to 13,695.8) | 7,765.6 (5,812.8 to 10,188.1) | -27.7% (-51.1% to 6.0%) | 71,195.8 (52,179.5 to 87,305.3) | 129,404.8 (101,589.0 to 159,259.6) | 81.8% (31.4% to 154.9%) | 1,032.1 (804.2 to 1,237.4) | 821.7 (652.6 to 1,008.6) | -20.4% (-39.9% to 8.9%) |
| Malaysia | Female | Edentulism | 9,359.4 (6,048.8 to 13,280.5) | 23,011.6 (14,613.9 to 32,251.7) | 145.9% (132.4% to 162.6%) | 188.2 (120.3 to 264.2) | 159.2 (101.5 to 224.0) | -15.4% (-19.6% to -10.5%) | 340,664.7 (272,868.7 to 408,794.7) | 842,365.3 (667,253.7 to 1,023,161.5) | 147.3% (135.0% to 161.9%) | 6,908.6 (5,568.7 to 8,329.4) | 5,866.2 (4,666.3 to 7,080.2) | -15.1% (-18.7% to -10.4%) | 25,424.7 (20,748.5 to 30,076.9) | 62,332.6 (49,910.0 to 74,504.9) | 145.2% (130.1% to 162.6%) | 468.2 (382.5 to 555.6) | 407.5 (326.3 to 486.8) | -13.0% (-16.6% to -8.3%) |
| Malaysia | Female | Other oral disorders | 4,721.3 (2,889.2 to 6,973.1) | 9,148.1 (5,576.7 to 13,539.8) | 93.8% (84.1% to 102.2%) | 57.4 (35.3 to 84.7) | 57.4 (35.0 to 85.0) | 0.0% (-4.2% to 4.2%) | 162,726.5 (154,545.2 to 170,409.2) | 317,370.3 (302,301.1 to 332,543.1) | 95.0% (92.6% to 97.5%) | 1,993.8 (1,903.3 to 2,082.6) | 1,993.8 (1,903.3 to 2,082.6) | 0.0% (0.0% to 0.0%) | NA | NA | NA | NA | NA | NA |
| Malaysia | Male | Main Oral disorders | 17,057.8 (9,746.9 to 26,781.7) | 36,066.7 (21,397.5 to 54,907.2) | 111.4% (84.3% to 136.1%) | 279.2 (166.6 to 422.6) | 232.9 (139.9 to 351.1) | -16.6% (-25.5% to -8.1%) | 3,890,816.4 (3,437,220.8 to 4,448,677.2) | 6,706,370.1 (5,774,510.5 to 7,751,357.3) | 72.4% (57.8% to 88.1%) | 46,162.7 (41,308.4 to 52,002.1) | 41,222.1 (35,938.9 to 47,097.0) | -10.7% (-18.2% to -2.9%) | 4,360,505.4 (3,537,621.5 to 5,297,829.6) | 7,447,763.8 (6,193,885.5 to 8,776,998.8) | 70.8% (53.8% to 94.6%) | 43,322.8 (35,835.9 to 52,043.8) | 46,721.3 (38,442.5 to 55,461.6) | 7.8% (-1.9% to 21.1%) |
| Malaysia | Male | Caries of deciduous teeth | 406.0 (179.7 to 836.0) | 427.7 (179.5 to 864.2) | 5.3% (-15.1% to 24.4%) | 3.4 (1.5 to 7.0) | 3.2 (1.3 to 6.4) | -6.5% (-24.4% to 10.6%) | 1,063,797.6 (859,818.3 to 1,279,406.7) | 1,117,384.5 (874,891.7 to 1,367,886.0) | 5.0% (-14.3% to 21.6%) | 8,944.9 (7,221.4 to 10,776.9) | 8,342.6 (6,568.1 to 10,165.9) | -6.7% (-23.9% to 8.0%) | 1,959,461.6 (1,309,632.8 to 2,772,380.1) | 2,723,259.0 (1,936,764.8 to 3,819,703.6) | 39.0% (16.9% to 84.0%) | 16,648.6 (11,026.8 to 23,787.5) | 19,953.8 (14,330.6 to 27,336.0) | 19.9% (1.5% to 61.0%) |
| Malaysia | Male | Caries of permanent teeth | 2,204.6 (930.8 to 4,217.9) | 4,241.5 (1,822.8 to 8,084.5) | 92.4% (71.7% to 120.7%) | 26.3 (11.4 to 50.7) | 24.3 (10.5 to 46.2) | -7.5% (-17.1% to 4.3%) | 2,217,613.2 (1,764,265.3 to 2,776,358.6) | 4,279,288.1 (3,358,587.4 to 5,363,072.6) | 93.0% (72.7% to 120.6%) | 26,582.4 (21,410.2 to 32,394.8) | 24,555.1 (19,532.1 to 30,638.3) | -7.6% (-17.3% to 3.9%) | 2,311,916.1 (1,890,924.5 to 2,826,177.1) | 4,546,754.8 (3,748,574.1 to 5,391,265.1) | 96.7% (74.0% to 125.2%) | 25,276.3 (21,007.7 to 30,243.6) | 25,663.0 (21,032.6 to 30,574.3) | 1.5% (-9.9% to 14.8%) |
| Malaysia | Male | Periodontal diseases | 4,845.4 (1,934.6 to 10,583.5) | 8,283.3 (3,215.7 to 17,557.5) | 71.0% (13.4% to 162.5%) | 75.7 (30.1 to 162.5) | 51.0 (19.6 to 108.4) | -32.6% (-54.9% to 1.7%) | 737,365.4 (522,360.7 to 952,993.2) | 1,276,421.7 (944,778.1 to 1,706,169.4) | 73.1% (14.7% to 163.0%) | 11,585.2 (8,556.5 to 14,750.6) | 7,892.3 (5,903.2 to 10,408.3) | -31.9% (-54.4% to 2.1%) | 72,136.0 (52,396.9 to 89,754.2) | 132,305.6 (100,699.5 to 164,658.3) | 83.4% (30.8% to 164.1%) | 1,043.8 (809.2 to 1,251.7) | 799.5 (625.5 to 974.0) | -23.4% (-42.4% to 4.4%) |
| Malaysia | Male | Edentulism | 5,468.8 (3,479.7 to 7,692.6) | 14,589.3 (9,232.1 to 20,845.7) | 166.8% (149.7% to 183.1%) | 124.1 (79.0 to 175.8) | 104.7 (67.0 to 150.6) | -15.7% (-20.0% to -11.2%) | 198,452.2 (155,995.5 to 242,408.0) | 531,928.5 (416,560.5 to 658,155.6) | 168.0% (154.1% to 181.9%) | 4,552.8 (3,596.6 to 5,543.4) | 3,847.7 (3,003.2 to 4,776.0) | -15.5% (-19.6% to -11.9%) | 16,991.7 (13,526.2 to 20,584.6) | 45,444.4 (35,610.3 to 56,283.7) | 167.4% (152.2% to 184.1%) | 354.0 (282.4 to 430.0) | 304.9 (240.1 to 375.2) | -13.9% (-17.6% to -9.5%) |
| Malaysia | Male | Other oral disorders | 4,133.0 (2,529.6 to 6,127.7) | 8,525.0 (5,213.3 to 12,523.5) | 106.3% (96.6% to 117.8%) | 49.7 (30.6 to 73.7) | 49.7 (30.4 to 72.9) | 0.1% (-4.5% to 4.9%) | 141,232.3 (134,378.2 to 148,569.1) | 292,578.1 (278,833.2 to 306,754.9) | 107.2% (103.9% to 110.0%) | 1,710.3 (1,630.8 to 1,786.0) | 1,710.3 (1,630.8 to 1,786.0) | 0.0% (0.0% to 0.0%) | NA | NA | NA | NA | NA | NA |
| Myanmar | Both | Main Oral disorders | 66,029.2 (38,157.7 to 102,591.2) | 100,473.7 (59,242.6 to 154,225.8) | 52.2% (35.4% to 69.3%) | 220.2 (130.5 to 336.4) | 188.9 (112.2 to 288.6) | -14.2% (-23.5% to -4.5%) | 16,414,926.7 (14,434,677.0 to 18,511,263.0) | 21,411,174.9 (18,605,168.1 to 24,085,951.3) | 30.4% (21.7% to 39.1%) | 42,679.1 (37,662.5 to 47,678.5) | 38,342.9 (33,507.2 to 42,954.9) | -10.2% (-15.8% to -4.4%) | 24,419,717.4 (20,749,160.9 to 28,647,694.8) | 30,270,483.7 (25,828,668.4 to 35,262,817.4) | 24.0% (17.2% to 31.4%) | 54,643.6 (47,187.5 to 63,175.6) | 53,507.5 (45,560.3 to 62,426.3) | -2.1% (-7.9% to 3.0%) |
| Myanmar | Both | Caries of deciduous teeth | 1,505.9 (637.8 to 3,007.8) | 1,536.7 (657.8 to 3,032.7) | 2.0% (-18.3% to 26.4%) | 3.0 (1.3 to 5.9) | 2.9 (1.2 to 5.7) | -3.0% (-22.3% to 20.1%) | 3,971,698.6 (3,177,733.4 to 4,927,482.3) | 3,999,890.0 (3,136,298.1 to 5,105,193.0) | 0.7% (-5.3% to 6.7%) | 7,820.0 (6,260.3 to 9,688.1) | 7,485.9 (5,870.7 to 9,526.2) | -4.3% (-10.0% to 1.3%) | 10,408,808.5 (7,631,677.5 to 14,464,318.4) | 10,818,637.2 (7,715,251.6 to 14,794,773.0) | 3.9% (-3.3% to 10.0%) | 20,301.5 (14,906.1 to 27,976.5) | 20,024.0 (14,339.9 to 26,985.2) | -1.4% (-8.2% to 4.7%) |
| Myanmar | Both | Caries of permanent teeth | 10,248.7 (4,391.3 to 19,787.1) | 13,548.3 (5,823.4 to 26,663.4) | 32.2% (20.6% to 43.1%) | 26.5 (11.4 to 52.0) | 23.5 (10.2 to 46.2) | -11.4% (-18.0% to -4.8%) | 10,371,288.2 (8,458,878.8 to 12,749,055.0) | 13,717,331.4 (11,013,599.6 to 16,709,143.7) | 32.3% (20.9% to 43.1%) | 27,008.6 (22,323.8 to 32,581.5) | 23,852.6 (19,262.8 to 28,920.8) | -11.7% (-17.8% to -5.6%) | 13,714,211.3 (11,678,252.9 to 15,825,498.5) | 18,982,011.6 (16,203,214.2 to 21,702,764.9) | 38.4% (26.5% to 51.1%) | 33,310.9 (28,776.1 to 38,331.1) | 32,608.3 (27,817.2 to 37,380.1) | -2.1% (-11.1% to 6.2%) |
| Myanmar | Both | Periodontal diseases | 15,350.8 (5,852.1 to 33,978.5) | 23,417.1 (8,835.5 to 50,769.7) | 52.5% (-1.9% to 141.3%) | 54.2 (20.8 to 117.5) | 43.5 (16.6 to 94.6) | -19.9% (-47.1% to 25.3%) | 2,362,371.7 (1,655,496.1 to 3,125,152.4) | 3,646,236.1 (2,617,287.4 to 4,849,842.4) | 54.3% (-0.3% to 143.9%) | 8,397.6 (5,960.2 to 11,055.2) | 6,796.2 (5,007.6 to 8,901.9) | -19.1% (-46.6% to 25.5%) | 237,621.4 (171,911.8 to 305,132.0) | 372,688.8 (279,450.9 to 472,908.2) | 56.8% (8.0% to 132.5%) | 789.8 (577.1 to 1,002.6) | 683.0 (521.9 to 860.1) | -13.5% (-38.6% to 25.8%) |
| Myanmar | Both | Edentulism | 18,608.4 (11,736.1 to 26,400.8) | 31,277.7 (19,937.4 to 44,635.4) | 68.1% (59.7% to 76.4%) | 82.9 (53.5 to 117.3) | 65.2 (41.5 to 93.2) | -21.4% (-24.8% to -17.8%) | 678,660.3 (527,236.6 to 836,631.9) | 1,144,843.5 (887,973.2 to 1,415,808.9) | 68.7% (62.2% to 75.6%) | 3,065.6 (2,392.4 to 3,795.7) | 2,410.1 (1,900.4 to 2,986.3) | -21.4% (-24.5% to -18.4%) | 59,076.3 (46,532.6 to 73,950.6) | 97,146.0 (75,614.3 to 121,731.3) | 64.4% (56.7% to 72.4%) | 241.5 (192.5 to 303.4) | 192.1 (151.4 to 240.6) | -20.5% (-23.6% to -16.7%) |
| Myanmar | Both | Other oral disorders | 20,315.4 (12,371.6 to 30,501.8) | 30,693.9 (18,925.9 to 45,814.2) | 51.1% (46.1% to 56.8%) | 53.6 (32.7 to 80.4) | 53.8 (33.2 to 80.2) | 0.4% (-2.5% to 3.6%) | 699,356.0 (668,595.7 to 729,002.0) | 1,058,782.8 (1,012,481.9 to 1,100,590.3) | 51.4% (49.7% to 53.2%) | 1,856.0 (1,776.5 to 1,927.7) | 1,859.1 (1,778.6 to 1,930.7) | 0.2% (0.1% to 0.2%) | NA | NA | NA | NA | NA | NA |
| Myanmar | Female | Main Oral disorders | 36,623.2 (21,481.4 to 56,561.6) | 58,672.4 (34,644.6 to 89,256.3) | 60.2% (43.5% to 77.1%) | 236.3 (141.7 to 357.4) | 203.2 (120.6 to 306.9) | -14.0% (-22.8% to -4.9%) | 8,335,370.8 (7,335,259.8 to 9,458,693.3) | 11,233,695.0 (9,822,499.0 to 12,630,690.5) | 34.8% (24.1% to 44.5%) | 42,601.8 (37,699.5 to 47,567.8) | 38,601.3 (33,808.1 to 43,249.6) | -9.4% (-16.0% to -3.0%) | 12,291,234.6 (10,428,680.6 to 14,470,108.1) | 15,431,764.2 (13,062,490.0 to 17,950,882.1) | 25.6% (14.8% to 35.1%) | 54,677.3 (46,971.3 to 63,376.4) | 53,693.3 (45,315.6 to 62,657.4) | -1.8% (-10.0% to 5.6%) |
| Myanmar | Female | Caries of deciduous teeth | 757.3 (319.7 to 1,515.6) | 761.9 (324.4 to 1,508.8) | 0.6% (-20.4% to 28.2%) | 3.0 (1.3 to 6.0) | 2.9 (1.2 to 5.7) | -3.5% (-23.6% to 22.9%) | 1,995,383.1 (1,578,025.4 to 2,508,844.8) | 1,981,599.2 (1,554,313.6 to 2,523,520.2) | -0.7% (-8.4% to 5.3%) | 7,934.2 (6,267.0 to 9,961.9) | 7,560.8 (5,920.2 to 9,613.8) | -4.7% (-12.2% to 1.0%) | 5,118,120.0 (3,710,084.4 to 7,166,783.9) | 5,324,442.3 (3,802,945.7 to 7,339,963.2) | 4.0% (-4.6% to 13.9%) | 20,150.5 (14,631.0 to 28,027.1) | 20,068.0 (14,442.9 to 27,234.8) | -0.4% (-8.5% to 8.9%) |
| Myanmar | Female | Caries of permanent teeth | 5,131.8 (2,186.9 to 10,008.1) | 6,974.2 (2,974.7 to 13,409.4) | 35.9% (20.4% to 53.1%) | 26.0 (11.1 to 50.8) | 23.2 (9.9 to 44.7) | -10.6% (-19.7% to -0.5%) | 5,204,345.0 (4,185,526.8 to 6,423,246.6) | 7,094,730.9 (5,730,455.2 to 8,636,698.0) | 36.3% (20.7% to 52.2%) | 26,478.8 (21,756.6 to 32,242.7) | 23,637.1 (19,122.6 to 28,700.6) | -10.7% (-19.8% to -0.9%) | 7,015,140.7 (5,906,060.8 to 8,064,798.2) | 9,839,318.5 (8,260,688.2 to 11,371,137.2) | 40.3% (23.7% to 57.2%) | 33,469.8 (28,545.1 to 38,504.3) | 32,715.0 (27,483.6 to 38,011.1) | -2.3% (-14.6% to 9.1%) |
| Myanmar | Female | Periodontal diseases | 7,788.4 (3,023.7 to 17,054.9) | 12,934.1 (4,876.7 to 28,093.9) | 66.1% (7.2% to 160.7%) | 53.0 (20.2 to 115.7) | 43.8 (16.6 to 94.2) | -17.4% (-45.1% to 29.0%) | 1,202,437.0 (857,663.9 to 1,595,812.8) | 2,024,344.2 (1,484,667.7 to 2,680,512.0) | 68.4% (8.8% to 161.9%) | 8,226.5 (5,857.9 to 10,812.6) | 6,872.3 (5,096.6 to 9,011.2) | -16.5% (-44.9% to 29.2%) | 122,220.3 (88,493.8 to 155,382.5) | 206,189.2 (156,515.7 to 260,948.3) | 68.7% (16.9% to 151.2%) | 786.1 (580.1 to 992.4) | 694.4 (537.3 to 871.6) | -11.7% (-37.1% to 29.8%) |
| Myanmar | Female | Edentulism | 11,841.8 (7,517.6 to 16,889.8) | 20,880.9 (13,394.0 to 30,001.9) | 76.3% (65.2% to 88.3%) | 97.0 (62.7 to 138.0) | 75.8 (48.4 to 108.7) | -21.8% (-26.5% to -17.2%) | 432,210.3 (337,395.1 to 532,875.4) | 766,125.9 (596,091.4 to 948,824.0) | 77.3% (68.7% to 86.4%) | 3,579.0 (2,818.4 to 4,426.9) | 2,800.7 (2,204.9 to 3,484.3) | -21.7% (-25.8% to -17.7%) | 35,753.6 (28,219.5 to 44,412.8) | 61,814.1 (48,228.6 to 77,159.2) | 72.9% (63.4% to 83.1%) | 270.9 (214.8 to 336.0) | 215.8 (170.3 to 267.2) | -20.3% (-24.1% to -15.5%) |
| Myanmar | Female | Other oral disorders | 11,103.8 (6,748.1 to 16,599.2) | 17,121.3 (10,486.2 to 25,579.7) | 54.2% (47.1% to 62.0%) | 57.4 (35.0 to 85.6) | 57.4 (35.2 to 85.9) | 0.1% (-4.1% to 4.8%) | 383,565.8 (364,666.6 to 402,502.4) | 593,864.6 (566,277.6 to 620,942.8) | 54.8% (52.8% to 56.9%) | 1,993.8 (1,903.3 to 2,082.6) | 1,993.8 (1,903.3 to 2,082.6) | 0.0% (0.0% to 0.0%) | NA | NA | NA | NA | NA | NA |
| Myanmar | Male | Main Oral disorders | 29,406.0 (16,842.8 to 46,758.9) | 41,801.2 (24,256.1 to 65,295.3) | 42.2% (24.1% to 61.2%) | 202.4 (117.6 to 316.7) | 171.4 (101.0 to 267.0) | -15.3% (-25.6% to -4.3%) | 8,079,555.9 (7,058,318.0 to 9,269,397.1) | 10,177,479.9 (8,723,156.5 to 11,654,562.3) | 26.0% (16.5% to 36.7%) | 42,754.0 (37,659.1 to 48,092.8) | 38,043.1 (32,747.5 to 43,243.8) | -11.0% (-18.0% to -3.7%) | 12,128,482.8 (10,219,083.9 to 14,426,119.2) | 14,838,719.5 (12,564,823.0 to 17,334,732.3) | 22.3% (13.4% to 32.2%) | 54,596.1 (46,889.5 to 63,755.4) | 53,308.2 (45,217.9 to 62,170.1) | -2.4% (-9.4% to 5.3%) |
| Myanmar | Male | Caries of deciduous teeth | 748.6 (318.9 to 1,480.5) | 774.8 (326.3 to 1,547.2) | 3.5% (-19.0% to 29.4%) | 2.9 (1.2 to 5.8) | 2.8 (1.2 to 5.7) | -2.5% (-23.8% to 21.9%) | 1,976,315.5 (1,558,653.0 to 2,455,072.2) | 2,018,290.8 (1,551,991.4 to 2,592,766.4) | 2.1% (-5.2% to 11.2%) | 7,708.1 (6,079.2 to 9,559.6) | 7,413.9 (5,705.5 to 9,505.9) | -3.8% (-10.6% to 4.7%) | 5,290,688.5 (3,871,569.0 to 7,339,540.3) | 5,494,194.9 (3,881,707.3 to 7,395,182.2) | 3.8% (-6.5% to 12.0%) | 20,450.1 (14,967.7 to 28,235.1) | 19,982.2 (14,172.8 to 26,636.0) | -2.3% (-12.1% to 5.6%) |
| Myanmar | Male | Caries of permanent teeth | 5,116.8 (2,208.6 to 10,026.2) | 6,574.1 (2,813.7 to 12,967.5) | 28.5% (15.5% to 43.9%) | 27.1 (11.9 to 53.6) | 23.9 (10.4 to 46.7) | -12.1% (-22.5% to -2.4%) | 5,166,943.2 (4,137,985.2 to 6,320,915.0) | 6,622,600.5 (5,277,439.4 to 8,087,486.7) | 28.2% (15.6% to 43.1%) | 27,572.9 (22,460.6 to 33,278.3) | 24,109.0 (19,391.1 to 29,137.4) | -12.6% (-21.9% to -3.2%) | 6,699,070.6 (5,626,867.7 to 7,827,498.1) | 9,142,693.1 (7,738,698.4 to 10,525,770.7) | 36.5% (21.5% to 53.8%) | 33,142.4 (28,307.9 to 38,498.2) | 32,492.1 (27,456.9 to 37,445.4) | -2.0% (-12.4% to 11.2%) |
| Myanmar | Male | Periodontal diseases | 7,562.4 (2,844.8 to 16,746.3) | 10,483.0 (3,989.2 to 22,605.8) | 38.6% (-11.3% to 123.0%) | 55.6 (20.9 to 121.2) | 43.1 (16.4 to 94.5) | -22.4% (-49.0% to 23.2%) | 1,159,934.7 (800,782.3 to 1,548,080.8) | 1,621,891.9 (1,140,360.8 to 2,192,806.1) | 39.8% (-10.9% to 126.5%) | 8,587.5 (6,047.6 to 11,371.5) | 6,715.9 (4,894.3 to 8,931.6) | -21.8% (-48.9% to 23.5%) | 115,401.1 (82,280.4 to 149,928.5) | 166,499.6 (121,893.9 to 214,865.4) | 44.3% (-2.4% to 115.7%) | 793.5 (579.1 to 1,014.1) | 669.8 (511.0 to 853.5) | -15.6% (-40.4% to 24.6%) |
| Myanmar | Male | Edentulism | 6,766.6 (4,228.9 to 9,655.9) | 10,396.8 (6,578.1 to 14,936.7) | 53.6% (44.4% to 64.3%) | 67.2 (42.8 to 96.5) | 51.8 (33.1 to 74.0) | -22.9% (-27.2% to -18.3%) | 246,450.0 (188,025.3 to 307,570.5) | 378,717.5 (290,592.0 to 472,177.0) | 53.7% (46.0% to 61.2%) | 2,484.7 (1,921.4 to 3,112.4) | 1,911.1 (1,489.1 to 2,371.5) | -23.1% (-26.5% to -19.1%) | 23,322.7 (18,245.6 to 29,658.7) | 35,331.9 (27,436.9 to 44,518.3) | 51.5% (43.8% to 60.3%) | 210.1 (164.4 to 264.8) | 164.1 (128.0 to 206.9) | -21.9% (-25.7% to -17.7%) |
| Myanmar | Male | Other oral disorders | 9,211.6 (5,637.4 to 13,911.0) | 13,572.6 (8,289.3 to 20,443.0) | 47.3% (40.2% to 55.2%) | 49.6 (30.3 to 74.7) | 49.8 (30.5 to 74.7) | 0.4% (-4.0% to 5.3%) | 315,790.2 (300,145.6 to 331,760.2) | 464,918.2 (442,908.9 to 486,592.1) | 47.2% (45.4% to 48.9%) | 1,710.3 (1,630.8 to 1,786.0) | 1,710.3 (1,630.8 to 1,786.0) | 0.0% (0.0% to 0.0%) | NA | NA | NA | NA | NA | NA |
| Philippines | Both | Main Oral disorders | 128,884.1 (75,385.0 to 196,183.9) | 254,652.7 (159,979.4 to 368,582.0) | 97.6% (74.6% to 127.8%) | 318.3 (191.2 to 475.6) | 271.7 (169.6 to 389.3) | -14.6% (-24.3% to -0.9%) | 29,855,037.4 (27,114,981.7 to 32,821,865.3) | 50,843,182.7 (46,504,982.6 to 55,446,414.6) | 70.3% (64.4% to 77.5%) | 51,301.7 (47,112.6 to 55,678.4) | 46,324.0 (42,719.0 to 50,135.6) | -9.7% (-12.6% to -6.4%) | 40,614,838.2 (34,621,948.0 to 48,087,838.9) | 65,987,507.7 (58,491,520.8 to 74,786,770.8) | 62.5% (54.5% to 70.1%) | 56,939.5 (49,633.8 to 65,822.8) | 56,111.7 (49,910.2 to 63,482.2) | -1.5% (-4.6% to 1.4%) |
| Philippines | Both | Caries of deciduous teeth | 3,162.4 (1,357.0 to 6,214.8) | 3,885.5 (1,691.0 to 7,605.2) | 22.9% (17.2% to 30.1%) | 3.6 (1.5 to 7.1) | 3.4 (1.5 to 6.5) | -6.6% (-10.9% to -1.1%) | 8,299,080.9 (6,923,735.4 to 9,778,399.0) | 10,163,187.1 (8,608,915.4 to 11,912,003.6) | 22.5% (17.6% to 28.3%) | 9,417.5 (7,844.4 to 11,100.7) | 8,769.6 (7,422.6 to 10,260.8) | -6.9% (-10.6% to -2.2%) | 18,135,470.0 (13,330,254.2 to 25,981,081.7) | 23,789,791.6 (17,960,590.7 to 33,039,631.4) | 31.2% (23.5% to 40.1%) | 20,828.1 (15,224.6 to 30,002.7) | 20,172.2 (15,332.4 to 27,671.7) | -3.1% (-9.5% to 4.4%) |
| Philippines | Both | Caries of permanent teeth | 17,984.9 (7,944.8 to 34,737.0) | 35,137.9 (15,703.4 to 67,016.2) | 95.4% (84.6% to 107.1%) | 32.1 (14.4 to 61.4) | 31.4 (14.1 to 59.8) | -2.2% (-6.2% to 2.9%) | 18,206,235.5 (15,482,060.7 to 21,337,146.2) | 35,639,298.2 (31,347,767.2 to 40,659,804.0) | 95.8% (85.1% to 107.5%) | 32,748.7 (28,604.6 to 37,592.0) | 31,952.1 (28,304.0 to 36,132.1) | -2.4% (-6.1% to 2.6%) | 21,869,119.3 (19,432,792.0 to 24,400,337.3) | 41,405,638.4 (37,247,842.1 to 45,526,593.3) | 89.3% (83.2% to 95.9%) | 34,650.4 (30,935.0 to 38,343.4) | 35,100.1 (31,652.9 to 38,527.6) | 1.3% (-0.7% to 3.7%) |
| Philippines | Both | Periodontal diseases | 29,916.4 (11,754.3 to 64,662.1) | 24,637.3 (9,711.1 to 51,485.6) | -17.6% (-32.1% to 4.8%) | 72.2 (28.6 to 156.9) | 24.9 (9.7 to 51.1) | -65.6% (-71.5% to -56.7%) | 4,589,974.3 (3,313,681.8 to 5,883,197.6) | 3,805,439.0 (3,031,749.8 to 4,688,830.9) | -17.1% (-31.9% to 5.1%) | 11,147.5 (8,329.2 to 14,035.8) | 3,862.0 (3,137.8 to 4,734.7) | -65.4% (-71.4% to -56.6%) | 470,411.3 (352,131.6 to 570,173.9) | 418,410.3 (343,421.5 to 508,732.7) | -11.1% (-27.5% to 12.5%) | 1,044.2 (822.6 to 1,236.8) | 427.3 (349.7 to 520.0) | -59.1% (-66.0% to -49.2%) |
| Philippines | Both | Edentulism | 46,930.4 (30,170.6 to 65,248.9) | 130,460.4 (83,879.5 to 181,801.5) | 178.0% (133.7% to 243.1%) | 156.7 (100.0 to 218.6) | 158.3 (101.6 to 220.2) | 1.0% (-15.5% to 26.0%) | 1,708,813.8 (1,365,992.9 to 2,067,152.9) | 4,751,216.1 (4,043,931.0 to 5,527,617.9) | 178.0% (133.8% to 243.7%) | 5,777.3 (4,610.7 to 6,947.4) | 5,821.4 (5,002.3 to 6,802.0) | 0.8% (-15.6% to 25.9%) | 139,837.7 (114,098.7 to 167,038.8) | 373,667.5 (320,809.7 to 433,449.6) | 167.2% (128.0% to 227.4%) | 416.8 (341.9 to 497.5) | 412.1 (357.3 to 473.5) | -1.1% (-16.2% to 21.1%) |
| Philippines | Both | Other oral disorders | 30,890.0 (19,042.0 to 46,205.5) | 60,531.6 (37,352.6 to 90,562.2) | 96.0% (93.3% to 98.4%) | 53.7 (33.3 to 80.1) | 53.8 (33.4 to 80.4) | 0.2% (-0.5% to 0.8%) | 1,061,451.0 (1,013,310.5 to 1,112,373.6) | 2,084,297.7 (1,994,763.4 to 2,177,281.1) | 96.4% (94.2% to 98.4%) | 1,862.2 (1,784.7 to 1,946.1) | 1,861.5 (1,784.8 to 1,945.3) | 0.0% (-0.1% to 0.0%) | NA | NA | NA | NA | NA | NA |
| Philippines | Female | Main Oral disorders | 71,505.7 (42,701.1 to 107,797.1) | 150,039.5 (94,346.8 to 215,326.1) | 109.8% (85.4% to 142.4%) | 351.2 (212.5 to 518.2) | 308.7 (192.8 to 440.1) | -12.1% (-21.6% to 1.6%) | 14,922,233.6 (13,625,859.2 to 16,322,913.3) | 25,706,111.9 (23,541,205.2 to 27,916,039.3) | 72.3% (66.9% to 78.6%) | 51,684.4 (47,785.2 to 55,749.3) | 47,256.0 (43,646.3 to 51,051.0) | -8.6% (-11.2% to -5.7%) | 20,075,747.0 (17,141,065.2 to 23,678,538.7) | 32,201,958.4 (28,610,080.1 to 36,451,938.0) | 60.4% (52.3% to 68.6%) | 57,025.6 (49,670.4 to 66,011.7) | 56,070.5 (49,967.2 to 63,425.3) | -1.7% (-4.9% to 1.3%) |
| Philippines | Female | Caries of deciduous teeth | 1,538.4 (659.4 to 3,051.9) | 1,873.1 (820.4 to 3,679.2) | 21.8% (16.2% to 29.0%) | 3.6 (1.5 to 7.1) | 3.4 (1.5 to 6.6) | -6.5% (-10.8% to -0.9%) | 4,035,490.0 (3,370,365.5 to 4,752,968.7) | 4,899,623.3 (4,149,785.7 to 5,724,940.6) | 21.4% (16.6% to 27.3%) | 9,411.9 (7,844.8 to 11,100.8) | 8,775.0 (7,433.2 to 10,240.8) | -6.8% (-10.3% to -2.2%) | 8,853,368.1 (6,496,237.3 to 12,667,275.6) | 11,475,907.8 (8,666,210.9 to 15,952,356.3) | 29.6% (22.2% to 38.8%) | 20,845.5 (15,259.1 to 29,935.2) | 20,175.7 (15,358.9 to 27,679.5) | -3.2% (-9.5% to 4.6%) |
| Philippines | Female | Caries of permanent teeth | 8,975.0 (3,985.9 to 17,271.0) | 17,472.4 (7,802.6 to 33,256.7) | 94.7% (86.6% to 103.8%) | 32.0 (14.4 to 61.2) | 31.4 (14.1 to 59.7) | -1.8% (-4.7% to 1.5%) | 9,112,492.5 (7,806,721.9 to 10,603,708.5) | 17,810,967.6 (15,667,378.4 to 20,194,528.5) | 95.5% (87.8% to 104.4%) | 32,760.5 (28,782.7 to 37,328.8) | 32,145.1 (28,436.0 to 36,216.8) | -1.9% (-4.6% to 1.3%) | 10,902,180.1 (9,715,306.4 to 12,130,893.1) | 20,264,965.4 (18,232,969.9 to 22,285,026.8) | 85.9% (80.4% to 92.1%) | 34,664.1 (30,953.6 to 38,231.2) | 34,954.2 (31,489.1 to 38,358.4) | 0.8% (-1.1% to 3.1%) |
| Philippines | Female | Periodontal diseases | 14,583.1 (5,731.6 to 31,612.1) | 12,998.9 (5,095.2 to 26,863.3) | -10.9% (-28.1% to 15.4%) | 69.5 (27.6 to 151.5) | 25.8 (10.1 to 52.8) | -62.8% (-69.8% to -52.5%) | 2,245,115.4 (1,618,295.1 to 2,879,178.8) | 2,019,578.9 (1,618,890.2 to 2,481,930.2) | -10.0% (-27.6% to 16.4%) | 10,758.2 (7,976.2 to 13,563.0) | 4,030.8 (3,265.2 to 4,945.6) | -62.5% (-69.6% to -52.0%) | 235,460.2 (177,095.7 to 284,543.0) | 232,110.4 (189,957.7 to 284,709.5) | -1.4% (-20.6% to 26.4%) | 1,040.6 (822.7 to 1,230.4) | 465.4 (382.0 to 568.8) | -55.3% (-63.2% to -43.8%) |
| Philippines | Female | Edentulism | 29,883.8 (19,299.0 to 41,600.7) | 85,621.6 (55,325.3 to 119,114.5) | 186.5% (141.8% to 252.9%) | 188.5 (120.5 to 260.0) | 190.4 (122.8 to 264.6) | 1.0% (-15.3% to 25.5%) | 1,088,116.4 (866,413.0 to 1,321,282.5) | 3,127,930.2 (2,677,697.1 to 3,624,088.7) | 187.5% (143.7% to 255.0%) | 6,946.8 (5,556.8 to 8,371.2) | 7,004.1 (6,022.4 to 8,103.3) | 0.8% (-15.6% to 25.2%) | 84,738.6 (69,695.1 to 99,665.3) | 228,974.8 (198,139.0 to 261,713.0) | 170.2% (132.0% to 226.2%) | 475.4 (393.5 to 560.5) | 475.2 (414.6 to 538.5) | 0.0% (-14.0% to 21.3%) |
| Philippines | Female | Other oral disorders | 16,525.3 (10,228.1 to 24,487.5) | 32,073.5 (19,988.4 to 47,296.5) | 94.1% (91.1% to 97.0%) | 57.6 (35.8 to 84.9) | 57.7 (35.9 to 85.0) | 0.1% (-0.8% to 1.0%) | 569,711.3 (541,782.5 to 598,847.8) | 1,110,631.8 (1,058,812.8 to 1,166,506.0) | 94.9% (92.6% to 97.1%) | 2,003.2 (1,910.4 to 2,104.5) | 2,003.2 (1,910.4 to 2,104.5) | 0.0% (0.0% to 0.0%) | NA | NA | NA | NA | NA | NA |
| Philippines | Male | Main Oral disorders | 57,378.4 (32,740.4 to 89,862.1) | 104,613.2 (64,189.9 to 153,433.3) | 82.3% (60.0% to 109.9%) | 283.9 (169.1 to 427.0) | 231.0 (143.0 to 330.5) | -18.6% (-28.3% to -5.5%) | 14,932,803.8 (13,447,127.3 to 16,517,966.9) | 25,137,070.8 (22,929,288.5 to 27,468,345.1) | 68.3% (61.0% to 76.5%) | 50,905.9 (46,265.9 to 55,606.9) | 45,325.7 (41,647.5 to 49,170.7) | -11.0% (-14.7% to -6.9%) | 20,539,091.2 (17,470,626.4 to 24,309,495.1) | 33,785,549.3 (29,926,729.0 to 38,342,406.9) | 64.5% (56.4% to 72.8%) | 56,853.3 (49,500.4 to 65,590.6) | 56,146.7 (49,936.2 to 63,539.2) | -1.2% (-4.5% to 1.9%) |
| Philippines | Male | Caries of deciduous teeth | 1,624.0 (701.0 to 3,163.7) | 2,012.4 (876.9 to 3,926.0) | 23.9% (17.7% to 31.6%) | 3.6 (1.5 to 7.0) | 3.4 (1.5 to 6.5) | -6.6% (-11.8% to -0.7%) | 4,263,590.8 (3,548,488.5 to 5,026,823.7) | 5,263,563.8 (4,451,646.1 to 6,169,246.0) | 23.5% (17.8% to 29.9%) | 9,422.9 (7,828.0 to 11,119.3) | 8,764.6 (7,411.4 to 10,241.1) | -7.0% (-11.2% to -2.0%) | 9,282,101.9 (6,821,209.7 to 13,313,806.0) | 12,313,883.7 (9,285,594.4 to 17,087,275.1) | 32.7% (24.9% to 41.8%) | 20,811.6 (15,189.8 to 30,069.5) | 20,169.1 (15,320.7 to 27,665.2) | -3.1% (-9.5% to 4.8%) |
| Philippines | Male | Caries of permanent teeth | 9,009.9 (3,955.8 to 17,333.9) | 17,665.5 (7,918.0 to 33,659.1) | 96.1% (82.2% to 111.9%) | 32.2 (14.3 to 61.8) | 31.3 (14.1 to 59.8) | -2.6% (-7.9% to 4.0%) | 9,093,743.0 (7,680,367.2 to 10,784,872.9) | 17,828,330.6 (15,630,850.8 to 20,402,465.4) | 96.1% (82.2% to 112.0%) | 32,739.2 (28,376.9 to 37,855.7) | 31,765.4 (28,218.3 to 35,999.3) | -3.0% (-8.0% to 3.6%) | 10,966,939.1 (9,727,392.5 to 12,283,861.6) | 21,140,673.0 (19,024,203.3 to 23,208,738.6) | 92.8% (85.6% to 100.1%) | 34,637.0 (30,828.0 to 38,446.3) | 35,242.5 (31,777.3 to 38,649.6) | 1.7% (-0.8% to 4.6%) |
| Philippines | Male | Periodontal diseases | 15,333.2 (6,021.2 to 33,050.0) | 11,638.4 (4,593.4 to 24,641.7) | -24.1% (-36.2% to -5.8%) | 75.1 (29.8 to 162.5) | 23.9 (9.3 to 49.7) | -68.2% (-73.1% to -61.1%) | 2,344,858.9 (1,683,738.0 to 3,005,075.1) | 1,785,860.1 (1,423,568.5 to 2,196,055.8) | -23.8% (-36.1% to -6.0%) | 11,555.7 (8,660.4 to 14,531.2) | 3,687.0 (2,981.0 to 4,485.7) | -68.1% (-73.1% to -61.2%) | 234,951.1 (175,110.9 to 285,056.8) | 186,299.9 (153,712.6 to 223,384.2) | -20.7% (-34.4% to -1.4%) | 1,047.7 (821.9 to 1,243.3) | 384.9 (316.9 to 463.6) | -63.3% (-69.1% to -55.4%) |
| Philippines | Male | Edentulism | 17,046.6 (10,871.5 to 23,979.3) | 44,838.8 (28,469.5 to 63,159.7) | 163.0% (118.9% to 234.1%) | 123.3 (78.9 to 175.4) | 122.5 (77.9 to 169.3) | -0.7% (-17.9% to 26.4%) | 620,697.4 (489,379.2 to 753,010.3) | 1,623,285.9 (1,362,422.7 to 1,928,661.4) | 161.5% (117.3% to 231.2%) | 4,550.6 (3,604.7 to 5,571.6) | 4,490.4 (3,803.1 to 5,378.4) | -1.3% (-18.4% to 25.7%) | 55,099.1 (44,586.9 to 67,009.8) | 144,692.7 (123,386.0 to 172,497.8) | 162.6% (119.3% to 229.0%) | 357.0 (289.4 to 433.7) | 350.3 (302.1 to 412.6) | -1.9% (-18.4% to 21.8%) |
| Philippines | Male | Other oral disorders | 14,364.7 (8,828.2 to 21,843.6) | 28,458.1 (17,450.2 to 43,266.3) | 98.1% (95.3% to 101.1%) | 49.8 (30.6 to 75.1) | 50.0 (30.8 to 75.7) | 0.4% (-0.5% to 1.4%) | 491,739.7 (468,445.0 to 518,460.3) | 973,665.8 (931,231.5 to 1,022,953.8) | 98.0% (95.8% to 100.3%) | 1,720.2 (1,646.1 to 1,803.4) | 1,720.2 (1,646.1 to 1,803.4) | 0.0% (0.0% to 0.0%) | NA | NA | NA | NA | NA | NA |
| Singapore | Both | Main Oral disorders | 5,139.1 (2,919.8 to 8,193.7) | 13,974.8 (8,105.5 to 22,377.4) | 171.9% (134.4% to 212.2%) | 188.5 (110.8 to 294.0) | 183.9 (107.1 to 291.4) | -2.5% (-13.8% to 11.1%) | 1,015,498.8 (907,373.6 to 1,151,996.0) | 2,006,914.8 (1,771,781.2 to 2,249,548.4) | 97.6% (76.9% to 122.2%) | 34,361.8 (30,762.9 to 38,243.8) | 32,782.7 (29,185.0 to 36,602.3) | -4.6% (-12.8% to 4.0%) | 1,608,174.8 (1,395,961.3 to 1,837,268.4) | 2,521,016.0 (2,169,957.1 to 2,871,078.4) | 56.8% (41.6% to 72.0%) | 53,893.2 (45,800.8 to 62,360.9) | 53,564.3 (45,764.0 to 61,544.1) | -0.6% (-6.9% to 5.6%) |
| Singapore | Both | Caries of deciduous teeth | 60.0 (25.5 to 117.6) | 72.3 (31.0 to 141.7) | 20.6% (6.6% to 36.4%) | 2.8 (1.2 to 5.6) | 2.4 (1.0 to 4.8) | -14.2% (-25.3% to -3.3%) | 156,666.2 (123,640.2 to 192,482.1) | 188,461.0 (144,367.5 to 234,966.7) | 20.3% (8.1% to 32.9%) | 7,432.8 (5,899.7 to 9,075.9) | 6,362.6 (4,858.6 to 7,938.0) | -14.4% (-24.2% to -5.7%) | 447,643.2 (325,924.9 to 599,796.5) | 596,509.9 (439,443.7 to 773,549.5) | 33.3% (21.7% to 43.2%) | 20,236.3 (14,972.0 to 26,407.2) | 20,211.6 (14,779.2 to 26,394.7) | -0.1% (-6.8% to 6.7%) |
| Singapore | Both | Caries of permanent teeth | 593.6 (257.1 to 1,143.3) | 967.3 (421.7 to 1,880.1) | 62.9% (45.4% to 84.7%) | 17.2 (7.5 to 33.1) | 16.3 (7.1 to 32.0) | -5.0% (-13.1% to 3.5%) | 597,860.9 (493,161.4 to 730,126.3) | 978,491.2 (786,994.4 to 1,177,574.4) | 63.7% (47.2% to 85.3%) | 17,355.7 (14,473.3 to 20,916.3) | 16,443.7 (13,339.3 to 19,902.2) | -5.3% (-12.7% to 2.6%) | 1,131,972.1 (980,691.9 to 1,291,699.2) | 1,840,224.6 (1,541,725.7 to 2,143,658.3) | 62.6% (43.2% to 84.1%) | 32,658.0 (27,963.4 to 37,575.2) | 32,333.9 (27,692.7 to 37,440.2) | -1.0% (-11.4% to 7.8%) |
| Singapore | Both | Periodontal diseases | 1,601.8 (628.6 to 3,502.0) | 5,121.8 (1,985.4 to 10,313.6) | 219.8% (114.5% to 376.2%) | 56.8 (22.1 to 124.2) | 60.0 (23.3 to 120.7) | 5.6% (-27.7% to 56.2%) | 244,776.6 (177,593.7 to 316,425.8) | 788,837.3 (594,423.4 to 1,008,013.8) | 222.3% (116.0% to 377.8%) | 8,725.2 (6,419.8 to 11,186.5) | 9,233.6 (7,023.5 to 11,807.3) | 5.8% (-27.6% to 55.3%) | 24,234.1 (18,044.1 to 30,244.5) | 69,150.3 (54,549.1 to 84,735.0) | 185.3% (106.1% to 304.1%) | 802.2 (609.6 to 981.5) | 844.1 (669.0 to 1,034.3) | 5.2% (-22.0% to 44.4%) |
| Singapore | Both | Edentulism | 1,165.4 (756.2 to 1,703.8) | 4,344.7 (2,811.7 to 6,381.8) | 272.8% (253.5% to 294.5%) | 57.9 (37.9 to 84.4) | 51.4 (33.4 to 75.6) | -11.3% (-14.9% to -7.1%) | 42,508.9 (33,155.9 to 52,868.1) | 158,761.5 (123,844.7 to 199,054.5) | 273.5% (255.8% to 290.9%) | 2,129.8 (1,693.6 to 2,635.1) | 1,881.0 (1,473.9 to 2,340.8) | -11.7% (-14.9% to -7.9%) | 4,325.5 (3,435.8 to 5,339.5) | 15,131.1 (11,752.4 to 19,034.6) | 249.8% (232.9% to 267.5%) | 196.7 (155.7 to 246.3) | 174.7 (136.2 to 221.5) | -11.2% (-14.0% to -7.5%) |
| Singapore | Both | Other oral disorders | 1,718.3 (1,049.9 to 2,557.6) | 3,468.7 (2,131.0 to 5,152.9) | 101.9% (94.8% to 110.2%) | 53.7 (32.9 to 79.7) | 53.7 (32.8 to 80.1) | -0.1% (-2.8% to 3.3%) | 58,965.9 (56,169.4 to 61,618.6) | 119,821.3 (113,757.1 to 125,770.2) | 103.2% (99.2% to 107.5%) | 1,848.7 (1,765.8 to 1,929.3) | 1,843.9 (1,762.3 to 1,924.5) | -0.3% (-0.4% to -0.1%) | NA | NA | NA | NA | NA | NA |
| Singapore | Female | Main Oral disorders | 2,507.8 (1,441.7 to 3,958.6) | 6,531.0 (3,817.8 to 10,310.1) | 160.4% (125.1% to 196.7%) | 177.7 (104.2 to 276.9) | 174.3 (102.3 to 275.8) | -1.9% (-13.5% to 11.1%) | 490,227.9 (431,939.1 to 557,180.4) | 938,880.7 (826,745.9 to 1,065,741.2) | 91.5% (69.9% to 115.2%) | 33,117.8 (29,597.2 to 37,234.3) | 31,594.3 (28,122.3 to 35,649.4) | -4.6% (-13.0% to 4.0%) | 789,820.5 (674,266.7 to 902,706.4) | 1,235,414.7 (1,055,903.2 to 1,417,089.1) | 56.4% (42.4% to 73.3%) | 53,532.8 (45,525.0 to 62,282.5) | 53,469.9 (45,362.0 to 61,949.0) | -0.1% (-6.5% to 7.1%) |
| Singapore | Female | Caries of deciduous teeth | 29.6 (12.4 to 58.8) | 35.4 (15.2 to 69.9) | 19.6% (2.4% to 37.2%) | 2.8 (1.2 to 5.6) | 2.4 (1.0 to 4.7) | -14.9% (-27.5% to -2.1%) | 77,221.5 (60,585.4 to 96,012.0) | 92,154.0 (70,008.8 to 117,034.2) | 19.3% (5.6% to 30.9%) | 7,387.3 (5,821.4 to 9,137.1) | 6,271.3 (4,767.5 to 7,981.4) | -15.1% (-25.3% to -5.9%) | 220,597.5 (158,109.9 to 289,855.9) | 296,245.0 (218,064.3 to 383,694.5) | 34.3% (22.7% to 47.9%) | 20,041.7 (14,762.6 to 26,293.0) | 20,178.9 (14,782.5 to 26,293.7) | 0.7% (-7.1% to 10.4%) |
| Singapore | Female | Caries of permanent teeth | 293.5 (121.6 to 562.7) | 473.6 (205.2 to 931.2) | 61.4% (42.7% to 85.3%) | 17.2 (7.3 to 32.5) | 16.3 (7.0 to 31.5) | -5.2% (-15.5% to 5.9%) | 296,219.6 (234,647.5 to 361,899.4) | 480,820.7 (388,620.2 to 588,729.4) | 62.3% (43.9% to 87.0%) | 17,395.7 (14,170.6 to 21,034.8) | 16,481.2 (13,399.5 to 20,108.4) | -5.3% (-14.7% to 5.9%) | 556,922.0 (475,012.5 to 640,314.5) | 902,354.0 (754,699.7 to 1,085,427.7) | 62.0% (42.8% to 83.7%) | 32,630.2 (27,784.0 to 37,814.6) | 32,392.6 (27,411.6 to 38,331.2) | -0.7% (-10.1% to 10.5%) |
| Singapore | Female | Periodontal diseases | 659.3 (257.1 to 1,445.8) | 2,094.2 (806.9 to 4,315.7) | 217.6% (102.4% to 404.3%) | 46.1 (18.0 to 100.3) | 50.3 (19.5 to 104.4) | 8.9% (-28.6% to 72.1%) | 101,106.0 (71,346.9 to 134,368.4) | 323,888.6 (239,805.5 to 422,983.2) | 220.3% (104.8% to 402.5%) | 7,103.1 (5,037.0 to 9,597.8) | 7,761.1 (5,703.5 to 10,097.2) | 9.3% (-28.2% to 70.4%) | 10,129.2 (7,247.3 to 13,053.2) | 29,520.4 (23,091.1 to 36,607.5) | 191.4% (102.2% to 331.0%) | 673.6 (494.4 to 860.5) | 732.9 (570.1 to 912.2) | 8.8% (-23.3% to 59.9%) |
| Singapore | Female | Edentulism | 571.1 (371.4 to 833.0) | 2,042.2 (1,337.6 to 3,006.4) | 257.6% (232.0% to 285.8%) | 52.1 (33.8 to 74.9) | 45.8 (30.0 to 67.3) | -12.1% (-17.8% to -6.1%) | 20,928.2 (16,687.7 to 26,079.2) | 75,171.6 (59,269.9 to 93,724.3) | 259.2% (236.4% to 284.5%) | 1,921.3 (1,517.3 to 2,385.3) | 1,684.8 (1,330.8 to 2,096.3) | -12.3% (-17.4% to -7.2%) | 2,171.8 (1,709.9 to 2,705.7) | 7,295.2 (5,576.6 to 9,317.8) | 235.9% (215.7% to 262.3%) | 187.3 (145.7 to 235.8) | 165.6 (126.6 to 212.3) | -11.6% (-16.1% to -5.3%) |
| Singapore | Female | Other oral disorders | 954.2 (583.4 to 1,416.7) | 1,885.6 (1,154.8 to 2,773.5) | 97.6% (88.4% to 107.0%) | 59.4 (36.6 to 88.0) | 59.5 (36.4 to 88.6) | 0.2% (-3.8% to 4.5%) | 32,854.7 (31,160.3 to 34,516.9) | 65,273.6 (61,649.8 to 68,491.2) | 98.7% (94.8% to 102.9%) | 2,047.7 (1,940.4 to 2,139.5) | 2,047.7 (1,940.4 to 2,139.5) | 0.0% (0.0% to 0.0%) | NA | NA | NA | NA | NA | NA |
| Singapore | Male | Main Oral disorders | 2,631.2 (1,488.7 to 4,217.7) | 7,443.9 (4,247.4 to 12,094.4) | 182.9% (141.2% to 228.3%) | 199.8 (116.9 to 314.0) | 192.0 (110.8 to 305.9) | -3.9% (-15.4% to 9.8%) | 525,270.8 (466,149.5 to 598,494.8) | 1,068,034.1 (940,011.0 to 1,201,385.7) | 103.3% (79.5% to 131.8%) | 35,687.0 (32,204.3 to 39,805.4) | 33,873.7 (30,039.2 to 37,775.5) | -5.1% (-13.6% to 4.8%) | 818,354.3 (706,099.1 to 933,677.0) | 1,285,601.3 (1,093,233.5 to 1,486,655.0) | 57.1% (33.2% to 79.1%) | 54,269.6 (46,307.6 to 62,799.4) | 53,641.2 (45,863.9 to 61,765.2) | -1.2% (-11.5% to 7.7%) |
| Singapore | Male | Caries of deciduous teeth | 30.4 (13.2 to 59.8) | 36.9 (15.8 to 73.0) | 21.6% (2.1% to 41.7%) | 2.9 (1.2 to 5.6) | 2.5 (1.1 to 4.9) | -13.4% (-27.3% to 1.3%) | 79,444.7 (62,823.2 to 97,471.7) | 96,307.0 (73,700.2 to 119,634.1) | 21.2% (6.7% to 38.2%) | 7,472.5 (5,951.3 to 9,078.3) | 6,451.5 (4,926.3 to 8,020.0) | -13.7% (-25.0% to -1.0%) | 227,045.7 (164,725.9 to 315,154.1) | 300,264.9 (218,355.3 to 380,630.9) | 32.2% (17.3% to 47.0%) | 20,425.2 (15,053.0 to 26,864.2) | 20,242.1 (14,614.9 to 26,352.2) | -0.9% (-9.5% to 8.0%) |
| Singapore | Male | Caries of permanent teeth | 300.1 (131.5 to 579.9) | 493.7 (214.3 to 950.1) | 64.5% (38.0% to 93.3%) | 17.2 (7.5 to 33.3) | 16.3 (7.1 to 32.0) | -4.9% (-16.2% to 8.2%) | 301,641.3 (243,531.2 to 370,125.9) | 497,670.5 (390,531.9 to 604,483.9) | 65.0% (38.6% to 93.9%) | 17,325.1 (14,115.4 to 21,014.7) | 16,404.9 (13,305.4 to 19,851.0) | -5.3% (-15.5% to 8.0%) | 575,050.1 (490,279.5 to 658,690.9) | 937,870.6 (761,618.2 to 1,110,694.6) | 63.1% (30.9% to 94.6%) | 32,706.3 (27,523.3 to 37,782.6) | 32,270.5 (27,232.4 to 37,469.4) | -1.3% (-16.3% to 12.8%) |
| Singapore | Male | Periodontal diseases | 942.4 (372.2 to 2,054.0) | 3,027.6 (1,169.2 to 6,072.8) | 221.3% (120.5% to 369.4%) | 68.2 (26.7 to 146.6) | 69.1 (26.9 to 137.9) | 1.3% (-28.9% to 45.3%) | 143,670.6 (105,019.6 to 186,274.0) | 464,948.7 (353,660.5 to 591,458.7) | 223.6% (121.4% to 367.7%) | 10,462.7 (7,929.4 to 13,319.2) | 10,615.1 (8,164.3 to 13,499.6) | 1.5% (-28.9% to 43.9%) | 14,104.8 (10,659.8 to 17,485.4) | 39,630.0 (31,237.0 to 48,434.2) | 181.0% (107.5% to 292.1%) | 933.8 (727.9 to 1,120.2) | 948.5 (763.8 to 1,158.7) | 1.6% (-23.2% to 38.2%) |
| Singapore | Male | Edentulism | 594.2 (374.9 to 863.6) | 2,302.5 (1,455.2 to 3,333.6) | 287.5% (261.8% to 314.2%) | 63.5 (40.8 to 91.8) | 56.0 (35.6 to 81.1) | -11.9% (-16.9% to -6.4%) | 21,580.7 (16,660.3 to 27,427.7) | 83,589.9 (63,850.5 to 106,313.6) | 287.3% (265.4% to 309.9%) | 2,332.2 (1,809.4 to 2,933.3) | 2,042.3 (1,560.6 to 2,558.3) | -12.4% (-16.7% to -7.8%) | 2,153.7 (1,683.2 to 2,713.0) | 7,835.9 (6,096.7 to 9,858.1) | 263.8% (241.2% to 291.8%) | 204.3 (160.5 to 255.1) | 180.2 (142.1 to 228.4) | -11.8% (-16.1% to -7.1%) |
| Singapore | Male | Other oral disorders | 764.1 (464.5 to 1,141.1) | 1,583.1 (973.2 to 2,369.9) | 107.2% (95.5% to 119.4%) | 48.0 (29.1 to 71.6) | 48.1 (29.4 to 72.5) | 0.2% (-4.8% to 5.1%) | 26,111.2 (24,779.6 to 27,450.8) | 54,547.7 (51,691.1 to 57,400.9) | 108.9% (103.9% to 114.2%) | 1,648.1 (1,571.3 to 1,728.3) | 1,648.1 (1,571.3 to 1,728.3) | 0.0% (0.0% to 0.0%) | NA | NA | NA | NA | NA | NA |
| Thailand | Both | Main Oral disorders | 120,129.3 (69,350.6 to 187,892.6) | 239,349.3 (140,105.0 to 370,447.4) | 99.2% (75.8% to 122.1%) | 265.1 (157.7 to 410.0) | 256.9 (150.7 to 400.8) | -3.1% (-12.3% to 7.5%) | 27,823,970.4 (24,807,271.0 to 31,505,181.8) | 35,300,379.9 (31,786,635.1 to 38,729,499.8) | 26.9% (16.4% to 37.8%) | 50,841.3 (45,912.0 to 56,440.6) | 48,745.5 (43,826.0 to 54,191.4) | -4.1% (-9.8% to 1.3%) | 31,336,906.6 (26,498,955.5 to 37,890,447.6) | 30,350,531.1 (26,443,445.5 to 35,235,626.4) | -3.1% (-12.2% to 6.3%) | 52,671.0 (44,760.5 to 63,046.3) | 54,319.1 (46,270.1 to 64,131.2) | 3.1% (-4.4% to 10.7%) |
| Thailand | Both | Caries of deciduous teeth | 2,178.7 (947.0 to 4,397.6) | 1,108.3 (462.5 to 2,239.4) | -49.1% (-54.4% to -43.4%) | 3.9 (1.7 to 7.8) | 3.4 (1.4 to 6.9) | -11.5% (-20.0% to -2.0%) | 5,708,922.9 (4,798,165.4 to 6,612,873.9) | 2,901,976.1 (2,412,039.9 to 3,380,413.5) | -49.2% (-53.5% to -44.2%) | 10,138.7 (8,586.6 to 11,690.2) | 8,966.4 (7,518.3 to 10,381.1) | -11.6% (-18.4% to -3.2%) | 10,838,104.2 (7,461,729.2 to 16,496,364.6) | 6,490,613.6 (4,533,902.3 to 9,967,761.8) | -40.1% (-47.0% to -30.2%) | 18,631.0 (13,220.5 to 27,533.7) | 19,240.9 (13,765.9 to 28,213.7) | 3.3% (-8.7% to 20.3%) |
| Thailand | Both | Caries of permanent teeth | 18,516.9 (7,979.1 to 34,994.1) | 22,570.4 (9,932.7 to 45,221.0) | 21.9% (6.5% to 39.5%) | 32.3 (14.0 to 60.6) | 30.3 (13.2 to 59.8) | -6.2% (-13.5% to 1.1%) | 18,721,483.3 (15,446,916.6 to 22,591,493.0) | 23,081,133.4 (19,118,337.4 to 27,176,226.9) | 23.3% (7.9% to 40.5%) | 32,805.7 (27,452.1 to 38,680.7) | 30,733.8 (25,497.4 to 36,682.9) | -6.3% (-13.4% to 1.4%) | 19,916,003.3 (16,829,561.6 to 22,901,851.8) | 22,612,897.2 (19,570,840.2 to 26,101,296.8) | 13.5% (2.3% to 27.9%) | 32,790.5 (27,888.8 to 37,598.7) | 33,761.0 (29,048.4 to 39,156.1) | 3.0% (-4.9% to 12.7%) |
| Thailand | Both | Periodontal diseases | 30,798.4 (12,153.7 to 64,871.8) | 76,313.3 (30,305.3 to 156,340.9) | 147.8% (76.2% to 235.2%) | 66.4 (26.6 to 138.9) | 77.3 (30.5 to 159.3) | 16.4% (-15.9% to 61.6%) | 4,726,488.1 (3,708,218.1 to 5,867,491.8) | 11,829,054.2 (9,466,031.6 to 14,514,573.3) | 150.3% (77.7% to 236.0%) | 10,253.9 (8,197.8 to 12,740.0) | 11,947.8 (9,457.2 to 15,050.1) | 16.5% (-16.3% to 60.8%) | 465,315.2 (367,969.3 to 551,916.3) | 966,442.3 (833,868.7 to 1,104,786.4) | 107.7% (64.2% to 169.9%) | 946.6 (766.2 to 1,110.5) | 1,057.6 (901.5 to 1,268.2) | 11.7% (-11.5% to 44.2%) |
| Thailand | Both | Edentulism | 38,467.8 (24,519.9 to 54,226.3) | 99,441.4 (63,377.4 to 143,276.0) | 158.5% (143.0% to 174.0%) | 108.8 (69.1 to 154.4) | 92.1 (59.1 to 131.7) | -15.4% (-18.9% to -11.5%) | 1,399,217.0 (1,101,339.2 to 1,704,380.1) | 3,652,143.1 (2,841,593.0 to 4,497,142.3) | 161.0% (145.7% to 175.0%) | 4,003.5 (3,179.4 to 4,898.4) | 3,380.8 (2,645.8 to 4,149.2) | -15.6% (-18.7% to -12.0%) | 117,483.8 (94,036.6 to 144,800.0) | 280,578.0 (219,905.2 to 351,018.5) | 138.8% (122.9% to 154.3%) | 303.0 (244.6 to 370.6) | 259.6 (206.3 to 321.4) | -14.3% (-17.9% to -10.5%) |
| Thailand | Both | Other oral disorders | 30,167.5 (18,265.1 to 45,487.3) | 39,916.0 (24,753.8 to 59,189.0) | 32.3% (27.0% to 38.1%) | 53.7 (32.7 to 80.9) | 53.8 (33.0 to 80.3) | 0.3% (-2.7% to 4.0%) | 1,036,624.2 (991,549.0 to 1,079,209.0) | 1,388,099.1 (1,329,464.7 to 1,447,217.2) | 33.9% (30.9% to 37.2%) | 1,855.1 (1,775.3 to 1,926.7) | 1,856.0 (1,776.1 to 1,927.5) | 0.0% (0.0% to 0.1%) | NA | NA | NA | NA | NA | NA |
| Thailand | Female | Main Oral disorders | 66,854.0 (39,270.9 to 102,919.8) | 137,391.6 (80,285.8 to 209,122.5) | 105.5% (83.6% to 127.7%) | 285.7 (171.0 to 435.9) | 275.1 (163.0 to 424.0) | -3.7% (-12.0% to 5.8%) | 14,106,049.2 (12,546,304.0 to 15,937,408.0) | 18,441,700.1 (16,597,312.3 to 20,114,189.8) | 30.7% (19.4% to 44.0%) | 50,960.0 (45,946.3 to 56,495.1) | 48,902.3 (44,038.8 to 54,200.1) | -4.0% (-10.4% to 3.1%) | 15,580,091.6 (13,224,184.3 to 18,655,915.7) | 15,472,711.5 (13,342,839.7 to 17,982,422.5) | -0.7% (-11.7% to 11.3%) | 52,428.5 (44,552.8 to 62,560.0) | 54,388.9 (46,209.1 to 64,000.7) | 3.7% (-5.6% to 12.9%) |
| Thailand | Female | Caries of deciduous teeth | 1,074.1 (463.2 to 2,163.6) | 538.4 (229.3 to 1,111.5) | -49.9% (-55.5% to -42.0%) | 3.9 (1.7 to 7.8) | 3.4 (1.5 to 7.0) | -11.8% (-21.6% to 1.7%) | 2,813,880.3 (2,374,596.9 to 3,281,020.7) | 1,409,113.7 (1,171,167.1 to 1,640,041.0) | -49.9% (-54.4% to -44.4%) | 10,167.8 (8,673.1 to 11,788.5) | 8,957.5 (7,530.4 to 10,355.9) | -11.9% (-19.2% to -2.8%) | 5,298,820.6 (3,554,424.1 to 8,124,758.2) | 3,158,623.4 (2,185,669.3 to 4,841,941.2) | -40.4% (-49.5% to -29.3%) | 18,553.8 (12,803.1 to 27,627.3) | 19,253.4 (13,679.1 to 28,165.8) | 3.8% (-11.5% to 23.5%) |
| Thailand | Female | Caries of permanent teeth | 9,381.4 (4,025.2 to 17,954.1) | 11,734.6 (5,164.5 to 23,143.0) | 25.1% (8.8% to 48.0%) | 32.2 (14.0 to 61.6) | 30.3 (13.2 to 59.9) | -5.9% (-16.1% to 5.2%) | 9,510,348.6 (7,738,246.1 to 11,484,727.8) | 12,050,954.0 (9,967,680.0 to 14,119,820.2) | 26.7% (11.1% to 48.7%) | 32,853.1 (26,970.1 to 39,056.1) | 30,884.8 (25,661.4 to 36,611.7) | -6.0% (-15.4% to 4.7%) | 9,979,632.6 (8,491,946.3 to 11,514,193.1) | 11,643,880.0 (9,894,890.4 to 13,567,360.2) | 16.7% (1.5% to 30.6%) | 32,617.8 (27,726.2 to 37,577.3) | 33,806.8 (28,790.7 to 39,654.5) | 3.6% (-7.3% to 14.8%) |
| Thailand | Female | Periodontal diseases | 14,794.9 (5,899.7 to 30,851.7) | 38,296.2 (15,095.7 to 80,602.3) | 158.8% (84.8% to 253.9%) | 61.7 (24.6 to 129.3) | 73.4 (29.2 to 156.7) | 18.9% (-16.0% to 64.0%) | 2,277,530.1 (1,758,855.9 to 2,802,141.5) | 5,961,482.3 (4,741,395.1 to 7,314,179.1) | 161.8% (86.8% to 253.1%) | 9,552.3 (7,493.6 to 11,682.3) | 11,382.8 (8,873.3 to 14,356.5) | 19.2% (-16.3% to 62.6%) | 228,977.4 (178,948.7 to 274,183.5) | 499,028.7 (426,369.3 to 573,003.8) | 117.9% (70.3% to 182.4%) | 910.2 (727.6 to 1,077.8) | 1,031.2 (868.6 to 1,235.7) | 13.3% (-11.3% to 49.3%) |
| Thailand | Female | Edentulism | 25,177.7 (15,857.7 to 35,253.2) | 64,637.3 (41,342.1 to 92,791.0) | 156.7% (140.6% to 175.1%) | 130.3 (82.1 to 184.1) | 110.3 (70.5 to 157.5) | -15.3% (-19.7% to -10.4%) | 916,275.6 (723,193.5 to 1,114,917.0) | 2,375,576.3 (1,858,093.6 to 2,930,393.5) | 159.3% (143.1% to 175.4%) | 4,782.7 (3,782.9 to 5,839.2) | 4,043.9 (3,179.6 to 4,966.4) | -15.4% (-19.6% to -11.1%) | 72,660.9 (58,363.6 to 88,456.8) | 171,179.4 (135,759.9 to 212,545.0) | 135.6% (117.9% to 151.6%) | 346.7 (280.8 to 420.7) | 297.4 (237.9 to 366.2) | -14.2% (-18.5% to -10.2%) |
| Thailand | Female | Other oral disorders | 16,425.9 (10,019.9 to 24,714.3) | 22,185.2 (13,650.9 to 32,979.9) | 35.1% (29.0% to 41.8%) | 57.6 (35.2 to 86.3) | 57.7 (35.3 to 86.2) | 0.0% (-4.1% to 4.2%) | 565,213.1 (537,139.9 to 592,746.7) | 774,209.7 (738,234.3 to 810,449.5) | 37.0% (33.6% to 40.8%) | 1,993.8 (1,903.3 to 2,082.6) | 1,993.8 (1,903.3 to 2,082.6) | 0.0% (0.0% to 0.0%) | NA | NA | NA | NA | NA | NA |
| Thailand | Male | Main Oral disorders | 53,275.3 (30,268.4 to 85,426.2) | 101,957.7 (58,736.3 to 160,891.1) | 91.4% (66.9% to 116.1%) | 241.8 (142.6 to 377.7) | 236.2 (136.0 to 373.0) | -2.3% (-12.3% to 9.5%) | 13,717,921.1 (12,200,400.6 to 15,581,546.8) | 16,858,679.8 (15,139,609.0 to 18,689,886.0) | 22.9% (12.4% to 35.0%) | 50,705.7 (45,724.2 to 56,497.6) | 48,570.9 (43,541.1 to 54,365.7) | -4.2% (-10.2% to 2.2%) | 15,756,815.0 (13,111,321.7 to 19,223,004.0) | 14,877,819.6 (12,943,694.9 to 17,267,237.1) | -5.6% (-15.1% to 5.4%) | 52,913.6 (44,257.5 to 63,704.3) | 54,254.5 (45,755.3 to 64,383.0) | 2.5% (-4.6% to 11.8%) |
| Thailand | Male | Caries of deciduous teeth | 1,104.6 (478.7 to 2,213.8) | 569.9 (239.9 to 1,124.6) | -48.4% (-55.5% to -41.3%) | 3.9 (1.7 to 7.7) | 3.4 (1.4 to 6.8) | -11.2% (-22.5% to 0.5%) | 2,895,042.6 (2,417,708.6 to 3,374,470.1) | 1,492,862.3 (1,229,709.5 to 1,755,934.4) | -48.4% (-54.1% to -43.4%) | 10,110.6 (8,517.1 to 11,682.8) | 8,974.8 (7,481.8 to 10,454.2) | -11.2% (-20.4% to -2.7%) | 5,539,283.6 (3,765,530.7 to 8,424,203.9) | 3,331,990.2 (2,271,196.2 to 5,021,439.3) | -39.8% (-47.5% to -29.1%) | 18,705.4 (12,989.3 to 27,176.4) | 19,229.0 (13,541.9 to 27,721.3) | 2.8% (-9.9% to 21.3%) |
| Thailand | Male | Caries of permanent teeth | 9,135.6 (3,985.6 to 17,367.8) | 10,835.8 (4,717.4 to 21,708.5) | 18.6% (2.6% to 36.4%) | 32.3 (14.3 to 60.9) | 30.2 (13.1 to 58.7) | -6.4% (-15.1% to 3.7%) | 9,211,134.7 (7,525,481.7 to 11,272,115.8) | 11,030,179.4 (9,009,377.2 to 13,128,449.4) | 19.7% (3.9% to 37.4%) | 32,759.9 (27,225.8 to 39,093.9) | 30,577.3 (25,117.6 to 37,098.1) | -6.7% (-15.0% to 3.3%) | 9,936,370.7 (8,215,694.1 to 11,563,769.8) | 10,969,017.2 (9,515,678.4 to 12,609,367.0) | 10.4% (-1.4% to 26.5%) | 32,966.8 (27,553.4 to 38,301.4) | 33,721.6 (28,841.8 to 39,097.4) | 2.3% (-7.2% to 14.4%) |
| Thailand | Male | Periodontal diseases | 16,003.4 (6,272.9 to 33,639.5) | 38,017.1 (14,926.9 to 76,222.1) | 137.6% (68.9% to 224.3%) | 71.6 (28.5 to 149.3) | 81.7 (32.3 to 165.2) | 14.1% (-18.0% to 56.2%) | 2,448,958.0 (1,950,592.1 to 3,071,876.4) | 5,867,571.9 (4,701,115.1 to 7,221,312.3) | 139.6% (69.8% to 225.9%) | 11,022.6 (8,921.4 to 13,682.4) | 12,578.6 (9,991.7 to 15,862.9) | 14.1% (-18.0% to 54.8%) | 236,337.8 (189,176.0 to 280,890.2) | 467,413.6 (402,950.1 to 533,523.3) | 97.8% (57.2% to 155.5%) | 984.8 (805.1 to 1,147.7) | 1,085.6 (924.3 to 1,303.4) | 10.2% (-11.2% to 43.4%) |
| Thailand | Male | Edentulism | 13,290.1 (8,393.1 to 19,216.7) | 34,804.1 (22,202.7 to 50,273.9) | 161.9% (140.1% to 182.1%) | 84.6 (53.9 to 122.5) | 71.2 (45.5 to 102.8) | -15.8% (-20.6% to -10.0%) | 482,941.4 (376,632.1 to 600,267.3) | 1,276,566.7 (986,694.2 to 1,594,128.6) | 164.3% (144.4% to 182.6%) | 3,115.6 (2,460.0 to 3,885.4) | 2,614.9 (2,028.5 to 3,244.3) | -16.1% (-20.6% to -10.7%) | 44,822.8 (35,343.0 to 56,391.1) | 109,398.5 (84,514.8 to 137,841.9) | 144.1% (126.0% to 164.9%) | 256.7 (204.5 to 317.6) | 218.3 (171.6 to 272.6) | -15.0% (-20.3% to -9.3%) |
| Thailand | Male | Other oral disorders | 13,741.6 (8,311.8 to 20,944.4) | 17,730.8 (10,972.3 to 26,144.3) | 29.0% (22.2% to 36.7%) | 49.6 (30.1 to 74.8) | 49.8 (30.5 to 74.1) | 0.4% (-4.2% to 5.1%) | 471,411.1 (448,282.1 to 495,086.3) | 613,889.3 (585,464.5 to 641,973.2) | 30.2% (26.8% to 33.8%) | 1,710.3 (1,630.8 to 1,786.0) | 1,710.3 (1,630.8 to 1,786.0) | 0.0% (0.0% to 0.0%) | NA | NA | NA | NA | NA | NA |
| Viet Nam | Both | Main Oral disorders | 117,753.8 (69,966.9 to 180,357.2) | 223,422.0 (133,286.8 to 334,996.1) | 89.7% (74.1% to 110.5%) | 236.4 (142.3 to 356.3) | 217.2 (129.6 to 324.8) | -8.1% (-15.2% to 1.6%) | 30,641,800.6 (27,163,360.1 to 34,354,088.8) | 44,022,357.6 (38,526,767.4 to 49,099,082.8) | 43.7% (33.5% to 54.5%) | 46,628.2 (41,756.8 to 51,648.5) | 43,493.7 (38,449.0 to 48,337.0) | -6.7% (-12.0% to -1.1%) | 41,741,287.9 (34,899,528.6 to 50,790,290.7) | 52,876,396.6 (44,780,245.2 to 62,408,498.8) | 26.7% (17.3% to 36.6%) | 54,269.1 (46,077.1 to 64,179.3) | 54,396.0 (45,551.6 to 64,578.4) | 0.2% (-7.1% to 6.4%) |
| Viet Nam | Both | Caries of deciduous teeth | 3,232.9 (1,366.1 to 6,422.8) | 2,961.8 (1,294.0 to 5,683.5) | -8.4% (-27.3% to 15.5%) | 3.4 (1.5 to 6.8) | 3.4 (1.5 to 6.5) | -1.2% (-21.9% to 24.0%) | 8,479,096.4 (6,859,378.8 to 10,233,764.1) | 7,692,459.2 (6,188,001.8 to 9,304,928.5) | -9.3% (-17.3% to 2.5%) | 9,038.1 (7,306.4 to 10,927.5) | 8,845.4 (7,119.2 to 10,655.8) | -2.1% (-10.8% to 10.4%) | 18,764,152.9 (13,626,554.3 to 27,327,933.8) | 17,657,434.4 (12,590,466.7 to 25,928,833.3) | -5.9% (-13.4% to 2.2%) | 20,113.7 (14,551.2 to 29,536.3) | 20,037.2 (14,372.5 to 29,371.2) | -0.4% (-8.0% to 8.1%) |
| Viet Nam | Both | Caries of permanent teeth | 19,700.4 (8,471.5 to 37,676.1) | 28,727.2 (12,610.1 to 55,564.8) | 45.8% (30.1% to 63.2%) | 31.4 (13.7 to 60.7) | 27.5 (12.1 to 53.4) | -12.3% (-19.9% to -4.9%) | 19,871,728.0 (16,430,134.0 to 23,881,755.9) | 29,059,937.7 (23,188,093.2 to 34,807,069.2) | 46.2% (30.2% to 63.1%) | 31,842.5 (26,534.5 to 37,499.8) | 27,864.3 (22,549.3 to 33,331.3) | -12.5% (-19.9% to -5.0%) | 22,580,238.1 (18,945,522.7 to 26,075,279.4) | 34,181,345.3 (29,040,885.5 to 39,761,757.0) | 51.4% (34.7% to 65.3%) | 33,289.7 (28,132.6 to 38,190.4) | 33,401.2 (28,354.6 to 38,688.2) | 0.3% (-10.4% to 8.8%) |
| Viet Nam | Both | Periodontal diseases | 16,212.6 (6,265.2 to 35,301.2) | 50,041.6 (19,277.2 to 106,343.1) | 208.7% (99.4% to 411.8%) | 35.7 (13.5 to 77.1) | 45.5 (17.5 to 96.7) | 27.5% (-17.0% to 108.8%) | 2,487,917.7 (1,754,881.6 to 3,308,619.6) | 7,737,647.0 (5,575,959.8 to 10,253,345.4) | 211.0% (100.3% to 409.4%) | 5,496.6 (3,873.3 to 7,341.5) | 7,059.9 (5,162.0 to 9,184.7) | 28.4% (-16.6% to 107.7%) | 267,065.9 (191,321.4 to 345,024.3) | 784,468.4 (587,668.9 to 988,093.4) | 193.7% (104.3% to 344.3%) | 559.0 (395.4 to 723.5) | 714.2 (550.7 to 891.9) | 27.8% (-10.8% to 93.5%) |
| Viet Nam | Both | Edentulism | 44,817.5 (28,862.7 to 63,505.2) | 85,177.1 (53,528.0 to 121,874.5) | 90.1% (80.5% to 100.2%) | 111.9 (72.0 to 159.7) | 86.8 (55.0 to 124.2) | -22.4% (-25.8% to -18.4%) | 1,630,496.1 (1,289,220.3 to 2,013,829.1) | 3,091,527.4 (2,399,898.4 to 3,828,197.8) | 89.6% (81.0% to 99.0%) | 4,096.9 (3,256.1 to 5,033.8) | 3,176.7 (2,468.8 to 3,929.2) | -22.5% (-25.7% to -18.9%) | 129,831.1 (104,669.6 to 162,133.0) | 253,148.4 (197,241.0 to 319,372.6) | 95.0% (84.5% to 105.5%) | 306.7 (246.7 to 377.5) | 243.4 (192.2 to 302.2) | -20.6% (-24.1% to -17.2%) |
| Viet Nam | Both | Other oral disorders | 33,790.4 (20,571.2 to 50,088.2) | 56,514.2 (34,616.7 to 84,855.1) | 67.2% (60.5% to 74.4%) | 54.0 (33.0 to 79.8) | 53.9 (33.0 to 80.8) | -0.1% (-3.3% to 3.0%) | 1,158,103.8 (1,108,200.3 to 1,208,898.9) | 1,943,343.8 (1,860,426.2 to 2,021,323.8) | 67.8% (64.6% to 71.3%) | 1,861.1 (1,780.7 to 1,932.7) | 1,853.6 (1,773.8 to 1,925.3) | -0.4% (-0.5% to -0.3%) | NA | NA | NA | NA | NA | NA |
| Viet Nam | Female | Main Oral disorders | 70,790.3 (42,686.8 to 106,559.7) | 130,865.2 (77,737.5 to 193,590.9) | 84.9% (70.0% to 102.9%) | 260.8 (157.6 to 390.2) | 238.0 (141.8 to 351.2) | -8.7% (-15.4% to 0.2%) | 16,042,522.9 (14,259,847.5 to 18,089,420.0) | 22,611,033.3 (19,983,235.1 to 25,447,756.0) | 40.9% (30.3% to 52.8%) | 47,274.3 (42,173.9 to 52,513.8) | 44,143.0 (39,063.8 to 49,525.7) | -6.6% (-12.5% to -0.3%) | 21,221,572.1 (17,626,908.6 to 25,272,334.0) | 26,069,586.2 (21,983,977.4 to 30,772,758.9) | 22.8% (13.0% to 34.2%) | 54,649.3 (46,282.6 to 63,980.8) | 54,570.6 (45,645.8 to 64,924.3) | -0.1% (-7.5% to 6.4%) |
| Viet Nam | Female | Caries of deciduous teeth | 1,571.4 (672.3 to 3,043.0) | 1,409.2 (610.3 to 2,836.7) | -10.3% (-29.2% to 15.5%) | 3.4 (1.5 to 6.6) | 3.4 (1.5 to 6.8) | -1.3% (-21.9% to 26.6%) | 4,123,025.2 (3,359,611.4 to 5,010,465.2) | 3,666,544.2 (2,908,853.9 to 4,505,718.8) | -11.1% (-19.1% to 0.6%) | 8,999.6 (7,320.0 to 10,941.0) | 8,813.7 (6,993.2 to 10,794.2) | -2.1% (-10.9% to 10.4%) | 9,233,604.1 (6,655,585.4 to 13,206,635.3) | 8,495,331.6 (5,962,889.6 to 12,679,413.0) | -8.0% (-16.6% to 1.6%) | 20,260.9 (14,534.4 to 29,277.2) | 20,160.1 (14,236.1 to 30,019.6) | -0.5% (-9.8% to 9.3%) |
| Viet Nam | Female | Caries of permanent teeth | 10,382.0 (4,476.9 to 19,971.3) | 14,595.8 (6,392.9 to 28,021.7) | 40.6% (24.4% to 59.3%) | 31.6 (14.0 to 60.4) | 27.8 (12.1 to 53.4) | -12.1% (-20.2% to -2.9%) | 10,502,826.4 (8,671,995.9 to 12,672,161.4) | 14,840,537.5 (12,079,173.6 to 17,818,700.1) | 41.3% (26.0% to 60.0%) | 32,142.7 (26,723.5 to 37,963.3) | 28,232.1 (23,206.0 to 33,740.0) | -12.2% (-20.2% to -3.4%) | 11,756,043.3 (9,793,723.9 to 13,592,076.9) | 16,998,978.1 (14,201,630.9 to 19,966,312.8) | 44.6% (28.5% to 60.5%) | 33,475.3 (28,240.4 to 38,322.6) | 33,412.4 (27,892.6 to 39,212.5) | -0.2% (-10.2% to 9.1%) |
| Viet Nam | Female | Periodontal diseases | 8,801.6 (3,404.6 to 19,041.2) | 26,163.1 (10,027.8 to 55,276.3) | 197.3% (93.9% to 378.6%) | 35.1 (13.3 to 75.8) | 44.9 (17.2 to 95.4) | 27.7% (-16.4% to 102.4%) | 1,355,684.3 (961,999.4 to 1,814,076.0) | 4,064,528.4 (2,960,099.4 to 5,319,408.8) | 199.8% (95.1% to 374.3%) | 5,425.3 (3,803.5 to 7,273.1) | 6,984.9 (5,111.5 to 9,057.5) | 28.7% (-16.0% to 103.9%) | 147,197.3 (105,644.2 to 190,431.2) | 413,233.7 (312,846.3 to 514,743.3) | 180.7% (100.4% to 317.7%) | 562.3 (398.6 to 729.4) | 716.3 (551.3 to 889.4) | 27.4% (-9.9% to 93.9%) |
| Viet Nam | Female | Edentulism | 31,146.9 (20,083.4 to 44,412.7) | 58,201.5 (36,581.2 to 83,465.9) | 86.9% (74.1% to 99.2%) | 132.9 (85.3 to 189.2) | 104.2 (65.8 to 149.9) | -21.6% (-26.4% to -16.2%) | 1,135,211.2 (896,884.8 to 1,382,846.0) | 2,118,691.1 (1,641,159.5 to 2,610,245.6) | 86.6% (75.9% to 98.2%) | 4,858.0 (3,847.3 to 5,917.1) | 3,805.6 (2,970.0 to 4,689.3) | -21.7% (-25.8% to -17.1%) | 84,727.4 (68,505.4 to 102,998.0) | 162,042.8 (127,286.0 to 203,232.3) | 91.3% (79.6% to 103.8%) | 350.8 (285.1 to 428.7) | 281.8 (223.8 to 348.0) | -19.7% (-24.1% to -15.0%) |
| Viet Nam | Female | Other oral disorders | 18,888.3 (11,629.7 to 27,924.9) | 30,495.6 (18,859.0 to 46,215.8) | 61.5% (53.2% to 70.0%) | 57.7 (35.5 to 84.8) | 57.8 (35.5 to 87.8) | 0.1% (-4.4% to 4.2%) | 648,830.4 (616,747.3 to 679,938.0) | 1,054,134.6 (1,003,797.5 to 1,103,256.1) | 62.5% (59.2% to 65.9%) | 1,993.8 (1,903.3 to 2,082.6) | 1,993.8 (1,903.3 to 2,082.6) | 0.0% (0.0% to 0.0%) | NA | NA | NA | NA | NA | NA |
| Viet Nam | Male | Main Oral disorders | 46,963.5 (27,805.2 to 72,553.6) | 92,556.8 (54,184.9 to 145,223.4) | 97.1% (78.0% to 122.3%) | 204.9 (122.5 to 311.0) | 192.6 (113.1 to 295.2) | -6.0% (-14.6% to 5.6%) | 14,599,277.7 (12,894,833.8 to 16,462,862.7) | 21,411,324.3 (18,512,262.1 to 24,203,397.7) | 46.7% (33.6% to 59.9%) | 45,825.7 (40,837.0 to 51,107.3) | 42,783.1 (37,572.3 to 48,033.9) | -6.6% (-13.1% to 0.3%) | 20,519,715.8 (17,113,603.9 to 25,567,513.5) | 26,806,810.4 (22,547,722.9 to 31,801,222.1) | 30.6% (17.5% to 43.5%) | 53,876.8 (45,433.5 to 64,161.1) | 54,230.9 (45,499.3 to 64,413.1) | 0.7% (-8.3% to 9.0%) |
| Viet Nam | Male | Caries of deciduous teeth | 1,661.5 (696.2 to 3,278.5) | 1,552.6 (673.5 to 3,089.6) | -6.6% (-27.1% to 20.7%) | 3.5 (1.5 to 6.8) | 3.4 (1.5 to 6.8) | -1.1% (-22.9% to 27.5%) | 4,356,071.2 (3,438,203.1 to 5,257,265.6) | 4,025,915.1 (3,244,149.5 to 4,952,682.6) | -7.6% (-19.1% to 7.3%) | 9,074.9 (7,157.7 to 10,956.0) | 8,874.5 (7,142.4 to 10,880.1) | -2.2% (-14.4% to 13.3%) | 9,530,548.7 (6,963,832.1 to 14,149,567.6) | 9,162,102.8 (6,477,703.7 to 13,232,754.9) | -3.9% (-16.7% to 8.1%) | 19,973.0 (14,533.5 to 29,855.0) | 19,924.4 (14,195.4 to 28,846.8) | -0.2% (-12.4% to 12.5%) |
| Viet Nam | Male | Caries of permanent teeth | 9,318.4 (4,014.0 to 17,742.3) | 14,131.4 (6,174.0 to 27,718.5) | 51.7% (32.2% to 72.0%) | 31.2 (13.6 to 61.0) | 27.3 (11.9 to 52.8) | -12.5% (-22.0% to -2.8%) | 9,368,901.7 (7,693,561.2 to 11,367,617.4) | 14,219,400.2 (11,253,651.4 to 17,216,253.0) | 51.8% (32.2% to 71.4%) | 31,506.2 (26,259.7 to 37,260.9) | 27,487.6 (22,125.8 to 33,211.4) | -12.8% (-21.9% to -3.4%) | 10,824,194.8 (8,995,408.4 to 12,626,611.9) | 17,182,367.2 (14,587,605.6 to 19,840,925.8) | 58.7% (38.0% to 75.3%) | 33,094.7 (27,586.2 to 38,499.6) | 33,392.3 (28,442.8 to 38,517.3) | 0.9% (-11.7% to 11.8%) |
| Viet Nam | Male | Periodontal diseases | 7,411.0 (2,865.7 to 16,124.0) | 23,878.6 (9,140.5 to 51,002.7) | 222.2% (104.7% to 444.3%) | 36.5 (13.8 to 78.7) | 46.4 (17.7 to 97.1) | 27.3% (-17.7% to 110.6%) | 1,132,233.4 (787,518.3 to 1,503,776.3) | 3,673,118.6 (2,624,384.1 to 5,052,047.9) | 224.4% (105.9% to 440.6%) | 5,599.9 (3,889.7 to 7,484.2) | 7,175.6 (5,312.1 to 9,571.3) | 28.1% (-17.4% to 109.7%) | 119,868.6 (84,723.5 to 155,162.8) | 371,234.8 (273,583.3 to 472,572.6) | 209.7% (107.7% to 388.4%) | 554.9 (390.3 to 716.7) | 712.2 (546.3 to 890.0) | 28.3% (-11.5% to 98.0%) |
| Viet Nam | Male | Edentulism | 13,670.5 (8,744.7 to 19,631.9) | 26,975.6 (16,745.4 to 38,772.9) | 97.3% (84.5% to 109.9%) | 84.1 (53.7 to 120.8) | 65.6 (41.9 to 94.9) | -22.1% (-26.8% to -17.2%) | 495,284.8 (385,903.3 to 625,349.7) | 972,836.2 (743,708.4 to 1,227,132.9) | 96.4% (86.0% to 107.4%) | 3,079.1 (2,425.5 to 3,860.1) | 2,396.7 (1,849.4 to 2,991.2) | -22.2% (-26.3% to -18.2%) | 45,103.7 (35,461.0 to 57,476.0) | 91,105.6 (70,170.4 to 116,143.5) | 102.0% (87.7% to 115.2%) | 254.1 (202.0 to 322.8) | 201.9 (158.1 to 255.2) | -20.6% (-25.0% to -16.4%) |
| Viet Nam | Male | Other oral disorders | 14,902.1 (9,006.1 to 22,388.8) | 26,018.6 (15,951.0 to 38,794.4) | 74.6% (65.6% to 84.9%) | 49.7 (30.3 to 74.6) | 50.0 (30.7 to 74.5) | 0.5% (-4.2% to 5.1%) | 509,273.3 (483,678.3 to 536,523.1) | 889,209.2 (847,372.9 to 931,708.2) | 74.6% (70.5% to 78.6%) | 1,710.3 (1,630.8 to 1,786.0) | 1,710.3 (1,630.8 to 1,786.0) | 0.0% (0.0% to 0.0%) | NA | NA | NA | NA | NA | NA |

**Appendix Table 4.** Number and rate of oral disorders by age group, sex, and country in 2021.

| measure | sex | age | cause | Number | Rate |
| --- | --- | --- | --- | --- | --- |
| Prevalence | Female | <5 | Main Oral disorders | 10,790,670.5 (8,459,481.1 to 12,822,770.9) | 40,712.0 (31,916.7 to 48,378.8) |
| Prevalence | Female | <5 | Caries of deciduous teeth | 10,697,635.8 (8,337,869.3 to 12,758,598.1) | 40,361.0 (31,457.8 to 48,136.7) |
| Prevalence | Female | <5 | Caries of permanent teeth | 0.0 (0.0 to 0.0) | 0.0 (0.0 to 0.0) |
| Prevalence | Female | <5 | Periodontal diseases | 0.0 (0.0 to 0.0) | 0.0 (0.0 to 0.0) |
| Prevalence | Female | <5 | Edentulism | 0.0 (0.0 to 0.0) | 0.0 (0.0 to 0.0) |
| Prevalence | Female | <5 | Other oral disorders | 242,946.6 (213,355.0 to 276,576.1) | 916.6 (805.0 to 1,043.5) |
| Prevalence | Female | 5-9 | Main Oral disorders | 14,103,914.9 (11,204,997.3 to 17,295,098.7) | 51,381.5 (40,820.5 to 63,007.2) |
| Prevalence | Female | 5-9 | Caries of deciduous teeth | 12,243,827.9 (9,386,928.7 to 15,702,559.6) | 44,605.1 (34,197.2 to 57,205.5) |
| Prevalence | Female | 5-9 | Caries of permanent teeth | 3,106,978.4 (1,475,629.9 to 5,479,636.9) | 11,318.9 (5,375.8 to 19,962.7) |
| Prevalence | Female | 5-9 | Periodontal diseases | 0.0 (0.0 to 0.0) | 0.0 (0.0 to 0.0) |
| Prevalence | Female | 5-9 | Edentulism | 0.0 (0.0 to 0.0) | 0.0 (0.0 to 0.0) |
| Prevalence | Female | 5-9 | Other oral disorders | 305,126.6 (257,961.3 to 350,254.4) | 1,111.6 (939.8 to 1,276.0) |
| Prevalence | Female | 10-14 | Main Oral disorders | 8,920,921.4 (6,112,289.4 to 12,166,802.3) | 32,725.6 (22,422.4 to 44,632.8) |
| Prevalence | Female | 10-14 | Caries of deciduous teeth | 1,672,435.0 (564,825.3 to 2,965,703.2) | 6,135.2 (2,072.0 to 10,879.4) |
| Prevalence | Female | 10-14 | Caries of permanent teeth | 7,494,568.7 (4,802,956.9 to 10,690,009.5) | 27,493.1 (17,619.2 to 39,215.3) |
| Prevalence | Female | 10-14 | Periodontal diseases | 0.0 (0.0 to 0.0) | 0.0 (0.0 to 0.0) |
| Prevalence | Female | 10-14 | Edentulism | 0.0 (0.0 to 0.0) | 0.0 (0.0 to 0.0) |
| Prevalence | Female | 10-14 | Other oral disorders | 320,561.1 (273,723.4 to 366,991.8) | 1,175.9 (1,004.1 to 1,346.3) |
| Prevalence | Female | 15-19 | Main Oral disorders | 8,674,949.2 (5,819,873.4 to 11,979,769.0) | 32,525.7 (21,820.9 to 44,916.8) |
| Prevalence | Female | 15-19 | Caries of deciduous teeth | 0.0 (0.0 to 0.0) | 0.0 (0.0 to 0.0) |
| Prevalence | Female | 15-19 | Caries of permanent teeth | 8,248,520.4 (5,332,389.1 to 11,644,417.0) | 30,926.9 (19,993.2 to 43,659.4) |
| Prevalence | Female | 15-19 | Periodontal diseases | 152,167.2 (86,236.6 to 252,712.5) | 570.5 (323.3 to 947.5) |
| Prevalence | Female | 15-19 | Edentulism | 0.0 (0.0 to 0.0) | 0.0 (0.0 to 0.0) |
| Prevalence | Female | 15-19 | Other oral disorders | 468,210.2 (410,003.5 to 527,620.5) | 1,755.5 (1,537.3 to 1,978.3) |
| Prevalence | Female | 20-24 | Main Oral disorders | 10,728,977.6 (8,265,608.3 to 13,684,752.3) | 40,032.7 (30,841.2 to 51,061.5) |
| Prevalence | Female | 20-24 | Caries of deciduous teeth | 0.0 (0.0 to 0.0) | 0.0 (0.0 to 0.0) |
| Prevalence | Female | 20-24 | Caries of permanent teeth | 9,975,996.4 (7,406,349.7 to 13,070,817.6) | 37,223.1 (27,635.1 to 48,770.7) |
| Prevalence | Female | 20-24 | Periodontal diseases | 589,369.5 (355,605.1 to 898,707.4) | 2,199.1 (1,326.9 to 3,353.3) |
| Prevalence | Female | 20-24 | Edentulism | 51,321.4 (30,587.5 to 74,360.2) | 191.5 (114.1 to 277.5) |
| Prevalence | Female | 20-24 | Other oral disorders | 574,287.6 (513,205.7 to 640,343.3) | 2,142.8 (1,914.9 to 2,389.3) |
| Prevalence | Female | 25-29 | Main Oral disorders | 11,616,028.3 (9,032,747.7 to 15,676,805.9) | 42,556.8 (33,092.6 to 57,433.9) |
| Prevalence | Female | 25-29 | Caries of deciduous teeth | 0.0 (0.0 to 0.0) | 0.0 (0.0 to 0.0) |
| Prevalence | Female | 25-29 | Caries of permanent teeth | 10,217,246.0 (7,406,767.5 to 14,710,524.6) | 37,432.1 (27,135.6 to 53,893.8) |
| Prevalence | Female | 25-29 | Periodontal diseases | 1,492,992.7 (942,413.0 to 2,164,856.8) | 5,469.8 (3,452.6 to 7,931.2) |
| Prevalence | Female | 25-29 | Edentulism | 155,478.1 (102,339.0 to 218,149.5) | 569.6 (374.9 to 799.2) |
| Prevalence | Female | 25-29 | Other oral disorders | 638,197.1 (560,750.8 to 717,227.8) | 2,338.1 (2,054.4 to 2,627.7) |
| Prevalence | Female | 30-34 | Main Oral disorders | 11,801,359.0 (9,400,311.5 to 15,325,200.2) | 44,372.5 (35,344.7 to 57,622.0) |
| Prevalence | Female | 30-34 | Caries of deciduous teeth | 0.0 (0.0 to 0.0) | 0.0 (0.0 to 0.0) |
| Prevalence | Female | 30-34 | Caries of permanent teeth | 9,596,006.5 (6,978,669.6 to 13,533,573.9) | 36,080.5 (26,239.4 to 50,885.5) |
| Prevalence | Female | 30-34 | Periodontal diseases | 2,618,526.6 (1,759,319.4 to 3,642,004.3) | 9,845.5 (6,614.9 to 13,693.7) |
| Prevalence | Female | 30-34 | Edentulism | 295,641.2 (214,303.3 to 400,345.3) | 1,111.6 (805.8 to 1,505.3) |
| Prevalence | Female | 30-34 | Other oral disorders | 658,261.9 (584,648.5 to 738,901.8) | 2,475.0 (2,198.2 to 2,778.2) |
| Prevalence | Female | 35-39 | Main Oral disorders | 12,352,139.8 (9,719,670.1 to 15,107,826.8) | 48,935.2 (38,506.2 to 59,852.3) |
| Prevalence | Female | 35-39 | Caries of deciduous teeth | 0.0 (0.0 to 0.0) | 0.0 (0.0 to 0.0) |
| Prevalence | Female | 35-39 | Caries of permanent teeth | 9,484,622.6 (6,282,028.4 to 13,038,863.2) | 37,575.0 (24,887.4 to 51,655.7) |
| Prevalence | Female | 35-39 | Periodontal diseases | 3,696,043.2 (2,602,317.4 to 4,851,839.6) | 14,642.5 (10,309.5 to 19,221.4) |
| Prevalence | Female | 35-39 | Edentulism | 464,355.2 (342,266.1 to 622,543.6) | 1,839.6 (1,355.9 to 2,466.3) |
| Prevalence | Female | 35-39 | Other oral disorders | 644,748.0 (573,248.8 to 724,761.0) | 2,554.3 (2,271.0 to 2,871.3) |
| Prevalence | Female | 40-44 | Main Oral disorders | 12,534,355.7 (10,023,612.7 to 15,047,087.3) | 52,672.4 (42,121.6 to 63,231.5) |
| Prevalence | Female | 40-44 | Caries of deciduous teeth | 0.0 (0.0 to 0.0) | 0.0 (0.0 to 0.0) |
| Prevalence | Female | 40-44 | Caries of permanent teeth | 9,063,524.5 (6,081,914.2 to 12,337,642.7) | 38,087.1 (25,557.7 to 51,845.7) |
| Prevalence | Female | 40-44 | Periodontal diseases | 4,616,133.7 (3,356,395.4 to 5,884,701.6) | 19,398.1 (14,104.4 to 24,728.9) |
| Prevalence | Female | 40-44 | Edentulism | 692,892.8 (519,883.4 to 904,232.3) | 2,911.7 (2,184.7 to 3,799.8) |
| Prevalence | Female | 40-44 | Other oral disorders | 622,890.6 (557,317.8 to 703,006.3) | 2,617.5 (2,342.0 to 2,954.2) |
| Prevalence | Female | 45-49 | Main Oral disorders | 12,045,636.9 (10,042,154.8 to 14,215,503.2) | 55,574.2 (46,330.8 to 65,585.2) |
| Prevalence | Female | 45-49 | Caries of deciduous teeth | 0.0 (0.0 to 0.0) | 0.0 (0.0 to 0.0) |
| Prevalence | Female | 45-49 | Caries of permanent teeth | 8,204,700.3 (5,528,233.5 to 11,146,905.5) | 37,853.5 (25,505.3 to 51,427.8) |
| Prevalence | Female | 45-49 | Periodontal diseases | 5,056,848.9 (3,897,561.9 to 6,220,687.8) | 23,330.5 (17,981.9 to 28,700.0) |
| Prevalence | Female | 45-49 | Edentulism | 996,145.9 (769,203.9 to 1,321,459.4) | 4,595.9 (3,548.8 to 6,096.7) |
| Prevalence | Female | 45-49 | Other oral disorders | 577,334.5 (514,697.2 to 652,906.4) | 2,663.6 (2,374.6 to 3,012.3) |
| Prevalence | Female | 50-54 | Main Oral disorders | 11,560,451.4 (9,641,828.3 to 13,665,412.1) | 58,261.2 (48,591.9 to 68,869.5) |
| Prevalence | Female | 50-54 | Caries of deciduous teeth | 0.0 (0.0 to 0.0) | 0.0 (0.0 to 0.0) |
| Prevalence | Female | 50-54 | Caries of permanent teeth | 7,535,470.4 (4,889,136.6 to 10,523,373.7) | 37,976.5 (24,639.8 to 53,034.6) |
| Prevalence | Female | 50-54 | Periodontal diseases | 5,132,021.5 (4,014,300.2 to 6,177,943.9) | 25,863.8 (20,230.9 to 31,135.0) |
| Prevalence | Female | 50-54 | Edentulism | 1,414,472.6 (1,100,476.8 to 1,792,290.7) | 7,128.5 (5,546.1 to 9,032.6) |
| Prevalence | Female | 50-54 | Other oral disorders | 530,140.8 (470,835.9 to 593,953.4) | 2,671.7 (2,372.9 to 2,993.3) |
| Prevalence | Female | 55-59 | Main Oral disorders | 10,531,721.7 (9,119,615.1 to 12,229,729.1) | 60,657.6 (52,524.6 to 70,437.3) |
| Prevalence | Female | 55-59 | Caries of deciduous teeth | 0.0 (0.0 to 0.0) | 0.0 (0.0 to 0.0) |
| Prevalence | Female | 55-59 | Caries of permanent teeth | 6,638,130.5 (4,428,751.3 to 9,202,727.3) | 38,232.4 (25,507.5 to 53,003.3) |
| Prevalence | Female | 55-59 | Periodontal diseases | 4,681,913.7 (3,759,851.6 to 5,536,665.8) | 26,965.6 (21,654.9 to 31,888.5) |
| Prevalence | Female | 55-59 | Edentulism | 1,868,024.5 (1,468,923.2 to 2,393,546.1) | 10,758.9 (8,460.3 to 13,785.7) |
| Prevalence | Female | 55-59 | Other oral disorders | 458,834.5 (404,678.2 to 518,259.7) | 2,642.7 (2,330.8 to 2,984.9) |
| Prevalence | Female | 60-64 | Main Oral disorders | 9,044,550.0 (8,081,671.7 to 10,216,432.4) | 62,880.9 (56,186.6 to 71,028.3) |
| Prevalence | Female | 60-64 | Caries of deciduous teeth | 0.0 (0.0 to 0.0) | 0.0 (0.0 to 0.0) |
| Prevalence | Female | 60-64 | Caries of permanent teeth | 5,552,584.8 (4,024,200.7 to 7,418,807.2) | 38,603.5 (27,977.7 to 51,578.2) |
| Prevalence | Female | 60-64 | Periodontal diseases | 3,811,964.8 (3,050,992.6 to 4,464,483.1) | 26,502.1 (21,211.6 to 31,038.7) |
| Prevalence | Female | 60-64 | Edentulism | 2,272,437.2 (1,805,268.3 to 2,839,943.0) | 15,798.8 (12,550.9 to 19,744.3) |
| Prevalence | Female | 60-64 | Other oral disorders | 377,619.6 (336,074.4 to 425,245.6) | 2,625.3 (2,336.5 to 2,956.5) |
| Prevalence | Female | 65-69 | Main Oral disorders | 7,040,101.3 (6,361,175.7 to 7,714,504.9) | 65,086.1 (58,809.4 to 71,321.0) |
| Prevalence | Female | 65-69 | Caries of deciduous teeth | 0.0 (0.0 to 0.0) | 0.0 (0.0 to 0.0) |
| Prevalence | Female | 65-69 | Caries of permanent teeth | 4,184,613.7 (3,090,305.5 to 5,304,271.7) | 38,687.0 (28,570.0 to 49,038.3) |
| Prevalence | Female | 65-69 | Periodontal diseases | 2,743,325.3 (2,211,922.4 to 3,235,265.0) | 25,362.2 (20,449.3 to 29,910.2) |
| Prevalence | Female | 65-69 | Edentulism | 2,368,976.1 (1,878,703.9 to 2,923,588.7) | 21,901.3 (17,368.7 to 27,028.7) |
| Prevalence | Female | 65-69 | Other oral disorders | 283,235.6 (250,615.0 to 312,981.2) | 2,618.5 (2,316.9 to 2,893.5) |
| Prevalence | Female | 70-74 | Main Oral disorders | 4,770,785.2 (4,286,503.0 to 5,250,808.6) | 67,258.9 (60,431.4 to 74,026.3) |
| Prevalence | Female | 70-74 | Caries of deciduous teeth | 0.0 (0.0 to 0.0) | 0.0 (0.0 to 0.0) |
| Prevalence | Female | 70-74 | Caries of permanent teeth | 2,750,286.8 (1,881,680.5 to 3,557,779.2) | 38,773.8 (26,528.1 to 50,157.8) |
| Prevalence | Female | 70-74 | Periodontal diseases | 1,693,732.1 (1,368,910.5 to 2,040,200.8) | 23,878.4 (19,299.0 to 28,762.9) |
| Prevalence | Female | 70-74 | Edentulism | 1,997,435.4 (1,618,077.2 to 2,491,692.5) | 28,160.0 (22,811.8 to 35,128.1) |
| Prevalence | Female | 70-74 | Other oral disorders | 174,411.1 (153,372.2 to 195,242.2) | 2,458.9 (2,162.3 to 2,752.5) |
| Prevalence | Female | 75-79 | Main Oral disorders | 3,058,041.3 (2,804,813.3 to 3,306,804.0) | 67,726.8 (62,118.5 to 73,236.2) |
| Prevalence | Female | 75-79 | Caries of deciduous teeth | 0.0 (0.0 to 0.0) | 0.0 (0.0 to 0.0) |
| Prevalence | Female | 75-79 | Caries of permanent teeth | 1,640,925.7 (1,217,921.6 to 2,106,134.0) | 36,341.8 (26,973.4 to 46,644.8) |
| Prevalence | Female | 75-79 | Periodontal diseases | 1,025,994.6 (821,727.9 to 1,230,279.2) | 22,722.8 (18,198.9 to 27,247.1) |
| Prevalence | Female | 75-79 | Edentulism | 1,492,030.1 (1,226,741.7 to 1,807,814.0) | 33,044.2 (27,168.8 to 40,037.9) |
| Prevalence | Female | 75-79 | Other oral disorders | 96,999.7 (86,554.0 to 107,340.1) | 2,148.3 (1,916.9 to 2,377.3) |
| Prevalence | Female | 80-84 | Main Oral disorders | 1,900,813.3 (1,732,134.7 to 2,070,163.0) | 67,656.5 (61,652.7 to 73,684.2) |
| Prevalence | Female | 80-84 | Caries of deciduous teeth | 0.0 (0.0 to 0.0) | 0.0 (0.0 to 0.0) |
| Prevalence | Female | 80-84 | Caries of permanent teeth | 945,350.1 (662,080.1 to 1,268,643.8) | 33,648.3 (23,565.7 to 45,155.4) |
| Prevalence | Female | 80-84 | Periodontal diseases | 607,712.4 (484,866.8 to 736,587.7) | 21,630.6 (17,258.1 to 26,217.7) |
| Prevalence | Female | 80-84 | Edentulism | 1,032,412.6 (852,582.6 to 1,226,455.8) | 36,747.1 (30,346.4 to 43,653.8) |
| Prevalence | Female | 80-84 | Other oral disorders | 49,946.2 (43,567.6 to 56,187.2) | 1,777.8 (1,550.7 to 1,999.9) |
| Prevalence | Female | 85-89 | Main Oral disorders | 942,826.0 (851,079.8 to 1,033,023.2) | 66,368.3 (59,910.0 to 72,717.6) |
| Prevalence | Female | 85-89 | Caries of deciduous teeth | 0.0 (0.0 to 0.0) | 0.0 (0.0 to 0.0) |
| Prevalence | Female | 85-89 | Caries of permanent teeth | 418,020.9 (276,131.5 to 587,174.6) | 29,425.7 (19,437.7 to 41,333.0) |
| Prevalence | Female | 85-89 | Periodontal diseases | 298,394.1 (230,332.4 to 373,749.4) | 21,004.9 (16,213.8 to 26,309.3) |
| Prevalence | Female | 85-89 | Edentulism | 552,793.1 (456,132.0 to 653,129.4) | 38,912.8 (32,108.5 to 45,975.7) |
| Prevalence | Female | 85-89 | Other oral disorders | 19,111.9 (16,836.5 to 21,397.4) | 1,345.3 (1,185.2 to 1,506.2) |
| Prevalence | Female | 90-94 | Main Oral disorders | 337,401.7 (299,466.7 to 373,245.0) | 64,386.6 (57,147.5 to 71,226.6) |
| Prevalence | Female | 90-94 | Caries of deciduous teeth | 0.0 (0.0 to 0.0) | 0.0 (0.0 to 0.0) |
| Prevalence | Female | 90-94 | Caries of permanent teeth | 126,810.8 (73,172.2 to 196,094.1) | 24,199.4 (13,963.5 to 37,420.8) |
| Prevalence | Female | 90-94 | Periodontal diseases | 110,144.3 (83,676.8 to 140,502.8) | 21,018.9 (15,968.1 to 26,812.3) |
| Prevalence | Female | 90-94 | Edentulism | 208,794.8 (166,772.1 to 251,573.3) | 39,844.5 (31,825.3 to 48,007.9) |
| Prevalence | Female | 90-94 | Other oral disorders | 5,973.8 (5,193.6 to 6,809.2) | 1,140.0 (991.1 to 1,299.4) |
| Prevalence | Female | 95+ | Main Oral disorders | 89,926.6 (79,160.3 to 101,467.3) | 61,106.3 (53,790.5 to 68,948.4) |
| Prevalence | Female | 95+ | Caries of deciduous teeth | 0.0 (0.0 to 0.0) | 0.0 (0.0 to 0.0) |
| Prevalence | Female | 95+ | Caries of permanent teeth | 25,138.8 (13,536.1 to 39,713.6) | 17,082.2 (9,197.9 to 26,985.9) |
| Prevalence | Female | 95+ | Periodontal diseases | 31,287.8 (22,780.1 to 40,340.8) | 21,260.5 (15,479.4 to 27,412.1) |
| Prevalence | Female | 95+ | Edentulism | 58,463.3 (44,397.4 to 72,474.4) | 39,726.6 (30,168.6 to 49,247.3) |
| Prevalence | Female | 95+ | Other oral disorders | 1,712.9 (1,431.4 to 2,004.2) | 1,164.0 (972.6 to 1,361.9) |
| Prevalence | Male | <5 | Main Oral disorders | 11,458,646.0 (8,891,140.0 to 13,603,230.5) | 40,686.0 (31,569.6 to 48,300.8) |
| Prevalence | Male | <5 | Caries of deciduous teeth | 11,373,698.5 (8,778,257.5 to 13,539,996.1) | 40,384.4 (31,168.8 to 48,076.3) |
| Prevalence | Male | <5 | Caries of permanent teeth | 0.0 (0.0 to 0.0) | 0.0 (0.0 to 0.0) |
| Prevalence | Male | <5 | Periodontal diseases | 0.0 (0.0 to 0.0) | 0.0 (0.0 to 0.0) |
| Prevalence | Male | <5 | Edentulism | 0.0 (0.0 to 0.0) | 0.0 (0.0 to 0.0) |
| Prevalence | Male | <5 | Other oral disorders | 221,713.5 (194,505.6 to 253,034.9) | 787.2 (690.6 to 898.4) |
| Prevalence | Male | 5-9 | Main Oral disorders | 14,873,921.5 (11,801,915.5 to 18,164,402.7) | 51,132.2 (40,571.5 to 62,443.9) |
| Prevalence | Male | 5-9 | Caries of deciduous teeth | 12,976,485.7 (9,852,498.3 to 16,477,606.4) | 44,609.3 (33,870.0 to 56,645.2) |
| Prevalence | Male | 5-9 | Caries of permanent teeth | 3,199,289.4 (1,509,942.3 to 5,512,372.3) | 10,998.2 (5,190.7 to 18,949.9) |
| Prevalence | Male | 5-9 | Periodontal diseases | 0.0 (0.0 to 0.0) | 0.0 (0.0 to 0.0) |
| Prevalence | Male | 5-9 | Edentulism | 0.0 (0.0 to 0.0) | 0.0 (0.0 to 0.0) |
| Prevalence | Male | 5-9 | Other oral disorders | 277,796.0 (237,760.6 to 320,585.8) | 955.0 (817.4 to 1,102.1) |
| Prevalence | Male | 10-14 | Main Oral disorders | 9,255,072.2 (6,321,080.0 to 12,639,654.9) | 32,027.8 (21,874.5 to 43,740.4) |
| Prevalence | Male | 10-14 | Caries of deciduous teeth | 1,779,831.4 (581,037.4 to 3,155,033.9) | 6,159.2 (2,010.7 to 10,918.2) |
| Prevalence | Male | 10-14 | Caries of permanent teeth | 7,757,532.8 (4,958,114.8 to 11,046,768.9) | 26,845.5 (17,157.9 to 38,228.1) |
| Prevalence | Male | 10-14 | Periodontal diseases | 0.0 (0.0 to 0.0) | 0.0 (0.0 to 0.0) |
| Prevalence | Male | 10-14 | Edentulism | 0.0 (0.0 to 0.0) | 0.0 (0.0 to 0.0) |
| Prevalence | Male | 10-14 | Other oral disorders | 291,492.7 (248,068.9 to 335,375.6) | 1,008.7 (858.5 to 1,160.6) |
| Prevalence | Male | 15-19 | Main Oral disorders | 8,925,556.8 (6,012,505.0 to 12,338,414.4) | 31,824.4 (21,437.8 to 43,993.1) |
| Prevalence | Male | 15-19 | Caries of deciduous teeth | 0.0 (0.0 to 0.0) | 0.0 (0.0 to 0.0) |
| Prevalence | Male | 15-19 | Caries of permanent teeth | 8,544,733.2 (5,574,117.3 to 12,042,880.0) | 30,466.6 (19,874.7 to 42,939.3) |
| Prevalence | Male | 15-19 | Periodontal diseases | 129,349.7 (76,422.4 to 217,027.6) | 461.2 (272.5 to 773.8) |
| Prevalence | Male | 15-19 | Edentulism | 0.0 (0.0 to 0.0) | 0.0 (0.0 to 0.0) |
| Prevalence | Male | 15-19 | Other oral disorders | 420,671.2 (365,246.6 to 482,650.7) | 1,499.9 (1,302.3 to 1,720.9) |
| Prevalence | Male | 20-24 | Main Oral disorders | 10,949,671.7 (8,402,587.8 to 14,018,711.2) | 39,332.7 (30,183.2 to 50,357.0) |
| Prevalence | Male | 20-24 | Caries of deciduous teeth | 0.0 (0.0 to 0.0) | 0.0 (0.0 to 0.0) |
| Prevalence | Male | 20-24 | Caries of permanent teeth | 10,318,879.2 (7,702,367.0 to 13,487,387.0) | 37,066.8 (27,667.9 to 48,448.5) |
| Prevalence | Male | 20-24 | Periodontal diseases | 476,488.3 (283,590.1 to 720,634.4) | 1,711.6 (1,018.7 to 2,588.6) |
| Prevalence | Male | 20-24 | Edentulism | 25,799.7 (14,063.9 to 40,546.8) | 92.7 (50.5 to 145.6) |
| Prevalence | Male | 20-24 | Other oral disorders | 511,001.8 (454,614.9 to 569,752.8) | 1,835.6 (1,633.0 to 2,046.6) |
| Prevalence | Male | 25-29 | Main Oral disorders | 11,614,960.3 (8,862,815.0 to 15,706,870.2) | 41,424.5 (31,609.0 to 56,018.2) |
| Prevalence | Male | 25-29 | Caries of deciduous teeth | 0.0 (0.0 to 0.0) | 0.0 (0.0 to 0.0) |
| Prevalence | Male | 25-29 | Caries of permanent teeth | 10,482,680.9 (7,569,115.9 to 14,966,374.9) | 37,386.2 (26,995.1 to 53,377.2) |
| Prevalence | Male | 25-29 | Periodontal diseases | 1,202,594.4 (764,529.8 to 1,762,834.5) | 4,289.0 (2,726.7 to 6,287.1) |
| Prevalence | Male | 25-29 | Edentulism | 73,560.3 (46,013.8 to 104,721.8) | 262.4 (164.1 to 373.5) |
| Prevalence | Male | 25-29 | Other oral disorders | 565,093.9 (500,810.1 to 632,591.2) | 2,015.4 (1,786.1 to 2,256.1) |
| Prevalence | Male | 30-34 | Main Oral disorders | 11,638,494.2 (9,241,083.7 to 15,327,890.2) | 42,535.6 (33,773.7 to 56,019.4) |
| Prevalence | Male | 30-34 | Caries of deciduous teeth | 0.0 (0.0 to 0.0) | 0.0 (0.0 to 0.0) |
| Prevalence | Male | 30-34 | Caries of permanent teeth | 9,824,590.6 (7,179,163.9 to 13,945,258.5) | 35,906.3 (26,237.9 to 50,966.2) |
| Prevalence | Male | 30-34 | Periodontal diseases | 2,186,994.5 (1,438,571.3 to 3,103,766.9) | 7,992.9 (5,257.6 to 11,343.4) |
| Prevalence | Male | 30-34 | Edentulism | 135,145.8 (92,398.8 to 187,088.9) | 493.9 (337.7 to 683.8) |
| Prevalence | Male | 30-34 | Other oral disorders | 583,815.9 (517,976.8 to 649,075.9) | 2,133.7 (1,893.1 to 2,372.2) |
| Prevalence | Male | 35-39 | Main Oral disorders | 12,059,977.7 (9,186,644.8 to 15,124,177.8) | 46,767.6 (35,625.0 to 58,650.3) |
| Prevalence | Male | 35-39 | Caries of deciduous teeth | 0.0 (0.0 to 0.0) | 0.0 (0.0 to 0.0) |
| Prevalence | Male | 35-39 | Caries of permanent teeth | 9,624,883.8 (6,358,386.5 to 13,313,003.6) | 37,324.5 (24,657.3 to 51,626.7) |
| Prevalence | Male | 35-39 | Periodontal diseases | 3,244,545.4 (2,214,276.0 to 4,434,010.2) | 12,582.1 (8,586.8 to 17,194.7) |
| Prevalence | Male | 35-39 | Edentulism | 205,587.9 (144,097.2 to 280,129.1) | 797.3 (558.8 to 1,086.3) |
| Prevalence | Male | 35-39 | Other oral disorders | 565,319.4 (502,065.7 to 633,600.6) | 2,192.3 (1,947.0 to 2,457.0) |
| Prevalence | Male | 40-44 | Main Oral disorders | 12,140,815.3 (9,612,062.7 to 14,894,034.5) | 50,443.3 (39,936.7 to 61,882.6) |
| Prevalence | Male | 40-44 | Caries of deciduous teeth | 0.0 (0.0 to 0.0) | 0.0 (0.0 to 0.0) |
| Prevalence | Male | 40-44 | Caries of permanent teeth | 9,104,814.7 (6,143,972.4 to 12,492,796.0) | 37,829.2 (25,527.3 to 51,905.8) |
| Prevalence | Male | 40-44 | Periodontal diseases | 4,235,541.0 (2,983,556.3 to 5,453,202.6) | 17,598.1 (12,396.2 to 22,657.3) |
| Prevalence | Male | 40-44 | Edentulism | 302,058.3 (218,541.2 to 399,443.9) | 1,255.0 (908.0 to 1,659.6) |
| Prevalence | Male | 40-44 | Other oral disorders | 540,001.5 (480,728.9 to 605,476.8) | 2,243.6 (1,997.4 to 2,515.7) |
| Prevalence | Male | 45-49 | Main Oral disorders | 11,508,447.6 (9,307,560.3 to 13,703,605.8) | 53,339.8 (43,139.1 to 63,514.1) |
| Prevalence | Male | 45-49 | Caries of deciduous teeth | 0.0 (0.0 to 0.0) | 0.0 (0.0 to 0.0) |
| Prevalence | Male | 45-49 | Caries of permanent teeth | 8,111,931.4 (5,457,787.4 to 10,862,847.9) | 37,597.5 (25,296.0 to 50,347.6) |
| Prevalence | Male | 45-49 | Periodontal diseases | 4,764,238.8 (3,514,117.1 to 5,956,204.4) | 22,081.5 (16,287.4 to 27,606.1) |
| Prevalence | Male | 45-49 | Edentulism | 441,991.2 (329,513.4 to 570,138.8) | 2,048.6 (1,527.2 to 2,642.5) |
| Prevalence | Male | 45-49 | Other oral disorders | 493,807.0 (437,606.0 to 560,273.9) | 2,288.7 (2,028.2 to 2,596.8) |
| Prevalence | Male | 50-54 | Main Oral disorders | 10,840,391.5 (8,858,777.7 to 12,929,937.1) | 56,035.8 (45,792.5 to 66,837.0) |
| Prevalence | Male | 50-54 | Caries of deciduous teeth | 0.0 (0.0 to 0.0) | 0.0 (0.0 to 0.0) |
| Prevalence | Male | 50-54 | Caries of permanent teeth | 7,297,790.5 (4,714,452.2 to 10,166,437.8) | 37,723.5 (24,369.8 to 52,552.0) |
| Prevalence | Male | 50-54 | Periodontal diseases | 4,898,860.7 (3,778,114.7 to 5,964,982.1) | 25,323.0 (19,529.7 to 30,834.0) |
| Prevalence | Male | 50-54 | Edentulism | 670,936.2 (516,320.2 to 863,597.3) | 3,468.2 (2,668.9 to 4,464.1) |
| Prevalence | Male | 50-54 | Other oral disorders | 444,388.8 (392,702.8 to 505,900.1) | 2,297.1 (2,029.9 to 2,615.1) |
| Prevalence | Male | 55-59 | Main Oral disorders | 9,626,277.5 (8,144,088.4 to 11,394,256.4) | 58,496.7 (49,489.8 to 69,240.3) |
| Prevalence | Male | 55-59 | Caries of deciduous teeth | 0.0 (0.0 to 0.0) | 0.0 (0.0 to 0.0) |
| Prevalence | Male | 55-59 | Caries of permanent teeth | 6,281,637.7 (4,103,670.3 to 8,769,966.8) | 38,172.1 (24,937.1 to 53,293.1) |
| Prevalence | Male | 55-59 | Periodontal diseases | 4,462,618.9 (3,570,798.2 to 5,288,719.2) | 27,118.3 (21,698.9 to 32,138.4) |
| Prevalence | Male | 55-59 | Edentulism | 968,620.0 (756,687.3 to 1,254,828.6) | 5,886.1 (4,598.2 to 7,625.3) |
| Prevalence | Male | 55-59 | Other oral disorders | 373,595.8 (327,750.1 to 424,057.6) | 2,270.3 (1,991.7 to 2,576.9) |
| Prevalence | Male | 60-64 | Main Oral disorders | 8,019,746.2 (7,047,990.7 to 9,150,945.6) | 60,567.4 (53,228.5 to 69,110.6) |
| Prevalence | Male | 60-64 | Caries of deciduous teeth | 0.0 (0.0 to 0.0) | 0.0 (0.0 to 0.0) |
| Prevalence | Male | 60-64 | Caries of permanent teeth | 5,114,515.6 (3,752,083.2 to 6,873,363.6) | 38,626.3 (28,336.8 to 51,909.6) |
| Prevalence | Male | 60-64 | Periodontal diseases | 3,615,232.0 (2,872,086.1 to 4,265,425.6) | 27,303.3 (21,690.8 to 32,213.7) |
| Prevalence | Male | 60-64 | Edentulism | 1,273,579.8 (993,603.7 to 1,638,094.1) | 9,618.4 (7,504.0 to 12,371.4) |
| Prevalence | Male | 60-64 | Other oral disorders | 297,369.6 (263,638.3 to 337,739.4) | 2,245.8 (1,991.1 to 2,550.7) |
| Prevalence | Male | 65-69 | Main Oral disorders | 5,937,889.4 (5,321,751.6 to 6,580,954.9) | 62,534.5 (56,045.7 to 69,306.9) |
| Prevalence | Male | 65-69 | Caries of deciduous teeth | 0.0 (0.0 to 0.0) | 0.0 (0.0 to 0.0) |
| Prevalence | Male | 65-69 | Caries of permanent teeth | 3,680,684.7 (2,725,497.7 to 4,660,099.8) | 38,762.9 (28,703.4 to 49,077.6) |
| Prevalence | Male | 65-69 | Periodontal diseases | 2,543,520.8 (2,067,330.0 to 3,000,631.3) | 26,786.9 (21,772.0 to 31,601.0) |
| Prevalence | Male | 65-69 | Edentulism | 1,377,287.7 (1,077,825.8 to 1,736,908.4) | 14,504.8 (11,351.1 to 18,292.1) |
| Prevalence | Male | 65-69 | Other oral disorders | 211,130.9 (186,261.3 to 234,428.4) | 2,223.5 (1,961.6 to 2,468.9) |
| Prevalence | Male | 70-74 | Main Oral disorders | 3,812,849.8 (3,386,338.9 to 4,253,159.3) | 64,392.3 (57,189.3 to 71,828.3) |
| Prevalence | Male | 70-74 | Caries of deciduous teeth | 0.0 (0.0 to 0.0) | 0.0 (0.0 to 0.0) |
| Prevalence | Male | 70-74 | Caries of permanent teeth | 2,291,966.7 (1,577,244.6 to 2,973,930.7) | 38,707.3 (26,636.9 to 50,224.4) |
| Prevalence | Male | 70-74 | Periodontal diseases | 1,530,554.6 (1,245,514.2 to 1,829,852.0) | 25,848.4 (21,034.5 to 30,903.0) |
| Prevalence | Male | 70-74 | Edentulism | 1,176,960.4 (933,144.4 to 1,510,205.9) | 19,876.8 (15,759.2 to 25,504.7) |
| Prevalence | Male | 70-74 | Other oral disorders | 123,492.4 (107,852.6 to 138,204.1) | 2,085.6 (1,821.4 to 2,334.0) |
| Prevalence | Male | 75-79 | Main Oral disorders | 2,178,156.9 (1,972,713.9 to 2,373,187.8) | 64,584.6 (58,493.0 to 70,367.5) |
| Prevalence | Male | 75-79 | Caries of deciduous teeth | 0.0 (0.0 to 0.0) | 0.0 (0.0 to 0.0) |
| Prevalence | Male | 75-79 | Caries of permanent teeth | 1,216,136.2 (906,240.0 to 1,569,420.6) | 36,059.7 (26,871.0 to 46,535.0) |
| Prevalence | Male | 75-79 | Periodontal diseases | 844,215.7 (682,593.7 to 1,003,466.8) | 25,031.9 (20,239.6 to 29,753.8) |
| Prevalence | Male | 75-79 | Edentulism | 829,065.2 (668,617.2 to 1,023,150.5) | 24,582.7 (19,825.2 to 30,337.5) |
| Prevalence | Male | 75-79 | Other oral disorders | 61,796.4 (55,046.4 to 69,007.1) | 1,832.3 (1,632.2 to 2,046.1) |
| Prevalence | Male | 80-84 | Main Oral disorders | 1,184,011.8 (1,068,428.6 to 1,301,240.6) | 64,531.5 (58,231.9 to 70,920.7) |
| Prevalence | Male | 80-84 | Caries of deciduous teeth | 0.0 (0.0 to 0.0) | 0.0 (0.0 to 0.0) |
| Prevalence | Male | 80-84 | Caries of permanent teeth | 609,675.4 (417,155.8 to 829,479.0) | 33,228.8 (22,736.0 to 45,208.6) |
| Prevalence | Male | 80-84 | Periodontal diseases | 441,784.0 (355,302.4 to 529,919.2) | 24,078.3 (19,364.8 to 28,881.9) |
| Prevalence | Male | 80-84 | Edentulism | 528,304.4 (427,299.3 to 635,719.8) | 28,793.8 (23,288.8 to 34,648.2) |
| Prevalence | Male | 80-84 | Other oral disorders | 27,934.5 (24,498.9 to 31,444.9) | 1,522.5 (1,335.3 to 1,713.8) |
| Prevalence | Male | 85-89 | Main Oral disorders | 526,827.4 (472,508.8 to 584,587.1) | 63,608.5 (57,050.1 to 70,582.3) |
| Prevalence | Male | 85-89 | Caries of deciduous teeth | 0.0 (0.0 to 0.0) | 0.0 (0.0 to 0.0) |
| Prevalence | Male | 85-89 | Caries of permanent teeth | 240,253.6 (157,237.2 to 339,333.0) | 29,007.9 (18,984.6 to 40,970.6) |
| Prevalence | Male | 85-89 | Periodontal diseases | 191,294.9 (151,561.9 to 235,031.3) | 23,096.7 (18,299.4 to 28,377.4) |
| Prevalence | Male | 85-89 | Edentulism | 268,320.3 (215,189.0 to 320,999.0) | 32,396.7 (25,981.7 to 38,757.0) |
| Prevalence | Male | 85-89 | Other oral disorders | 9,545.7 (8,479.0 to 10,736.6) | 1,152.5 (1,023.7 to 1,296.3) |
| Prevalence | Male | 90-94 | Main Oral disorders | 182,587.5 (162,048.2 to 202,445.5) | 62,546.5 (55,510.6 to 69,348.9) |
| Prevalence | Male | 90-94 | Caries of deciduous teeth | 0.0 (0.0 to 0.0) | 0.0 (0.0 to 0.0) |
| Prevalence | Male | 90-94 | Caries of permanent teeth | 70,479.5 (40,649.8 to 107,967.3) | 24,143.2 (13,924.8 to 36,984.9) |
| Prevalence | Male | 90-94 | Periodontal diseases | 65,266.6 (50,807.0 to 81,317.3) | 22,357.5 (17,404.2 to 27,855.7) |
| Prevalence | Male | 90-94 | Edentulism | 103,696.3 (83,667.5 to 125,901.7) | 35,521.8 (28,660.8 to 43,128.4) |
| Prevalence | Male | 90-94 | Other oral disorders | 2,864.4 (2,485.3 to 3,258.8) | 981.2 (851.4 to 1,116.3) |
| Prevalence | Male | 95+ | Main Oral disorders | 53,743.1 (48,076.3 to 59,981.5) | 61,072.7 (54,633.1 to 68,162.0) |
| Prevalence | Male | 95+ | Caries of deciduous teeth | 0.0 (0.0 to 0.0) | 0.0 (0.0 to 0.0) |
| Prevalence | Male | 95+ | Caries of permanent teeth | 15,635.3 (8,761.6 to 24,161.7) | 17,767.7 (9,956.5 to 27,456.9) |
| Prevalence | Male | 95+ | Periodontal diseases | 18,670.6 (13,658.3 to 24,074.0) | 21,216.9 (15,521.1 to 27,357.3) |
| Prevalence | Male | 95+ | Edentulism | 34,223.2 (27,238.3 to 41,593.7) | 38,890.6 (30,953.1 to 47,266.4) |
| Prevalence | Male | 95+ | Other oral disorders | 890.1 (746.8 to 1,033.0) | 1,011.5 (848.6 to 1,173.9) |
| Incidence | Female | <5 | Main Oral disorders | 12,778,097.9 (9,205,903.5 to 15,971,161.6) | 48,210.3 (34,732.8 to 60,257.4) |
| Incidence | Female | <5 | Caries of deciduous teeth | 12,778,097.9 (9,205,903.5 to 15,971,161.6) | 48,210.3 (34,732.8 to 60,257.4) |
| Incidence | Female | <5 | Caries of permanent teeth | 0.0 (0.0 to 0.0) | 0.0 (0.0 to 0.0) |
| Incidence | Female | <5 | Periodontal diseases | 0.0 (0.0 to 0.0) | 0.0 (0.0 to 0.0) |
| Incidence | Female | <5 | Edentulism | 0.0 (0.0 to 0.0) | 0.0 (0.0 to 0.0) |
| Incidence | Female | 5-9 | Main Oral disorders | 41,186,228.8 (29,535,705.7 to 51,926,807.4) | 150,044.2 (107,600.5 to 189,172.8) |
| Incidence | Female | 5-9 | Caries of deciduous teeth | 34,974,743.7 (24,349,961.7 to 44,818,046.8) | 127,415.3 (88,708.5 to 163,275.1) |
| Incidence | Female | 5-9 | Caries of permanent teeth | 6,211,485.1 (2,839,687.1 to 10,648,979.7) | 22,628.9 (10,345.2 to 38,794.9) |
| Incidence | Female | 5-9 | Periodontal diseases | 0.0 (0.0 to 0.0) | 0.0 (0.0 to 0.0) |
| Incidence | Female | 5-9 | Edentulism | 0.0 (0.0 to 0.0) | 0.0 (0.0 to 0.0) |
| Incidence | Female | 10-14 | Main Oral disorders | 21,102,029.1 (11,448,248.0 to 39,977,017.7) | 77,410.8 (41,996.8 to 146,652.0) |
| Incidence | Female | 10-14 | Caries of deciduous teeth | 10,019,052.3 (1,755,789.4 to 28,908,788.7) | 36,754.0 (6,441.0 to 106,049.2) |
| Incidence | Female | 10-14 | Caries of permanent teeth | 11,082,976.8 (7,517,470.3 to 14,960,310.2) | 40,656.9 (27,577.1 to 54,880.5) |
| Incidence | Female | 10-14 | Periodontal diseases | 0.0 (0.0 to 0.0) | 0.0 (0.0 to 0.0) |
| Incidence | Female | 10-14 | Edentulism | 0.0 (0.0 to 0.0) | 0.0 (0.0 to 0.0) |
| Incidence | Female | 15-19 | Main Oral disorders | 12,950,436.7 (9,970,903.8 to 16,075,856.5) | 48,556.2 (37,384.8 to 60,274.6) |
| Incidence | Female | 15-19 | Caries of deciduous teeth | 0.0 (0.0 to 0.0) | 0.0 (0.0 to 0.0) |
| Incidence | Female | 15-19 | Caries of permanent teeth | 12,897,896.4 (9,936,016.0 to 16,036,064.3) | 48,359.2 (37,253.9 to 60,125.4) |
| Incidence | Female | 15-19 | Periodontal diseases | 52,540.3 (28,513.4 to 92,247.0) | 197.0 (106.9 to 345.9) |
| Incidence | Female | 15-19 | Edentulism | 0.0 (0.0 to 0.0) | 0.0 (0.0 to 0.0) |
| Incidence | Female | 20-24 | Main Oral disorders | 15,493,967.0 (12,555,831.1 to 17,988,539.9) | 57,812.1 (46,849.2 to 67,120.0) |
| Incidence | Female | 20-24 | Caries of deciduous teeth | 0.0 (0.0 to 0.0) | 0.0 (0.0 to 0.0) |
| Incidence | Female | 20-24 | Caries of permanent teeth | 15,326,090.2 (12,375,198.9 to 17,808,249.1) | 57,185.7 (46,175.2 to 66,447.3) |
| Incidence | Female | 20-24 | Periodontal diseases | 153,220.3 (90,423.0 to 240,190.3) | 571.7 (337.4 to 896.2) |
| Incidence | Female | 20-24 | Edentulism | 14,656.5 (8,980.3 to 21,091.0) | 54.7 (33.5 to 78.7) |
| Incidence | Female | 25-29 | Main Oral disorders | 14,691,698.6 (11,265,121.1 to 17,766,742.7) | 53,824.8 (41,271.2 to 65,090.7) |
| Incidence | Female | 25-29 | Caries of deciduous teeth | 0.0 (0.0 to 0.0) | 0.0 (0.0 to 0.0) |
| Incidence | Female | 25-29 | Caries of permanent teeth | 14,396,469.9 (11,025,096.9 to 17,548,616.9) | 52,743.2 (40,391.8 to 64,291.5) |
| Incidence | Female | 25-29 | Periodontal diseases | 269,464.3 (151,419.5 to 415,204.5) | 987.2 (554.7 to 1,521.2) |
| Incidence | Female | 25-29 | Edentulism | 25,764.4 (17,235.7 to 37,355.4) | 94.4 (63.1 to 136.9) |
| Incidence | Female | 30-34 | Main Oral disorders | 13,006,921.7 (10,136,046.5 to 15,448,752.7) | 48,905.3 (38,111.0 to 58,086.5) |
| Incidence | Female | 30-34 | Caries of deciduous teeth | 0.0 (0.0 to 0.0) | 0.0 (0.0 to 0.0) |
| Incidence | Female | 30-34 | Caries of permanent teeth | 12,639,228.7 (9,746,418.7 to 15,038,637.2) | 47,522.8 (36,646.0 to 56,544.5) |
| Incidence | Female | 30-34 | Periodontal diseases | 333,906.8 (207,720.2 to 474,616.2) | 1,255.5 (781.0 to 1,784.5) |
| Incidence | Female | 30-34 | Edentulism | 33,786.2 (21,572.2 to 50,002.6) | 127.0 (81.1 to 188.0) |
| Incidence | Female | 35-39 | Main Oral disorders | 11,239,066.9 (8,655,815.6 to 13,476,284.8) | 44,525.5 (34,291.5 to 53,388.7) |
| Incidence | Female | 35-39 | Caries of deciduous teeth | 0.0 (0.0 to 0.0) | 0.0 (0.0 to 0.0) |
| Incidence | Female | 35-39 | Caries of permanent teeth | 10,827,263.8 (8,312,812.8 to 13,070,035.1) | 42,894.1 (32,932.7 to 51,779.2) |
| Incidence | Female | 35-39 | Periodontal diseases | 367,702.7 (227,869.5 to 517,232.7) | 1,456.7 (902.7 to 2,049.1) |
| Incidence | Female | 35-39 | Edentulism | 44,100.4 (27,077.3 to 66,244.2) | 174.7 (107.3 to 262.4) |
| Incidence | Female | 40-44 | Main Oral disorders | 9,786,745.3 (7,572,354.5 to 12,206,395.9) | 41,126.2 (31,820.8 to 51,294.2) |
| Incidence | Female | 40-44 | Caries of deciduous teeth | 0.0 (0.0 to 0.0) | 0.0 (0.0 to 0.0) |
| Incidence | Female | 40-44 | Caries of permanent teeth | 9,339,062.7 (7,014,764.5 to 11,705,305.0) | 39,245.0 (29,477.7 to 49,188.5) |
| Incidence | Female | 40-44 | Periodontal diseases | 382,224.8 (230,537.2 to 515,636.7) | 1,606.2 (968.8 to 2,166.8) |
| Incidence | Female | 40-44 | Edentulism | 65,457.7 (42,117.4 to 98,518.3) | 275.1 (177.0 to 414.0) |
| Incidence | Female | 45-49 | Main Oral disorders | 8,255,649.8 (6,605,051.1 to 10,242,059.7) | 38,088.6 (30,473.3 to 47,253.1) |
| Incidence | Female | 45-49 | Caries of deciduous teeth | 0.0 (0.0 to 0.0) | 0.0 (0.0 to 0.0) |
| Incidence | Female | 45-49 | Caries of permanent teeth | 7,789,705.4 (6,112,359.6 to 9,767,777.5) | 35,938.9 (28,200.2 to 45,065.0) |
| Incidence | Female | 45-49 | Periodontal diseases | 371,268.3 (244,323.3 to 488,400.6) | 1,712.9 (1,127.2 to 2,253.3) |
| Incidence | Female | 45-49 | Edentulism | 94,676.1 (61,166.4 to 138,926.8) | 436.8 (282.2 to 641.0) |
| Incidence | Female | 50-54 | Main Oral disorders | 6,908,474.0 (5,397,457.4 to 8,882,522.2) | 34,816.6 (27,201.5 to 44,765.2) |
| Incidence | Female | 50-54 | Caries of deciduous teeth | 0.0 (0.0 to 0.0) | 0.0 (0.0 to 0.0) |
| Incidence | Female | 50-54 | Caries of permanent teeth | 6,415,981.4 (4,866,871.7 to 8,419,190.6) | 32,334.6 (24,527.6 to 42,430.2) |
| Incidence | Female | 50-54 | Periodontal diseases | 357,949.9 (252,066.6 to 473,791.3) | 1,804.0 (1,270.3 to 2,387.8) |
| Incidence | Female | 50-54 | Edentulism | 134,542.7 (90,924.2 to 196,810.7) | 678.1 (458.2 to 991.9) |
| Incidence | Female | 55-59 | Main Oral disorders | 5,468,287.3 (4,209,215.8 to 6,855,362.4) | 31,494.7 (24,243.1 to 39,483.6) |
| Incidence | Female | 55-59 | Caries of deciduous teeth | 0.0 (0.0 to 0.0) | 0.0 (0.0 to 0.0) |
| Incidence | Female | 55-59 | Caries of permanent teeth | 4,970,744.3 (3,692,791.4 to 6,380,366.2) | 28,629.1 (21,268.7 to 36,747.8) |
| Incidence | Female | 55-59 | Periodontal diseases | 319,751.4 (217,568.1 to 420,819.1) | 1,841.6 (1,253.1 to 2,423.7) |
| Incidence | Female | 55-59 | Edentulism | 177,791.6 (120,934.1 to 255,405.4) | 1,024.0 (696.5 to 1,471.0) |
| Incidence | Female | 60-64 | Main Oral disorders | 4,151,714.3 (3,228,405.1 to 5,235,431.4) | 28,864.2 (22,445.0 to 36,398.6) |
| Incidence | Female | 60-64 | Caries of deciduous teeth | 0.0 (0.0 to 0.0) | 0.0 (0.0 to 0.0) |
| Incidence | Female | 60-64 | Caries of permanent teeth | 3,683,638.2 (2,739,423.7 to 4,757,362.4) | 25,610.0 (19,045.4 to 33,074.9) |
| Incidence | Female | 60-64 | Periodontal diseases | 268,552.1 (187,335.4 to 349,856.8) | 1,867.1 (1,302.4 to 2,432.3) |
| Incidence | Female | 60-64 | Edentulism | 199,524.0 (137,106.9 to 271,642.5) | 1,387.2 (953.2 to 1,888.6) |
| Incidence | Female | 65-69 | Main Oral disorders | 2,935,320.8 (2,353,870.4 to 3,659,640.9) | 27,137.2 (21,761.7 to 33,833.6) |
| Incidence | Female | 65-69 | Caries of deciduous teeth | 0.0 (0.0 to 0.0) | 0.0 (0.0 to 0.0) |
| Incidence | Female | 65-69 | Caries of permanent teeth | 2,554,877.3 (1,964,652.3 to 3,278,222.0) | 23,620.0 (18,163.3 to 30,307.3) |
| Incidence | Female | 65-69 | Periodontal diseases | 205,952.3 (138,493.5 to 268,945.4) | 1,904.0 (1,280.4 to 2,486.4) |
| Incidence | Female | 65-69 | Edentulism | 174,491.1 (113,429.0 to 239,070.1) | 1,613.2 (1,048.7 to 2,210.2) |
| Incidence | Female | 70-74 | Main Oral disorders | 1,790,335.7 (1,383,389.0 to 2,322,966.3) | 25,240.3 (19,503.1 to 32,749.4) |
| Incidence | Female | 70-74 | Caries of deciduous teeth | 0.0 (0.0 to 0.0) | 0.0 (0.0 to 0.0) |
| Incidence | Female | 70-74 | Caries of permanent teeth | 1,536,943.2 (1,147,961.0 to 2,063,100.7) | 21,667.9 (16,184.0 to 29,085.8) |
| Incidence | Female | 70-74 | Periodontal diseases | 136,823.8 (91,746.6 to 177,405.1) | 1,929.0 (1,293.5 to 2,501.1) |
| Incidence | Female | 70-74 | Edentulism | 116,568.6 (75,752.8 to 161,265.3) | 1,643.4 (1,068.0 to 2,273.5) |
| Incidence | Female | 75-79 | Main Oral disorders | 1,080,467.3 (851,409.7 to 1,371,700.5) | 23,929.2 (18,856.3 to 30,379.2) |
| Incidence | Female | 75-79 | Caries of deciduous teeth | 0.0 (0.0 to 0.0) | 0.0 (0.0 to 0.0) |
| Incidence | Female | 75-79 | Caries of permanent teeth | 921,596.2 (691,813.5 to 1,204,381.6) | 20,410.7 (15,321.7 to 26,673.6) |
| Incidence | Female | 75-79 | Periodontal diseases | 87,828.2 (59,424.8 to 110,889.2) | 1,945.1 (1,316.1 to 2,455.9) |
| Incidence | Female | 75-79 | Edentulism | 71,042.9 (45,293.7 to 102,078.5) | 1,573.4 (1,003.1 to 2,260.7) |
| Incidence | Female | 80-84 | Main Oral disorders | 624,782.2 (488,002.5 to 780,918.1) | 22,238.2 (17,369.7 to 27,795.6) |
| Incidence | Female | 80-84 | Caries of deciduous teeth | 0.0 (0.0 to 0.0) | 0.0 (0.0 to 0.0) |
| Incidence | Female | 80-84 | Caries of permanent teeth | 530,245.9 (394,824.1 to 682,049.8) | 18,873.3 (14,053.2 to 24,276.5) |
| Incidence | Female | 80-84 | Periodontal diseases | 54,241.4 (36,612.8 to 71,229.8) | 1,930.6 (1,303.2 to 2,535.3) |
| Incidence | Female | 80-84 | Edentulism | 40,294.8 (25,517.4 to 57,052.8) | 1,434.2 (908.3 to 2,030.7) |
| Incidence | Female | 85-89 | Main Oral disorders | 290,327.1 (222,540.6 to 371,139.2) | 20,437.0 (15,665.3 to 26,125.6) |
| Incidence | Female | 85-89 | Caries of deciduous teeth | 0.0 (0.0 to 0.0) | 0.0 (0.0 to 0.0) |
| Incidence | Female | 85-89 | Caries of permanent teeth | 245,519.2 (181,671.4 to 323,714.9) | 17,282.8 (12,788.4 to 22,787.3) |
| Incidence | Female | 85-89 | Periodontal diseases | 27,675.8 (16,672.7 to 36,583.5) | 1,948.2 (1,173.6 to 2,575.2) |
| Incidence | Female | 85-89 | Edentulism | 17,132.1 (11,422.5 to 25,790.0) | 1,206.0 (804.1 to 1,815.4) |
| Incidence | Female | 90-94 | Main Oral disorders | 95,998.5 (71,347.3 to 128,117.7) | 18,319.5 (13,615.3 to 24,448.8) |
| Incidence | Female | 90-94 | Caries of deciduous teeth | 0.0 (0.0 to 0.0) | 0.0 (0.0 to 0.0) |
| Incidence | Female | 90-94 | Caries of permanent teeth | 80,231.9 (55,699.9 to 111,491.4) | 15,310.7 (10,629.3 to 21,276.0) |
| Incidence | Female | 90-94 | Periodontal diseases | 10,210.8 (5,399.2 to 13,574.3) | 1,948.5 (1,030.3 to 2,590.4) |
| Incidence | Female | 90-94 | Edentulism | 5,555.7 (3,312.9 to 8,612.6) | 1,060.2 (632.2 to 1,643.6) |
| Incidence | Female | 95+ | Main Oral disorders | 23,184.8 (13,759.7 to 34,275.9) | 15,754.4 (9,349.9 to 23,290.9) |
| Incidence | Female | 95+ | Caries of deciduous teeth | 0.0 (0.0 to 0.0) | 0.0 (0.0 to 0.0) |
| Incidence | Female | 95+ | Caries of permanent teeth | 18,941.0 (9,390.8 to 29,597.7) | 12,870.7 (6,381.1 to 20,112.1) |
| Incidence | Female | 95+ | Periodontal diseases | 2,782.0 (1,554.6 to 3,789.4) | 1,890.4 (1,056.4 to 2,574.9) |
| Incidence | Female | 95+ | Edentulism | 1,461.7 (738.0 to 2,691.3) | 993.3 (501.5 to 1,828.8) |
| Incidence | Male | <5 | Main Oral disorders | 13,503,743.3 (9,693,717.0 to 16,927,758.4) | 47,947.5 (34,419.3 to 60,105.1) |
| Incidence | Male | <5 | Caries of deciduous teeth | 13,503,743.3 (9,693,717.0 to 16,927,758.4) | 47,947.5 (34,419.3 to 60,105.1) |
| Incidence | Male | <5 | Caries of permanent teeth | 0.0 (0.0 to 0.0) | 0.0 (0.0 to 0.0) |
| Incidence | Male | <5 | Periodontal diseases | 0.0 (0.0 to 0.0) | 0.0 (0.0 to 0.0) |
| Incidence | Male | <5 | Edentulism | 0.0 (0.0 to 0.0) | 0.0 (0.0 to 0.0) |
| Incidence | Male | 5-9 | Main Oral disorders | 43,451,236.2 (31,065,854.2 to 54,738,611.9) | 149,372.6 (106,795.3 to 188,175.3) |
| Incidence | Male | 5-9 | Caries of deciduous teeth | 36,989,974.9 (25,628,582.6 to 47,523,367.6) | 127,160.7 (88,103.5 to 163,371.4) |
| Incidence | Male | 5-9 | Caries of permanent teeth | 6,461,261.4 (2,883,984.7 to 11,144,087.3) | 22,211.9 (9,914.3 to 38,310.1) |
| Incidence | Male | 5-9 | Periodontal diseases | 0.0 (0.0 to 0.0) | 0.0 (0.0 to 0.0) |
| Incidence | Male | 5-9 | Edentulism | 0.0 (0.0 to 0.0) | 0.0 (0.0 to 0.0) |
| Incidence | Male | 10-14 | Main Oral disorders | 22,459,808.3 (12,033,202.9 to 42,733,586.5) | 77,723.7 (41,641.7 to 147,882.4) |
| Incidence | Male | 10-14 | Caries of deciduous teeth | 10,729,911.2 (1,900,146.6 to 31,305,581.1) | 37,131.6 (6,575.6 to 108,335.1) |
| Incidence | Male | 10-14 | Caries of permanent teeth | 11,729,897.1 (7,898,139.7 to 15,766,733.6) | 40,592.1 (27,332.0 to 54,561.8) |
| Incidence | Male | 10-14 | Periodontal diseases | 0.0 (0.0 to 0.0) | 0.0 (0.0 to 0.0) |
| Incidence | Male | 10-14 | Edentulism | 0.0 (0.0 to 0.0) | 0.0 (0.0 to 0.0) |
| Incidence | Male | 15-19 | Main Oral disorders | 13,669,182.3 (10,491,318.4 to 16,839,924.5) | 48,738.0 (37,407.2 to 60,043.4) |
| Incidence | Male | 15-19 | Caries of deciduous teeth | 0.0 (0.0 to 0.0) | 0.0 (0.0 to 0.0) |
| Incidence | Male | 15-19 | Caries of permanent teeth | 13,626,023.7 (10,456,160.8 to 16,798,475.0) | 48,584.1 (37,281.8 to 59,895.6) |
| Incidence | Male | 15-19 | Periodontal diseases | 43,158.6 (24,289.6 to 72,989.8) | 153.9 (86.6 to 260.2) |
| Incidence | Male | 15-19 | Edentulism | 0.0 (0.0 to 0.0) | 0.0 (0.0 to 0.0) |
| Incidence | Male | 20-24 | Main Oral disorders | 16,088,186.5 (13,123,655.1 to 18,668,381.0) | 57,790.9 (47,141.9 to 67,059.3) |
| Incidence | Male | 20-24 | Caries of deciduous teeth | 0.0 (0.0 to 0.0) | 0.0 (0.0 to 0.0) |
| Incidence | Male | 20-24 | Caries of permanent teeth | 15,959,397.3 (13,008,955.1 to 18,522,942.0) | 57,328.2 (46,729.9 to 66,536.8) |
| Incidence | Male | 20-24 | Periodontal diseases | 121,724.2 (69,835.5 to 191,153.3) | 437.2 (250.9 to 686.6) |
| Incidence | Male | 20-24 | Edentulism | 7,065.0 (4,153.8 to 10,412.5) | 25.4 (14.9 to 37.4) |
| Incidence | Male | 25-29 | Main Oral disorders | 15,038,591.8 (11,498,999.8 to 18,075,977.9) | 53,634.8 (41,010.9 to 64,467.5) |
| Incidence | Male | 25-29 | Caries of deciduous teeth | 0.0 (0.0 to 0.0) | 0.0 (0.0 to 0.0) |
| Incidence | Male | 25-29 | Caries of permanent teeth | 14,804,371.1 (11,232,695.9 to 17,812,498.3) | 52,799.4 (40,061.1 to 63,527.8) |
| Incidence | Male | 25-29 | Periodontal diseases | 222,685.2 (124,737.0 to 351,099.8) | 794.2 (444.9 to 1,252.2) |
| Incidence | Male | 25-29 | Edentulism | 11,535.5 (7,156.1 to 17,169.4) | 41.1 (25.5 to 61.2) |
| Incidence | Male | 30-34 | Main Oral disorders | 13,367,437.8 (10,232,005.6 to 15,796,445.5) | 48,854.4 (37,395.3 to 57,731.8) |
| Incidence | Male | 30-34 | Caries of deciduous teeth | 0.0 (0.0 to 0.0) | 0.0 (0.0 to 0.0) |
| Incidence | Male | 30-34 | Caries of permanent teeth | 13,053,547.5 (9,953,875.3 to 15,401,898.1) | 47,707.2 (36,378.8 to 56,289.8) |
| Incidence | Male | 30-34 | Periodontal diseases | 299,341.0 (180,153.8 to 453,600.4) | 1,094.0 (658.4 to 1,657.8) |
| Incidence | Male | 30-34 | Edentulism | 14,549.3 (8,877.6 to 21,649.9) | 53.2 (32.4 to 79.1) |
| Incidence | Male | 35-39 | Main Oral disorders | 11,486,379.1 (8,848,038.9 to 13,766,718.9) | 44,543.2 (34,311.9 to 53,386.2) |
| Incidence | Male | 35-39 | Caries of deciduous teeth | 0.0 (0.0 to 0.0) | 0.0 (0.0 to 0.0) |
| Incidence | Male | 35-39 | Caries of permanent teeth | 11,113,214.4 (8,501,214.1 to 13,392,379.3) | 43,096.1 (32,967.0 to 51,934.5) |
| Incidence | Male | 35-39 | Periodontal diseases | 354,419.2 (212,554.9 to 509,957.2) | 1,374.4 (824.3 to 1,977.6) |
| Incidence | Male | 35-39 | Edentulism | 18,745.4 (11,149.7 to 28,708.8) | 72.7 (43.2 to 111.3) |
| Incidence | Male | 40-44 | Main Oral disorders | 9,904,532.0 (7,651,740.8 to 12,380,255.8) | 41,151.9 (31,791.9 to 51,438.2) |
| Incidence | Male | 40-44 | Caries of deciduous teeth | 0.0 (0.0 to 0.0) | 0.0 (0.0 to 0.0) |
| Incidence | Male | 40-44 | Caries of permanent teeth | 9,494,849.2 (7,236,206.2 to 11,929,071.4) | 39,449.7 (30,065.4 to 49,563.6) |
| Incidence | Male | 40-44 | Periodontal diseases | 380,610.9 (232,776.7 to 523,720.3) | 1,581.4 (967.2 to 2,176.0) |
| Incidence | Male | 40-44 | Edentulism | 29,071.9 (18,208.4 to 44,533.9) | 120.8 (75.7 to 185.0) |
| Incidence | Male | 45-49 | Main Oral disorders | 8,214,355.0 (6,515,320.3 to 10,112,863.5) | 38,072.2 (30,197.5 to 46,871.5) |
| Incidence | Male | 45-49 | Caries of deciduous teeth | 0.0 (0.0 to 0.0) | 0.0 (0.0 to 0.0) |
| Incidence | Male | 45-49 | Caries of permanent teeth | 7,798,869.8 (6,072,067.9 to 9,703,765.6) | 36,146.5 (28,143.1 to 44,975.4) |
| Incidence | Male | 45-49 | Periodontal diseases | 368,126.9 (234,939.6 to 490,601.5) | 1,706.2 (1,088.9 to 2,273.9) |
| Incidence | Male | 45-49 | Edentulism | 47,358.3 (30,225.5 to 71,547.3) | 219.5 (140.1 to 331.6) |
| Incidence | Male | 50-54 | Main Oral disorders | 6,725,261.2 (5,154,809.5 to 8,550,980.8) | 34,764.0 (26,646.1 to 44,201.4) |
| Incidence | Male | 50-54 | Caries of deciduous teeth | 0.0 (0.0 to 0.0) | 0.0 (0.0 to 0.0) |
| Incidence | Male | 50-54 | Caries of permanent teeth | 6,298,971.7 (4,693,935.3 to 8,114,078.8) | 32,560.4 (24,263.7 to 41,943.0) |
| Incidence | Male | 50-54 | Periodontal diseases | 347,946.0 (243,089.1 to 458,141.2) | 1,798.6 (1,256.6 to 2,368.2) |
| Incidence | Male | 50-54 | Edentulism | 78,343.5 (53,337.8 to 112,578.2) | 405.0 (275.7 to 581.9) |
| Incidence | Male | 55-59 | Main Oral disorders | 5,154,516.3 (4,015,808.5 to 6,489,852.9) | 31,322.8 (24,403.2 to 39,437.4) |
| Incidence | Male | 55-59 | Caries of deciduous teeth | 0.0 (0.0 to 0.0) | 0.0 (0.0 to 0.0) |
| Incidence | Male | 55-59 | Caries of permanent teeth | 4,738,385.0 (3,565,970.0 to 6,100,908.5) | 28,794.1 (21,669.6 to 37,073.8) |
| Incidence | Male | 55-59 | Periodontal diseases | 301,068.0 (211,378.3 to 391,187.4) | 1,829.5 (1,284.5 to 2,377.2) |
| Incidence | Male | 55-59 | Edentulism | 115,063.3 (77,747.4 to 165,456.6) | 699.2 (472.5 to 1,005.4) |
| Incidence | Male | 60-64 | Main Oral disorders | 3,788,741.8 (2,918,632.5 to 4,772,519.2) | 28,613.7 (22,042.4 to 36,043.4) |
| Incidence | Male | 60-64 | Caries of deciduous teeth | 0.0 (0.0 to 0.0) | 0.0 (0.0 to 0.0) |
| Incidence | Male | 60-64 | Caries of permanent teeth | 3,407,105.2 (2,564,714.7 to 4,329,773.0) | 25,731.4 (19,369.5 to 32,699.7) |
| Incidence | Male | 60-64 | Periodontal diseases | 244,139.7 (166,617.7 to 319,023.6) | 1,843.8 (1,258.3 to 2,409.4) |
| Incidence | Male | 60-64 | Edentulism | 137,497.0 (93,759.0 to 188,565.6) | 1,038.4 (708.1 to 1,424.1) |
| Incidence | Male | 65-69 | Main Oral disorders | 2,557,995.6 (2,027,047.9 to 3,194,387.4) | 26,939.4 (21,347.7 to 33,641.5) |
| Incidence | Male | 65-69 | Caries of deciduous teeth | 0.0 (0.0 to 0.0) | 0.0 (0.0 to 0.0) |
| Incidence | Male | 65-69 | Caries of permanent teeth | 2,255,804.2 (1,752,250.7 to 2,897,184.6) | 23,756.9 (18,453.7 to 30,511.5) |
| Incidence | Male | 65-69 | Periodontal diseases | 178,212.8 (120,416.8 to 231,530.6) | 1,876.8 (1,268.2 to 2,438.4) |
| Incidence | Male | 65-69 | Edentulism | 123,978.5 (79,802.1 to 170,764.0) | 1,305.7 (840.4 to 1,798.4) |
| Incidence | Male | 70-74 | Main Oral disorders | 1,486,383.0 (1,144,449.3 to 1,925,815.9) | 25,102.4 (19,327.7 to 32,523.6) |
| Incidence | Male | 70-74 | Caries of deciduous teeth | 0.0 (0.0 to 0.0) | 0.0 (0.0 to 0.0) |
| Incidence | Male | 70-74 | Caries of permanent teeth | 1,288,863.5 (951,232.1 to 1,713,836.9) | 21,766.6 (16,064.6 to 28,943.7) |
| Incidence | Male | 70-74 | Periodontal diseases | 112,416.7 (76,523.1 to 145,377.3) | 1,898.5 (1,292.3 to 2,455.2) |
| Incidence | Male | 70-74 | Edentulism | 85,102.7 (57,526.3 to 118,756.3) | 1,437.2 (971.5 to 2,005.6) |
| Incidence | Male | 75-79 | Main Oral disorders | 805,646.3 (630,922.6 to 1,015,670.8) | 23,888.3 (18,707.5 to 30,115.7) |
| Incidence | Male | 75-79 | Caries of deciduous teeth | 0.0 (0.0 to 0.0) | 0.0 (0.0 to 0.0) |
| Incidence | Male | 75-79 | Caries of permanent teeth | 690,465.4 (518,226.9 to 897,783.7) | 20,473.0 (15,366.0 to 26,620.2) |
| Incidence | Male | 75-79 | Periodontal diseases | 64,701.7 (44,191.9 to 80,670.4) | 1,918.5 (1,310.3 to 2,392.0) |
| Incidence | Male | 75-79 | Edentulism | 50,479.2 (32,567.1 to 73,350.7) | 1,496.8 (965.6 to 2,174.9) |
| Incidence | Male | 80-84 | Main Oral disorders | 409,729.1 (321,272.9 to 515,905.9) | 22,331.2 (17,510.1 to 28,118.1) |
| Incidence | Male | 80-84 | Caries of deciduous teeth | 0.0 (0.0 to 0.0) | 0.0 (0.0 to 0.0) |
| Incidence | Male | 80-84 | Caries of permanent teeth | 347,513.4 (257,417.3 to 445,391.6) | 18,940.3 (14,029.9 to 24,274.9) |
| Incidence | Male | 80-84 | Periodontal diseases | 34,978.5 (24,424.1 to 45,477.0) | 1,906.4 (1,331.2 to 2,478.6) |
| Incidence | Male | 80-84 | Edentulism | 27,237.3 (17,270.0 to 38,615.1) | 1,484.5 (941.3 to 2,104.6) |
| Incidence | Male | 85-89 | Main Oral disorders | 169,625.4 (132,380.0 to 215,141.5) | 20,480.4 (15,983.4 to 25,975.9) |
| Incidence | Male | 85-89 | Caries of deciduous teeth | 0.0 (0.0 to 0.0) | 0.0 (0.0 to 0.0) |
| Incidence | Male | 85-89 | Caries of permanent teeth | 142,698.4 (105,832.5 to 184,313.4) | 17,229.2 (12,778.1 to 22,253.8) |
| Incidence | Male | 85-89 | Periodontal diseases | 15,875.4 (9,821.7 to 20,657.1) | 1,916.8 (1,185.9 to 2,494.1) |
| Incidence | Male | 85-89 | Edentulism | 11,051.6 (7,388.6 to 16,186.2) | 1,334.4 (892.1 to 1,954.3) |
| Incidence | Male | 90-94 | Main Oral disorders | 53,293.2 (39,925.9 to 69,294.6) | 18,255.9 (13,676.8 to 23,737.3) |
| Incidence | Male | 90-94 | Caries of deciduous teeth | 0.0 (0.0 to 0.0) | 0.0 (0.0 to 0.0) |
| Incidence | Male | 90-94 | Caries of permanent teeth | 44,150.0 (30,576.6 to 59,656.4) | 15,123.9 (10,474.2 to 20,435.7) |
| Incidence | Male | 90-94 | Periodontal diseases | 5,599.6 (3,082.9 to 7,387.9) | 1,918.2 (1,056.1 to 2,530.8) |
| Incidence | Male | 90-94 | Edentulism | 3,543.5 (2,219.6 to 5,330.6) | 1,213.9 (760.3 to 1,826.0) |
| Incidence | Male | 95+ | Main Oral disorders | 13,816.4 (8,431.9 to 20,066.5) | 15,700.8 (9,581.9 to 22,803.2) |
| Incidence | Male | 95+ | Caries of deciduous teeth | 0.0 (0.0 to 0.0) | 0.0 (0.0 to 0.0) |
| Incidence | Male | 95+ | Caries of permanent teeth | 11,148.8 (5,894.7 to 17,124.7) | 12,669.3 (6,698.6 to 19,460.2) |
| Incidence | Male | 95+ | Periodontal diseases | 1,645.6 (905.6 to 2,258.7) | 1,870.1 (1,029.1 to 2,566.7) |
| Incidence | Male | 95+ | Edentulism | 1,022.0 (565.8 to 1,733.9) | 1,161.4 (642.9 to 1,970.4) |
| DALYs | Female | <5 | Main Oral disorders | 11,330.8 (6,331.3 to 18,062.1) | 42.7 (23.9 to 68.1) |
| DALYs | Female | <5 | Caries of deciduous teeth | 4,092.5 (1,764.7 to 8,291.6) | 15.4 (6.7 to 31.3) |
| DALYs | Female | <5 | Caries of permanent teeth | 0.0 (0.0 to 0.0) | 0.0 (0.0 to 0.0) |
| DALYs | Female | <5 | Periodontal diseases | 0.0 (0.0 to 0.0) | 0.0 (0.0 to 0.0) |
| DALYs | Female | <5 | Edentulism | 0.0 (0.0 to 0.0) | 0.0 (0.0 to 0.0) |
| DALYs | Female | <5 | Other oral disorders | 7,238.3 (4,298.6 to 10,708.5) | 27.3 (16.2 to 40.4) |
| DALYs | Female | 5-9 | Main Oral disorders | 16,928.4 (9,206.6 to 27,850.0) | 61.7 (33.5 to 101.5) |
| DALYs | Female | 5-9 | Caries of deciduous teeth | 4,691.8 (2,028.8 to 9,073.7) | 17.1 (7.4 to 33.1) |
| DALYs | Female | 5-9 | Caries of permanent teeth | 3,142.7 (994.2 to 7,224.2) | 11.4 (3.6 to 26.3) |
| DALYs | Female | 5-9 | Periodontal diseases | 0.0 (0.0 to 0.0) | 0.0 (0.0 to 0.0) |
| DALYs | Female | 5-9 | Edentulism | 0.0 (0.0 to 0.0) | 0.0 (0.0 to 0.0) |
| DALYs | Female | 5-9 | Other oral disorders | 9,094.0 (5,391.0 to 13,807.6) | 33.1 (19.6 to 50.3) |
| DALYs | Female | 10-14 | Main Oral disorders | 17,660.0 (9,932.6 to 30,475.1) | 64.8 (36.4 to 111.8) |
| DALYs | Female | 10-14 | Caries of deciduous teeth | 639.7 (164.4 to 1,547.8) | 2.3 (0.6 to 5.7) |
| DALYs | Female | 10-14 | Caries of permanent teeth | 7,508.1 (2,936.2 to 15,908.4) | 27.5 (10.8 to 58.4) |
| DALYs | Female | 10-14 | Periodontal diseases | 0.0 (0.0 to 0.0) | 0.0 (0.0 to 0.0) |
| DALYs | Female | 10-14 | Edentulism | 0.0 (0.0 to 0.0) | 0.0 (0.0 to 0.0) |
| DALYs | Female | 10-14 | Other oral disorders | 9,512.3 (5,595.5 to 14,679.7) | 34.9 (20.5 to 53.9) |
| DALYs | Female | 15-19 | Main Oral disorders | 23,064.2 (12,495.0 to 38,228.2) | 86.5 (46.8 to 143.3) |
| DALYs | Female | 15-19 | Caries of deciduous teeth | 0.0 (0.0 to 0.0) | 0.0 (0.0 to 0.0) |
| DALYs | Female | 15-19 | Caries of permanent teeth | 8,260.3 (3,083.5 to 17,188.5) | 31.0 (11.6 to 64.4) |
| DALYs | Female | 15-19 | Periodontal diseases | 1,016.7 (325.0 to 2,316.4) | 3.8 (1.2 to 8.7) |
| DALYs | Female | 15-19 | Edentulism | 0.0 (0.0 to 0.0) | 0.0 (0.0 to 0.0) |
| DALYs | Female | 15-19 | Other oral disorders | 13,787.2 (8,170.1 to 20,848.0) | 51.7 (30.6 to 78.2) |
| DALYs | Female | 20-24 | Main Oral disorders | 32,255.6 (17,894.1 to 52,609.2) | 120.4 (66.8 to 196.3) |
| DALYs | Female | 20-24 | Caries of deciduous teeth | 0.0 (0.0 to 0.0) | 0.0 (0.0 to 0.0) |
| DALYs | Female | 20-24 | Caries of permanent teeth | 9,981.7 (3,822.7 to 19,525.0) | 37.2 (14.3 to 72.9) |
| DALYs | Female | 20-24 | Periodontal diseases | 3,926.9 (1,391.3 to 9,058.2) | 14.7 (5.2 to 33.8) |
| DALYs | Female | 20-24 | Edentulism | 1,489.6 (770.6 to 2,512.2) | 5.6 (2.9 to 9.4) |
| DALYs | Female | 20-24 | Other oral disorders | 16,857.5 (10,298.0 to 25,280.9) | 62.9 (38.4 to 94.3) |
| DALYs | Female | 25-29 | Main Oral disorders | 43,184.8 (24,516.7 to 70,247.0) | 158.2 (89.8 to 257.4) |
| DALYs | Female | 25-29 | Caries of deciduous teeth | 0.0 (0.0 to 0.0) | 0.0 (0.0 to 0.0) |
| DALYs | Female | 25-29 | Caries of permanent teeth | 10,114.2 (4,224.4 to 19,882.3) | 37.1 (15.5 to 72.8) |
| DALYs | Female | 25-29 | Periodontal diseases | 9,900.6 (3,636.0 to 21,608.6) | 36.3 (13.3 to 79.2) |
| DALYs | Female | 25-29 | Edentulism | 4,498.5 (2,443.4 to 7,020.3) | 16.5 (9.0 to 25.7) |
| DALYs | Female | 25-29 | Other oral disorders | 18,671.5 (11,474.4 to 28,208.4) | 68.4 (42.0 to 103.3) |
| DALYs | Female | 30-34 | Main Oral disorders | 54,493.3 (30,052.3 to 88,085.3) | 204.9 (113.0 to 331.2) |
| DALYs | Female | 30-34 | Caries of deciduous teeth | 0.0 (0.0 to 0.0) | 0.0 (0.0 to 0.0) |
| DALYs | Female | 30-34 | Caries of permanent teeth | 9,488.7 (3,876.1 to 18,797.1) | 35.7 (14.6 to 70.7) |
| DALYs | Female | 30-34 | Periodontal diseases | 17,303.4 (6,343.5 to 37,450.2) | 65.1 (23.9 to 140.8) |
| DALYs | Female | 30-34 | Edentulism | 8,515.5 (5,063.4 to 13,369.1) | 32.0 (19.0 to 50.3) |
| DALYs | Female | 30-34 | Other oral disorders | 19,185.6 (11,662.3 to 29,206.5) | 72.1 (43.8 to 109.8) |
| DALYs | Female | 35-39 | Main Oral disorders | 65,788.7 (36,828.6 to 107,517.9) | 260.6 (145.9 to 426.0) |
| DALYs | Female | 35-39 | Caries of deciduous teeth | 0.0 (0.0 to 0.0) | 0.0 (0.0 to 0.0) |
| DALYs | Female | 35-39 | Caries of permanent teeth | 9,387.4 (3,747.7 to 18,856.5) | 37.2 (14.8 to 74.7) |
| DALYs | Female | 35-39 | Periodontal diseases | 24,350.2 (9,920.1 to 52,083.6) | 96.5 (39.3 to 206.3) |
| DALYs | Female | 35-39 | Edentulism | 13,321.3 (7,832.2 to 20,447.7) | 52.8 (31.0 to 81.0) |
| DALYs | Female | 35-39 | Other oral disorders | 18,729.8 (11,135.0 to 27,790.5) | 74.2 (44.1 to 110.1) |
| DALYs | Female | 40-44 | Main Oral disorders | 76,942.3 (42,516.2 to 124,603.7) | 323.3 (178.7 to 523.6) |
| DALYs | Female | 40-44 | Caries of deciduous teeth | 0.0 (0.0 to 0.0) | 0.0 (0.0 to 0.0) |
| DALYs | Female | 40-44 | Caries of permanent teeth | 8,900.6 (3,499.4 to 17,006.9) | 37.4 (14.7 to 71.5) |
| DALYs | Female | 40-44 | Periodontal diseases | 30,281.5 (12,310.9 to 64,595.5) | 127.3 (51.7 to 271.4) |
| DALYs | Female | 40-44 | Edentulism | 19,762.8 (11,741.4 to 29,358.1) | 83.0 (49.3 to 123.4) |
| DALYs | Female | 40-44 | Other oral disorders | 17,997.5 (10,726.4 to 26,604.8) | 75.6 (45.1 to 111.8) |
| DALYs | Female | 45-49 | Main Oral disorders | 85,894.6 (47,872.8 to 137,097.9) | 396.3 (220.9 to 632.5) |
| DALYs | Female | 45-49 | Caries of deciduous teeth | 0.0 (0.0 to 0.0) | 0.0 (0.0 to 0.0) |
| DALYs | Female | 45-49 | Caries of permanent teeth | 8,042.9 (3,248.4 to 16,465.4) | 37.1 (15.0 to 76.0) |
| DALYs | Female | 45-49 | Periodontal diseases | 33,021.6 (13,288.1 to 67,751.0) | 152.3 (61.3 to 312.6) |
| DALYs | Female | 45-49 | Edentulism | 28,247.4 (17,386.9 to 42,250.8) | 130.3 (80.2 to 194.9) |
| DALYs | Female | 45-49 | Other oral disorders | 16,582.6 (10,058.5 to 24,587.8) | 76.5 (46.4 to 113.4) |
| DALYs | Female | 50-54 | Main Oral disorders | 95,801.4 (55,384.6 to 148,863.4) | 482.8 (279.1 to 750.2) |
| DALYs | Female | 50-54 | Caries of deciduous teeth | 0.0 (0.0 to 0.0) | 0.0 (0.0 to 0.0) |
| DALYs | Female | 50-54 | Caries of permanent teeth | 7,343.6 (2,996.3 to 15,730.2) | 37.0 (15.1 to 79.3) |
| DALYs | Female | 50-54 | Periodontal diseases | 33,376.0 (13,149.7 to 66,793.9) | 168.2 (66.3 to 336.6) |
| DALYs | Female | 50-54 | Edentulism | 39,952.1 (23,791.5 to 59,334.8) | 201.3 (119.9 to 299.0) |
| DALYs | Female | 50-54 | Other oral disorders | 15,129.7 (9,146.1 to 22,260.7) | 76.2 (46.1 to 112.2) |
| DALYs | Female | 55-59 | Main Oral disorders | 102,068.3 (60,895.2 to 153,411.6) | 587.9 (350.7 to 883.6) |
| DALYs | Female | 55-59 | Caries of deciduous teeth | 0.0 (0.0 to 0.0) | 0.0 (0.0 to 0.0) |
| DALYs | Female | 55-59 | Caries of permanent teeth | 6,425.7 (2,707.7 to 13,924.8) | 37.0 (15.6 to 80.2) |
| DALYs | Female | 55-59 | Periodontal diseases | 30,313.7 (11,718.8 to 59,528.1) | 174.6 (67.5 to 342.9) |
| DALYs | Female | 55-59 | Edentulism | 52,319.2 (31,148.0 to 75,869.1) | 301.3 (179.4 to 437.0) |
| DALYs | Female | 55-59 | Other oral disorders | 13,009.7 (7,996.1 to 18,932.1) | 74.9 (46.1 to 109.0) |
| DALYs | Female | 60-64 | Main Oral disorders | 103,393.3 (61,855.4 to 154,256.4) | 718.8 (430.0 to 1,072.4) |
| DALYs | Female | 60-64 | Caries of deciduous teeth | 0.0 (0.0 to 0.0) | 0.0 (0.0 to 0.0) |
| DALYs | Female | 60-64 | Caries of permanent teeth | 5,307.0 (2,214.0 to 10,713.7) | 36.9 (15.4 to 74.5) |
| DALYs | Female | 60-64 | Periodontal diseases | 24,474.9 (9,472.4 to 48,183.1) | 170.2 (65.9 to 335.0) |
| DALYs | Female | 60-64 | Edentulism | 63,006.1 (38,655.5 to 88,018.9) | 438.0 (268.7 to 611.9) |
| DALYs | Female | 60-64 | Other oral disorders | 10,605.3 (6,521.0 to 15,521.2) | 73.7 (45.3 to 107.9) |
| DALYs | Female | 65-69 | Main Oral disorders | 93,913.0 (57,222.1 to 137,857.3) | 868.2 (529.0 to 1,274.5) |
| DALYs | Female | 65-69 | Caries of deciduous teeth | 0.0 (0.0 to 0.0) | 0.0 (0.0 to 0.0) |
| DALYs | Female | 65-69 | Caries of permanent teeth | 3,948.7 (1,748.1 to 7,349.4) | 36.5 (16.2 to 67.9) |
| DALYs | Female | 65-69 | Periodontal diseases | 17,381.5 (6,711.0 to 35,203.2) | 160.7 (62.0 to 325.5) |
| DALYs | Female | 65-69 | Edentulism | 64,747.2 (40,088.7 to 92,354.5) | 598.6 (370.6 to 853.8) |
| DALYs | Female | 65-69 | Other oral disorders | 7,835.6 (4,840.1 to 11,663.2) | 72.4 (44.7 to 107.8) |
| DALYs | Female | 70-74 | Main Oral disorders | 71,505.0 (45,871.9 to 104,896.4) | 1,008.1 (646.7 to 1,478.8) |
| DALYs | Female | 70-74 | Caries of deciduous teeth | 0.0 (0.0 to 0.0) | 0.0 (0.0 to 0.0) |
| DALYs | Female | 70-74 | Caries of permanent teeth | 2,551.5 (1,103.7 to 4,758.1) | 36.0 (15.6 to 67.1) |
| DALYs | Female | 70-74 | Periodontal diseases | 10,557.8 (4,097.8 to 21,425.6) | 148.8 (57.8 to 302.1) |
| DALYs | Female | 70-74 | Edentulism | 53,649.8 (35,413.2 to 75,976.7) | 756.4 (499.3 to 1,071.1) |
| DALYs | Female | 70-74 | Other oral disorders | 4,746.0 (2,917.0 to 7,222.8) | 66.9 (41.1 to 101.8) |
| DALYs | Female | 75-79 | Main Oral disorders | 49,703.3 (31,554.6 to 69,258.2) | 1,100.8 (698.8 to 1,533.9) |
| DALYs | Female | 75-79 | Caries of deciduous teeth | 0.0 (0.0 to 0.0) | 0.0 (0.0 to 0.0) |
| DALYs | Female | 75-79 | Caries of permanent teeth | 1,487.8 (676.3 to 2,797.1) | 33.0 (15.0 to 61.9) |
| DALYs | Female | 75-79 | Periodontal diseases | 6,293.6 (2,407.0 to 13,214.0) | 139.4 (53.3 to 292.7) |
| DALYs | Female | 75-79 | Edentulism | 39,333.8 (25,561.5 to 55,716.0) | 871.1 (566.1 to 1,233.9) |
| DALYs | Female | 75-79 | Other oral disorders | 2,588.1 (1,531.0 to 3,937.1) | 57.3 (33.9 to 87.2) |
| DALYs | Female | 80-84 | Main Oral disorders | 32,575.6 (20,962.5 to 45,766.1) | 1,159.5 (746.1 to 1,629.0) |
| DALYs | Female | 80-84 | Caries of deciduous teeth | 0.0 (0.0 to 0.0) | 0.0 (0.0 to 0.0) |
| DALYs | Female | 80-84 | Caries of permanent teeth | 840.6 (366.2 to 1,600.1) | 29.9 (13.0 to 57.0) |
| DALYs | Female | 80-84 | Periodontal diseases | 3,670.2 (1,418.9 to 7,806.4) | 130.6 (50.5 to 277.9) |
| DALYs | Female | 80-84 | Edentulism | 26,754.6 (17,550.0 to 37,896.7) | 952.3 (624.7 to 1,348.9) |
| DALYs | Female | 80-84 | Other oral disorders | 1,310.2 (791.5 to 1,934.9) | 46.6 (28.2 to 68.9) |
| DALYs | Female | 85-89 | Main Oral disorders | 16,690.4 (10,668.4 to 23,351.1) | 1,174.9 (751.0 to 1,643.8) |
| DALYs | Female | 85-89 | Caries of deciduous teeth | 0.0 (0.0 to 0.0) | 0.0 (0.0 to 0.0) |
| DALYs | Female | 85-89 | Caries of permanent teeth | 367.7 (153.5 to 720.3) | 25.9 (10.8 to 50.7) |
| DALYs | Female | 85-89 | Periodontal diseases | 1,772.8 (670.5 to 3,834.3) | 124.8 (47.2 to 269.9) |
| DALYs | Female | 85-89 | Edentulism | 14,059.0 (9,187.1 to 19,502.3) | 989.7 (646.7 to 1,372.8) |
| DALYs | Female | 85-89 | Other oral disorders | 490.8 (306.5 to 728.1) | 34.6 (21.6 to 51.3) |
| DALYs | Female | 90-94 | Main Oral disorders | 6,092.7 (3,923.7 to 8,574.8) | 1,162.7 (748.8 to 1,636.3) |
| DALYs | Female | 90-94 | Caries of deciduous teeth | 0.0 (0.0 to 0.0) | 0.0 (0.0 to 0.0) |
| DALYs | Female | 90-94 | Caries of permanent teeth | 109.1 (43.3 to 221.4) | 20.8 (8.3 to 42.3) |
| DALYs | Female | 90-94 | Periodontal diseases | 639.7 (247.1 to 1,362.4) | 122.1 (47.1 to 260.0) |
| DALYs | Female | 90-94 | Edentulism | 5,193.6 (3,403.0 to 7,232.6) | 991.1 (649.4 to 1,380.2) |
| DALYs | Female | 90-94 | Other oral disorders | 150.3 (94.7 to 219.1) | 28.7 (18.1 to 41.8) |
| DALYs | Female | 95+ | Main Oral disorders | 1,657.9 (1,071.4 to 2,354.3) | 1,126.6 (728.0 to 1,599.8) |
| DALYs | Female | 95+ | Caries of deciduous teeth | 0.0 (0.0 to 0.0) | 0.0 (0.0 to 0.0) |
| DALYs | Female | 95+ | Caries of permanent teeth | 21.1 (8.5 to 44.1) | 14.3 (5.7 to 30.0) |
| DALYs | Female | 95+ | Periodontal diseases | 177.4 (71.6 to 366.2) | 120.5 (48.7 to 248.8) |
| DALYs | Female | 95+ | Edentulism | 1,417.3 (920.1 to 2,018.8) | 963.1 (625.2 to 1,371.8) |
| DALYs | Female | 95+ | Other oral disorders | 42.1 (26.4 to 62.8) | 28.6 (18.0 to 42.7) |
| DALYs | Male | <5 | Main Oral disorders | 10,957.2 (6,015.9 to 17,533.8) | 38.9 (21.4 to 62.3) |
| DALYs | Male | <5 | Caries of deciduous teeth | 4,351.8 (1,832.9 to 8,815.0) | 15.5 (6.5 to 31.3) |
| DALYs | Male | <5 | Caries of permanent teeth | 0.0 (0.0 to 0.0) | 0.0 (0.0 to 0.0) |
| DALYs | Male | <5 | Periodontal diseases | 0.0 (0.0 to 0.0) | 0.0 (0.0 to 0.0) |
| DALYs | Male | <5 | Edentulism | 0.0 (0.0 to 0.0) | 0.0 (0.0 to 0.0) |
| DALYs | Male | <5 | Other oral disorders | 6,605.4 (3,942.0 to 9,913.6) | 23.5 (14.0 to 35.2) |
| DALYs | Male | 5-9 | Main Oral disorders | 16,483.6 (8,862.2 to 27,445.7) | 56.7 (30.5 to 94.4) |
| DALYs | Male | 5-9 | Caries of deciduous teeth | 4,977.8 (2,159.6 to 9,425.1) | 17.1 (7.4 to 32.4) |
| DALYs | Male | 5-9 | Caries of permanent teeth | 3,234.4 (1,038.4 to 7,534.4) | 11.1 (3.6 to 25.9) |
| DALYs | Male | 5-9 | Periodontal diseases | 0.0 (0.0 to 0.0) | 0.0 (0.0 to 0.0) |
| DALYs | Male | 5-9 | Edentulism | 0.0 (0.0 to 0.0) | 0.0 (0.0 to 0.0) |
| DALYs | Male | 5-9 | Other oral disorders | 8,271.4 (4,916.5 to 12,583.4) | 28.4 (16.9 to 43.3) |
| DALYs | Male | 10-14 | Main Oral disorders | 17,134.4 (9,436.0 to 29,263.3) | 59.3 (32.7 to 101.3) |
| DALYs | Male | 10-14 | Caries of deciduous teeth | 682.0 (163.5 to 1,641.7) | 2.4 (0.6 to 5.7) |
| DALYs | Male | 10-14 | Caries of permanent teeth | 7,782.0 (3,048.8 to 16,324.0) | 26.9 (10.6 to 56.5) |
| DALYs | Male | 10-14 | Periodontal diseases | 0.0 (0.0 to 0.0) | 0.0 (0.0 to 0.0) |
| DALYs | Male | 10-14 | Edentulism | 0.0 (0.0 to 0.0) | 0.0 (0.0 to 0.0) |
| DALYs | Male | 10-14 | Other oral disorders | 8,670.4 (5,049.5 to 13,551.4) | 30.0 (17.5 to 46.9) |
| DALYs | Male | 15-19 | Main Oral disorders | 21,955.5 (11,579.7 to 37,187.6) | 78.3 (41.3 to 132.6) |
| DALYs | Male | 15-19 | Caries of deciduous teeth | 0.0 (0.0 to 0.0) | 0.0 (0.0 to 0.0) |
| DALYs | Male | 15-19 | Caries of permanent teeth | 8,595.8 (3,230.1 to 17,918.3) | 30.6 (11.5 to 63.9) |
| DALYs | Male | 15-19 | Periodontal diseases | 869.1 (273.7 to 1,935.7) | 3.1 (1.0 to 6.9) |
| DALYs | Male | 15-19 | Edentulism | 0.0 (0.0 to 0.0) | 0.0 (0.0 to 0.0) |
| DALYs | Male | 15-19 | Other oral disorders | 12,490.6 (7,366.7 to 19,093.2) | 44.5 (26.3 to 68.1) |
| DALYs | Male | 20-24 | Main Oral disorders | 29,478.9 (15,978.7 to 48,835.9) | 105.9 (57.4 to 175.4) |
| DALYs | Male | 20-24 | Caries of deciduous teeth | 0.0 (0.0 to 0.0) | 0.0 (0.0 to 0.0) |
| DALYs | Male | 20-24 | Caries of permanent teeth | 10,402.8 (3,925.6 to 20,385.4) | 37.4 (14.1 to 73.2) |
| DALYs | Male | 20-24 | Periodontal diseases | 3,189.6 (1,105.3 to 7,241.8) | 11.5 (4.0 to 26.0) |
| DALYs | Male | 20-24 | Edentulism | 758.1 (340.2 to 1,303.4) | 2.7 (1.2 to 4.7) |
| DALYs | Male | 20-24 | Other oral disorders | 15,128.3 (9,115.3 to 22,513.1) | 54.3 (32.7 to 80.9) |
| DALYs | Male | 25-29 | Main Oral disorders | 37,314.5 (20,638.3 to 62,028.4) | 133.1 (73.6 to 221.2) |
| DALYs | Male | 25-29 | Caries of deciduous teeth | 0.0 (0.0 to 0.0) | 0.0 (0.0 to 0.0) |
| DALYs | Male | 25-29 | Caries of permanent teeth | 10,462.8 (4,317.2 to 20,837.7) | 37.3 (15.4 to 74.3) |
| DALYs | Male | 25-29 | Periodontal diseases | 8,017.3 (2,936.7 to 17,549.7) | 28.6 (10.5 to 62.6) |
| DALYs | Male | 25-29 | Edentulism | 2,148.1 (1,128.6 to 3,627.3) | 7.7 (4.0 to 12.9) |
| DALYs | Male | 25-29 | Other oral disorders | 16,686.2 (10,325.0 to 24,779.9) | 59.5 (36.8 to 88.4) |
| DALYs | Male | 30-34 | Main Oral disorders | 45,502.7 (24,688.0 to 76,109.2) | 166.3 (90.2 to 278.2) |
| DALYs | Male | 30-34 | Caries of deciduous teeth | 0.0 (0.0 to 0.0) | 0.0 (0.0 to 0.0) |
| DALYs | Male | 30-34 | Caries of permanent teeth | 9,796.7 (3,922.5 to 19,245.0) | 35.8 (14.3 to 70.3) |
| DALYs | Male | 30-34 | Periodontal diseases | 14,555.2 (5,342.7 to 32,126.8) | 53.2 (19.5 to 117.4) |
| DALYs | Male | 30-34 | Edentulism | 3,946.7 (2,247.7 to 6,299.5) | 14.4 (8.2 to 23.0) |
| DALYs | Male | 30-34 | Other oral disorders | 17,204.1 (10,578.6 to 25,970.6) | 62.9 (38.7 to 94.9) |
| DALYs | Male | 35-39 | Main Oral disorders | 53,747.0 (28,602.5 to 90,982.7) | 208.4 (110.9 to 352.8) |
| DALYs | Male | 35-39 | Caries of deciduous teeth | 0.0 (0.0 to 0.0) | 0.0 (0.0 to 0.0) |
| DALYs | Male | 35-39 | Caries of permanent teeth | 9,607.7 (3,903.6 to 19,502.9) | 37.3 (15.1 to 75.6) |
| DALYs | Male | 35-39 | Periodontal diseases | 21,547.6 (8,553.9 to 45,722.0) | 83.6 (33.2 to 177.3) |
| DALYs | Male | 35-39 | Edentulism | 5,985.2 (3,428.3 to 9,475.8) | 23.2 (13.3 to 36.7) |
| DALYs | Male | 35-39 | Other oral disorders | 16,606.5 (9,991.1 to 24,850.6) | 64.4 (38.7 to 96.4) |
| DALYs | Male | 40-44 | Main Oral disorders | 61,639.3 (32,248.5 to 104,843.6) | 256.1 (134.0 to 435.6) |
| DALYs | Male | 40-44 | Caries of deciduous teeth | 0.0 (0.0 to 0.0) | 0.0 (0.0 to 0.0) |
| DALYs | Male | 40-44 | Caries of permanent teeth | 9,037.0 (3,619.5 to 17,573.6) | 37.5 (15.0 to 73.0) |
| DALYs | Male | 40-44 | Periodontal diseases | 28,075.8 (10,914.0 to 59,736.5) | 116.7 (45.3 to 248.2) |
| DALYs | Male | 40-44 | Edentulism | 8,741.2 (5,092.1 to 13,178.2) | 36.3 (21.2 to 54.8) |
| DALYs | Male | 40-44 | Other oral disorders | 15,785.3 (9,450.3 to 24,061.3) | 65.6 (39.3 to 100.0) |
| DALYs | Male | 45-49 | Main Oral disorders | 66,532.6 (35,607.7 to 112,935.0) | 308.4 (165.0 to 523.4) |
| DALYs | Male | 45-49 | Caries of deciduous teeth | 0.0 (0.0 to 0.0) | 0.0 (0.0 to 0.0) |
| DALYs | Male | 45-49 | Caries of permanent teeth | 8,034.0 (3,224.8 to 16,182.9) | 37.2 (14.9 to 75.0) |
| DALYs | Male | 45-49 | Periodontal diseases | 31,419.6 (12,425.8 to 64,957.3) | 145.6 (57.6 to 301.1) |
| DALYs | Male | 45-49 | Edentulism | 12,699.6 (7,587.8 to 19,411.9) | 58.9 (35.2 to 90.0) |
| DALYs | Male | 45-49 | Other oral disorders | 14,379.4 (8,415.0 to 21,387.2) | 66.6 (39.0 to 99.1) |
| DALYs | Male | 50-54 | Main Oral disorders | 71,302.0 (38,949.6 to 116,426.8) | 368.6 (201.3 to 601.8) |
| DALYs | Male | 50-54 | Caries of deciduous teeth | 0.0 (0.0 to 0.0) | 0.0 (0.0 to 0.0) |
| DALYs | Male | 50-54 | Caries of permanent teeth | 7,179.2 (2,925.7 to 15,565.0) | 37.1 (15.1 to 80.5) |
| DALYs | Male | 50-54 | Periodontal diseases | 32,149.8 (12,571.0 to 65,084.3) | 166.2 (65.0 to 336.4) |
| DALYs | Male | 50-54 | Edentulism | 19,137.9 (11,292.7 to 28,606.1) | 98.9 (58.4 to 147.9) |
| DALYs | Male | 50-54 | Other oral disorders | 12,835.2 (7,569.3 to 19,009.1) | 66.3 (39.1 to 98.3) |
| DALYs | Male | 55-59 | Main Oral disorders | 73,237.3 (41,922.6 to 115,540.8) | 445.0 (254.8 to 702.1) |
| DALYs | Male | 55-59 | Caries of deciduous teeth | 0.0 (0.0 to 0.0) | 0.0 (0.0 to 0.0) |
| DALYs | Male | 55-59 | Caries of permanent teeth | 6,132.7 (2,564.5 to 13,328.8) | 37.3 (15.6 to 81.0) |
| DALYs | Male | 55-59 | Periodontal diseases | 29,094.8 (11,233.9 to 58,038.3) | 176.8 (68.3 to 352.7) |
| DALYs | Male | 55-59 | Edentulism | 27,333.0 (16,375.2 to 40,702.9) | 166.1 (99.5 to 247.3) |
| DALYs | Male | 55-59 | Other oral disorders | 10,676.8 (6,641.0 to 15,707.4) | 64.9 (40.4 to 95.5) |
| DALYs | Male | 60-64 | Main Oral disorders | 72,200.0 (42,536.0 to 112,895.9) | 545.3 (321.2 to 852.6) |
| DALYs | Male | 60-64 | Caries of deciduous teeth | 0.0 (0.0 to 0.0) | 0.0 (0.0 to 0.0) |
| DALYs | Male | 60-64 | Caries of permanent teeth | 4,928.9 (2,055.5 to 10,045.4) | 37.2 (15.5 to 75.9) |
| DALYs | Male | 60-64 | Periodontal diseases | 23,346.2 (9,125.9 to 45,944.1) | 176.3 (68.9 to 347.0) |
| DALYs | Male | 60-64 | Edentulism | 35,518.4 (21,336.2 to 50,749.8) | 268.2 (161.1 to 383.3) |
| DALYs | Male | 60-64 | Other oral disorders | 8,406.4 (5,253.7 to 12,480.9) | 63.5 (39.7 to 94.3) |
| DALYs | Male | 65-69 | Main Oral disorders | 63,435.4 (37,922.0 to 94,475.0) | 668.1 (399.4 to 995.0) |
| DALYs | Male | 65-69 | Caries of deciduous teeth | 0.0 (0.0 to 0.0) | 0.0 (0.0 to 0.0) |
| DALYs | Male | 65-69 | Caries of permanent teeth | 3,493.0 (1,562.8 to 6,747.3) | 36.8 (16.5 to 71.1) |
| DALYs | Male | 65-69 | Periodontal diseases | 16,215.4 (6,228.8 to 32,259.1) | 170.8 (65.6 to 339.7) |
| DALYs | Male | 65-69 | Edentulism | 37,851.8 (22,948.2 to 53,734.8) | 398.6 (241.7 to 565.9) |
| DALYs | Male | 65-69 | Other oral disorders | 5,875.2 (3,555.4 to 8,995.4) | 61.9 (37.4 to 94.7) |
| DALYs | Male | 70-74 | Main Oral disorders | 46,907.7 (29,112.2 to 69,668.8) | 792.2 (491.7 to 1,176.6) |
| DALYs | Male | 70-74 | Caries of deciduous teeth | 0.0 (0.0 to 0.0) | 0.0 (0.0 to 0.0) |
| DALYs | Male | 70-74 | Caries of permanent teeth | 2,136.6 (925.4 to 4,071.8) | 36.1 (15.6 to 68.8) |
| DALYs | Male | 70-74 | Periodontal diseases | 9,594.9 (3,713.0 to 19,407.3) | 162.0 (62.7 to 327.8) |
| DALYs | Male | 70-74 | Edentulism | 31,795.9 (20,262.6 to 45,727.7) | 537.0 (342.2 to 772.3) |
| DALYs | Male | 70-74 | Other oral disorders | 3,380.3 (2,108.1 to 5,119.4) | 57.1 (35.6 to 86.5) |
| DALYs | Male | 75-79 | Main Oral disorders | 30,021.2 (18,589.6 to 42,965.6) | 890.2 (551.2 to 1,274.0) |
| DALYs | Male | 75-79 | Caries of deciduous teeth | 0.0 (0.0 to 0.0) | 0.0 (0.0 to 0.0) |
| DALYs | Male | 75-79 | Caries of permanent teeth | 1,112.0 (504.5 to 2,078.6) | 33.0 (15.0 to 61.6) |
| DALYs | Male | 75-79 | Periodontal diseases | 5,219.9 (2,037.2 to 11,001.1) | 154.8 (60.4 to 326.2) |
| DALYs | Male | 75-79 | Edentulism | 22,027.8 (13,920.6 to 31,688.8) | 653.1 (412.8 to 939.6) |
| DALYs | Male | 75-79 | Other oral disorders | 1,661.5 (1,010.5 to 2,443.1) | 49.3 (30.0 to 72.4) |
| DALYs | Male | 80-84 | Main Oral disorders | 17,810.9 (11,063.7 to 25,219.4) | 970.7 (603.0 to 1,374.5) |
| DALYs | Male | 80-84 | Caries of deciduous teeth | 0.0 (0.0 to 0.0) | 0.0 (0.0 to 0.0) |
| DALYs | Male | 80-84 | Caries of permanent teeth | 548.4 (236.6 to 1,066.9) | 29.9 (12.9 to 58.1) |
| DALYs | Male | 80-84 | Periodontal diseases | 2,693.0 (1,052.1 to 5,563.3) | 146.8 (57.3 to 303.2) |
| DALYs | Male | 80-84 | Edentulism | 13,829.0 (8,913.9 to 19,643.7) | 753.7 (485.8 to 1,070.6) |
| DALYs | Male | 80-84 | Other oral disorders | 740.6 (450.0 to 1,069.5) | 40.4 (24.5 to 58.3) |
| DALYs | Male | 85-89 | Main Oral disorders | 8,535.1 (5,367.7 to 12,007.1) | 1,030.5 (648.1 to 1,449.7) |
| DALYs | Male | 85-89 | Caries of deciduous teeth | 0.0 (0.0 to 0.0) | 0.0 (0.0 to 0.0) |
| DALYs | Male | 85-89 | Caries of permanent teeth | 214.2 (91.3 to 417.7) | 25.9 (11.0 to 50.4) |
| DALYs | Male | 85-89 | Periodontal diseases | 1,151.0 (440.3 to 2,421.4) | 139.0 (53.2 to 292.4) |
| DALYs | Male | 85-89 | Edentulism | 6,921.0 (4,465.9 to 9,671.1) | 835.6 (539.2 to 1,167.7) |
| DALYs | Male | 85-89 | Other oral disorders | 249.0 (154.1 to 358.1) | 30.1 (18.6 to 43.2) |
| DALYs | Male | 90-94 | Main Oral disorders | 3,138.2 (1,988.2 to 4,509.4) | 1,075.0 (681.1 to 1,544.7) |
| DALYs | Male | 90-94 | Caries of deciduous teeth | 0.0 (0.0 to 0.0) | 0.0 (0.0 to 0.0) |
| DALYs | Male | 90-94 | Caries of permanent teeth | 61.7 (24.6 to 127.1) | 21.1 (8.4 to 43.5) |
| DALYs | Male | 90-94 | Periodontal diseases | 384.4 (148.2 to 799.5) | 131.7 (50.7 to 273.9) |
| DALYs | Male | 90-94 | Edentulism | 2,619.2 (1,716.9 to 3,678.8) | 897.2 (588.1 to 1,260.2) |
| DALYs | Male | 90-94 | Other oral disorders | 73.0 (45.6 to 106.4) | 25.0 (15.6 to 36.5) |
| DALYs | Male | 95+ | Main Oral disorders | 988.4 (640.5 to 1,396.8) | 1,123.2 (727.8 to 1,587.3) |
| DALYs | Male | 95+ | Caries of deciduous teeth | 0.0 (0.0 to 0.0) | 0.0 (0.0 to 0.0) |
| DALYs | Male | 95+ | Caries of permanent teeth | 13.4 (5.3 to 28.3) | 15.2 (6.1 to 32.2) |
| DALYs | Male | 95+ | Periodontal diseases | 107.6 (42.2 to 218.8) | 122.3 (48.0 to 248.6) |
| DALYs | Male | 95+ | Edentulism | 845.2 (551.5 to 1,207.4) | 960.5 (626.8 to 1,372.1) |
| DALYs | Male | 95+ | Other oral disorders | 22.1 (13.8 to 32.4) | 25.2 (15.7 to 36.8) |

**Appendix Table 5.** Decomposition of changes in oral disorder burden between 1990 and 2021.

| Location | Cause | Measure | Factor | Contribution | Contribution (%) | Total |
| --- | --- | --- | --- | --- | --- | --- |
| ASEAN | Main Oral disorders | Prevalence | Attributed to Age Structure Shift | 16029380 | 14.60% | 1.1E+08 |
| ASEAN | Main Oral disorders | Prevalence | Attributed to Population Growth | 1.08E+08 | 97.70% | 1.1E+08 |
| ASEAN | Main Oral disorders | Prevalence | Attributed to Epidemiologic Changes | -1.3E+07 | -12.30% | 1.1E+08 |
| ASEAN | Main Oral disorders | Incidence | Attributed to Age Structure Shift | -4.3E+07 | -45.60% | 93737814 |
| ASEAN | Main Oral disorders | Incidence | Attributed to Population Growth | 1.34E+08 | 143.00% | 93737814 |
| ASEAN | Main Oral disorders | Incidence | Attributed to Epidemiologic Changes | 2430265 | 2.60% | 93737814 |
| ASEAN | Main Oral disorders | DALYs | Attributed to Age Structure Shift | 420300.5 | 48.80% | 862002.4 |
| ASEAN | Main Oral disorders | DALYs | Attributed to Population Growth | 524187.2 | 60.80% | 862002.4 |
| ASEAN | Main Oral disorders | DALYs | Attributed to Epidemiologic Changes | -82485.2 | -9.60% | 862002.4 |
| ASEAN | Caries of deciduous teeth | Prevalence | Attributed to Age Structure Shift | -2.2E+07 | 1484.30% | -1481707 |
| ASEAN | Caries of deciduous teeth | Prevalence | Attributed to Population Growth | 21864553 | -1475.60% | -1481707 |
| ASEAN | Caries of deciduous teeth | Prevalence | Attributed to Epidemiologic Changes | -1353901 | 91.40% | -1481707 |
| ASEAN | Caries of deciduous teeth | Incidence | Attributed to Age Structure Shift | -4.9E+07 | -1955.80% | 2482511 |
| ASEAN | Caries of deciduous teeth | Incidence | Attributed to Population Growth | 49874449 | 2009.00% | 2482511 |
| ASEAN | Caries of deciduous teeth | Incidence | Attributed to Epidemiologic Changes | 1161901 | 46.80% | 2482511 |
| ASEAN | Caries of deciduous teeth | DALYs | Attributed to Age Structure Shift | -8402.35 | 1806.90% | -465.011 |
| ASEAN | Caries of deciduous teeth | DALYs | Attributed to Population Growth | 8351.209 | -1795.90% | -465.011 |
| ASEAN | Caries of deciduous teeth | DALYs | Attributed to Epidemiologic Changes | -413.876 | 89.00% | -465.011 |
| ASEAN | Caries of permanent teeth | Prevalence | Attributed to Age Structure Shift | 19114558 | 24.90% | 76813523 |
| ASEAN | Caries of permanent teeth | Prevalence | Attributed to Population Growth | 69100255 | 90.00% | 76813523 |
| ASEAN | Caries of permanent teeth | Prevalence | Attributed to Epidemiologic Changes | -1.1E+07 | -14.80% | 76813523 |
| ASEAN | Caries of permanent teeth | Incidence | Attributed to Age Structure Shift | 3614502 | 4.20% | 87001974 |
| ASEAN | Caries of permanent teeth | Incidence | Attributed to Population Growth | 81612391 | 93.80% | 87001974 |
| ASEAN | Caries of permanent teeth | Incidence | Attributed to Epidemiologic Changes | 1775082 | 2.00% | 87001974 |
| ASEAN | Caries of permanent teeth | DALYs | Attributed to Age Structure Shift | 17934.67 | 23.80% | 75212.79 |
| ASEAN | Caries of permanent teeth | DALYs | Attributed to Population Growth | 68230.64 | 90.70% | 75212.79 |
| ASEAN | Caries of permanent teeth | DALYs | Attributed to Epidemiologic Changes | -10952.5 | -14.60% | 75212.79 |
| ASEAN | Periodontal diseases | Prevalence | Attributed to Age Structure Shift | 21196251 | 54.50% | 38878470 |
| ASEAN | Periodontal diseases | Prevalence | Attributed to Population Growth | 21188785 | 54.50% | 38878470 |
| ASEAN | Periodontal diseases | Prevalence | Attributed to Epidemiologic Changes | -3506566 | -9.00% | 38878470 |
| ASEAN | Periodontal diseases | Incidence | Attributed to Age Structure Shift | 1550620 | 49.40% | 3141688 |
| ASEAN | Periodontal diseases | Incidence | Attributed to Population Growth | 1958183 | 62.30% | 3141688 |
| ASEAN | Periodontal diseases | Incidence | Attributed to Epidemiologic Changes | -367115 | -11.70% | 3141688 |
| ASEAN | Periodontal diseases | DALYs | Attributed to Age Structure Shift | 137139.7 | 54.40% | 251952 |
| ASEAN | Periodontal diseases | DALYs | Attributed to Population Growth | 138026.3 | 54.80% | 251952 |
| ASEAN | Periodontal diseases | DALYs | Attributed to Epidemiologic Changes | -23214 | -9.20% | 251952 |
| ASEAN | Edentulism | Prevalence | Attributed to Age Structure Shift | 8907709 | 63.90% | 13949963 |
| ASEAN | Edentulism | Prevalence | Attributed to Population Growth | 6828025 | 48.90% | 13949963 |
| ASEAN | Edentulism | Prevalence | Attributed to Epidemiologic Changes | -1785770 | -12.80% | 13949963 |
| ASEAN | Edentulism | Incidence | Attributed to Age Structure Shift | 690871.7 | 62.10% | 1111641 |
| ASEAN | Edentulism | Incidence | Attributed to Population Growth | 560373.4 | 50.40% | 1111641 |
| ASEAN | Edentulism | Incidence | Attributed to Epidemiologic Changes | -139604 | -12.60% | 1111641 |
| ASEAN | Edentulism | DALYs | Attributed to Age Structure Shift | 243398.3 | 63.60% | 382641.2 |
| ASEAN | Edentulism | DALYs | Attributed to Population Growth | 187631.7 | 49.00% | 382641.2 |
| ASEAN | Edentulism | DALYs | Attributed to Epidemiologic Changes | -48388.8 | -12.60% | 382641.2 |
| ASEAN | Other oral disorders | Prevalence | Attributed to Age Structure Shift | 1106988 | 20.90% | 5294707 |
| ASEAN | Other oral disorders | Prevalence | Attributed to Population Growth | 4195025 | 79.20% | 5294707 |
| ASEAN | Other oral disorders | Prevalence | Attributed to Epidemiologic Changes | -7306.28 | -0.10% | 5294707 |
| ASEAN | Other oral disorders | Incidence | Attributed to Age Structure Shift | NA | NA% | NA |
| ASEAN | Other oral disorders | Incidence | Attributed to Population Growth | NA | NA% | NA |
| ASEAN | Other oral disorders | Incidence | Attributed to Epidemiologic Changes | NA | NA% | NA |
| ASEAN | Other oral disorders | DALYs | Attributed to Age Structure Shift | 30230.15 | 19.80% | 152661.4 |
| ASEAN | Other oral disorders | DALYs | Attributed to Population Growth | 121947.3 | 79.90% | 152661.4 |
| ASEAN | Other oral disorders | DALYs | Attributed to Epidemiologic Changes | 483.9598 | 0.30% | 152661.4 |
| Brunei | Main Oral disorders | Prevalence | Attributed to Age Structure Shift | 4121.419 | 6.40% | 64819 |
| Brunei | Main Oral disorders | Prevalence | Attributed to Population Growth | 64199.02 | 99.00% | 64819 |
| Brunei | Main Oral disorders | Prevalence | Attributed to Epidemiologic Changes | -3501.44 | -5.40% | 64819 |
| Brunei | Main Oral disorders | Incidence | Attributed to Age Structure Shift | -29274.3 | -37.60% | 77840.01 |
| Brunei | Main Oral disorders | Incidence | Attributed to Population Growth | 105899.7 | 136.00% | 77840.01 |
| Brunei | Main Oral disorders | Incidence | Attributed to Epidemiologic Changes | 1214.649 | 1.60% | 77840.01 |
| Brunei | Main Oral disorders | DALYs | Attributed to Age Structure Shift | 172.1604 | 39.80% | 432.3954 |
| Brunei | Main Oral disorders | DALYs | Attributed to Population Growth | 295.2336 | 68.30% | 432.3954 |
| Brunei | Main Oral disorders | DALYs | Attributed to Epidemiologic Changes | -34.9986 | -8.10% | 432.3954 |
| Brunei | Caries of deciduous teeth | Prevalence | Attributed to Age Structure Shift | -14581.5 | 9038.60% | -161.324 |
| Brunei | Caries of deciduous teeth | Prevalence | Attributed to Population Growth | 14473.25 | -8971.50% | -161.324 |
| Brunei | Caries of deciduous teeth | Prevalence | Attributed to Epidemiologic Changes | -53.1093 | 32.90% | -161.324 |
| Brunei | Caries of deciduous teeth | Incidence | Attributed to Age Structure Shift | -34954.8 | -1198.10% | 2917.618 |
| Brunei | Caries of deciduous teeth | Incidence | Attributed to Population Growth | 37579.64 | 1288.00% | 2917.618 |
| Brunei | Caries of deciduous teeth | Incidence | Attributed to Epidemiologic Changes | 292.7381 | 10.00% | 2917.618 |
| Brunei | Caries of deciduous teeth | DALYs | Attributed to Age Structure Shift | -5.58016 | 13159.70% | -0.0424 |
| Brunei | Caries of deciduous teeth | DALYs | Attributed to Population Growth | 5.53636 | ####### | -0.0424 |
| Brunei | Caries of deciduous teeth | DALYs | Attributed to Epidemiologic Changes | 0.001396 | -3.30% | -0.0424 |
| Brunei | Caries of permanent teeth | Prevalence | Attributed to Age Structure Shift | 4115.381 | 10.60% | 38673.49 |
| Brunei | Caries of permanent teeth | Prevalence | Attributed to Population Growth | 33636.58 | 87.00% | 38673.49 |
| Brunei | Caries of permanent teeth | Prevalence | Attributed to Epidemiologic Changes | 921.5215 | 2.40% | 38673.49 |
| Brunei | Caries of permanent teeth | Incidence | Attributed to Age Structure Shift | 4320.801 | 6.00% | 72205.83 |
| Brunei | Caries of permanent teeth | Incidence | Attributed to Population Growth | 66561.78 | 92.20% | 72205.83 |
| Brunei | Caries of permanent teeth | Incidence | Attributed to Epidemiologic Changes | 1323.251 | 1.80% | 72205.83 |
| Brunei | Caries of permanent teeth | DALYs | Attributed to Age Structure Shift | 3.829188 | 10.00% | 38.22209 |
| Brunei | Caries of permanent teeth | DALYs | Attributed to Population Growth | 33.40644 | 87.40% | 38.22209 |
| Brunei | Caries of permanent teeth | DALYs | Attributed to Epidemiologic Changes | 0.986459 | 2.60% | 38.22209 |
| Brunei | Periodontal diseases | Prevalence | Attributed to Age Structure Shift | 14612.53 | 58.70% | 24884.91 |
| Brunei | Periodontal diseases | Prevalence | Attributed to Population Growth | 15768.3 | 63.40% | 24884.91 |
| Brunei | Periodontal diseases | Prevalence | Attributed to Epidemiologic Changes | -5495.92 | -22.10% | 24884.91 |
| Brunei | Periodontal diseases | Incidence | Attributed to Age Structure Shift | 1121.245 | 49.30% | 2275.835 |
| Brunei | Periodontal diseases | Incidence | Attributed to Population Growth | 1560.599 | 68.60% | 2275.835 |
| Brunei | Periodontal diseases | Incidence | Attributed to Epidemiologic Changes | -406.008 | -17.80% | 2275.835 |
| Brunei | Periodontal diseases | DALYs | Attributed to Age Structure Shift | 94.46229 | 59.10% | 159.8182 |
| Brunei | Periodontal diseases | DALYs | Attributed to Population Growth | 102.6365 | 64.20% | 159.8182 |
| Brunei | Periodontal diseases | DALYs | Attributed to Epidemiologic Changes | -37.2806 | -23.30% | 159.8182 |
| Brunei | Edentulism | Prevalence | Attributed to Age Structure Shift | 2106.464 | 52.40% | 4018.231 |
| Brunei | Edentulism | Prevalence | Attributed to Population Growth | 1861.046 | 46.30% | 4018.231 |
| Brunei | Edentulism | Prevalence | Attributed to Epidemiologic Changes | 50.72174 | 1.30% | 4018.231 |
| Brunei | Edentulism | Incidence | Attributed to Age Structure Shift | 238.3782 | 54.10% | 440.7234 |
| Brunei | Edentulism | Incidence | Attributed to Population Growth | 197.6777 | 44.90% | 440.7234 |
| Brunei | Edentulism | Incidence | Attributed to Epidemiologic Changes | 4.667493 | 1.10% | 440.7234 |
| Brunei | Edentulism | DALYs | Attributed to Age Structure Shift | 57.54381 | 52.60% | 109.301 |
| Brunei | Edentulism | DALYs | Attributed to Population Growth | 50.59724 | 46.30% | 109.301 |
| Brunei | Edentulism | DALYs | Attributed to Epidemiologic Changes | 1.159949 | 1.10% | 109.301 |
| Brunei | Other oral disorders | Prevalence | Attributed to Age Structure Shift | 799.9086 | 18.40% | 4349.23 |
| Brunei | Other oral disorders | Prevalence | Attributed to Population Growth | 3536.06 | 81.30% | 4349.23 |
| Brunei | Other oral disorders | Prevalence | Attributed to Epidemiologic Changes | 13.26153 | 0.30% | 4349.23 |
| Brunei | Other oral disorders | Incidence | Attributed to Age Structure Shift | NA | NA% | NA |
| Brunei | Other oral disorders | Incidence | Attributed to Population Growth | NA | NA% | NA |
| Brunei | Other oral disorders | Incidence | Attributed to Epidemiologic Changes | NA | NA% | NA |
| Brunei | Other oral disorders | DALYs | Attributed to Age Structure Shift | 21.90527 | 17.50% | 125.0965 |
| Brunei | Other oral disorders | DALYs | Attributed to Population Growth | 103.057 | 82.40% | 125.0965 |
| Brunei | Other oral disorders | DALYs | Attributed to Epidemiologic Changes | 0.134244 | 0.10% | 125.0965 |
| Cambodia | Main Oral disorders | Prevalence | Attributed to Age Structure Shift | 354361.4 | 9.90% | 3594348 |
| Cambodia | Main Oral disorders | Prevalence | Attributed to Population Growth | 3434666 | 95.60% | 3594348 |
| Cambodia | Main Oral disorders | Prevalence | Attributed to Epidemiologic Changes | -194680 | -5.40% | 3594348 |
| Cambodia | Main Oral disorders | Incidence | Attributed to Age Structure Shift | -985745 | -28.40% | 3476720 |
| Cambodia | Main Oral disorders | Incidence | Attributed to Population Growth | 4290726 | 123.40% | 3476720 |
| Cambodia | Main Oral disorders | Incidence | Attributed to Epidemiologic Changes | 171739.2 | 4.90% | 3476720 |
| Cambodia | Main Oral disorders | DALYs | Attributed to Age Structure Shift | 8409.064 | 39.30% | 21414.44 |
| Cambodia | Main Oral disorders | DALYs | Attributed to Population Growth | 13515.45 | 63.10% | 21414.44 |
| Cambodia | Main Oral disorders | DALYs | Attributed to Epidemiologic Changes | -510.073 | -2.40% | 21414.44 |
| Cambodia | Caries of deciduous teeth | Prevalence | Attributed to Age Structure Shift | -744987 | -1543.70% | 48258.48 |
| Cambodia | Caries of deciduous teeth | Prevalence | Attributed to Population Growth | 812457.5 | 1683.60% | 48258.48 |
| Cambodia | Caries of deciduous teeth | Prevalence | Attributed to Epidemiologic Changes | -19212.1 | -39.80% | 48258.48 |
| Cambodia | Caries of deciduous teeth | Incidence | Attributed to Age Structure Shift | -1469419 | -502.20% | 292585 |
| Cambodia | Caries of deciduous teeth | Incidence | Attributed to Population Growth | 1771016 | 605.30% | 292585 |
| Cambodia | Caries of deciduous teeth | Incidence | Attributed to Epidemiologic Changes | -9011.14 | -3.10% | 292585 |
| Cambodia | Caries of deciduous teeth | DALYs | Attributed to Age Structure Shift | -283.956 | -1220.10% | 23.27357 |
| Cambodia | Caries of deciduous teeth | DALYs | Attributed to Population Growth | 309.5787 | 1330.20% | 23.27357 |
| Cambodia | Caries of deciduous teeth | DALYs | Attributed to Epidemiologic Changes | -2.34902 | -10.10% | 23.27357 |
| Cambodia | Caries of permanent teeth | Prevalence | Attributed to Age Structure Shift | 760366.7 | 27.10% | 2808139 |
| Cambodia | Caries of permanent teeth | Prevalence | Attributed to Population Growth | 2355754 | 83.90% | 2808139 |
| Cambodia | Caries of permanent teeth | Prevalence | Attributed to Epidemiologic Changes | -307982 | -11.00% | 2808139 |
| Cambodia | Caries of permanent teeth | Incidence | Attributed to Age Structure Shift | 436410.5 | 14.20% | 3067266 |
| Cambodia | Caries of permanent teeth | Incidence | Attributed to Population Growth | 2460864 | 80.20% | 3067266 |
| Cambodia | Caries of permanent teeth | Incidence | Attributed to Epidemiologic Changes | 169991.7 | 5.50% | 3067266 |
| Cambodia | Caries of permanent teeth | DALYs | Attributed to Age Structure Shift | 728.0013 | 26.30% | 2765.095 |
| Cambodia | Caries of permanent teeth | DALYs | Attributed to Population Growth | 2324.431 | 84.10% | 2765.095 |
| Cambodia | Caries of permanent teeth | DALYs | Attributed to Epidemiologic Changes | -287.338 | -10.40% | 2765.095 |
| Cambodia | Periodontal diseases | Prevalence | Attributed to Age Structure Shift | 384053.5 | 37.00% | 1038693 |
| Cambodia | Periodontal diseases | Prevalence | Attributed to Population Growth | 453297.8 | 43.60% | 1038693 |
| Cambodia | Periodontal diseases | Prevalence | Attributed to Epidemiologic Changes | 201342.2 | 19.40% | 1038693 |
| Cambodia | Periodontal diseases | Incidence | Attributed to Age Structure Shift | 33778.15 | 35.80% | 94297.33 |
| Cambodia | Periodontal diseases | Incidence | Attributed to Population Growth | 45066.32 | 47.80% | 94297.33 |
| Cambodia | Periodontal diseases | Incidence | Attributed to Epidemiologic Changes | 15452.86 | 16.40% | 94297.33 |
| Cambodia | Periodontal diseases | DALYs | Attributed to Age Structure Shift | 2484.984 | 36.70% | 6763.413 |
| Cambodia | Periodontal diseases | DALYs | Attributed to Population Growth | 2947.077 | 43.60% | 6763.413 |
| Cambodia | Periodontal diseases | DALYs | Attributed to Epidemiologic Changes | 1331.352 | 19.70% | 6763.413 |
| Cambodia | Edentulism | Prevalence | Attributed to Age Structure Shift | 168914.6 | 61.20% | 276205.3 |
| Cambodia | Edentulism | Prevalence | Attributed to Population Growth | 165509.4 | 59.90% | 276205.3 |
| Cambodia | Edentulism | Prevalence | Attributed to Epidemiologic Changes | -58218.7 | -21.10% | 276205.3 |
| Cambodia | Edentulism | Incidence | Attributed to Age Structure Shift | 13485.79 | 59.70% | 22571.67 |
| Cambodia | Edentulism | Incidence | Attributed to Population Growth | 13780.13 | 61.10% | 22571.67 |
| Cambodia | Edentulism | Incidence | Attributed to Epidemiologic Changes | -4694.26 | -20.80% | 22571.67 |
| Cambodia | Edentulism | DALYs | Attributed to Age Structure Shift | 4607.76 | 61.00% | 7553.544 |
| Cambodia | Edentulism | DALYs | Attributed to Population Growth | 4534.474 | 60.00% | 7553.544 |
| Cambodia | Edentulism | DALYs | Attributed to Epidemiologic Changes | -1588.69 | -21.00% | 7553.544 |
| Cambodia | Other oral disorders | Prevalence | Attributed to Age Structure Shift | 31321.94 | 21.20% | 147891.4 |
| Cambodia | Other oral disorders | Prevalence | Attributed to Population Growth | 117068.3 | 79.20% | 147891.4 |
| Cambodia | Other oral disorders | Prevalence | Attributed to Epidemiologic Changes | -498.921 | -0.30% | 147891.4 |
| Cambodia | Other oral disorders | Incidence | Attributed to Age Structure Shift | NA | NA% | NA |
| Cambodia | Other oral disorders | Incidence | Attributed to Population Growth | NA | NA% | NA |
| Cambodia | Other oral disorders | Incidence | Attributed to Epidemiologic Changes | NA | NA% | NA |
| Cambodia | Other oral disorders | DALYs | Attributed to Age Structure Shift | 872.275 | 20.20% | 4309.117 |
| Cambodia | Other oral disorders | DALYs | Attributed to Population Growth | 3399.891 | 78.90% | 4309.117 |
| Cambodia | Other oral disorders | DALYs | Attributed to Epidemiologic Changes | 36.95166 | 0.90% | 4309.117 |
| Indonesia | Main Oral disorders | Prevalence | Attributed to Age Structure Shift | 7687386 | 14.80% | 51907084 |
| Indonesia | Main Oral disorders | Prevalence | Attributed to Population Growth | 46274435 | 89.10% | 51907084 |
| Indonesia | Main Oral disorders | Prevalence | Attributed to Epidemiologic Changes | -2054736 | -4.00% | 51907084 |
| Indonesia | Main Oral disorders | Incidence | Attributed to Age Structure Shift | -1.8E+07 | -44.50% | 40633603 |
| Indonesia | Main Oral disorders | Incidence | Attributed to Population Growth | 57745725 | 142.10% | 40633603 |
| Indonesia | Main Oral disorders | Incidence | Attributed to Epidemiologic Changes | 985121.2 | 2.40% | 40633603 |
| Indonesia | Main Oral disorders | DALYs | Attributed to Age Structure Shift | 175581.3 | 44.10% | 398265.1 |
| Indonesia | Main Oral disorders | DALYs | Attributed to Population Growth | 230929.4 | 58.00% | 398265.1 |
| Indonesia | Main Oral disorders | DALYs | Attributed to Epidemiologic Changes | -8245.56 | -2.10% | 398265.1 |
| Indonesia | Caries of deciduous teeth | Prevalence | Attributed to Age Structure Shift | -9470464 | 12392.30% | -76422.1 |
| Indonesia | Caries of deciduous teeth | Prevalence | Attributed to Population Growth | 9031966 | ####### | -76422.1 |
| Indonesia | Caries of deciduous teeth | Prevalence | Attributed to Epidemiologic Changes | 362075.7 | -473.80% | -76422.1 |
| Indonesia | Caries of deciduous teeth | Incidence | Attributed to Age Structure Shift | -2.2E+07 | 6856.50% | -315896 |
| Indonesia | Caries of deciduous teeth | Incidence | Attributed to Population Growth | 20857482 | -6602.60% | -315896 |
| Indonesia | Caries of deciduous teeth | Incidence | Attributed to Epidemiologic Changes | 486025.9 | -153.90% | -315896 |
| Indonesia | Caries of deciduous teeth | DALYs | Attributed to Age Structure Shift | -3617.26 | ####### | 1.434017 |
| Indonesia | Caries of deciduous teeth | DALYs | Attributed to Population Growth | 3448.872 | ####### | 1.434017 |
| Indonesia | Caries of deciduous teeth | DALYs | Attributed to Epidemiologic Changes | 169.8229 | 11842.50% | 1.434017 |
| Indonesia | Caries of permanent teeth | Prevalence | Attributed to Age Structure Shift | 8668447 | 25.10% | 34524458 |
| Indonesia | Caries of permanent teeth | Prevalence | Attributed to Population Growth | 29176503 | 84.50% | 34524458 |
| Indonesia | Caries of permanent teeth | Prevalence | Attributed to Epidemiologic Changes | -3320491 | -9.60% | 34524458 |
| Indonesia | Caries of permanent teeth | Incidence | Attributed to Age Structure Shift | 2583567 | 6.70% | 38743043 |
| Indonesia | Caries of permanent teeth | Incidence | Attributed to Population Growth | 35655478 | 92.00% | 38743043 |
| Indonesia | Caries of permanent teeth | Incidence | Attributed to Epidemiologic Changes | 503997.7 | 1.30% | 38743043 |
| Indonesia | Caries of permanent teeth | DALYs | Attributed to Age Structure Shift | 8203.111 | 24.20% | 33835.26 |
| Indonesia | Caries of permanent teeth | DALYs | Attributed to Population Growth | 28816.2 | 85.20% | 33835.26 |
| Indonesia | Caries of permanent teeth | DALYs | Attributed to Epidemiologic Changes | -3184.05 | -9.40% | 33835.26 |
| Indonesia | Periodontal diseases | Prevalence | Attributed to Age Structure Shift | 10812110 | 46.80% | 23120440 |
| Indonesia | Periodontal diseases | Prevalence | Attributed to Population Growth | 11426961 | 49.40% | 23120440 |
| Indonesia | Periodontal diseases | Prevalence | Attributed to Epidemiologic Changes | 881369.3 | 3.80% | 23120440 |
| Indonesia | Periodontal diseases | Incidence | Attributed to Age Structure Shift | 718080.2 | 40.90% | 1756778 |
| Indonesia | Periodontal diseases | Incidence | Attributed to Population Growth | 1007668 | 57.40% | 1756778 |
| Indonesia | Periodontal diseases | Incidence | Attributed to Epidemiologic Changes | 31030.64 | 1.80% | 1756778 |
| Indonesia | Periodontal diseases | DALYs | Attributed to Age Structure Shift | 70331.56 | 46.50% | 151088.7 |
| Indonesia | Periodontal diseases | DALYs | Attributed to Population Growth | 74705.84 | 49.40% | 151088.7 |
| Indonesia | Periodontal diseases | DALYs | Attributed to Epidemiologic Changes | 6051.315 | 4.00% | 151088.7 |
| Indonesia | Edentulism | Prevalence | Attributed to Age Structure Shift | 3191383 | 58.60% | 5444922 |
| Indonesia | Edentulism | Prevalence | Attributed to Population Growth | 2670184 | 49.00% | 5444922 |
| Indonesia | Edentulism | Prevalence | Attributed to Epidemiologic Changes | -416645 | -7.70% | 5444922 |
| Indonesia | Edentulism | Incidence | Attributed to Age Structure Shift | 260513.9 | 57.90% | 449678.2 |
| Indonesia | Edentulism | Incidence | Attributed to Population Growth | 225097.4 | 50.10% | 449678.2 |
| Indonesia | Edentulism | Incidence | Attributed to Epidemiologic Changes | -35933.2 | -8.00% | 449678.2 |
| Indonesia | Edentulism | DALYs | Attributed to Age Structure Shift | 87801.33 | 58.50% | 150091.4 |
| Indonesia | Edentulism | DALYs | Attributed to Population Growth | 73614.84 | 49.00% | 150091.4 |
| Indonesia | Edentulism | DALYs | Attributed to Epidemiologic Changes | -11324.7 | -7.50% | 150091.4 |
| Indonesia | Other oral disorders | Prevalence | Attributed to Age Structure Shift | 467349.6 | 21.30% | 2190826 |
| Indonesia | Other oral disorders | Prevalence | Attributed to Population Growth | 1729872 | 79.00% | 2190826 |
| Indonesia | Other oral disorders | Prevalence | Attributed to Epidemiologic Changes | -6395.35 | -0.30% | 2190826 |
| Indonesia | Other oral disorders | Incidence | Attributed to Age Structure Shift | NA | NA% | NA |
| Indonesia | Other oral disorders | Incidence | Attributed to Population Growth | NA | NA% | NA |
| Indonesia | Other oral disorders | Incidence | Attributed to Epidemiologic Changes | NA | NA% | NA |
| Indonesia | Other oral disorders | DALYs | Attributed to Age Structure Shift | 12862.56 | 20.30% | 63248.28 |
| Indonesia | Other oral disorders | DALYs | Attributed to Population Growth | 50343.62 | 79.60% | 63248.28 |
| Indonesia | Other oral disorders | DALYs | Attributed to Epidemiologic Changes | 42.09153 | 0.10% | 63248.28 |
| Laos | Main Oral disorders | Prevalence | Attributed to Age Structure Shift | 29774.59 | 2.30% | 1310800 |
| Laos | Main Oral disorders | Prevalence | Attributed to Population Growth | 1409119 | 107.50% | 1310800 |
| Laos | Main Oral disorders | Prevalence | Attributed to Epidemiologic Changes | -128094 | -9.80% | 1310800 |
| Laos | Main Oral disorders | Incidence | Attributed to Age Structure Shift | -349400 | -21.60% | 1616688 |
| Laos | Main Oral disorders | Incidence | Attributed to Population Growth | 1916236 | 118.50% | 1616688 |
| Laos | Main Oral disorders | Incidence | Attributed to Epidemiologic Changes | 49853.05 | 3.10% | 1616688 |
| Laos | Main Oral disorders | DALYs | Attributed to Age Structure Shift | 1413.174 | 24.00% | 5899.706 |
| Laos | Main Oral disorders | DALYs | Attributed to Population Growth | 4749.741 | 80.50% | 5899.706 |
| Laos | Main Oral disorders | DALYs | Attributed to Epidemiologic Changes | -263.208 | -4.50% | 5899.706 |
| Laos | Caries of deciduous teeth | Prevalence | Attributed to Age Structure Shift | -256802 | -224.80% | 114235.9 |
| Laos | Caries of deciduous teeth | Prevalence | Attributed to Population Growth | 383791.2 | 336.00% | 114235.9 |
| Laos | Caries of deciduous teeth | Prevalence | Attributed to Epidemiologic Changes | -12753.5 | -11.20% | 114235.9 |
| Laos | Caries of deciduous teeth | Incidence | Attributed to Age Structure Shift | -530865 | -163.90% | 323879.4 |
| Laos | Caries of deciduous teeth | Incidence | Attributed to Population Growth | 841259.3 | 259.70% | 323879.4 |
| Laos | Caries of deciduous teeth | Incidence | Attributed to Epidemiologic Changes | 13485.47 | 4.20% | 323879.4 |
| Laos | Caries of deciduous teeth | DALYs | Attributed to Age Structure Shift | -98.0779 | -222.60% | 44.06126 |
| Laos | Caries of deciduous teeth | DALYs | Attributed to Population Growth | 146.5844 | 332.70% | 44.06126 |
| Laos | Caries of deciduous teeth | DALYs | Attributed to Epidemiologic Changes | -4.44522 | -10.10% | 44.06126 |
| Laos | Caries of permanent teeth | Prevalence | Attributed to Age Structure Shift | 215002.3 | 21.90% | 979619.3 |
| Laos | Caries of permanent teeth | Prevalence | Attributed to Population Growth | 931574.5 | 95.10% | 979619.3 |
| Laos | Caries of permanent teeth | Prevalence | Attributed to Epidemiologic Changes | -166957 | -17.00% | 979619.3 |
| Laos | Caries of permanent teeth | Incidence | Attributed to Age Structure Shift | 174012.3 | 13.80% | 1264890 |
| Laos | Caries of permanent teeth | Incidence | Attributed to Population Growth | 1059390 | 83.80% | 1264890 |
| Laos | Caries of permanent teeth | Incidence | Attributed to Epidemiologic Changes | 31487.84 | 2.50% | 1264890 |
| Laos | Caries of permanent teeth | DALYs | Attributed to Age Structure Shift | 209.3821 | 21.50% | 972.9758 |
| Laos | Caries of permanent teeth | DALYs | Attributed to Population Growth | 922.2752 | 94.80% | 972.9758 |
| Laos | Caries of permanent teeth | DALYs | Attributed to Epidemiologic Changes | -158.682 | -16.30% | 972.9758 |
| Laos | Periodontal diseases | Prevalence | Attributed to Age Structure Shift | 56724.7 | 23.70% | 239106.5 |
| Laos | Periodontal diseases | Prevalence | Attributed to Population Growth | 110009.3 | 46.00% | 239106.5 |
| Laos | Periodontal diseases | Prevalence | Attributed to Epidemiologic Changes | 72372.53 | 30.30% | 239106.5 |
| Laos | Periodontal diseases | Incidence | Attributed to Age Structure Shift | 5493.382 | 23.40% | 23445.53 |
| Laos | Periodontal diseases | Incidence | Attributed to Population Growth | 11276.89 | 48.10% | 23445.53 |
| Laos | Periodontal diseases | Incidence | Attributed to Epidemiologic Changes | 6675.256 | 28.50% | 23445.53 |
| Laos | Periodontal diseases | DALYs | Attributed to Age Structure Shift | 370.2133 | 23.70% | 1562.872 |
| Laos | Periodontal diseases | DALYs | Attributed to Population Growth | 717.283 | 45.90% | 1562.872 |
| Laos | Periodontal diseases | DALYs | Attributed to Epidemiologic Changes | 475.3753 | 30.40% | 1562.872 |
| Laos | Edentulism | Prevalence | Attributed to Age Structure Shift | 22835.78 | 44.90% | 50845.22 |
| Laos | Edentulism | Prevalence | Attributed to Population Growth | 49198.77 | 96.80% | 50845.22 |
| Laos | Edentulism | Prevalence | Attributed to Epidemiologic Changes | -21189.3 | -41.70% | 50845.22 |
| Laos | Edentulism | Incidence | Attributed to Age Structure Shift | 1959.249 | 43.80% | 4473.422 |
| Laos | Edentulism | Incidence | Attributed to Population Growth | 4309.688 | 96.30% | 4473.422 |
| Laos | Edentulism | Incidence | Attributed to Epidemiologic Changes | -1795.51 | -40.10% | 4473.422 |
| Laos | Edentulism | DALYs | Attributed to Age Structure Shift | 628.7083 | 44.80% | 1402.443 |
| Laos | Edentulism | DALYs | Attributed to Population Growth | 1356.558 | 96.70% | 1402.443 |
| Laos | Edentulism | DALYs | Attributed to Epidemiologic Changes | -582.823 | -41.60% | 1402.443 |
| Laos | Other oral disorders | Prevalence | Attributed to Age Structure Shift | 10673.29 | 16.20% | 65806.25 |
| Laos | Other oral disorders | Prevalence | Attributed to Population Growth | 55199.84 | 83.90% | 65806.25 |
| Laos | Other oral disorders | Prevalence | Attributed to Epidemiologic Changes | -66.8711 | -0.10% | 65806.25 |
| Laos | Other oral disorders | Incidence | Attributed to Age Structure Shift | NA | NA% | NA |
| Laos | Other oral disorders | Incidence | Attributed to Population Growth | NA | NA% | NA |
| Laos | Other oral disorders | Incidence | Attributed to Epidemiologic Changes | NA | NA% | NA |
| Laos | Other oral disorders | DALYs | Attributed to Age Structure Shift | 302.9482 | 15.80% | 1917.354 |
| Laos | Other oral disorders | DALYs | Attributed to Population Growth | 1607.04 | 83.80% | 1917.354 |
| Laos | Other oral disorders | DALYs | Attributed to Epidemiologic Changes | 7.365831 | 0.40% | 1917.354 |
| Malaysia | Main Oral disorders | Prevalence | Attributed to Age Structure Shift | 490031.7 | 9.10% | 5361581 |
| Malaysia | Main Oral disorders | Prevalence | Attributed to Population Growth | 6065978 | 113.10% | 5361581 |
| Malaysia | Main Oral disorders | Prevalence | Attributed to Epidemiologic Changes | -1194429 | -22.30% | 5361581 |
| Malaysia | Main Oral disorders | Incidence | Attributed to Age Structure Shift | -1800510 | -31.90% | 5647953 |
| Malaysia | Main Oral disorders | Incidence | Attributed to Population Growth | 6659561 | 117.90% | 5647953 |
| Malaysia | Main Oral disorders | Incidence | Attributed to Epidemiologic Changes | 788901.8 | 14.00% | 5647953 |
| Malaysia | Main Oral disorders | DALYs | Attributed to Age Structure Shift | 19216.51 | 45.70% | 42053.73 |
| Malaysia | Main Oral disorders | DALYs | Attributed to Population Growth | 33508.1 | 79.70% | 42053.73 |
| Malaysia | Main Oral disorders | DALYs | Attributed to Epidemiologic Changes | -10670.9 | -25.40% | 42053.73 |
| Malaysia | Caries of deciduous teeth | Prevalence | Attributed to Age Structure Shift | -1077071 | -1057.30% | 101873.6 |
| Malaysia | Caries of deciduous teeth | Prevalence | Attributed to Population Growth | 1308274 | 1284.20% | 101873.6 |
| Malaysia | Caries of deciduous teeth | Prevalence | Attributed to Epidemiologic Changes | -129330 | -127.00% | 101873.6 |
| Malaysia | Caries of deciduous teeth | Incidence | Attributed to Age Structure Shift | -2147115 | -151.20% | 1420217 |
| Malaysia | Caries of deciduous teeth | Incidence | Attributed to Population Growth | 2742238 | 193.10% | 1420217 |
| Malaysia | Caries of deciduous teeth | Incidence | Attributed to Epidemiologic Changes | 825093.8 | 58.10% | 1420217 |
| Malaysia | Caries of deciduous teeth | DALYs | Attributed to Age Structure Shift | -411.646 | -993.10% | 41.44998 |
| Malaysia | Caries of deciduous teeth | DALYs | Attributed to Population Growth | 499.8939 | 1206.00% | 41.44998 |
| Malaysia | Caries of deciduous teeth | DALYs | Attributed to Epidemiologic Changes | -46.798 | -112.90% | 41.44998 |
| Malaysia | Caries of permanent teeth | Prevalence | Attributed to Age Structure Shift | 715168.4 | 19.00% | 3755038 |
| Malaysia | Caries of permanent teeth | Prevalence | Attributed to Population Growth | 3612887 | 96.20% | 3755038 |
| Malaysia | Caries of permanent teeth | Prevalence | Attributed to Epidemiologic Changes | -573018 | -15.30% | 3755038 |
| Malaysia | Caries of permanent teeth | Incidence | Attributed to Age Structure Shift | 246864.6 | 6.10% | 4043997 |
| Malaysia | Caries of permanent teeth | Incidence | Attributed to Population Growth | 3759592 | 93.00% | 4043997 |
| Malaysia | Caries of permanent teeth | Incidence | Attributed to Epidemiologic Changes | 37540.2 | 0.90% | 4043997 |
| Malaysia | Caries of permanent teeth | DALYs | Attributed to Age Structure Shift | 678.6628 | 18.40% | 3694.813 |
| Malaysia | Caries of permanent teeth | DALYs | Attributed to Population Growth | 3573.385 | 96.70% | 3694.813 |
| Malaysia | Caries of permanent teeth | DALYs | Attributed to Epidemiologic Changes | -557.236 | -15.10% | 3694.813 |
| Malaysia | Periodontal diseases | Prevalence | Attributed to Age Structure Shift | 730310 | 68.90% | 1059660 |
| Malaysia | Periodontal diseases | Prevalence | Attributed to Population Growth | 1144796 | 108.00% | 1059660 |
| Malaysia | Periodontal diseases | Prevalence | Attributed to Epidemiologic Changes | -815446 | -77.00% | 1059660 |
| Malaysia | Periodontal diseases | Incidence | Attributed to Age Structure Shift | 64319.94 | 54.30% | 118378.7 |
| Malaysia | Periodontal diseases | Incidence | Attributed to Population Growth | 116290.8 | 98.20% | 118378.7 |
| Malaysia | Periodontal diseases | Incidence | Attributed to Epidemiologic Changes | -62232 | -52.60% | 118378.7 |
| Malaysia | Periodontal diseases | DALYs | Attributed to Age Structure Shift | 4709.492 | 70.00% | 6726.053 |
| Malaysia | Periodontal diseases | DALYs | Attributed to Population Growth | 7446.553 | 110.70% | 6726.053 |
| Malaysia | Periodontal diseases | DALYs | Attributed to Epidemiologic Changes | -5429.99 | -80.70% | 6726.053 |
| Malaysia | Edentulism | Prevalence | Attributed to Age Structure Shift | 471960.1 | 56.50% | 835176.9 |
| Malaysia | Edentulism | Prevalence | Attributed to Population Growth | 529178.6 | 63.40% | 835176.9 |
| Malaysia | Edentulism | Prevalence | Attributed to Epidemiologic Changes | -165962 | -19.90% | 835176.9 |
| Malaysia | Edentulism | Incidence | Attributed to Age Structure Shift | 35420.16 | 54.20% | 65360.55 |
| Malaysia | Edentulism | Incidence | Attributed to Population Growth | 41440.52 | 63.40% | 65360.55 |
| Malaysia | Edentulism | Incidence | Attributed to Epidemiologic Changes | -11500.1 | -17.60% | 65360.55 |
| Malaysia | Edentulism | DALYs | Attributed to Age Structure Shift | 12888.24 | 56.60% | 22772.73 |
| Malaysia | Edentulism | DALYs | Attributed to Population Growth | 14513.04 | 63.70% | 22772.73 |
| Malaysia | Edentulism | DALYs | Attributed to Epidemiologic Changes | -4628.55 | -20.30% | 22772.73 |
| Malaysia | Other oral disorders | Prevalence | Attributed to Age Structure Shift | 49219.62 | 16.10% | 305989.6 |
| Malaysia | Other oral disorders | Prevalence | Attributed to Population Growth | 257340.9 | 84.10% | 305989.6 |
| Malaysia | Other oral disorders | Prevalence | Attributed to Epidemiologic Changes | -570.903 | -0.20% | 305989.6 |
| Malaysia | Other oral disorders | Incidence | Attributed to Age Structure Shift | NA | NA% | NA |
| Malaysia | Other oral disorders | Incidence | Attributed to Population Growth | NA | NA% | NA |
| Malaysia | Other oral disorders | Incidence | Attributed to Epidemiologic Changes | NA | NA% | NA |
| Malaysia | Other oral disorders | DALYs | Attributed to Age Structure Shift | 1351.761 | 15.30% | 8818.686 |
| Malaysia | Other oral disorders | DALYs | Attributed to Population Growth | 7475.23 | 84.80% | 8818.686 |
| Malaysia | Other oral disorders | DALYs | Attributed to Epidemiologic Changes | -8.30589 | -0.10% | 8818.686 |
| Myanmar | Main Oral disorders | Prevalence | Attributed to Age Structure Shift | 690074.6 | 13.80% | 4996248 |
| Myanmar | Main Oral disorders | Prevalence | Attributed to Population Growth | 6288010 | 125.90% | 4996248 |
| Myanmar | Main Oral disorders | Prevalence | Attributed to Epidemiologic Changes | -1981836 | -39.70% | 4996248 |
| Myanmar | Main Oral disorders | Incidence | Attributed to Age Structure Shift | -2777659 | -47.50% | 5850766 |
| Myanmar | Main Oral disorders | Incidence | Attributed to Population Growth | 9113928 | 155.80% | 5850766 |
| Myanmar | Main Oral disorders | Incidence | Attributed to Epidemiologic Changes | -485502 | -8.30% | 5850766 |
| Myanmar | Main Oral disorders | DALYs | Attributed to Age Structure Shift | 19288.59 | 56.00% | 34444.47 |
| Myanmar | Main Oral disorders | DALYs | Attributed to Population Growth | 27485.18 | 79.80% | 34444.47 |
| Myanmar | Main Oral disorders | DALYs | Attributed to Epidemiologic Changes | -12329.3 | -35.80% | 34444.47 |
| Myanmar | Caries of deciduous teeth | Prevalence | Attributed to Age Structure Shift | -1171449 | -4155.40% | 28191.33 |
| Myanmar | Caries of deciduous teeth | Prevalence | Attributed to Population Growth | 1348878 | 4784.70% | 28191.33 |
| Myanmar | Caries of deciduous teeth | Prevalence | Attributed to Epidemiologic Changes | -149237 | -529.40% | 28191.33 |
| Myanmar | Caries of deciduous teeth | Incidence | Attributed to Age Structure Shift | -3065165 | -747.90% | 409828.8 |
| Myanmar | Caries of deciduous teeth | Incidence | Attributed to Population Growth | 3587156 | 875.30% | 409828.8 |
| Myanmar | Caries of deciduous teeth | Incidence | Attributed to Epidemiologic Changes | -112162 | -27.40% | 409828.8 |
| Myanmar | Caries of deciduous teeth | DALYs | Attributed to Age Structure Shift | -447.219 | -1449.50% | 30.8528 |
| Myanmar | Caries of deciduous teeth | DALYs | Attributed to Population Growth | 514.6103 | 1668.00% | 30.8528 |
| Myanmar | Caries of deciduous teeth | DALYs | Attributed to Epidemiologic Changes | -36.5383 | -118.40% | 30.8528 |
| Myanmar | Caries of permanent teeth | Prevalence | Attributed to Age Structure Shift | 811814 | 24.30% | 3346043 |
| Myanmar | Caries of permanent teeth | Prevalence | Attributed to Population Growth | 4000356 | 119.60% | 3346043 |
| Myanmar | Caries of permanent teeth | Prevalence | Attributed to Epidemiologic Changes | -1466127 | -43.80% | 3346043 |
| Myanmar | Caries of permanent teeth | Incidence | Attributed to Age Structure Shift | 167859.9 | 3.20% | 5267800 |
| Myanmar | Caries of permanent teeth | Incidence | Attributed to Population Growth | 5401111 | 102.50% | 5267800 |
| Myanmar | Caries of permanent teeth | Incidence | Attributed to Epidemiologic Changes | -301171 | -5.70% | 5267800 |
| Myanmar | Caries of permanent teeth | DALYs | Attributed to Age Structure Shift | 753.4427 | 22.80% | 3299.572 |
| Myanmar | Caries of permanent teeth | DALYs | Attributed to Population Growth | 3951.638 | 119.80% | 3299.572 |
| Myanmar | Caries of permanent teeth | DALYs | Attributed to Epidemiologic Changes | -1405.51 | -42.60% | 3299.572 |
| Myanmar | Periodontal diseases | Prevalence | Attributed to Age Structure Shift | 1029319 | 80.20% | 1283864 |
| Myanmar | Periodontal diseases | Prevalence | Attributed to Population Growth | 992425.2 | 77.30% | 1283864 |
| Myanmar | Periodontal diseases | Prevalence | Attributed to Epidemiologic Changes | -737880 | -57.50% | 1283864 |
| Myanmar | Periodontal diseases | Incidence | Attributed to Age Structure Shift | 88711.56 | 65.70% | 135067.4 |
| Myanmar | Periodontal diseases | Incidence | Attributed to Population Growth | 99834.25 | 73.90% | 135067.4 |
| Myanmar | Periodontal diseases | Incidence | Attributed to Epidemiologic Changes | -53478.4 | -39.60% | 135067.4 |
| Myanmar | Periodontal diseases | DALYs | Attributed to Age Structure Shift | 6597.096 | 81.80% | 8066.281 |
| Myanmar | Periodontal diseases | DALYs | Attributed to Population Growth | 6412.23 | 79.50% | 8066.281 |
| Myanmar | Periodontal diseases | DALYs | Attributed to Epidemiologic Changes | -4943.04 | -61.30% | 8066.281 |
| Myanmar | Edentulism | Prevalence | Attributed to Age Structure Shift | 387211.5 | 83.10% | 466183.2 |
| Myanmar | Edentulism | Prevalence | Attributed to Population Growth | 301366.6 | 64.60% | 466183.2 |
| Myanmar | Edentulism | Prevalence | Attributed to Epidemiologic Changes | -222395 | -47.70% | 466183.2 |
| Myanmar | Edentulism | Incidence | Attributed to Age Structure Shift | 30934.24 | 81.30% | 38069.71 |
| Myanmar | Edentulism | Incidence | Attributed to Population Growth | 25826.53 | 67.80% | 38069.71 |
| Myanmar | Edentulism | Incidence | Attributed to Epidemiologic Changes | -18691.1 | -49.10% | 38069.71 |
| Myanmar | Edentulism | DALYs | Attributed to Age Structure Shift | 10490.33 | 82.80% | 12669.3 |
| Myanmar | Edentulism | DALYs | Attributed to Population Growth | 8245.577 | 65.10% | 12669.3 |
| Myanmar | Edentulism | DALYs | Attributed to Epidemiologic Changes | -6066.61 | -47.90% | 12669.3 |
| Myanmar | Other oral disorders | Prevalence | Attributed to Age Structure Shift | 69435.19 | 19.30% | 359426.8 |
| Myanmar | Other oral disorders | Prevalence | Attributed to Population Growth | 288125.2 | 80.20% | 359426.8 |
| Myanmar | Other oral disorders | Prevalence | Attributed to Epidemiologic Changes | 1866.433 | 0.50% | 359426.8 |
| Myanmar | Other oral disorders | Incidence | Attributed to Age Structure Shift | NA | NA% | NA |
| Myanmar | Other oral disorders | Incidence | Attributed to Population Growth | NA | NA% | NA |
| Myanmar | Other oral disorders | Incidence | Attributed to Epidemiologic Changes | NA | NA% | NA |
| Myanmar | Other oral disorders | DALYs | Attributed to Age Structure Shift | 1894.938 | 18.30% | 10378.46 |
| Myanmar | Other oral disorders | DALYs | Attributed to Population Growth | 8361.128 | 80.60% | 10378.46 |
| Myanmar | Other oral disorders | DALYs | Attributed to Epidemiologic Changes | 122.3958 | 1.20% | 10378.46 |
| Philippines | Main Oral disorders | Prevalence | Attributed to Age Structure Shift | 1606232 | 7.70% | 20988145 |
| Philippines | Main Oral disorders | Prevalence | Attributed to Population Growth | 23240127 | 110.70% | 20988145 |
| Philippines | Main Oral disorders | Prevalence | Attributed to Epidemiologic Changes | -3858213 | -18.40% | 20988145 |
| Philippines | Main Oral disorders | Incidence | Attributed to Age Structure Shift | -4852027 | -19.10% | 25372669 |
| Philippines | Main Oral disorders | Incidence | Attributed to Population Growth | 30832308 | 121.50% | 25372669 |
| Philippines | Main Oral disorders | Incidence | Attributed to Epidemiologic Changes | -607611 | -2.40% | 25372669 |
| Philippines | Main Oral disorders | DALYs | Attributed to Age Structure Shift | 50539.53 | 40.20% | 125768.6 |
| Philippines | Main Oral disorders | DALYs | Attributed to Population Growth | 108801.7 | 86.50% | 125768.6 |
| Philippines | Main Oral disorders | DALYs | Attributed to Epidemiologic Changes | -33572.7 | -26.70% | 125768.6 |
| Philippines | Caries of deciduous teeth | Prevalence | Attributed to Age Structure Shift | -3112922 | -167.00% | 1864106 |
| Philippines | Caries of deciduous teeth | Prevalence | Attributed to Population Growth | 5545856 | 297.50% | 1864106 |
| Philippines | Caries of deciduous teeth | Prevalence | Attributed to Epidemiologic Changes | -568827 | -30.50% | 1864106 |
| Philippines | Caries of deciduous teeth | Incidence | Attributed to Age Structure Shift | -6224325 | -110.10% | 5654322 |
| Philippines | Caries of deciduous teeth | Incidence | Attributed to Population Growth | 12494726 | 221.00% | 5654322 |
| Philippines | Caries of deciduous teeth | Incidence | Attributed to Epidemiologic Changes | -616080 | -10.90% | 5654322 |
| Philippines | Caries of deciduous teeth | DALYs | Attributed to Age Structure Shift | -1187.91 | -164.30% | 723.1242 |
| Philippines | Caries of deciduous teeth | DALYs | Attributed to Population Growth | 2116.457 | 292.70% | 723.1242 |
| Philippines | Caries of deciduous teeth | DALYs | Attributed to Epidemiologic Changes | -205.427 | -28.40% | 723.1242 |
| Philippines | Caries of permanent teeth | Prevalence | Attributed to Age Structure Shift | 3005147 | 17.20% | 17433063 |
| Philippines | Caries of permanent teeth | Prevalence | Attributed to Population Growth | 15167160 | 87.00% | 17433063 |
| Philippines | Caries of permanent teeth | Prevalence | Attributed to Epidemiologic Changes | -739244 | -4.20% | 17433063 |
| Philippines | Caries of permanent teeth | Incidence | Attributed to Age Structure Shift | 1141254 | 5.80% | 19536519 |
| Philippines | Caries of permanent teeth | Incidence | Attributed to Population Growth | 17908220 | 91.70% | 19536519 |
| Philippines | Caries of permanent teeth | Incidence | Attributed to Epidemiologic Changes | 487044.6 | 2.50% | 19536519 |
| Philippines | Caries of permanent teeth | DALYs | Attributed to Age Structure Shift | 2860.228 | 16.70% | 17152.98 |
| Philippines | Caries of permanent teeth | DALYs | Attributed to Population Growth | 14966.64 | 87.30% | 17152.98 |
| Philippines | Caries of permanent teeth | DALYs | Attributed to Epidemiologic Changes | -673.888 | -3.90% | 17152.98 |
| Philippines | Periodontal diseases | Prevalence | Attributed to Age Structure Shift | 1556428 | -198.40% | -784535 |
| Philippines | Periodontal diseases | Prevalence | Attributed to Population Growth | 2823112 | -359.80% | -784535 |
| Philippines | Periodontal diseases | Prevalence | Attributed to Epidemiologic Changes | -5164075 | 658.20% | -784535 |
| Philippines | Periodontal diseases | Incidence | Attributed to Age Structure Shift | 133934.7 | -257.60% | -52001 |
| Philippines | Periodontal diseases | Incidence | Attributed to Population Growth | 290620.4 | -558.90% | -52001 |
| Philippines | Periodontal diseases | Incidence | Attributed to Epidemiologic Changes | -476556 | 916.40% | -52001 |
| Philippines | Periodontal diseases | DALYs | Attributed to Age Structure Shift | 10049.48 | -190.40% | -5279.07 |
| Philippines | Periodontal diseases | DALYs | Attributed to Population Growth | 18358.69 | -347.80% | -5279.07 |
| Philippines | Periodontal diseases | DALYs | Attributed to Epidemiologic Changes | -33687.2 | 638.10% | -5279.07 |
| Philippines | Edentulism | Prevalence | Attributed to Age Structure Shift | 1284634 | 42.20% | 3042402 |
| Philippines | Edentulism | Prevalence | Attributed to Population Growth | 1734235 | 57.00% | 3042402 |
| Philippines | Edentulism | Prevalence | Attributed to Epidemiologic Changes | 23533.6 | 0.80% | 3042402 |
| Philippines | Edentulism | Incidence | Attributed to Age Structure Shift | 97108.9 | 41.50% | 233829.8 |
| Philippines | Edentulism | Incidence | Attributed to Population Growth | 138740.8 | 59.30% | 233829.8 |
| Philippines | Edentulism | Incidence | Attributed to Epidemiologic Changes | -2019.92 | -0.90% | 233829.8 |
| Philippines | Edentulism | DALYs | Attributed to Age Structure Shift | 35079.43 | 42.00% | 83529.94 |
| Philippines | Edentulism | DALYs | Attributed to Population Growth | 47615.26 | 57.00% | 83529.94 |
| Philippines | Edentulism | DALYs | Attributed to Epidemiologic Changes | 835.2518 | 1.00% | 83529.94 |
| Philippines | Other oral disorders | Prevalence | Attributed to Age Structure Shift | 135965.1 | 13.30% | 1022847 |
| Philippines | Other oral disorders | Prevalence | Attributed to Population Growth | 885601.7 | 86.60% | 1022847 |
| Philippines | Other oral disorders | Prevalence | Attributed to Epidemiologic Changes | 1279.76 | 0.10% | 1022847 |
| Philippines | Other oral disorders | Incidence | Attributed to Age Structure Shift | NA | NA% | NA |
| Philippines | Other oral disorders | Incidence | Attributed to Population Growth | NA | NA% | NA |
| Philippines | Other oral disorders | Incidence | Attributed to Epidemiologic Changes | NA | NA% | NA |
| Philippines | Other oral disorders | DALYs | Attributed to Age Structure Shift | 3738.3 | 12.60% | 29641.59 |
| Philippines | Other oral disorders | DALYs | Attributed to Population Growth | 25744.66 | 86.90% | 29641.59 |
| Philippines | Other oral disorders | DALYs | Attributed to Epidemiologic Changes | 158.6322 | 0.50% | 29641.59 |
| Singapore | Main Oral disorders | Prevalence | Attributed to Age Structure Shift | 116898.2 | 11.80% | 991416 |
| Singapore | Main Oral disorders | Prevalence | Attributed to Population Growth | 915919.2 | 92.40% | 991416 |
| Singapore | Main Oral disorders | Prevalence | Attributed to Epidemiologic Changes | -41401.5 | -4.20% | 991416 |
| Singapore | Main Oral disorders | Incidence | Attributed to Age Structure Shift | -384476 | -42.10% | 912841.1 |
| Singapore | Main Oral disorders | Incidence | Attributed to Population Growth | 1297356 | 142.10% | 912841.1 |
| Singapore | Main Oral disorders | Incidence | Attributed to Epidemiologic Changes | -38.9519 | 0.00% | 912841.1 |
| Singapore | Main Oral disorders | DALYs | Attributed to Age Structure Shift | 3471.999 | 39.30% | 8835.789 |
| Singapore | Main Oral disorders | DALYs | Attributed to Population Growth | 5550.211 | 62.80% | 8835.789 |
| Singapore | Main Oral disorders | DALYs | Attributed to Epidemiologic Changes | -186.42 | -2.10% | 8835.789 |
| Singapore | Caries of deciduous teeth | Prevalence | Attributed to Age Structure Shift | -61664.9 | -193.90% | 31794.87 |
| Singapore | Caries of deciduous teeth | Prevalence | Attributed to Population Growth | 112416.9 | 353.60% | 31794.87 |
| Singapore | Caries of deciduous teeth | Prevalence | Attributed to Epidemiologic Changes | -18957.1 | -59.60% | 31794.87 |
| Singapore | Caries of deciduous teeth | Incidence | Attributed to Age Structure Shift | -198115 | -133.10% | 148866.7 |
| Singapore | Caries of deciduous teeth | Incidence | Attributed to Population Growth | 336688.9 | 226.20% | 148866.7 |
| Singapore | Caries of deciduous teeth | Incidence | Attributed to Epidemiologic Changes | 10292.72 | 6.90% | 148866.7 |
| Singapore | Caries of deciduous teeth | DALYs | Attributed to Age Structure Shift | -23.6329 | -191.50% | 12.34208 |
| Singapore | Caries of deciduous teeth | DALYs | Attributed to Population Growth | 43.07153 | 349.00% | 12.34208 |
| Singapore | Caries of deciduous teeth | DALYs | Attributed to Epidemiologic Changes | -7.09657 | -57.50% | 12.34208 |
| Singapore | Caries of permanent teeth | Prevalence | Attributed to Age Structure Shift | -67352.9 | -17.70% | 380630.3 |
| Singapore | Caries of permanent teeth | Prevalence | Attributed to Population Growth | 492576.3 | 129.40% | 380630.3 |
| Singapore | Caries of permanent teeth | Prevalence | Attributed to Epidemiologic Changes | -44593.1 | -11.70% | 380630.3 |
| Singapore | Caries of permanent teeth | Incidence | Attributed to Age Structure Shift | -208635 | -29.50% | 708252.6 |
| Singapore | Caries of permanent teeth | Incidence | Attributed to Population Growth | 928326.7 | 131.10% | 708252.6 |
| Singapore | Caries of permanent teeth | Incidence | Attributed to Epidemiologic Changes | -11438.7 | -1.60% | 708252.6 |
| Singapore | Caries of permanent teeth | DALYs | Attributed to Age Structure Shift | -72.5279 | -19.40% | 373.6424 |
| Singapore | Caries of permanent teeth | DALYs | Attributed to Population Growth | 488.0526 | 130.60% | 373.6424 |
| Singapore | Caries of permanent teeth | DALYs | Attributed to Epidemiologic Changes | -41.8823 | -11.20% | 373.6424 |
| Singapore | Periodontal diseases | Prevalence | Attributed to Age Structure Shift | 224991.4 | 41.40% | 544060.7 |
| Singapore | Periodontal diseases | Prevalence | Attributed to Population Growth | 291395.4 | 53.60% | 544060.7 |
| Singapore | Periodontal diseases | Prevalence | Attributed to Epidemiologic Changes | 27673.95 | 5.10% | 544060.7 |
| Singapore | Periodontal diseases | Incidence | Attributed to Age Structure Shift | 15867.24 | 35.30% | 44916.29 |
| Singapore | Periodontal diseases | Incidence | Attributed to Population Growth | 26818.52 | 59.70% | 44916.29 |
| Singapore | Periodontal diseases | Incidence | Attributed to Epidemiologic Changes | 2230.527 | 5.00% | 44916.29 |
| Singapore | Periodontal diseases | DALYs | Attributed to Age Structure Shift | 1450.089 | 41.20% | 3520.061 |
| Singapore | Periodontal diseases | DALYs | Attributed to Population Growth | 1897.84 | 53.90% | 3520.061 |
| Singapore | Periodontal diseases | DALYs | Attributed to Epidemiologic Changes | 172.1323 | 4.90% | 3520.061 |
| Singapore | Edentulism | Prevalence | Attributed to Age Structure Shift | 71402.28 | 61.40% | 116252.6 |
| Singapore | Edentulism | Prevalence | Attributed to Population Growth | 56762.29 | 48.80% | 116252.6 |
| Singapore | Edentulism | Prevalence | Attributed to Epidemiologic Changes | -11912 | -10.20% | 116252.6 |
| Singapore | Edentulism | Incidence | Attributed to Age Structure Shift | 6406.964 | 59.30% | 10805.56 |
| Singapore | Edentulism | Incidence | Attributed to Population Growth | 5522.098 | 51.10% | 10805.56 |
| Singapore | Edentulism | Incidence | Attributed to Epidemiologic Changes | -1123.51 | -10.40% | 10805.56 |
| Singapore | Edentulism | DALYs | Attributed to Age Structure Shift | 1939.596 | 61.00% | 3179.314 |
| Singapore | Edentulism | DALYs | Attributed to Population Growth | 1553.126 | 48.90% | 3179.314 |
| Singapore | Edentulism | DALYs | Attributed to Epidemiologic Changes | -313.409 | -9.90% | 3179.314 |
| Singapore | Other oral disorders | Prevalence | Attributed to Age Structure Shift | 6911.206 | 11.40% | 60855.41 |
| Singapore | Other oral disorders | Prevalence | Attributed to Population Growth | 54001.5 | 88.70% | 60855.41 |
| Singapore | Other oral disorders | Prevalence | Attributed to Epidemiologic Changes | -57.2945 | -0.10% | 60855.41 |
| Singapore | Other oral disorders | Incidence | Attributed to Age Structure Shift | NA | NA% | NA |
| Singapore | Other oral disorders | Incidence | Attributed to Population Growth | NA | NA% | NA |
| Singapore | Other oral disorders | Incidence | Attributed to Epidemiologic Changes | NA | NA% | NA |
| Singapore | Other oral disorders | DALYs | Attributed to Age Structure Shift | 178.4738 | 10.20% | 1750.429 |
| Singapore | Other oral disorders | DALYs | Attributed to Population Growth | 1568.121 | 89.60% | 1750.429 |
| Singapore | Other oral disorders | DALYs | Attributed to Epidemiologic Changes | 3.83497 | 0.20% | 1750.429 |
| Thailand | Main Oral disorders | Prevalence | Attributed to Age Structure Shift | 3474287 | 46.50% | 7476410 |
| Thailand | Main Oral disorders | Prevalence | Attributed to Population Growth | 5040028 | 67.40% | 7476410 |
| Thailand | Main Oral disorders | Prevalence | Attributed to Epidemiologic Changes | -1037905 | -13.90% | 7476410 |
| Thailand | Main Oral disorders | Incidence | Attributed to Age Structure Shift | -6994330 | 709.10% | -986375 |
| Thailand | Main Oral disorders | Incidence | Attributed to Population Growth | 5001819 | -507.10% | -986375 |
| Thailand | Main Oral disorders | Incidence | Attributed to Epidemiologic Changes | 1006136 | -102.00% | -986375 |
| Thailand | Main Oral disorders | DALYs | Attributed to Age Structure Shift | 96030.68 | 80.50% | 119220 |
| Thailand | Main Oral disorders | DALYs | Attributed to Population Growth | 28437.91 | 23.90% | 119220 |
| Thailand | Main Oral disorders | DALYs | Attributed to Epidemiologic Changes | -5248.56 | -4.40% | 119220 |
| Thailand | Caries of deciduous teeth | Prevalence | Attributed to Age Structure Shift | -3021099 | 107.60% | -2806947 |
| Thailand | Caries of deciduous teeth | Prevalence | Attributed to Population Growth | 705198.6 | -25.10% | -2806947 |
| Thailand | Caries of deciduous teeth | Prevalence | Attributed to Epidemiologic Changes | -491046 | 17.50% | -2806947 |
| Thailand | Caries of deciduous teeth | Incidence | Attributed to Age Structure Shift | -6098272 | 140.30% | -4347491 |
| Thailand | Caries of deciduous teeth | Incidence | Attributed to Population Growth | 1435337 | -33.00% | -4347491 |
| Thailand | Caries of deciduous teeth | Incidence | Attributed to Epidemiologic Changes | 315444.5 | -7.30% | -4347491 |
| Thailand | Caries of deciduous teeth | DALYs | Attributed to Age Structure Shift | -1153.25 | 107.70% | -1070.44 |
| Thailand | Caries of deciduous teeth | DALYs | Attributed to Population Growth | 269.2079 | -25.10% | -1070.44 |
| Thailand | Caries of deciduous teeth | DALYs | Attributed to Epidemiologic Changes | -186.396 | 17.40% | -1070.44 |
| Thailand | Caries of permanent teeth | Prevalence | Attributed to Age Structure Shift | 2334011 | 53.50% | 4359650 |
| Thailand | Caries of permanent teeth | Prevalence | Attributed to Population Growth | 3353576 | 76.90% | 4359650 |
| Thailand | Caries of permanent teeth | Prevalence | Attributed to Epidemiologic Changes | -1327938 | -30.50% | 4359650 |
| Thailand | Caries of permanent teeth | Incidence | Attributed to Age Structure Shift | -1373190 | -50.90% | 2696894 |
| Thailand | Caries of permanent teeth | Incidence | Attributed to Population Growth | 3422676 | 126.90% | 2696894 |
| Thailand | Caries of permanent teeth | Incidence | Attributed to Epidemiologic Changes | 647408 | 24.00% | 2696894 |
| Thailand | Caries of permanent teeth | DALYs | Attributed to Age Structure Shift | 2029.281 | 50.10% | 4053.426 |
| Thailand | Caries of permanent teeth | DALYs | Attributed to Population Growth | 3297.047 | 81.30% | 4053.426 |
| Thailand | Caries of permanent teeth | DALYs | Attributed to Epidemiologic Changes | -1272.9 | -31.40% | 4053.426 |
| Thailand | Periodontal diseases | Prevalence | Attributed to Age Structure Shift | 4617811 | 65.00% | 7102566 |
| Thailand | Periodontal diseases | Prevalence | Attributed to Population Growth | 1271989 | 17.90% | 7102566 |
| Thailand | Periodontal diseases | Prevalence | Attributed to Epidemiologic Changes | 1212766 | 17.10% | 7102566 |
| Thailand | Periodontal diseases | Incidence | Attributed to Age Structure Shift | 314216.9 | 62.70% | 501127.1 |
| Thailand | Periodontal diseases | Incidence | Attributed to Population Growth | 112009.9 | 22.40% | 501127.1 |
| Thailand | Periodontal diseases | Incidence | Attributed to Epidemiologic Changes | 74900.25 | 14.90% | 501127.1 |
| Thailand | Periodontal diseases | DALYs | Attributed to Age Structure Shift | 29493.54 | 64.80% | 45514.9 |
| Thailand | Periodontal diseases | DALYs | Attributed to Population Growth | 8234.986 | 18.10% | 45514.9 |
| Thailand | Periodontal diseases | DALYs | Attributed to Epidemiologic Changes | 7786.379 | 17.10% | 45514.9 |
| Thailand | Edentulism | Prevalence | Attributed to Age Structure Shift | 2283620 | 101.40% | 2252926 |
| Thailand | Edentulism | Prevalence | Attributed to Population Growth | 404271 | 17.90% | 2252926 |
| Thailand | Edentulism | Prevalence | Attributed to Epidemiologic Changes | -434965 | -19.30% | 2252926 |
| Thailand | Edentulism | Incidence | Attributed to Age Structure Shift | 162915.5 | 99.90% | 163094.2 |
| Thailand | Edentulism | Incidence | Attributed to Population Growth | 31795.52 | 19.50% | 163094.2 |
| Thailand | Edentulism | Incidence | Attributed to Epidemiologic Changes | -31616.8 | -19.40% | 163094.2 |
| Thailand | Edentulism | DALYs | Attributed to Age Structure Shift | 61634.88 | 101.10% | 60973.58 |
| Thailand | Edentulism | DALYs | Attributed to Population Growth | 11033.44 | 18.10% | 60973.58 |
| Thailand | Edentulism | DALYs | Attributed to Epidemiologic Changes | -11694.7 | -19.20% | 60973.58 |
| Thailand | Other oral disorders | Prevalence | Attributed to Age Structure Shift | 156395.8 | 44.50% | 351474.9 |
| Thailand | Other oral disorders | Prevalence | Attributed to Population Growth | 193807.4 | 55.10% | 351474.9 |
| Thailand | Other oral disorders | Prevalence | Attributed to Epidemiologic Changes | 1271.685 | 0.40% | 351474.9 |
| Thailand | Other oral disorders | Incidence | Attributed to Age Structure Shift | NA | NA% | NA |
| Thailand | Other oral disorders | Incidence | Attributed to Population Growth | NA | NA% | NA |
| Thailand | Other oral disorders | Incidence | Attributed to Epidemiologic Changes | NA | NA% | NA |
| Thailand | Other oral disorders | DALYs | Attributed to Age Structure Shift | 4026.232 | 41.30% | 9748.558 |
| Thailand | Other oral disorders | DALYs | Attributed to Population Growth | 5603.235 | 57.50% | 9748.558 |
| Thailand | Other oral disorders | DALYs | Attributed to Epidemiologic Changes | 119.0921 | 1.20% | 9748.558 |
| Viet Nam | Main Oral disorders | Prevalence | Attributed to Age Structure Shift | 1712563 | 12.80% | 13380557 |
| Viet Nam | Main Oral disorders | Prevalence | Attributed to Population Growth | 14233252 | 106.40% | 13380557 |
| Viet Nam | Main Oral disorders | Prevalence | Attributed to Epidemiologic Changes | -2565258 | -19.20% | 13380557 |
| Viet Nam | Main Oral disorders | Incidence | Attributed to Age Structure Shift | -7294782 | -65.50% | 11135109 |
| Viet Nam | Main Oral disorders | Incidence | Attributed to Population Growth | 18250756 | 163.90% | 11135109 |
| Viet Nam | Main Oral disorders | Incidence | Attributed to Epidemiologic Changes | 179134.7 | 1.60% | 11135109 |
| Viet Nam | Main Oral disorders | DALYs | Attributed to Age Structure Shift | 55182.46 | 52.20% | 105668.2 |
| Viet Nam | Main Oral disorders | DALYs | Attributed to Population Growth | 63655.28 | 60.20% | 105668.2 |
| Viet Nam | Main Oral disorders | DALYs | Attributed to Epidemiologic Changes | -13169.5 | -12.50% | 105668.2 |
| Viet Nam | Caries of deciduous teeth | Prevalence | Attributed to Age Structure Shift | -3901706 | 496.00% | -786637 |
| Viet Nam | Caries of deciduous teeth | Prevalence | Attributed to Population Growth | 3217019 | -409.00% | -786637 |
| Viet Nam | Caries of deciduous teeth | Prevalence | Attributed to Epidemiologic Changes | -101950 | 13.00% | -786637 |
| Viet Nam | Caries of deciduous teeth | Incidence | Attributed to Age Structure Shift | -8341193 | 753.70% | -1106718 |
| Viet Nam | Caries of deciduous teeth | Incidence | Attributed to Population Growth | 7228492 | -653.10% | -1106718 |
| Viet Nam | Caries of deciduous teeth | Incidence | Attributed to Epidemiologic Changes | 5982.747 | -0.50% | -1106718 |
| Viet Nam | Caries of deciduous teeth | DALYs | Attributed to Age Structure Shift | -1495.01 | 551.50% | -271.071 |
| Viet Nam | Caries of deciduous teeth | DALYs | Attributed to Population Growth | 1232.1 | -454.50% | -271.071 |
| Viet Nam | Caries of deciduous teeth | DALYs | Attributed to Epidemiologic Changes | -8.15684 | 3.00% | -271.071 |
| Viet Nam | Caries of permanent teeth | Prevalence | Attributed to Age Structure Shift | 3120873 | 34.00% | 9188210 |
| Viet Nam | Caries of permanent teeth | Prevalence | Attributed to Population Growth | 9336916 | 101.60% | 9188210 |
| Viet Nam | Caries of permanent teeth | Prevalence | Attributed to Epidemiologic Changes | -3269580 | -35.60% | 9188210 |
| Viet Nam | Caries of permanent teeth | Incidence | Attributed to Age Structure Shift | 731306.1 | 6.30% | 11601107 |
| Viet Nam | Caries of permanent teeth | Incidence | Attributed to Population Growth | 10766309 | 92.80% | 11601107 |
| Viet Nam | Caries of permanent teeth | Incidence | Attributed to Epidemiologic Changes | 103492.4 | 0.90% | 11601107 |
| Viet Nam | Caries of permanent teeth | DALYs | Attributed to Age Structure Shift | 2962.893 | 32.80% | 9026.801 |
| Viet Nam | Caries of permanent teeth | DALYs | Attributed to Population Growth | 9241.757 | 102.40% | 9026.801 |
| Viet Nam | Caries of permanent teeth | DALYs | Attributed to Epidemiologic Changes | -3177.85 | -35.20% | 9026.801 |
| Viet Nam | Periodontal diseases | Prevalence | Attributed to Age Structure Shift | 2364797 | 45.00% | 5249729 |
| Viet Nam | Periodontal diseases | Prevalence | Attributed to Population Growth | 1779992 | 33.90% | 5249729 |
| Viet Nam | Periodontal diseases | Prevalence | Attributed to Epidemiologic Changes | 1104941 | 21.00% | 5249729 |
| Viet Nam | Periodontal diseases | Incidence | Attributed to Age Structure Shift | 217625 | 42.10% | 517402.6 |
| Viet Nam | Periodontal diseases | Incidence | Attributed to Population Growth | 183510.9 | 35.50% | 517402.6 |
| Viet Nam | Periodontal diseases | Incidence | Attributed to Epidemiologic Changes | 116266.6 | 22.50% | 517402.6 |
| Viet Nam | Periodontal diseases | DALYs | Attributed to Age Structure Shift | 15324.79 | 45.30% | 33828.99 |
| Viet Nam | Periodontal diseases | DALYs | Attributed to Population Growth | 11546.41 | 34.10% | 33828.99 |
| Viet Nam | Periodontal diseases | DALYs | Attributed to Epidemiologic Changes | 6957.787 | 20.60% | 33828.99 |
| Viet Nam | Edentulism | Prevalence | Attributed to Age Structure Shift | 1182984 | 81.00% | 1461031 |
| Viet Nam | Edentulism | Prevalence | Attributed to Population Growth | 896265 | 61.30% | 1461031 |
| Viet Nam | Edentulism | Prevalence | Attributed to Epidemiologic Changes | -618217 | -42.30% | 1461031 |
| Viet Nam | Edentulism | Incidence | Attributed to Age Structure Shift | 97479.4 | 79.00% | 123317.4 |
| Viet Nam | Edentulism | Incidence | Attributed to Population Growth | 72444.99 | 58.70% | 123317.4 |
| Viet Nam | Edentulism | Incidence | Attributed to Epidemiologic Changes | -46607 | -37.80% | 123317.4 |
| Viet Nam | Edentulism | DALYs | Attributed to Age Structure Shift | 32654.57 | 80.90% | 40359.66 |
| Viet Nam | Edentulism | DALYs | Attributed to Population Growth | 24667.13 | 61.10% | 40359.66 |
| Viet Nam | Edentulism | DALYs | Attributed to Epidemiologic Changes | -16962 | -42.00% | 40359.66 |
| Viet Nam | Other oral disorders | Prevalence | Attributed to Age Structure Shift | 206948.3 | 26.40% | 785240 |
| Viet Nam | Other oral disorders | Prevalence | Attributed to Population Growth | 582595.2 | 74.20% | 785240 |
| Viet Nam | Other oral disorders | Prevalence | Attributed to Epidemiologic Changes | -4303.52 | -0.50% | 785240 |
| Viet Nam | Other oral disorders | Incidence | Attributed to Age Structure Shift | NA | NA% | NA |
| Viet Nam | Other oral disorders | Incidence | Attributed to Population Growth | NA | NA% | NA |
| Viet Nam | Other oral disorders | Incidence | Attributed to Epidemiologic Changes | NA | NA% | NA |
| Viet Nam | Other oral disorders | DALYs | Attributed to Age Structure Shift | 5735.226 | 25.20% | 22723.81 |
| Viet Nam | Other oral disorders | DALYs | Attributed to Population Growth | 16967.88 | 74.70% | 22723.81 |
| Viet Nam | Other oral disorders | DALYs | Attributed to Epidemiologic Changes | 20.71193 | 0.10% | 22723.81 |
